# Consulting people who use cannabis to plan a regulatory trial on non-medical cannabis sales in pharmacies

**DOI:** 10.1186/s40900-025-00791-3

**Published:** 2025-10-24

**Authors:** Anna Irina Schibli, F. H., N. N., A. S., Kathrin Bieri, Kevin Selby, Marie-Anne Durand, Reto Auer, Beatrice Metry

**Affiliations:** 1 Institute of Primary Health Care (BIHAM), University of Bern, Mittelstrasse 43, Bern, 3012 Switzerland; 2 Advisory group co-authors, anonymised at their request, Bern, Switzerland; 3 Department of Clinical Research (DCR), Faculty of Medicine, University of Bern, Bern, Switzerland; 4 Unisanté, University Center for Primary Care and Public Health, Department of Ambulatory Care, University of Lausanne, Lausanne, Switzerland; 5 The Dartmouth Institute for Health Policy & Clinical Practice, Dartmouth College, Lebanon, New Hampshire USA

**Keywords:** Patient and Public Involvement (PPI), Cannabis regulation, Advisory group, Consultation exercise

## Abstract

**Background:**

Switzerland has taken a different path to cannabis regulation than other countries. A 2021 law allows regulatory experiments on the production and sale of cannabis for non-medical purposes. The research team planned a randomised controlled trial (RCT) to test how selling cannabis in pharmacies affects users’ health and consumption habits. The study intervention also included counselling on risk reduction, such as smoking cessation. The research team aimed to incorporate the perspectives of people who use cannabis into the study design, despite challenges related to stigma and legal constraints.

**Methods:**

When planning the RCT, the research team mandated an external researcher to form an advisory group with regular users of non-medical cannabis. This researcher used convenience sampling (including snowball sampling) to recruit people who use cannabis, considering age, self-reported gender and cannabis use frequency. She conducted an individual interview, followed by iterative group discussions. She summarised the results into reports and sent them to the research group, who considered the results during study planning and formulated topics to be discussed in the next advisory group meeting.

**Results:**

In a two- and half-year long process (November 2021 to March 2024), eight people who use cannabis provided iterative feedback that informed the development of the study intervention. Based on their feedback, the research team expanded the product selection to include cannabis resin alongside cannabis flowers. The group’s feedback also led the research team to make several changes to the sales process in pharmacies. While the members of the advisory group expressed that much of their feedback had been considered, they noted that some key aspects — such as product pricing — had not been implemented. Legal and political constraints limited the research team’s ability to implement advisory group feedback.

**Conclusions:**

This experience showed that gathering user input is feasible, even in highly regulated, stigmatized contexts. Including the perspectives of people who use cannabis in the trial set-up allowed the research team to adapt the study intervention. Where they could not adapt the study intervention, it helped them prepare for possible reactions from future study participants and disseminate their findings to other stakeholders. Future research should test the feasibility and benefits of such activities throughout all research stages.

**Supplementary Information:**

The online version contains supplementary material available at 10.1186/s40900-025-00791-3.

## Abstract (plain language summary)

**Cannabis in Switzerland:**

Switzerland has taken a different path to cannabis policy than other countries. In 2021, the country adopted a law allowing researchers to test how cannabis could be produced and sold for non-medical (so-called ‘recreational’) use. The intent was to test different models before deciding on a law for legal cannabis sales. In Switzerland, where the production and sale of cannabis is still illegal, people who use cannabis face stigma and researchers running such trials have to follow strict laws.

**About our study:**

The research team aimed to test how selling non-medical cannabis in pharmacies would change the health and use habits of people who use cannabis. While planning this study, they wanted to gather the perspectives of people who use cannabis to improve the trial. The intention was to adapt the trial as much as possible to the needs of people who use cannabis while complying with the law.

**What the research team did:**

•They invited people who use cannabis from the city of Bern to form an advisory group who met regularly.

•An external researcher interviewed each person and held five group discussions.

•She summarised their feedback into reports. She made sure to protect their identities by anonymising their statements.

•The research team used this feedback to make changes to the study. For example, they added cannabis resin (hashish) as a purchase option in pharmacies, alongside cannabis flowers. However, in some cases they had to follow the law, even if it went against what participants wanted.

**Challenges and what they learned:**

The research team learned that it is possible to involve people who use cannabis in research, even though cannabis is still illegal and strict rules apply. They found the feedback from people who use cannabis valuable. Although they could not meet all the advisory group’s needs, they were able to make many changes to the study.

**Supplementary Information:**

The online version contains supplementary material available at 10.1186/s40900-025-00791-3.

## Introduction

Cannabis policy is evolving rapidly in many European countries [1]. In this complex process with many stakeholders involved, there is growing demand to involve people who use cannabis. Patient and Public Involvement (PPI)^1^ aims to involve those who are affected by a research topic using a collaborative approach [3]. This is not only a matter of justice, but also because the developments become more relevant and better suited to people’s needs [4]. By incorporating the needs and preferences of those affected, PPI has been reported to improve recruitment success and trial adherence [5, 6]. In this article, We^2^ describe how the research team sought to involve people who use cannabis in the set-up of a large regulatory trial. The trial tested the health and social effects of selling non-medical cannabis in pharmacies within the Swiss legal context.

When it comes to investigating models for future cannabis policy, Switzerland has taken an unusual approach: Pilot trials are to test how controlled cannabis sales impact user behaviour and health outcomes. The evidence generated by the trials will inform policymakers and other stakeholders for future cannabis policies. The Swiss Narcotics Act was amended in 2021, permitting these trials under strictly regulated conditions for a ten-year period [7]. The Safer Cannabis - Research in Pharmacies Randomised Controlled Trial (SCRIPT) is one of these pilot trials.

The involved cities initiated discussions for the conduct of SCRIPT as early as 2012. They involved researchers from the University of Bern. A first version of the trial was submitted in 2017. The Federal Office of Public Health (FOPH) rejected the proposal for not complying with regulations. Though legal advisers involved in SCRIPT argued that the project adhered to regulations, the FOPH interpreted the law differently and withheld approval. As a result, the project was put on hold. Planning resumed in 2021 after the amendment of the Narcotics Act.

The SCRIPT trial aims to test the health and social effects of allowing people who use cannabis for non-medical purposes to buy regulated cannabis in pharmacies. The study intends to include 1091 participants from the cities of Bern, Biel and Lucerne. At the end of the baseline visit, participants are randomly assigned to the intervention or control group. The intervention group can immediately buy cannabis in pharmacies and receive counselling; the control group waits six months during which they likely continue buying cannabis on the illicit market. The trial intervention has two main components. First, participants can buy various cannabis products from pharmacies. Second, the trial includes dedicated counselling on smoking cessation and risk-reduction. SCRIPT and the law allowing such trials draw from the principle of harm reduction and risk minimisation, which is one of the four pillars of Swiss drug policy [8]. The primary outcome in SCRIPT is abstinence from smoking tobacco and cannabis. Secondary outcomes include further health and social outcomes.

### Patient and public involvement in a regulatory context

The term “Patient and Public Involvement” as we understand it refers to any form of involvement of people affected by the topic [9]. Bammer [10] differentiates five different levels of participation for complex research problems, such as drug regulation work. The levels range from informing the public, to empowering people to conduct their own research. While Bammer considers each as legitimate, the different levels of participation must be carefully described to match the circumstances. While the approach we describe here lies at the ‘consult’ level, other approaches operate on a higher participatory level. An example of those are the consumer-led cannabis social clubs [11].

Involving people who use cannabis in substance regulation trials presented the research team with unique challenges. Firstly, regulatory requirements for pilot trials limited flexibility in study design [12]. Secondly, the stigma surrounding cannabis use can lead to societal judgement. People who use cannabis might be hesitant to talk about their use, even to healthcare professionals [13]. This required strategies to protect the anonymity of people who use cannabis [14]. Thirdly, the research team had to balance the perspectives of people who use cannabis with broader public health priorities, such as protecting minors [15]. Lastly, the politically sensitive environment around cannabis regulation inherently shaped the way the research team conducted and reported this work. The research team decided to convey a neutral stance, aligning neither with pro- nor anti-regulation positions. Such dynamics have been described by Sznitman and colleagues [16] and show that more than purely scientific considerations, social forces also influence the production of evidence for Swiss cannabis regulation.

### Objectives of this article

In this article, we provide a detailed account of the PPI process for the design of the protocol of the SCRIPT trial. We reflect on the adaptions made to the study intervention based on advisory group feedback, changes that could not be made, and the unique challenges the research team was presented with.

## Methods

### Aims

The consultation of the advisory group^3^ had two central aims:

1. To adapt the cannabis sales and the accompanying counselling interventions to the needs of people who use cannabis.
2. To optimise recruitment and support the use of the trial intervention by future study participants.

### Framework for the PPI

The research team launched the PPI activities in 2021, nine years after initial discussions about SCRIPT began. When co-author RA became principal investigator in 2020, the project was already well advanced. Political stakeholders of the involved cities had decided that pharmacies would serve as a point of sale from 2012 to 2016 and decided to pursue this sales location after 2021. The legal framework defined many research areas, such as points of sale, maximum quantity of product sold per person, product quality including THC/CBD limits, packaging and advertising restrictions, and participant eligibility – among other regulated areas [18].

Given these constraints, the research team searched for a pragmatic approach to involve people who use cannabis. The areas where they saw the greatest potential for their input were the sales process, product selection, and communication with future study participants. They also explored topics related to the study intervention in areas where the law limited advisory group influence. They aimed to anticipate the reactions from future study participants and bring the perspective of people who use cannabis to other stakeholders involved in the study planning, such as the involved cities and authorities, and other researchers planning such studies. They chose a model that aligns with Bammer’s ‘consult’ stage [10]. The group members thus had an advisory role, while the final decision-making authority remained with the research team.

### Recruitment

During the planning phase of the SCRIPT study in 2021, the research team recruited people who use non-medical cannabis of different genders and ages in the cantons of Bern and Lucerne. The team used four recruitment methods. First, they contacted people who had previously expressed an interest in the study. Second, they posted flyers at facilities offering anonymous drug-checking services. Third, they reached out to people from their professional networks who used cannabis, Finally, they used snowball sampling, allowing people who use cannabis to refer others.

They decided to exclude people who use cannabis for medical reasons and people who were politically or professionally committed to cannabis regulation. They aimed for a group size of eight to ten people because their previous research suggested this number gives enough variety and allows all members to participate [19].

### Declaration of consent and ethics application

The group members signed a Declaration of Consent in which they agreed to the use of their anonymised statements in reports and articles. They agreed to the terms of confidentiality and co-operation in the group. The PPI concept for SCRIPT was submitted to the Bern Cantonal Ethics Committee (KEK) for review, which stated that the research project fell outside the scope of the Swiss law on human research and that they were thus not responsible (declaration of non-responsibility of the 20th of June 2024, number Req-2021-00609).

### Funding

The project was initially financed by the City of Bern and internal funds available from the research team, with funding from the Swiss National Science Foundation (SNSF) secured in April 2023 and Tobacco Control Fund (TCF) in the autumn of 2023.

### Setting

Between November 2021 and March 2024, the qualitative researcher (BM) conducted a first individual Zoom interview with each eligible person, followed by five in-person group discussions. These one-hour individual interviews aimed to document each advisory group member’s preferences regarding cannabis use and purchasing habits. During these interviews, she provided a brief overview of the goals and practical aspects of the PPI project, focusing on pragmatic information about the SCRIPT study. The group discussions, held on the premises of the University of Bern, each lasted two hours.

BM worked exclusively with the advisory group and had no other role in the research project. She had no prior experience in cannabis regulation research. BM had led an advisory group for a research project linked to colorectal cancer screening [19]. In this present project, she acted as a moderator. She focused on gathering the advisory group’s input and conveying their views to the research team. The research team suggested the topics of the interviews and discussions. Being in regular exchange with the pharmacy teams, they made sure to integrate their questions and concerns. BM structured the interviews and discussions using a predefined questionnaire. The original German questionnaires are available in Additional File 2, the automatically translated English version in Additional File 3. She further explored the advisory group’s statements using the laddering technique [20]. She made a consistent effort to ensure that everyone’s opinion was heard, including those of quieter individuals. No consensus or decision was sought during the meetings. Instead, the laddering technique was used to address individual attitudes, experiences, and opinions.

BM digitally recorded all interviews. Only BM and the group members attended the sessions, except on two occasions when authors AIS and KB each participated as non-intervening observers. Apart from these exceptions, the research team never met the advisory group members in person during this period. This separation was based on prior PPI experience, where the research team believed that advisory group members spoke more openly when only BM and no other researchers were present.

### Evaluation and feedback cycle

BM digitally recorded each interview and discussion. After the sessions, she transcribed, coded and analysed the interviews and discussions with Mayring’s qualitative content analysis [21]. For coding and analyses, she used the qualitative data software “MAXQDA”. In the reports, she anonymised the statements of the group members and coded socio-demographic data to describe the characteristics of the advisory group.

She presented the findings in reports, distributed to the research team within two to four weeks after each session. To emphasise differing opinions, she indicated the number of people who shared each view in parentheses, making contradictory perspectives visible.

**Fig. 1:**
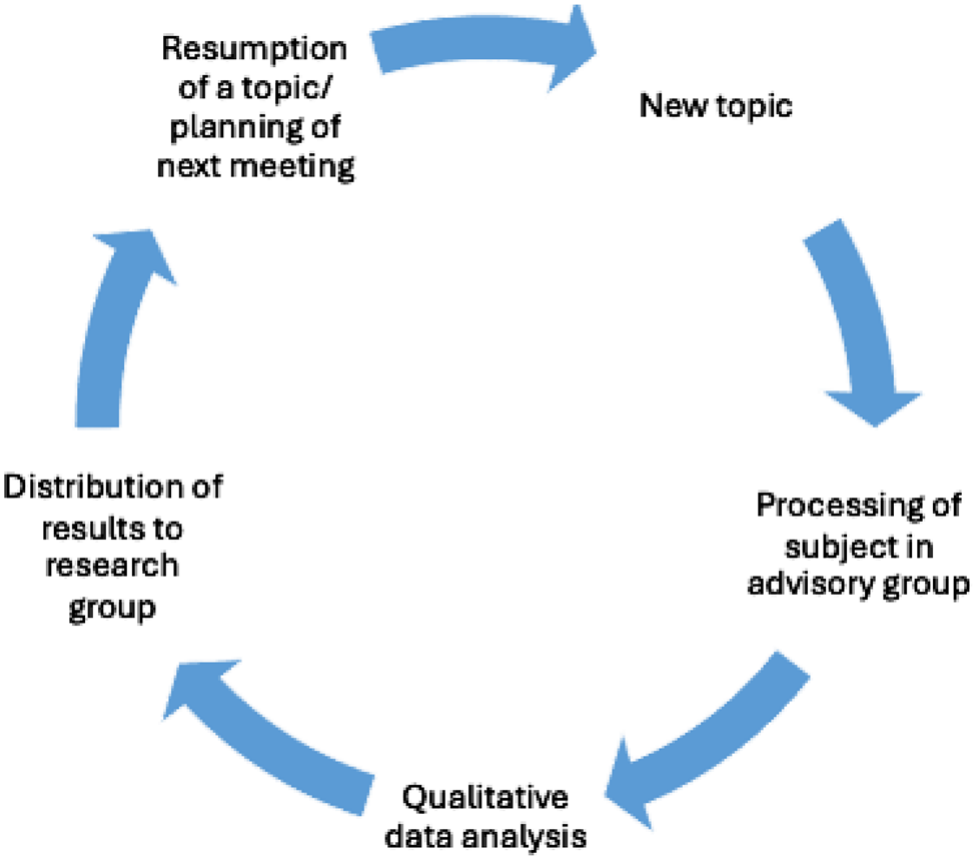
Procedure for the processing of topics by the advisory group and the connection to the research group

The research team read and discussed the report to adapt the study design accordingly. The research team then met with BM to discuss the report, inform about the proposed changes in the SCRIPT research protocol and draft the agenda for the next advisory group sessions. BM discussed the adjustments with the advisory group in the following sessions (Fig. 1). BM sent the reports to the advisory group members and invited them to comment. Furthermore, pharmacists, who play a crucial role in the trial, were regularly informed about advisory group findings. Finally, reports were also sent to further stakeholders involved in SCRIPT.

After completion of the trial planning phase in spring 2024, AIS attended an advisory group session led by BM and members of the advisory group reflected on the influence of the findings of the advisory group discussions on the study design. Author AIS presented a list of all advisory group feedback that BM had accumulated throughout the PPI project to both the advisory group and the research group. Each member of both groups reviewed the feedback and selected five aspects they considered most relevant for the study design. We summarise the most frequently selected aspects in the results sections of this article. We added the aspect of pricing policy retrospectively, as this aspect triggered some discussion after the start of the study.

In the last group discussion, AIS invited the advisory group members to contribute to the creation of this article. Three members (FH, NN and AS) agreed and provided both verbal and written feedback on this article. Together with AIS, they evaluated the extent to which the aspects reported back by the advisory group had been implemented. On their request, AIS anonymised them using only their initials for this article. Due to some co-authors’ limited knowledge of the English language, the initial draft of the article was written in German and later translated into English. The final draft version was translated back to German using an online translation tool (DeepL) with necessary adjustments made for clarity, for approval by those not fluent in English. We used the GRIPP2 long form checklist to guide the reporting of our PPI work (Additional File 1) [22].

### Compensation

The research team paid the advisory group members CHF 30 per hour (equating roughly to 30 USD) for their participation in interviews and discussions as well as for their contribution to this article. BM provided pastries and drinks during the advisory group meetings. In addition, the research team ensured the advisory group members could participate in the SCRIPT study, provided that they meet the eligibility criteria for the study.

## Results

### Advisory group members

Nine people who use cannabis were reached with the recruitment methods. All of them were included, so the group number matched the research team’s intended group size. The advisory group members had different ages and genders (Table 1). One person dropped out before the individual interviews began due to cessation of cannabis. While all group members were Swiss, various professions and consumption habits were represented. The group members had no prior PPI experience. Two members were recruited from the researchers’ environment, two via flyer distribution and four had already registered their interest in advance of the study. The recruiting method for one advisory group member is unclear, as this information was lost and we were unable to reach them after the advisory group meetings concluded.

**Table 1:** Advisory group members characteristics

Gender	• Women	3 (33%)
	• Men	5 (56%)
	• Non-binary	1 (11%)
Age	• 20–30 years	6 (67%)
	• 31–40 years	1 (11%)
	• 40–50 years	1 (11%)
	• > 50 years	1 (11%)
Occupation	• Student	3 (33%)
	• Logistics	2 (22%)
	• Finance	1 (11%)
	• Photography	1 (11%)
	• Graphics	1 (11%)
	• Fitness	1 (11%)
Frequency of use	• Daily/near-daily (≥ 20days/month)	6 (67%)
	• Non-daily users (weekly or monthly use)	3 (33%

### Meetings

Between November 2021 and March 2024, BM held one individual interview via zoom and five group discussions in person. The final group session was mainly to review the group’s work. Attendance at the group sessions varied (Table 2), with intermittently low attendance rates in the middle of the planning phase.

**Table 2:** Number of advisory group members attending the individual interviews and group discussions

	Individual interviews 2021	Group discussion 1 July 2022	Group discussion 2 March 2023	Group discussion 3 June 2023	Group discussion 4 November 2023	Group discussion 5 March 2024
Number of attendees	8	6	5	4	5	7

### Advisory group feedback and study design adjustments

The advisory group provided extensive feedback. Most of their comments focused on the study intervention and less on the study design. The most important feedback leading to key adjustments are summarised below and presented in Table 3. The reports summarising each interview and discussion in more detail can be found in Additional File 2 (German) and 3 (English).

**Table 3:** Left column: key feedback from the advisory group. Right column: impact on study intervention

Key feedback from the Advisory Group	Impact on Study Intervention
Include cannabis resin in addition to cannabis flowers.	• Expansion of the product range with cannabis resin, described as pressed pollen of low quality by advisory group.
Pharmacy staff should provide competent advice, including expert information on product effects, properties, and range. Ensure transparency about product details.	• Provision of an overview table with detailed information on the products and their ingredients.
Allow for visual and olfactory inspection of products.	• Sight glasses for cannabis flowers and resin, “scent tab” for the cannabis flowers to enable sampling.
Price per gram of cannabis flowers should not exceed CHF 10 to remain competitive with the illicit market.	• Despite feedback, product prices were set in the upper range compared to other Swiss cannabis trials.
Offer prevention talks discreetly; make information materials easily accessible. Staff should adopt a respectful tone and discreet approach.	• Low-threshold access to prevention materials was provided via the website and leaflet distribution at pharmacies. • Training of sales staff in prevention dialogue.
Reject the idea of sale through a local foundation for addiction aid due to its association with hard drugs. Consider organic shop, self-marketing by cannabis farmers, head or grow shops, cannabis bars, tobacco shops or kiosks as alternative sales points.	• The idea of sales by local foundation for addiction aid was rejected. • The pharmacy sales location was retained, although some advisory members found other sales locations to be more suitable.

### Product selection and presentation

The advisory group emphasised the importance of offering both cannabis flowers and cannabis resin. They viewed cannabis flowers as more of an everyday product, while cannabis resin was seen as a specialty item. Initially, the research team had only planned to offer cannabis flowers. Also, the advisory group emphasised the importance of products being visible and having a scent option which would help them assess the quality of the products sold. The advisory group felt the pricing of the product to be a very important topic. They suggested pricing cannabis flowers under CHF 10 per gram to be in line with the illicit cannabis market price. In the last advisory group meeting, members were invited to give feedback on the final products, the packaging, and the display containers, which they valued positively.

Adjustments:

- The research team added cannabis resin to the product selection. The advisory group, however, found the resin to be more similar to “pressed pollen” than traditional resin and therefore of low quality.
- The research team decided to equip the pharmacies with sight glasses for the display of flowers and cannabis resin. The containers for the cannabis flowers have a “scent tab”, which allows the products to be sampled.
- Contrary to the opinion of most advisory group members, the research team, in collaboration with further stakeholders, set product prices higher than the illicit market prices. The higher prices aimed at simulating a potential future tax on cannabis sales, with revenues supporting counselling and participant compensation during the trial. The advisory group found the prices too expensive.

### Sales process in pharmacies

The advisory group stressed the need for well-trained sales staff who could provide detailed product information. Some members voiced the request for the salespeople having personal experience with cannabis use. They requested transparency on product details such as ingredients, THC/CBD levels, and product conditions. As a positive aspect of the pharmacies, they mentioned destigmatisation.

Regarding the counselling by pharmacy personnel planned in the study, they wanted an unobtrusive approach on the part of the sales staff. The salesperson’s demeanour and tone of voice were crucial. According to the advisory group, information material on prevention should be easily available and offered unobtrusively.

Adjustments:

- The research team planned extended training for pharmacy staff, including a half-day session and an online course. The training covered information on cannabis, nicotine, tobacco use and methods of use, as well as guidance on smoking cessation counselling and safer use practices. The research team instructed the pharmacy staff to offer counselling in an empathetic and discreet manner.
- The research team provided the pharmacies with detailed product overviews and prevention materials for distribution to study participants. Additionally, the research team provided these materials on the SCRIPT website for study participants.

### Alternative points of sale

Due to political circumstances, alternative locations for cannabis sales other than pharmacies had to be considered. The research team proposed a sale through a local foundation that supports individuals with substance use disorders, a suggestion that the advisory group clearly rejected. They associated the foundation with other illicit substances, such as heroin, and were concerned that this association would increase the stigma surrounding people who use cannabis. The advisory group suggested organic food shops, self-marketing by cannabis farmers, head or grow shops, cannabis bars, tobacco shops or kiosks.

Adjustments:

- Because of the clear rejection of the proposal of cannabis sale through an addiction foundation, the research team did not pursue discussions with the involved cities to explore this possibility.
- The involved cities’ executive branches decided to limit cannabis sales to pharmacies. This decision was not left to the research team. Other sales locations suggested by the advisory group were not adopted. The advisory group found that the pharmacies were not optimal for the sale of non-medical cannabis due to the limited experience with such products. However, they hoped that pharmacy sales could help reduce stigma. In response, the research team adapted the training and pharmacy procedures as closely as possible to advisory group expectations, while acknowledging that most pharmacy sales staff lacked the “insider knowledge” the advisory group valued.

### Other adjustments

The research team made additional adjustments to the study intervention and one adjustment to the study design based on the advisory group’s feedback. These are not discussed in greater depth here, as they are not among the five most chosen aspects (see Methods section). Some changes worth highlighting are:

- The research team adapted the language and layout of instruction materials and website content, including leaflets on safer use and videos on risk reduction and smoking cessation.
- After some advisory group members expressed high trust in filters, the research team emphasised the limited risk-reduction benefits associated with filter use. The advisory group provided feedback on the information materials related to this topic.
- The research team instructed the pharmacies to provide a separate sales room for the sale of study products for confidentiality.
- The research team adjusted the product selection, e.g. excluding rectal suppositories from the product selection.
- The research team revised the informed consent language about contacting people in the participants’ environment, such as family members or primary care physicians. They clarified when such contact would occur (e.g. to track serious adverse events if the participant could not be reached). They also added an option allowing participants to choose not to name a contact person.

### Debriefing group meetings and the advisory role

Both the group moderator BM and the advisory group members perceived the advisory group discussions as harmonious, appreciating the neutral approach. Advisory group members reported a bonding experience within the group. Some advisory group members reported feeling empowered by contributing to the research design. When debriefing with our advisory group co-authors, they expressed satisfaction with their advisory role. However, some advisory group members expressed a desire to better understand the reasons why the research team did not implement specific recommendations. One member noted that meeting the research team directly would have enhanced the feeling of “being recognised as a person” within the process.

## Discussion

In developing a regulatory trial on cannabis sales in pharmacies “SCRIPT”, the research team consulted people who use cannabis to gather their perspectives. They gave numerous suggestions for the study intervention.

Advisory group feedback helped the research team see the “blind spots” in their reflections, aligning with Kristina Staley’s insight: “Researchers “don’t know what they don’t know” […]” [23]. The research team anticipated feedback regarding the importance of pharmacy staff having personal cannabis experience, for example. The research team did not expect the strong request for cannabis resin (hashish). They had initially planned to offer only cannabis flowers, since most advisory group members stated that this was their main form of consumption. Equally unexpected was the clear rejection of a local addiction foundation as a sales location. The research team had underestimated the stigma attached to it, largely due to the association with other illicit drugs such as heroin.

### Challenges in implementing feedback

The research team could not integrate all advisory group feedback into the study design. Regulatory restrictions limited their flexibility, for example, in the choice of sales locations. As a result, the sale of cannabis was ultimately confined to pharmacies. In Europe, cannabis sales in pharmacies are limited to medical cannabis [24], therefore staff have little experience with non-medical cannabis. Consequently, the research team could not fulfil the advisory group’s request for peer-led sales as could be found in Cannabis Social Clubs [11]. Also, the research team could not fully realise advisory group members’ preference for high-quality cannabis resin due to logistical and financial constraints in the production process of the cannabis producer.

The most controversial topic proved to be the pricing of study products. Advisory group members put great value on competitiveness with the illicit market. However, the research team pricing strategy was shaped by public health considerations, supported by further stakeholders in SCRIPT and pilot trials in planning in Switzerland at that time, and thus the inclusion of a simulated tax. This led the research team to set a price they considered realistic for future policies and comparable to the pricing of regulated cannabis internationally, which is typically higher than that of illicit cannabis. Advisory group members expressed frustration with the limitations on their influence in this area. This highlights the inherent tension between asking advisory group members their opinion on topics where their influence was in fact limited. In retrospect, more effort from the research team side to handle their concern would have been needed (see more on this limitation below).

In the advisory group discussions, stigma proved to be less a barrier than anticipated by the research team. Group moderator BM felt that the advisory group members spoke freely and confidently about their experiences. Anonymisation of advisory group members in the data reports allowed to protect their identities, providing reassurance for those concerned about professional or social repercussions. The diversified recruitment strategy helped to reach people who use cannabis despite many of them not disclosing their use publicly.

### Strengths and limitations

A key strength of this PPI project was the long duration of user involvement in the study set-up. While the design of the initial SCRIPT project started in 2012, the first interviews with participants of the advisory group were held in 2021, months after the law allowing such trials was enforced. This was three years before the first participant was included in SCRIPT in spring 2024. This allowed the comments and concerns from the advisory group to flow into grant proposal to funding agencies, the protocol submitted to the Ethics Commission and to the authorities, and the training of pharmacies.

Furthermore, the group composition was diverse in terms of gender and age. Additionally, due to early organisation of PPI funding, we were able to remunerate advisory group members.

A unique feature of our method was the group moderator outside the core research team. On the one hand, this helped to create a space where advisory group members felt comfortable expressing themselves freely. On the other hand, this prevented direct communication between the research team and the advisory group. Bammer [10] outlines the “promises” researchers make at various levels of involvement in complex research. At the “consult” level, the key commitment is to keep those consulted informed about how their input influences decision-making. This highlights the importance of providing regular feedback from the side of the research team, especially when the research team decided against advisory group recommendations.

Further limitations included intermittent low participation rate and the lost information on the recruitment method of one advisory group member. We mainly attributed the intermittent low participation rate to the delays in the study’s launch, discouraging some advisory group members. At the same time, the delays in study’s launch were an opportunity for the perspective of the advisory group to flow more extensively into the research protocol and study processes.

Some drug regulation research projects involve more diverse groups of public representatives with differing perspectives and values [10, 25]. The SCRIPT research team decided to consult people who use cannabis, but they did not include representatives from law enforcement or advocates for the protection of minors, for example. They felt that their own perspectives as well as the legal foundation for the trials had more blind spots concerning the consumer perspective than other public health or safety considerations.

The research team chose to exclude people active in cannabis advocacy from the advisory group. They wanted to avoid this advisory group being perceived as a sounding board for advocacy groups for cannabis regulation in a politically tense environment. Other researchers might have decided differently. The research team recognises that excluding experienced consumer advocates might have strengthened existing power imbalances between the advisory group and the research team [2] Also, Bammer [10] found that in contrast to higher participation levels, the involvement of groups with pro- or contra views on prohibition at the “consult” level was relatively unproblematic for maintaining a neutral stance. The research team plans further PPI activities during the conduct of the SCRIPT trial and explore further on the collaboration with members from advocacy groups.

### Applicability for future research

The model chosen by the research team was a pragmatic response to the constraints of the SCRIPT trial. It might be useful for researchers working under similar circumstances, whether in cannabis policy, drug regulation, or other complex fields of research. Researchers using this model should prioritise regular feedback to the advisory group on decision-making and the impact of their input.

Future research could test PPI activities in this field with more responsibility given to people who use cannabis. For future projects, the three advisory group authors suggested four domains where they could see people who use cannabis taking over more responsibility: Study products, sales processes, participant communication, and safer use recommendations. Extensive training that builds knowledge of the complex legal and political framework over time would be required. This would enable the study team to tailor the study intervention more effectively to cannabis users’ needs. However, as described above, this might demand dedicated thinking and planning and special thought on how opposing public perspectives (such as the protection of minors, for example) could be included.

## Conclusions

This PPI approach engaged people who use cannabis as consultants. They met regularly with a researcher external to the core research team to provide feedback on study design choices and further aspects decided by the research team. This approach proved to be both feasible and useful in the context of cannabis regulation research.

The advisory group provided valuable insights, making the research team recognise important blind spots. Their feedback allowed the research team to adapt the study intervention accordingly. While this experience highlighted the benefits of this advisory role, it also revealed the challenges of balancing regulatory constraints and public health priorities with advisory group expectations. The research team is planning to involve people who use cannabis in the later stages of the SCRIPT study.

## Supplementary Information

Below is the link to the electronic supplementary material.

Supplementary Material 1

Supplementary Material 2

Supplementary Material 3

## Data Availability

No datasets were generated or analysed during the current study.

## Abbreviations

SCRIPT: The Safer Cannabis - Research in Pharmacies Randomised Controlled Trial
FOPH: Federal Office of Public Health
PPI: Patient and Public Involvement
KEK: Cantonal Ethics Committee
TCF: Tobacco Control Fund
SNSF: Swiss National Science Foundation

## Footnotes

1 In the drug policy context, PPI is also referred to as “consumer engagement”. We use the more neutral term of “Patient Public Involvement” [2].

2 “we” in this manuscript refers to the authors of this article, including the advisory group authors. When referring specifically to the research group or the advisory group, they are identified as such.

3 Some authors in the English literature distinguish between the terms “discussion groups” for participatory exchange and “focus groups” for the collection of qualitative data [17]. Our project included both. We therefore use the term “advisory group”, which reflects the function of the group
