# Supplementary material for: Consulting people who use cannabis to plan a regulatory trial on non-medical cannabis sales in pharmacies
Source: Res Involv Engagem. 2025 Oct 24;11:124. doi: 10.1186/s40900-025-00791-3 (PMC12553168; doi:10.1186/s40900-025-00791-3)
Supplement: Supplementary file 2 — Supplementary Material 2 [file 40900_2025_791_MOESM2_ESM.pdf]

Auswertung der Einzelinterviews zu SCRIPT-2

Die Interviews wurden mit Cannabiskonsumierenden Personen geführt

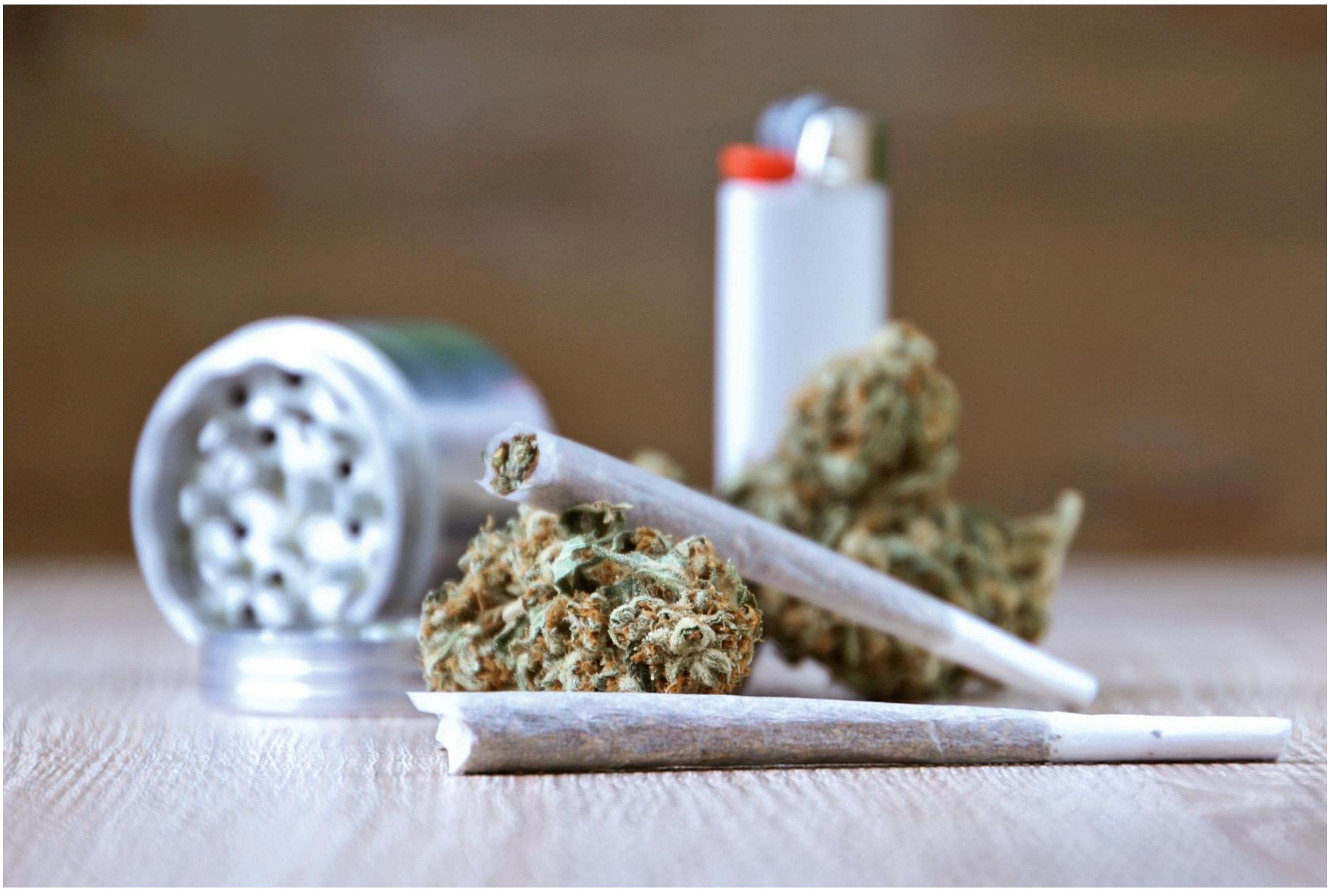

Beatrice Metry

Berner Institut für Hausarztmedizin

März 2022

Inhaltsverzeichnis

1 Einleitung 3
2 Methodisches Vorgehen 4
2.1 Zuständigkeitsabklärung bei der Kantonalen Ethikkommission 4
2.2 Erhebung und Auswertung 4
2.3 Stichprobe 4
2.4 Interviewinhalte 5
3 Ergebnisse 5
3.1 Fragebogen zum Cannabiskonsum 6
3.2 Interviews 6
3.2.1 Was mit dem Cannabiskonsum verbunden wird und Tageszeit des Konsums 6
3.2.2 Aktuelle Situation des Cannabiserwerbs (Schwarzmarkt) 8
3.2.3 Erste Gedanken zum regulierten Cannabiserwerb in Apotheken 9
3.2.4 Verkaufsablauf in Apotheken 9
3.2.5 Cannabisprodukte in Apotheken 10
3.2.6 Vor- und Nachteile des Cannabiserwerbs in Apotheken 12
3.2.7 Musskriterien 13
3.2.8 Unterschiede des Cannabisangebotes in Bezug auf das Alter der Konsumenten 14
3.2.9 Preis 14
3.2.10 Datenschutz 15
3.2.11 Eine andere Perspektive 16
4 Fazit 17
5 Anhang 1: Frageroute 19
6 Anhang 2: Informed Consent 23
7 Anhang 3: Glossar 26

1 Einleitung

Mit der Änderung des Betäubungsmittelgesetzes, die im Mai 2021 in Kraft getreten ist, wird es nun möglich, wissenschaftliche Untersuchungen zum nicht medizinischen Cannabiskonsum durchzuführen.
Eine Studie zum regulierten Erwerb und Konsum von Cannabisprodukten in Berner Apotheken ist für den Herbst 2022 geplant und von der Universität Bern durchgeführt. Das übergeordnete Ziel der Studie ist es zu evaluieren, wie sich der regulierte Cannabiserwerb auf das Konsumverhalten, die Gesundheit und das Sozialleben der bereits Cannabis konsumierenden Personen im Vergleich zu Personen, die ihr Cannabis weiterhin auf dem Schwarzmarkt erwerben, auswirkt.
Um Verkaufsabläufe und Cannabisprodukte möglichst kundengerecht aufzubereiten und einzurichten, Personen interviewt, die bereits regelmässig Cannabis zum Genuss konsumieren.
Das Ziel dieser Interviews wie auch der zukünftigen Gruppendiskussionen ist es, ein kundengerechtes Angebot in den Apotheken für die bevorstehende Studie schaffen. Dier Start der Umsetzungsstudie ist für den Herbst 2022 beplant.

Dieser Auswertungsbericht ist so aufgebaut, dass zuerst das methodische Vorgehen beschrieben wird und anschliessend, entlang der Frageroute die Ergebnisse präsentiert werden. Am Ende folgt ein kurzes Fazit sowie Ideen, welche Themen in den nächsten Gruppendiskussionen aufgenommen werden könnten.

2 Methodisches Vorgehen

Nachfolgend werden die verschiedenen Teile des Vorgehens beschrieben. Alle befragten Personen haben vor dem Interview einen sogenannten Informed consent (siehe Anhang 2) unterzeichnet und sich durch ihre Unterschrift mit dem Interview und dessen Auswertung einverstanden erklärt. Darin wurde ihnen Anonymität zugesichert.

2.1 Zuständigkeitsabklärung bei der Kantonalen Ethikkommission

Am 31. Mai 2021 wurde das Konzept zur qualitativen Begleitforschung mit dem Titel «Einsatz einer partizipativen Begleitgruppe bestehend aus Cannabiskonsumierenden Erwachsenen als Ergänzung während der Planungsphase des Projekts SCRIPT 2» bei der Kantonalen Ethikkommission (KEK) eingereicht, um deren Zuständigkeit zu prüfen. Die KEK befand, dass sie *nicht* zuständig sei. Dies bedeutet, dass diese Forschung nicht unter das Humangesetz Artikel 2, Absatz 1 fällt und Einzel- und Gruppeninterviews mit der ausgewählten Zielgruppe durchgeführt werden können, ohne ein Gesuch einzureichen. Nach diesem Bescheid vom 20. Juni 2021 wurde mit der Akquise von Teilnehmenden gestartet.

Die Akquise von cannabiskonsumierenden Erwachsenen, die an Einzel- und Gruppeninterviews teilnehmen wollen, nahm einige Zeit in Anspruch. Zuerst wurde der Fokus ausschliesslich auf die Teilnahme an Gruppeninterviews gelegt, darauf meldete sich lediglich eine Person. Erst als Einzelinterviews in den Vordergrund gerückt wurden, meldeten sich mehrere Interessierte und die Untersuchung konnte starten.

2.2 Erhebung und Auswertung

Die Daten wurden mittels Einzelinterview via Zoom entlang einer definierten Frageroute im Zeitraum zwischen dem 1. November 2022 und dem 13. Dezember 2022 erhoben. Die Gespräche dauerten im Schnitt 60 Minuten und wurden digital aufgezeichnet sowie anschliessend transkribiert.

Die Transkripte wurden mittels der Software MAXQDA inhaltsanalytische ausgewertet. Das bedeutet in einem ersten Schritt die Codierung der Texte, anschliessend eine Verdichtung der einzelnen Aussagen sowie das Verschriftlichen der Ergebnisse.

2.3 Stichprobe

Die Teilnahme an der SCRTIP-2 Studie wurde auf verschiedenen Wegen ausgeschrieben und aktiv nach cannabiskonsumierenden Personen gesucht. So wurde per Mail wie auch mittels Aushang darauf aufmerksam gemacht. Die Ausschreibung stellte ausschliesslich Gruppeninterviews bzw. die Arbeit in einer aktiven Begleitgruppe in Aussicht. Daraufhin meldete sich lediglich eine Person. Erst mit der Aufnahme von Einzelinterviews an Stelle von Gruppeninterviews konnten genügend Personen akquiriert werden. Alle befragen Personen gaben am Ende des Einzelinterviews an, mit Gruppendiskussionen einverstanden zu sein.

Es wurden insgesamt neun Personen einzeln, via zoom interviewt. Davon leben fünf Personen in der Stadt Bern, vier Personen in der Agglomeration von Bern. Alle befragten Personen gaben an, zurzeit berufstätig bzw. in Ausbildung oder im Studium zu sein. Die Branchen, in denen die Befragten arbeiten sind vielfältig, sie nannten folgende: Studium (3, 2 davon Multimedia Production), Logistik (2), Finanzbranche, Grafik, Fotografie und Fitnessbereich. Die Befragten äusserten zum Zeitpunkt der Befragung allein (3), in einer Partnerschaft (3), in der Ursprungsfamilie (2) und in einer Wohngemeinschaft zu wohnen. Alle befragten Personen gaben an, im Besitz der Schweizer Staatsbürgerschaft zu sein. Untenstehend wird die Stichprobe tabellarisch dargestellt.

Tabelle 1 Übersicht der Stichprobe

<table>
<tr><td>Jahrgang:</td><td colspan="3">1957 bis 2002
1957, 1975, 1990, 1991, 1993, 1999, 2000, 2001, 2000</td></tr>
<tr><td>Geschlecht</td><td>5 männlich</td><td>3 weiblich</td><td>1 non-binär</td></tr>
<tr><td>Berufsbranche:</td><td colspan="2">Studium (3)
Logistik (2)
Finanzen (1)</td><td>Fotografie (1)
Grafik (1)
Fitness (1)</td></tr>
<tr><td>Wohnsituation</td><td colspan="2">Alleinlebend (3)
In Partnerschaft lebend (3)</td><td>In der Ursprungsfamilie lebend (2)
In Wohngemeinschaft lebend (1)</td></tr>
<tr><td>Nationalität</td><td colspan="3">Schweiz (9)</td></tr>
</table>

2.4 Interviewinhalte

Das Interview umfasste fünf Themenblöcke, die nachfolgend kurz beschrieben werden. Als Einstieg wurde die Frage gestellt, was die Befragten mit dem Konsum von Cannabis verbinden bzw. zu welcher Tageszeit sie Cannabis konsumieren. Die aktuelle Situation der Cannabisbeschaffung, also Vor- und Nachteile des Schwarzmarktes bzw. des Eigenanbaus. Ein weiterer Themenblock befasst sich mit dem Verkauf von Cannabis in Apotheken. Dabei wurden der optimale Verkaufsablauf, die gewünschten Cannabisprodukte, Vor- und Nachteile der Cannabiserwerbs in Apotheken und der Datenschutz näher beleuchtet. Anschliessend wurden die Befragten eingeladen, die Perspektive von anderen, ihnen bekannten Cannabiskonsumierenden einzunehmen und deren Meinung einzubringen. Als weiteres wichtiges Thema wurde der Preis pro Gramm Cannabis auf dem Schwarzmarkt und in der Apotheke aufgenommen. Als Abschluss folgten Fragen zu sozio-demographischen Aspekten, um die Stichprobe beschreiben zu können.

3 Ergebnisse

Die Ergebnisse werden nachfolgend entlang der Frageroute aufgeführt. Zahlen in runden Klammern weisen darauf hin, wie viele der Befragten in diese Richtung geantwortet haben. Befindet sich keine Klammer hinter der Aussage, handelt es sich um eine Einzelnennung. In eckigen Klammern werden

die Nummer des Transkriptes (T) sowie der Absatz in der MAXQDA-Datei (A), in welchem das genannte Zitat gefunden werden kann, aufgeführt.
Die Teilnehmenden habe bereits mit dem Informed consent einen kurzen Fragebogen zu ihrem Cannabiskonsum ausgefüllt. Als erstes werden diese Ergebnisse dargestellt. Anschliessend folgen die Ergebnisse aus den geführten Interviews.

3.1 Fragebogen zum Cannabiskonsum

Alle neun Personen haben diesen kurzen Fragebogen zur ihrem aktuellen Cannabiskonsum vor gängig ausgefüllt.
Die Befragten gaben an, Tetrahydrocannabinol (>1%) also illegales Cannabis zu konsumieren (9). Sechs Personen konsumieren laut ihren Angaben auch Tetrahydrocannabinol (<1%) und zwei Personen auch Cannabinol-Produkte (CBD). Die meisten der befragten Personen konsumieren täglich (6), wöchentlich oder wöchentlich – monatlich (2) Cannabis. Alle Befragten gaben an, Cannabisblüten zu rauchen. Vier Personen gaben an, ebenfalls Harz zu konsumieren. Vier Personen gaben an, Cannabisblüten zu vaporisieren, eine Person gab an, e-Flüssigkeit zu dampfen. Auf die Frage, weshalb sie Cannabis konsumieren würden, wurden am meisten «zum Genuss, einfach so» angekreuzt (9), gefolgt von «zur Entspannung» (7), «gegen Nervosität» (5), «gegen Krankheit/ Schmerz» (2) und «andere Gründe».
Es kann sein, dass die Angaben aus dem vorangegangenen Fragebogen nicht mit den Interviewergebnissen übereinstimmen. Das liegt in der Natur der unterschiedlichen Erhebungsarten und auch daran, dass in den Interviews, nicht so explizit nach den Konsumgewohnheiten gefragt wurde, wie im Fragebogen.

3.2 Interviews

Nachfolgend werden die Ergebnisse aus den geführten Interviews dargestellt.

3.2.1 Was mit dem Cannabiskonsum verbunden wird und Tageszeit des Konsums

Auf die Frage, was die Cannabiskonsumierende mit dem Konsum von Cannabis verbinden, äusserten sie sich unterschiedlich. Doch eines scheint für alle gleich zu sein, so gaben alle Befragten an, dass Cannabis für sie Entspannung (9) bedeute. Dies verdeutlicht untenstehendes Zitat sehr gut.

> «Generell mit Entspannung, Runterfahren, ebenso Entspannung. Ja, Kreativität. Das ist etwas therapeutisch-medizinisches zur Entspannung und auch Lockerheit. Ja, das sind so gute Sachen.» [T5-A3]

Des Weiteren wurden Genuss (6), Lebensgefühl (5), Ritual (5), Leidenschaft (3), Gelassenheit (2), Zusammengehörigkeit, Sinnlichkeit, intensive Wahrnehmung (z.B. von Musik) und Spontaneität als Assoziationen zum eigenen Cannabiskonsum genannt. Aus dieser Vielfalt, was Cannabis für die Befragten bedeutet, nachfolgend zwei Zitate, die diese Aussagen untermalen. Das erste Zitat bezieht sich auf das Ritual und die zweite Aussage auf die Leidenschaft, die mit dieser Pflanze einher geht.

> «Es ist so, dass ich am Morgen aufstehe, einen Kaffee trinke und mir mal einen Joint zurecht mache und den nachher mit einem Kaffee zusammen auf dem Balkon geniesse. Es ist also eigentlich schon am Morgen. Und wenn ich arbeite, dann vor der Arbeit natürlich. Ich stehe am Morgen auf, nachher rauche ich eines und gehe nachher arbeiten, gemütlich.» [T8-A9]
>
> «Es ist eine vielseitige Pflanze und ich bin fasziniert von dieser Pflanze.» [T8-A13]

Nebst diesen Genussaspekten wurde auch von drei Personen die Medikation mit Cannabis erwähnt, dies vor allem in Zusammenhang mit Schmerzen (z.B. Menstruations-, Rückenschmerzen) oder zur Behandlung einer Hyperaktivität.

Die meisten der Befragten konsumieren Cannabis abends (7). Das nachfolgende Zitat steht beispielhaft dafür.

> «Das ist eigentlich immer gegen Abend, so ungefähr 2-3 Stunden bevor ich schlafen gehe. Das hat damit zu tun, dass ich den Aftereffekt ausschlafen kann und ich am nächsten Tag eigentlich nichts mehr spüre.» [T6-A7]

Zwei Personen gaben an, Cannabis auch vormittags *vor*, jedoch nicht *während* der Arbeit zu konsumieren. Drei Personen äusserten, weder vor noch während der Arbeit Cannabis zu konsumieren, zum einen, weil die Konzentration weniger ausgeprägt sei (2) und zum anderen, weil Cannabis und dessen Konsum von der Gesellschaft immer noch stigmatisiert werde (2), und bei der Arbeit deshalb niemand wissen soll, dass in der Freizeit Cannabis konsumiert werde.

> «Nein, ich konsumiere nicht während der Arbeit. Aber auf der Arbeit, weiss auch sonst niemand, dass ich in der Freizeit kiffe. Weil, es ist gleichwohl Stigma behaftet, das es dann ein bisschen als Sündenbock hinhalten muss, vielleicht wenn man krank ist oder mal etwas vergisst oder sonst unkonzentriert ist. Dann heisst es sehr schnell: "Aha, der kifft halt."» [T5-A53]

In Bezug auf die Wirkung von Cannabis während der Arbeit sagten je eine Person, ihre Konzentration bzw. die Fokussierung sei besser nach dem Cannabiskonsum. Eine Person meinte, sie würde besser arbeiten nach dem Cannabiskonsum, weil sie weniger nervös sei.

Alle Befragten gaben an, aktuell vorwiegend Cannabisblüten zu rauchen. Je zwei Personen betonten, dass sie Indoor-, bzw. Outdoorhanf bevorzugen. Harz werde ebenfalls konsumiert (4), teilweise gemischt mit Blüten statt mit Tabak. Drei Personen äusserten regelmässig CBD-Hanf zu konsumieren (rauchen (3), in Beautyprodukten (1).

3.2.2 Aktuelle Situation des Cannabiserwerbs (Schwarzmarkt)

Als Bezugsquellen wurden vorwiegend Bekannte genannt (7), dies mit der Begründung, dass da die Qualität sicher sei. Falls die Bekannten nichts mehr zu verkaufen hätten, würden sie bei Bekannten von Bekannten Cannabis kaufen. Eine Person merkte an, dies sei unangenehm, sie käme sich in dieser Situation als Bittsteller vor und das würde bei ihr Stress auslösen. Mehrere Personen äusserten, im Ausgang, wenn bei anderen mitgeraucht werde, könne es sein, dass die Quelle des Grases nicht bekannt sei, das sei dann eher etwas Spontanes (3). Versiegt eine Cannabisquelle, z.B. weil die Indooranlage von der Polizei entdeckt wurde oder durch einen Wohnortwechsel der cannabiskonsumierenden Person, gehe die Suche nach einem neuen, vertrauenswürdigen Dealer los. Dies sei anstrengend und zeitaufwendig (3). Die Mehrheit der befragten Personen äusserte die Überzeugung, dass der Schwarzmarkt keine Vorteile habe (6). Von den anderen wurden folgende Vorteile ins Feld geführt: es gebe exotischere Sorten auf dem Markt (mit dem Preis, dass da die Herkunft nicht bekannt sei); die gelebte Gemeinschaft unter Cannabiskonsumierenden wurde positiv hervorgehoben; die Anonymität, wenn das gewünscht sei, sei gegeben; auf dem Schwarzmarkt werde keine Cannabissteuer erhoben, wie das in der regulierten Abgabe zu erwarten sei (Analog der Alkohol- oder Tabaksteuer). Alle befragten Personen meinten, dass die Kriminalisierung des Cannabiskonsums einer der anstrengendsten Aspekte am Schwarzmarkt sei. Was nachfolgendes Zitat prägnant aufzeigt:

> «Es ist immer so, dass dich jeden Moment die Polizei erwischen kann. Und dann gibt es eine Busse und das Gras ist weg. Ja, das ist halt einfach traurig. Ich finde es traurig, dass eine Pflanze illegal sein kann. Das ist ja wirklich eine Pflanze. Dass etwas Chemisches illegal ist, das finde ich in Ordnung, absolut. Kein Problem. Aber die Pflanze selbst, ...// Weil böse sind sie nicht.» [T8-A37]

Es wurden folgende weitere Nachteile genannt: eine gesundheitliche Gefahr von gestrecktem Cannabis sei vorhanden (3); man müsse nehmen, was auf dem Markt sei (2); die gekaufte Menge sei meist geringer als vereinbart (z.B. 4.7 Gramm statt 5 Gramm); reines Cannabis sei auf dem Schwarzmarkt kaum zu bekommen; die Qualität sei unterschiedlich; das Rumhängen mit Kriminellen und die Unzuverlässigkeit mancher Dealer (als Käufer sitze man am kürzeren Hebel), sei anstrengend.

Mehrere Personen gaben an, ihr Cannabis selbst anzubauen (4), eine dieser Personen schilderte eine sehr reiche Ernte gehabt zu haben und dies als Nachteil zu empfinden, da die Ernte den Eigenbedarf deutlich übersteige. Auch Eigenanbau ist illegal und es bestehe immer ein Risiko erwischt zu werden (3). Vorteile des Eigenanbaus werden in der Qualität des Endproduktes gesehen; auch sei eigenes Gras nicht so stark wie gekauftes. Eine Person merkte an, dass ein oder zwei Cannabispflanzen im Garten zu ziehen ein Hobby sei und auch Spass mache.

Eine Stimme sprach den Jugendschutz auf dem Schwarzmarkt an, denn bei der regulierten Abgabe, dürften lediglich Personen ab einem Alter von 18 Jahren Cannabis in der Apotheke erwerben, die jüngeren würden sich weiterhin auf dem Schwarzmarkt eindecken. Eine andere Person gab zu bedenken, dass die regulierte Abgabe dem Schwarzmarkt ein Dorn im Auge sein könnte.

3.2.3 Erste Gedanken zum regulierten Cannabiserwerb in Apotheken

Die Befragten wurden gebeten, sich gedanklich in die Situation der regulierten Cannabisabgabe in Apotheken zu versetzen und zu schildern, was ihnen als erstes durch den Kopf gehe. Mehrere Personen verglichen das bevorstehende Setting mit Cannabisverkaufsstellen im Ausland (Coffeeshops in Amsterdam, Social Clubs in Spanien) (3), mit einem Tee- und Kräuterladen (2) und auch mit einer Netflixserie in der es um eine Hanfapotheke geht (Disjointed). Einer Person kam als erstes ein separates Regal für CBD- wie auch THC-haltige Cannabisprodukte in der Apotheke in den Sinn. Eine andere Person äusserte die Gedanken, es gäbe ein Cannabissortiment mit verschiedenen Sorten, in sicherer Qualität. Die Cannabisprodukte wären beschriftet und die Produkteigenschaften beschrieben (2). Folgendes Zitat lässt die ersten Gedanken bildlich werden:

> «Ich sehe vor mir einen Tresen, dahinter steht einer im Laborkittel, weil das Ganze muss recht steril bleiben. Und ja, dass man so verschiedene Vorratsgläser hat, wo man daran riechen kann. Und man es [Ergänzung: das Cannabis] anfassen und sehen kann, wie es aussieht. Und hinten sind diese verschiedenen Gläser. Dass man noch Zubehör kaufen kann, Papierchen, Filter. Dass es eventuell noch ein Fumoir geben würde, damit man es [Ergänzung: das Cannabis] gerade probieren kann, das wäre natürlich auch nicht schlecht. Vielleicht noch gemütliche Sofas, dass man zusammensitzen, zusammen reden kann. Weil, Kiffer reden gerne miteinander.» [T8-A43]

Einer weiteren befragten Person gingen als erstes die Vorteile des Cannabisangebots in Apotheken durch den Kopf. So beschrieb sie etwa, keinen Beschaffungsstress mehr zu haben und Cannabis in guter, gleichbleibender Qualität, in der vereinbarten Menge zu erhalten. Drei Personen äusserten, ihre ersten Gedanken seien Freude über diesen politischen und gesellschaftlichen Schritt. Dies untermalt nachstehendes Zitat:

> «Einen guten Schritt des Parlaments und der Gesellschaft, würde ich behaupten. Ich finde das super, ist das so. Das hilft auch vielen Leute, denke ich. Und wenn ich nachher konkret daran denken, wie ich in den Laden reingehe, dann würde es mich sicher interessieren, wie es im Laden präsentiert wird. Wie es vielleicht riecht sogar. Wie die Mitarbeitenden darauf zu sprechen sind. Ob sie hilfsbereit sind, ob sie vielleicht beraten können. Ich würde sehr interessiert – also, wenn es einen Stand hat - an diesem Cannabisstand vorbei gehen und dort reinschauen. Und vielleicht auch eine Broschüre durchlesen. Das würde mich sehr interessieren. [T3-A39]

Eine Person äusserte, dieser Gedanke fühle sich speziell an. Sie denke an Menschen, die ihr Methadon abholen, um von ihrer Sucht loszukommen.

3.2.4 Verkaufsablauf in Apotheken

Die meisten der Befragten äusserten, sich das Verkaufsgespräch im öffentlichen Verkaufsraum der Apotheke vorzustellen (7) und sie ihren Produktewunsch offen aussprechen würden. Zwei Personen meldeten gewisse Vorbehalte an, Cannabisprodukte im öffentlichen Bereich einer Apotheke zu kaufen.

So meinte die eine Person, gerade zu Beginn des regulierten Cannabiserwerbs, wäre es hilfreich einen Nebenraum zu haben, um ein Cannabisverkaufsgespräch zu führen und dies so lange bis, Cannabis in der Gesellschaft als «normal» angesehen werde. Und die zweite Person meinte, es sollte grundsätzlich die Möglichkeit bestehen, ein Verkaufsgespräch zu persönlichen Themen im Nebenraum führen zu können. Zwei andere Personen gaben zu bedenken, dass der Cannabisverkauf im öffentlichen Bereich der Apotheke, zu einer gesellschaftlichen Akzeptanz beitrage und deshalb auf Nebenschauplätze zu verzichten sei.

In den geführten Interviews wurde deutlich, dass sich Cannabiskonsumierende in der Apotheke ein kompetentes Beratungsgespräch wünschten (6). Die Angestellten der Apotheke sollen analog zu Medikamenten auch in Bezug auf Cannabis in der Lage sein, die Kundschaft entsprechend ihren Bedürfnissen zu beraten (6). So sollten sie fachkundige Auskünfte (Wirkung, Eigenschaften, …) zu verschiedenen Cannabissorten und -produkten geben können (4). Gewünscht wäre des Weiteren eine Sortimentsübersicht entweder mündlich (3) oder schriftlich in Form einer Produkteliste analog der Karte der Coffeeshops in Amsterdam (2), wie das folgende Zitat deutlich macht:

> «Vielleicht gäbe es auch eine Liste. […] Da sind so diese Sorten, die Preise. So eine Art Karte, das wäre noch gut. Da kann man schnell durchlesen, es hat von dem, es hat von dem. Oder, dass die Verkaufsperson fragen könnte: "Wofür brauchen Sie es? Ist es eher, dass Sie am Wochenende high sein möchten, oder eher für...//" Ja, einfach wirklich eine kompetente Beratung wie ich für jedes andere Arznei oder Genussmittel auch bekommen würde.» [T4-A37]

Des Weiteren betonte eine Stimme, sie möchte über Cannabis-Neuigkeiten informiert werden.

Im Verkaufsraum sei es wichtig, dass die Produkte zu sehen (2), jedoch ausschliesslich für Angestellte der Apotheke zugänglich seien. Dies könnte in Form einer Vitrine oder eines Regals realisiert werden. Die Sichtbarkeit von Cannabisprodukten würde ebenfalls zur gesellschaftlichen Anerkennung von Cannabis und zur Normalität beitragen, gab sich eine Stimme überzeugt.

3.2.5 Cannabisprodukte in Apotheken

Die Befragten äusserten klar, dass sie Blüten (9) sowie Haschisch (8) in der Apotheke erwarten. Dabei werden Blüten als Standardprodukt (9) und Haschisch als etwas besonders (5) gesehen.

Die befragten Personen gaben an, ein Blütenangebot aus Outdoor- und Indoorproduktion zu erwarten, den einen sei Outdoor in Bioqualität (3) sehr wichtig und anderen die Indoorqualität (3) und wieder andere finden beide Varianten fein (3). Des Weiteren wurde die Auswahl aus verschiedenen Cannabissorten (Sativa, Indica, Ruderalis) (4) sowie aus unterschiedlichen CBD- und THC-Gehalten (4) als Erwartung geäussert.

In Bezug auf Haschisch wurde ebenfalls eine Vielfalt gewünscht, es wurden Namen wie: Charas, schwarzer Afghan, roter Libanon und grüner Maroc wie auch ein Mischprodukte (Moonrock) erwähnt.

> «Unbedingt eben Harz. Also die festen. Afghan, Libanon, Maroc, alle diese feinen Sachen. Weil die kann man nicht selbst herstellen. Also, die kann ich nur kaufen.» [T9-A49]

Weitere Cannabisprodukte, die in einer Apotheke zukünftig gern gesehen wären: Gummibärchen (5), Crèmen (Lippen-, Körpercrèmen) (4), Öl (zum Einreiben und zum Einnehmen) (3), Sirup (2), Cookies (2), Tee, Hustensirup, Halspastillen, Kaugummi und Liquids für Vaporizer. Tabletten lehnen die Befragten ab, da fehle der Genuss (Geschmack, Duft) und das Ritual (Bauen, Rollen). Eine Person gab zu bedenken, Cannabisgummibärchen würden sie an Alkopop erinnern, und es sei unfair gegenüber Jugendlichen, solche auf den Markt zu bringen. Zwei Personen fanden, dass Edibles klar gekennzeichnet sein müssten, insbesondere Süssigkeiten wie Gummibärchen, damit für alle klar und sichtbar sei, dass Cannabis drin sei und verzögert wirke.

Produkte, die in Zusammenhang mit dem Cannabiskonsum stehen wie bspw. Filter, Papierchen, Feuerzeug, Vaporizer, Pens und vieles mehr, würden in einer Apotheke nicht zwingend erwartet (3), dafür gäbe es die Growshops bzw. die Fourtwenty-Shops (2).

> «Weisst du, es gibt ja diesen Fourtwenty-Laden. Es gibt doch ein paar, so Growshops oder so. Da bekommst du all diese Kiffer-Accessesoirs. Ich denke nicht unbedingt, dass es das alles in der Apotheke geben müsste. Aber wirklich die Breite der Cannabisprodukte, also der Stoff quasi schon. Dort wünsche ich mir eine Vielfalt von allem was es gibt.» [T1-A47]

Eine Person strich heraus, dass gerade Filter in Apotheken, die Cannabis verkaufen, angeboten, ja sogar im Verkaufsgespräch aufgenommen werden sollen. Denn es geistere bei vielen Cannabiskonsumierenden die These herum, dass Filter, das THC und somit das Rauscherlebnis minimiere. Die Filter sollten als Prävention und Aufklärung (Gesundheit, Wirkung) mitverkauft werden.

> «Was ich immer allen predigen und was ich finde, das sollte die Apothekerin auch verkaufen können. Das wären Filter. Dass sie vor allem den jungen Menschen sagt: Ihr wisst, dass THC, mit dem ihr euch berauschen wollt, durch jeden Filter hindurch geht. Nur das, was für die Lunge nicht gut ist, im Filter hängen bleibt. Darf ich Ihnen noch ein Päckli Filter dazugeben? Das finde ich extrem wichtig. Ich sehe immer wieder Jungs, die ihren Mädchen etwas anbieten und alle sind am Husten wie wild. Und wenn man weiss, dass es immer noch *in* ist, nur so ein Papierfilter zu machen und keinen richtigen Filter zu gebrauchen. Und wenn ich den Jungen das sage, dann sagen sie: Ja, aber das ist so teuer. Sie haben einfach zu wenig von diesem Cannabis und meinen es bleibe THC im Filter hängen und das ist nicht wahr. Das ist eine Aufklärung, und das finde ich eine wichtige Information. Die Berauschung ist genau gleich, auch mit einem guten Filter. Und, wenn man sieht, was da alles im Filter drin ist, dann ist ja klar, wo das sonst ist, wenn man ohne Filter raucht. Also, das ist wirklich eine ganz wichtige Information, die man allen Jungen sagen muss. [T9-A47]

Ganz besonderen Wert legen die Befragten auf die Transparenz sei dies nun in Bezug auf Inhaltsstoffe, Sorte, Wirkung und die THC- und CBD-Gehalte (6) sowie auf die Art der Wachstums- und Düngebedingungen (z.B. Indoor, Outdoor) (6). Diese Informationen sollten gut sichtbar auf der Verpackung zu lesen sein. Des Weiteren wird Wert auf das Aussehen der Blüten und auf den Geruch gelegt, dies bedeutet, dass die Blüten gut sichtbar (5) und riechbar (4) (z. B in Form eines Testers) vorgelegt würden. Dieser Aspekt wird mit nachfolgendem Zitat unterstrichen:

> «Ich fände dies schon noch positiv. Wie bei den Schminksachen, wo es immer ein Tester gibt, dass es auch da ein Tester gäbe, dass man nicht die Katze im Sack kaufen müsste. Es gibt Leute, die viel mehr auf Zahlen, Daten, Fakten sind und die einfach alle Angaben genau haben wollen. Und es gibt Leute, die mehr auf alles andere schauen. Also mehr auf die Sinne achten. Und ich finde, es sollte beide ansprechen.» [T1-A74]

Auf ein Beratungs- oder Verkaufsgespräch legen einige der befragten Personen (4) Wert, um ein Produkt kaufen zu können, das möglichst ihren Vorstellungen entspreche. Zwei Stimmen äusserten, ihnen sei eine Vielfalt von Produkten und Hanfsorten wichtig. Zwei Personen äusserten, sie würden lediglich Outdoor-Hanf kaufen und zwei weitere Personen dagegen ausschliesslich Indoor-Hanf. Eine Person meinte, es sei ihr egal, ob Outdoor- oder Indoor-Hanf, Hauptsache, der THC-Gehalt sei nicht zu hoch. Dagegen meinten zwei Personen, ihnen sei ein hoher THC-Gehalt sehr wichtig. Des Weiteren wurden noch Aspekte wie Regionalität und Preis-Leistung als wichtig genannt.

Die befragten Personen äusserten sich dazu, welche der oben erwähnten, erwarteten Produkte, sie selbst kaufen würden. Blüten nannten acht Personen, wobei eine Person spezifizierte, lediglich Indoor-Cannabis zu kaufen. Eine weitere Person gab an, gar keine Blüten zu kaufen, da sie weiterhin selbst anbauen würde. Sie hätte jedoch an Haschisch Interesse, wie auch weitere Befragte (6). Vier Personen meinten, sie würden sich durch das Sortiment durchprobieren. Einzelnennungen waren: CBD- bzw. THC-Öl (auch zum Einnehmen), Cookies und Halswehbonbons.

Auf die Frage, was sie von drei Produkten mit unterschiedlichen CBD- und THC-Gehalten (CBD hoch THC tief/ CBD und THC mittel/ CBD tief, THC hoch) halten würden, meinten fünf Personen, das sei ein guter Anfang. Eine dieser Personen äusserte weiter, an CBD gar kein Interesse zu haben, da es ihr davon übel werde und sich dann wohl für die dritte Variante (CBD tief, THC hoch) entscheiden würde. Eine weitere Stimme ergänzte, dass die Pflanze Cannabis deutlich mehr zu bieten habe als diese drei Varianten.

Die Verpackung von Cannabisprodukten wurde von einigen Befragten thematisiert. So gaben ein paar an, die Verpackung sei gar nicht wichtig (3). Andere fanden jedoch, die Verpackung dürfte neutral, schlicht und doch edel gehalten sein (2), zum Beispiel in einer Kartonschachtel mit Sichtfenster (2), in einem Glas (2) oder sonst einem Döschen.

3.2.6 Vor- und Nachteile des Cannabiserwerbs in Apotheken

Im regulierten Erwerb von Cannabis in Apotheken werden viele ***Vorteile*** gesehen. So wurde die sichere Qualität (9), der einfachere Bezug im Sinne einer Alltagsbesorgung (5), die Auswahl von Cannabisprodukten (4) und die kompetente Beratung (3) positiv hervorgehoben. Des Weiteren wurden die Transparenz der Inhaltsstoffe (THC-, CBD-Gehalt) (2) und die persönliche Sicherheit während des Kaufprozesses genannt (Apotheke versus Unterführung). Nachfolgendes Zitat einer befragten Person zeigt auf, wie der Cannabiskauf zu den Alltagsbesorgungen werden könnte:

> «Dass es wie ein Teil der Alltagsbesorgungen werden darf. Ich gehe die Münstergasse rauf über den Markt, kaufe Kartoffeln, Gemüse und Milch. Ich kann auf dem Rückweg - ich habe

> immer das Gefühl, es müsste bei mir in der Rathausapotheke sein...// Ja, einfach etwas Normales. So wie ich mein Päckchen Panadol hole oder so...// Ich sehe überhaupt nichts, was daran anstössig sein soll, dass es das in der Apotheke gibt. Ich denke, das ist schon der Ort, wo es hingehört.» [T4-A27]

Auf gesellschaftlicher Ebene äusserten die Befragten folgende Vorteile zu sehen: Cannabis würde in der Gesellschaft anerkannt (5); die illegalen Strukturen würden aufgebrochen und damit der Schwarzmarkt geschwächt (5); regionale Bauern würden berücksichtigt und das generiere Arbeitsplätze sowie Polizeiressourcen würden frei. Zwei Personen gaben an, ausschliesslich Vorteile im Cannabisverkauf in Apotheken zu sehen.

> «Ich persönliche sehe jetzt keinen Nachteil. Nein, eben, ich habe das Gefühl, es ist besser verfügbar, es ist näher vorhanden. Man muss sich nicht eine Woche im Voraus vorbereiten, wenn man geschwind etwas kaufen will. Ich sehe eigentlich nur Vorteile.» [T5-A79]

Nachteile werden in den vermutet höheren Preisen gesehen (5). Eine dieser Personen gab an, dass es gerade für junge Menschen oder Menschen mit einem beschränkten Budget, schwierig werde in der Apotheke einen höheren Preis zu bezahlen und diese Menschen zurück in den Schwarzmarkt gedrängt würden. Diese Bedenken zeigt das nachstehende Zitat:

> «Ein Junger, der mit seinem Lehrlingslohn kämpft, für den sind zwei Franken mehr, halt zwei Franken mehr.» [T9-A94]

Eine andere Stimme ging davon aus, dass die Preise höher als auf dem Schwarzmarkt sein müssen, da das Verkaufspersonal sowie Qualitätsprüfung und eine allfällige Steuer auf die Produkte abgewälzt würden. Da es sich in den Apotheken um eine regulierte Abgabe handle, sei eine gewisse Kontrolle (des Cannabiskonsums) gegeben und dies könnte auch störend sein (3). Eine weitere Person meinte, der Cannabiskonsum könne zum Mainstream werden, weil Cannabis auf diese Weise für alle zugänglich sei.

3.2.7 Musskriterien

Auf die Frage, welche Muss-Kriterien erfüllt sein müssten, damit die Befragten ihr Cannabis zukünftig in der Apotheke kaufen würden, wurden folgend Kriterien geäussert: einwandfreie Qualität der Produkte (8); der Preis müsse stimmen (5); eine Auswahl an Cannabisprodukten (dies mache den Unterschied zum Schwarzmarkt) (4); eine kompetente Beratung (3) sowie Fachwissen bzgl. der optimalen Lagerung von Cannabis. Es folgten Einzelnennungen. So machte eine Stimme deutlich, dass sie Bioqualität erwarte. Eine andere Person verlangte Transparenz bzgl. Inhaltsstoffen und Düngung. Und eine dritte Person äusserte, der Cannabis sollte (auch) in Apotheken wie ein Alltagprodukt behandelt werden (keine Stigmatisierung). Eine weitere Stimme meinte, sie möchte das Produkte ansehen und riechen dürfen, bevor sie es kaufe.

3.2.8 Unterschiede des Cannabisangebotes in Bezug auf das Alter der Konsumenten

Während dem Interview wurde gefragt, worin der Unterschied von jüngeren und älteren Cannabiskonsumierenden Personen liege, was heterogenen Sichtweisen aufzeigte. So meinten zwei Personen, ältere Menschen würden lieber Cannabis mit einem geringeren THC-Gehalt konsumieren, jüngere eher einen hohen THC-Gehalt bevorzugen. Eine Person meinte, ältere Personen würden eher Haschisch, jüngere eher Blüten bevorzugen. Ältere würden Qualität vor Quantität sehen und eher Diskretion bevorzugen. Eine Person äusserte, das Alter spiele keine Rolle, kiffen würde Menschen verbinden, wie nachstehendes Zitat hervorhebt:

> «Ich glaube, das ist wieder das Gefühl von Gemeinschaft. Weil, es ist eigentlich egal, ob du einen 18 jährigen vor dir hast oder einen 80 jährigen. Wenn du rausfindest, der kifft, dann verbindet dich schon etwas mit dieser Person.» [T1-A128]

3.2.9 Preis

Schwarzmarkt

Die Einheiten wie auf dem Schwarzmarkt eingekauft wird, wurden unterschiedlich angegeben. So kaufen die einen ihr Cannabis in Litergefässen, andere in Konfitürengläser, wieder andere in Franken- oder nach Grammangaben. Die wohl häufigste Verkaufseinheit scheint die Frankenangabe zu sein. So gaben einige der befragten Personen (5) an, 20er, 50er oder 100er-Päckchen zu kaufen wobei sich die Zahlen – 20, 50, 100 – auf den zu bezahlenden Betrag beziehe. Die meisten gaben an, pro Einkauf 50 Franken auszugeben (4). Drei Personen äusserten, gerne grössere Mengen auf einmal zu beziehen, damit es für eine Weile reiche und der Preis etwas tiefer liege (Mengenrabatt). Zwei der Befragten meinten, sie würden ihr Gras selbst anbauen und auf dem Schwarzmarkt lediglich Haschisch zu kaufen bzw. manchmal während dem Ausgang Gras auf dem Schwarzmarkt erwerben. Die Preise auf dem Schwarzmarkt für Cannabisblüten wurden zwar unterschiedlich angegeben, sind jedoch in einem Range zwischen fünf und zehn Franken pro Gramm zu verorden. Als Beispiel nachfolgende Aussage einer befragten Person:

> «Also, soviel ich weiss, bezahlt mein Freund sechs Franken pro Gramm. Aber das ist extrem billig, das ist ein Freundschaftspreis. Ich glaube im Normalfall bezahlt man pro Gramm zwischen acht bis zehn Franken. Aber zehn Franken ist dann auch wieder hennenteuer.» [T7-A138]

Zwei Personen machten Preisunterschiede zwischen Indoor- und Outdoorblüten. So würden sie für Outdoorblüten zwischen fünf und sieben Franken pro Gramm bezahlen und für Indoor-Blüten zwischen acht und zwölf Franken pro Gramm, dies weil Indoor-Blüten in der Aufzucht mehr kosten würden (Raummiete, Strom, Dünger, höheres Risiko erwischt zu werden). Haschisch sei auf Schwarzmarkt etwas teurer als Cannabisblüten. So gaben die Befragten an, für ein Gramm Haschisch auf dem Schwarzmarkt zwischen zehn und zwanzig Franken zu bezahlen, das komme auf die Sorte und die Exklusivität des Produktes an.

Apotheke

Was Cannabisblüten in der Apotheke kosten dürften, wurde sehr unterschiedlich beantwortet. So meinten die einen, Cannabisblüten sollten in der Apotheke gleich viel oder nur gering mehr kosten wie auf dem Schwarzmarkt (3), eine Stimme präzisierte, maximal fünf Prozent dürfte der Preis in der Apotheke höher sein. Die Begründung war, dass es unter Cannabiskonsumierenden auch Menschen gebe, für die auch ein marginaler Mehrpreis nicht zu bezahlen sei und diese Menschen somit weiterhin auf dem Schwarzmarkt einkaufen würden. Ebenfalls drei Personen meinten, zehn Franken pro Gramm Cannabisblüten wäre ein fairer Preis. Einige Personen differenzierten die Preise. Für Outdoorcannabisblüten fänden diese Stimmen einen Preis von sieben bis maximal acht Franken pro Gramm stimmig (3). Sie würden für Indoor-Cannabisblüten bis zwölf Franken, für ganz besondere Cannabissorten sogar bis 15 oder 20 Franken bezahlen. Die Qualität müsste jedoch im Exklusivbereich liegen (2). Eine Person äusserte, sie würde in der Apotheke für Cannabisblüten bis zwanzig Franken pro Gramm bezahlen, dies mit der Begründung, dass in den Apotheken eine Auswahl vorliege, die Qualität stimme und die Produktions- Lagerungs- und Lohnkosten auch berücksichtig sein müssten. Dieselbe Person präzisierte, dass hochpreisiges Cannabis auch in der Qualität, Herkunft und Wuchs exklusiv sein müsse und sich dadurch deutlich von Standardprodukten, wie sie im Interview vorgestellt wurden, abheben müssten. Zwei Personen gaben zu bedenken, dass Cannabis in Apotheken zum Preis von zehn bzw. zwölf Franken pro Gramm abschreckend wirken würden.

Haschisch dürfte in der Apotheke je nach Sorte, Herkunft und Qualität zwischen zehn und zwanzig Franken pro Gramm kosten. Geschwärmt wurde unter anderem von schwarzem Afghan, roten Libanon und grünen Maroc. Eine Stimme vergleicht die Situation mit Wein, wie nachfolgendes Zitat zeigt:

> «Wenn ich jetzt beste Qualität schwarzer Afghan bekäme und das ich sonst nirgends bekomme. Wenn ich jetzt weiss, ich kann das regelmässig in der Apotheke holen, dann würde ich auf den Preis schauen. Aber, wenn ich das einmalig kann und ich jetzt 200 für diese 10 Gramm bezahlen müsste, dann würde ich das auch tun. Wenn es der beste ever ist, dann bezahlen Sie 50 oder auch 100 Franken. Wenn es der beste ever ist, dann bezahlen Sie halt auch einmal 100 für eine Flasche (Wein) oder so. Obwohl, das natürlich wahnsinnig viel Geld ist für eine Flasche Wein, aber wenn Sie wissen, da können Sie ihren Liebsten eine Riesenfreude machen, und es ist ein super, super Wein, ja, dann halt.» [T9-A170]

Der Onlinehandel wurde von einer Person erwähnt. Sie gab an, manchmal kalifornische Cannabisblüten zu kaufen und dafür einen wesentlich teureren Preis (~14 Franken pro Gramm) zu bezahlen, jedoch auch eine exklusive Qualität zu erhalten.

3.2.10 Datenschutz

In Bezug auf den Datenschutz sind sich die Befragten einig darüber, dass die Daten nicht an dritte (Polizei, Strassenverkehrsamt, Arbeitgebende etc.) weitergegeben werden dürfen und dies sichergestellt werden muss (9). Eine Person äusserte verschmitzt, dass für alle, die ein Smartphone

auf sich tragen, der Datenschutz eh nicht gewährleistet sei. Mehrere Personen äusserten, ein separater Raum für Beratung und Verkauf sollte zur Verfügung stehen (4), so dass sich Cannabiskonsumierende, die dies wünschen, schützen könnten und ihren persönlichen Angaben (Name, Adresse) nicht im Verkaufsraum nennen müssten. Als weitere Schutzmassnahmen nannte eine Stimme, die schlichte Verpackung der Cannabisprodukte.

Ausweisen möchten sich die Befragten mit einer Karte in Kreditkartengrösse (4) oder mit einer App (3), mit einem Chip am Schlüsselbund. Lediglich eine Person äusserte am liebsten eine entsprechende App zu nutzen, da sie das Smartphone eh immer dabei habe. Mehrere Personen gaben an, kein Smartphone zu besitzen (3), weshalb lediglich eine Karte in Frage käme. Eine Person merkte an, dass die Karte möglichst neutral gestaltet sein sollte. Und eine weitere Person gab zu bedenken, dass eine Ausweiskontrolle junge Leute abhalten könnte, ihr Cannabis in der Apotheke zu kaufen. Des Weiteren regte eine Person an, die Studienkarte jeweils mit einem offiziellen Ausweis (z.B. Identitätskarte) zu kontrollieren. Eine weitere Person meinte, die Studienanmeldung sollte nicht über eine online-Plattform erfolgen, sondern analog auf Papier, dies fände sie sicherer.

Im Zusammenhang mit dem Datenschutz nahm eine Person noch das Thema Polizeikontrolle auf. Sie regte an, auf dem Studien- bzw. Cannabisausweis eine eindeutige ID-Nummer zu vermerken, mit der die Polizei bei den Studienleitenden die Echtheit prüfen könne.

3.2.11 Eine andere Perspektive

Die Befragten wurden gebeten, die Perspektive einer ihnen bekannten Cannabiskonsumierenden Person einzunehmen und zu äussern, was dieser Person in Bezug auf den Erwerb von Cannabis in Apotheken besonders wichtig sein könnte. Datenschutz und Diskretion (4) sowie die Gelegenheit, den Kauf in einem separaten Raum (3) abzuwickeln, wurden genannt. Diese Aussagen werden mit nachfolgenden Zitaten untermalt:

> «Ich denke als erstes einmal an meine Partnerin. Sie konsumiert auch Cannabis. […] Also, sie ist in einem religiösen Haus aufgewachsen. Und Cannabis ist in der Religion selbst nicht etwas, wo man gerne darüber spricht. Es ist in dem Sinn verboten. Und, vielleicht im Laden selbst hätte sie kein Problem, das (Cannabis) zu erwerben, aber wenn die Familie dies rausfinden würde, dann kann ich mir vorstellen, dass die Familie das nicht…» [T6-A157]
> «Und wenn das dann rauskäme. Das kann ich mir vorstellen, das würde wohl nicht so gut rauskommen. [T6-A159]

Des Weiteren nannten die Befragten den Preis (2), die einfache und regelmässige Zugänglichkeit (Apotheken haben Ladenöffnungszeiten) (2), die Beratung und Aufklärung durch Fachpersonen, ein regelmässiges, differenziertes Angebot sowie der Übergang in ein Alltagsprodukt als wichtige Aspekte aus Sicht ihrer Bekannten. Ebenfalls sei es wichtig, nebst Cannabisblüten auch Haschischprodukte anzubieten, äusserte eine weitere Stimme.

4 Fazit

Die befragten Personen begrüssen den regulierten Verkauf von Cannabis in Apotheken. Sie erwarten Cannabisblüten sowie Haschisch und allenfalls noch Edibles kaufen zu können. Ganz besonderen Wert legen sie auf die Qualität und den Reinheitsgrad der Cannabisprodukte, manche schwärmen von Produkten in Bioqualität. Eine vorgesehene Auswahl in Bezug auf die THC- und CBD-Gehalte (THC-hoch – CBD tief/ THC und CBD mittel/ THC tief und CBD hoch) taxierten sie als guten Anfang in die richtige Richtung. Als grosse Vorteile werden eine sichere Cannabisqualität sowie die Anerkennung von Cannabis in der Gesellschaft gesehen. Ein allfälliger Nachteil wird in den höheren Preisen vermutet. Der aktuelle Preis auf dem Schwarzmarkt wurde in einem Range von fünf bis zehn Franken pro Gramm Cannabisblüten angegeben. In der Apotheke möchten die meisten Befragten nicht mehr als zehn Franken pro Gramm für Cannabisblüten bezahlen. Falls Steuern anfallen, sollten diese in Suchprävention investiert werden. Der optimale Verkaufsablauf beinhaltet nebst einer kompetenten Beratung, auch die Möglichkeit an den Produkten riechen bzw. das Produkt sichten zu können, dabei ist die Idee mit Testdöschen zu arbeiten aufgekommen. Wichtig ist den Befragten, dass in Apotheken eine Auswahl von Cannabisprodukten (Cannabisblüten, Haschisch etc.) angeboten werde, dies würde die Apotheken deutlich vom Schwarzmarkt abheben. Alle Befragten gaben an, selber direkt im Verkaufsraum der Apotheke einzukaufen, doch werde ein separater Raum für die Beratung und den Verkauf von Cannabis gewünscht, damit auch jene, die sich nicht «zeigen» möchten, in der Apotheke, geschützt vor neugierigen Blicken, Cannabis erwerben können. Dieser separate Raum wurde ebenfalls in Zusammenhang mit dem Thema Datenschutz aufgenommen. In Bezug auf den Datenschutz verlangten die Befragte, dass ihre persönlichen Daten nicht an dritte (Polizei, Strassenverkehrsamt etc.) weitergegeben werden.

Für die Gruppendiskussion mit denselben Personen wie hier interviewt wurden, sind folgende Themen für eine Vertiefung denkbar:

- Die Diskrepanz zwischen dem Wunsch der Akzeptanz von Cannabis in der Gesellschaft und dem gleichzeitigen Wunsch nach einem separaten Raum in Apotheken.
- Der optimale Verkaufsablauf mit dem Blick auf die Ansprache durch das Apothekenpersonal. Denn sollte ein (Daten-)Schutzbedürfnis vorliegen, ist es wichtig, dass die Kundschaft sich nicht vor andere Kundschaft zu ihrem Cannabiskauf äussern sollte. Wie könnte dies elegant gelöst werden?
- Indoor und Outdoor thematisieren, um zu sehen, ob cannabiskonsumierende Personen mit den Outdoorprodukten, die in der Studie angeboten werden, erreicht werden kann. Oder, was die Outdoorprodukte bieten müssten, damit auch Indoor-Cannabiskonsumierende in die Apotheke kämen.

- Wie wichtig Haschisch als Cannabisprodukt für die Zielgruppe ist. Falls kein Haschisch angeboten würde, wie diese Gruppe Konsumierende trotzdem in der Apotheke Cannabis erwerben würden.
- Mehrere Personen gaben an, keinen Tabak zu konsumieren, auch nicht um die Joints zu drehen. Da wäre interessant zu erfahren, mit was sie Cannabis insbesondere Haschisch vermischen, um diese Zusatzstoffe (z.B. Cannabisblätter) allenfalls auch in der Apotheke anbieten zu können.

5 Anhang 1: Frageroute

Einleitung

Ich stelle mich vor: Wissenschaftliche Mitarbeiterin am BIHM, verantwortlich für die Interviews und deren Auswertung während der Studie Cannabis Trail

Das Forschungsprojekt SCRIPT 2 (The ***S***afer ***C***annabis – ***R***esearch *I*n ***P***harmacies randomized controlled ***T***rial 2) befasst sich mit dem regulierten Verkauf von Cannabisprodukten in Apotheken in der Stadt Bern. Das Ziel ist: Erkenntnisse über die Auswirkungen auf das Konsum- und Kaufverhalten und das Befinden der Studienteilnehmenden zu gewinnen. Der Verkauf von Cannabisprodukten in Apotheken ist für den Herbst 2022 vorgesehen.

Es ist uns wichtig, die Erfahrungen, Ideen und Sichtweisen von Personen, die regelmässig Cannabis konsumieren, möglichst früh in die Planung des Cannabis-Angebotes und die Verkaufsabläufe in den Apotheken einzubeziehen, weshalb wir uns an Sie wenden.

Themen des Interviews sind Verkaufsabläufe, Cannabisprodukte und Datenschutz

Das Interview dauert zirka 60-90 Minuten.

Das Interview wird digital aufgenommen. Das ermöglicht uns, das Gesagte möglichst genau ins Schriftliche zu übertragen, um anschliessend eine computergestützte Auswertung vornehmen zu können. Nach der Auswertung wird die Aufnahme gelöscht, so dass das was Sie gesagt haben anonym ist und nicht mit Ihrer Person in Zusammenhang gebracht werden kann. Ihre Aussagen werden mit den Angaben der anderen Interviewpartner im Schlussbericht zusammengefasst. Ihr Name wird im Bericht nicht erscheinen.

Sie haben die Einverständniserklärung bereits unterzeichnet und eingereicht. Besten Dank.

Haben Sie noch Fragen bevor wir mit dem Interview beginnen? Sind Sie mit dem beschriebenen Vorgehen einverstanden? (→Aufnahme starten)

An dieser Stelle schon einmal ein herzliches Dankeschön, dass Sie sich für das Interview Zeit nehmen. Dann beginnen wir mit dem Interview.

Einstieg

- **Was verbinden Sie mit dem Konsum von Cannabis?** *Entspannung, Gesellschaft, Musik, Feierabend, …*

- **Zu welchen Tageszeiten konsumieren Sie Cannabis?**

Aktuelle Situation, Schwarzmarkt

Wir starten mit der Sicht auf die aktuelle Situation. Im Moment gibt es lediglich die Möglichkeit auf dem Schwarzmarkt THC-haltiges Cannabis zu kaufen.

- **Wie kommen Sie aktuell zu Cannabis?** *(von Freunden, Bekannten immer vom selben Händler/ Händlerin, auf der Strasse, bei Unbekannten, Mal so, mal so, …)*
- **Gibt es Aspekte, die Sie beim aktuellen Erwerb von Cannabis** *(auf dem Schwarzmarkt)* **anstrengend finden?**
    - Wenn ja, welche Aspekte sind das?
    - Und was wünschen Sie sich stattdessen?
- **Gibt es Aspekte, der aktuellen Situation des Cannabiserwerbs, die Sie als Vorteil sehen?**
    - Aspekte, die Sie auch zukünftig nicht missen möchten?

Apotheke

Nun machen wir einen gedanklichen Sprung in die Zukunft.

- **Wenn Sie an den Erwerb von Cannabis in einer Apotheke denken, was geht Ihnen da als erstes durch den Kopf?**
    - Wie sieht das aus?
    - Welche weiteren Details zeigen sich in Ihrer Vorstellung?

Verkaufsablauf

Nun kommen wir zum konkreten Verkaufsablauf in der Apotheke.

- **Stellen Sie sich einmal vor, Sie kommen in die Apotheke herein. Sie werden begrüsst, und nachher, wie geht es weiter?**
    - Bitte schildern Sie mir ihre Vorstellung so detailliert wie möglich.
    - Wie stellen Sie sich den Verkaufsablauf vor?

Cannabisprodukte

Nun kommen wir zu den Cannabisprodukten, die in der Apotheke angeboten werden sollen.

- **Welche Art von Cannabisprodukten würden Sie in einer Apotheke erwarten?** *(z.B. Blüten, Harz, Tinktur, Flüssigkeit zum Verdampfen, Tabletten, …)*
- **Was denken Sie, welche Produkte würden Sie selber kaufen?**
- **Welche Produkte würden Sie ausprobieren wollen?**
- **Auf was legen Sie besonderen Wert bei der Produktewahl?** (*Z.B. THC-Konzentration, Verpackung, Darreichungsform, Reinheitsgrad, …)*

Vor- und Nachteile Cannabisverkauf in Apotheken

- **Sehen Sie Vorteile darin Cannabisprodukte in der Apotheke kaufen zu können?**
    - Wenn ja, welche?
    - Was spricht dafür, Cannabis in der Apotheke zu kaufen?
- **Sehen Sie auch Nachteile, beim Erwerb von Cannabis in der Apotheke?**
    - Wenn ja, welche?
    - Was könnte Sie davon abhalten Cannabis in der Apotheke zu kaufen?

Datenschutz

Da der Handel mit Cannabis sowie das Konsumieren und Auf-sich-tragen von Cannabis in grösseren Mengen immer noch illegal ist, spreche ich gerne das Thema Datenschutz an.

- **Ist Datenschutz in Bezug auf den Erwerb von Cannabis in Apotheken ein Thema?**
    - Wenn ja, welche Aspekte sind Ihnen da wichtig?
    - Welche Aspekte kommen Ihnen dazu noch in den Sinn?
    - Welche Vorkehrungen würden Sie begrüssen?

Muss-Kriterien

Jetzt haben wir den Erwerb von Cannabis in der Apotheke aus verschiedenen Seiten beleuchtet und über verschiedenes gesprochen.

- **Wenn Sie an den Cannabiserwerb in Apotheken denken, was *muss* unbedingt beachtet werden, damit Sie zukünftig Cannabis in der Apotheke erwerben?**
    - Ev. vorher genannte Vorteile des Schwarzmarktes aufnehmen und fragen:

Andere Perspektiven einholen

In einem weiteren Schritt geht es uns darum, Ihre Einschätzung einzuholen, wie andere Cannabiskonsumierende den Cannabiserwerb in Apotheken sehen könnten.

- **Wenn Sie nun an Cannabiskonsumierende denken, die Sie kennen, was denken Sie, was ist diesen Personen besonders wichtig, wenn es um den Erwerb von Cannabis in Apotheken geht?**
- **Denken Sie, dass das Alter von Cannabiskonsumierenden einen Unterschied macht in Bezug auf den Erwerb von Cannabis in Apotheken?**
    - Falls ja, worin denken Sie, liegen die Unterschiede? Bitte machen Sie dazu nähere Angaben.
    - Falls nein, wo denken Sie, liegen die Gemeinsamkeiten von Cannabiskonsumierenden?

Kosten / Preis

Nun haben wir über Cannabisprodukte, Verkaufsablauf in Apotheken und auch über den Schwarzmarkt gesprochen. Uns interessieren auch die Preise.

- **Welche Art Cannabis kaufen Sie aktuell auf dem Schwarzmarkt?**
  - In welcher Menge pro Einkauf?
  - Wie viel bezahlen Sie dafür? *(In CHF)*
  - *Falls mögliche, nennen Sie mir den Preis pro Gramm?*
- ***Was wären Sie bereit in der Apotheke für eine vergleichbare Menge Cannabis zu bezahlen?***
  - *Falls mögliche, nennen Sie mir den Preis pro Gramm?*

Sozio-Demographische Angaben

Damit wir die Stichprobe möglichst gut beschreiben können, stelle ich Ihnen noch ein paar Fragen zu Ihrer Person.

- **Welchen Beruf üben Sie zurzeit aus?**
  - In welchem Umfang?
- **Wie beschreiben Sie Ihre aktuelle Wohnsituation?**

allein / bei meiner Ursprungsfamilie / in einer WG / in einer Partnerschaft

Dank und Abschluss

Wir sind fast am Ende des Interviews angelangt.

- **Gibt es nun noch etwas, worüber wir nicht gesprochen haben, Sie jedoch denken, das sei im Zusammenhang mit dem Thema wichtig?**
  - Oder gibt es etwas, das Sie uns unbedingt noch mit auf den Weg geben möchten?

Besten Dank für das Interview und Ihre wertvollen Antworten, sowie auch für die Zeit, die Sie sich dafür genommen haben. Gerne wende ich mich erneut an Sie, für weitere Interviews.

6 Anhang 2: Informed Consent

Ihre Erfahrungen mit Cannabis sind gefragt!

Sehr geehrte Interessierte

Mit der Änderung des Betäubungsmittelgesetzes, die im Mai 2021 in Kraft getreten ist, wird es nun möglich, wissenschaftliche Untersuchungen zum nicht medizinischen Cannabiskonsum durchzuführen.

Eine Studie zum regulierten Erwerb und Konsum von Cannabisprodukten in Berner Apotheken ist für den Herbst 2022 geplant und wird von der Universität Bern durchgeführt. Das übergeordnete Ziel der Studie ist es zu evaluieren, wie sich der regulierte Cannabiserwerb auf das Konsumverhalten, die Gesundheit und das Sozialleben der bereits Cannabis konsumierenden Personen im Vergleich zu Personen, die ihr Cannabis weiterhin auf dem Schwarzmarkt erwerben, auswirkt.

Um Verkaufsabläufe und Cannabisprodukte möglichst kundengerecht aufzubereiten und einzurichten, führen wir Einzelinterviews durch und gründen gegebenenfalls eine sogenannte partizipative Begleitgruppe. Für die Einzelinterviews und die partizipative Begleitgruppe suchen wir Menschen, die bereits regelmässig Cannabis zum Genuss konsumieren.

Ziel der Arbeit ist es, mit Menschen, die bereits regelmässig Cannabis zum Genuss konsumieren, ein kundengerechtes Angebot in den Apotheken für die bevorstehende Studie zu schaffen.

Vorgehen
Es können auf zwei Arten Informationen gesammelt werden. Zum einen mit Einzelinterviews, zum anderen in einer sogenannten partizipativen Begleitgruppe, wo Themen in einem Gruppengespräch diskutiert werden. Zurzeit ist noch offen, ob beide Datenerhebungsverfahren zum Einsatz kommen. Wir starten mit den Einzelinterviews und werden Sie allenfalls später zu einem Gruppengespräch einladen.

Grundsätzliches zu den Einzelinterviews
- Einzelinterviews dauern zirka 60-90 Minuten und werden digital (z.B via Zoom) durchgeführt.
- Die Interviews werden digital aufgezeichnet (Audio), anschliessend verschriftlicht und inhaltsanalytisch sowie anonymisiert ausgewertet.
- Die Interviews werden mit CHF 30.- pro Stunde vergütet.

Arbeit in der partizipativen Begleitgruppe
- Ein Treffen der Begleitgruppe dauert zirka 2-3 Stunden und findet in den Räumen der Universität Bern statt
- Acht bis zehn Teilnehmende
- Das/ Die Gruppentreffen werden digital aufgezeichnet (Audio), anschliessend verschriftlicht und inhaltsanalytisch sowie anonymisiert ausgewertet.
- Die Schweigepflicht über alle persönlichen Informationen bzgl. der Teilnehmenden, die erarbeiteten und diskutierten Inhalte, sowie der unterschiedlichen Meinungen innerhalb der Gruppe ist auch über die gemeinsame Arbeit hinaus verpflichtend und gilt sowohl für Teilnehmende wie Forschende.
- Die Sitzungen werden mit CHF 30.- pro Stunde vergütet.

Das bringen Sie mit:

- Interesse an der aktiven Auseinandersetzung mit dem Thema Cannabiserwerb in Apotheken
- Diskussionsbereitschaft, mit der Fähigkeit, andere Meinungen gelten zu lassen
- Regelmässiger Cannabiskonsum (mindestens monatlich, mit THC Gehalt >1% zum Genuss)
- Mindestalter von 18 Jahren

Ausschlusskriterien

Verbandstätigkeit oder ein Beruf, welcher mit Cannabis oder dessen Konsum in Verbindung steht.
Personen, welche ausschliesslich aus medizinischen Gründen Cannabis konsumieren.
Bei der Auswahl der Teilnehmenden wird auf Alter, Geschlecht und Konsumverhalten geachtet.

Haben Sie Fragen? Dann melden Sie sich entweder bei der Person, die Sie angefragt hat oder bei Beatrice Metry unter beatrice.metry@biham.unibe.ch oder Anna Schöni unter anna.schoeni@biham.unibe.ch

Wir freuen uns auf Ihre Kontaktaufnahme!
Beste Grüsse

Leiterin der Einzelinterviews und der partizipativen Begleitgruppe	Aus der Forschungsgruppe
Beatrice Metry	Anna Schöni

Einverständniserklärung

Ich habe die obenstehende Information für Teilnehmende der Einzelinterviews und der partizipativen Begleitgruppe zum Thema *Cannabiskonsum* gelesen und verstanden, worum es in den bevorstehenden Gesprächen gehen wird. Ich akzeptiere die Informationen wie auch die darin beschriebenen Verantwortlichkeiten und die Form der Zusammenarbeit. Sollte ich noch Fragen haben, wende ich mich direkt an Anna Schöni aus der Forschungsgruppe oder an die Leiterin der Einzelinterviews und der partizipativen Begleitgruppe Beatrice Metry.

Ich weiss, dass ich die Teilnahme an den Interviews bzw. der partizipativen Begleitgruppe jederzeit ohne Grund und ohne negative Folgen rückgängig machen kann. Ich weiss, dass die gesammelten Daten absolut vertraulich behandelt werden, und dass sie nicht mit meiner Person in Verbindung gebracht werden können.

Ich bestätige mit meiner Unterschrift, dass ich die Erklärungen verstanden habe, und dass mit dem beschriebenen Vorgehen einverstanden bin.

Name und Vorname: ____

Adresse: ____

Telefonnummer: ____

Ort, Datum: ____

Unterschrift: ____

7 Anhang 3: Glossar

Begriff	Erklärung
Blue Cookie	Blue Cookies ist eine indicadominierte Hybridkreuzung von zwei schwergewichtigen Hanfsorten, Girl Scout Cookies und Blueberry. Blue Cookies versetzt die Konsumierenden in Euphorie. Die Wirkung beginnt im Kopf, anschliessend verteilt sie sich über den ganzen Körper und führt zur Entspannung. Süße Beerenaromen verschmelzen mit erdigen Kirschnoten. Anfänger sollten sich Blue Cookies mit Vorsicht nähern. Für Cannabisgewohnte Personen ist die Potenz dieser Sorte perfekt. Möglicherweise stoßen Sie auch auf eine andere Variation von Blue Cookies, einem ausgewählten Phänotyp von Girl Scout Cookies, dem die Blueberry-Genetik fehlt.
BHO	Butan-Hasch-Oel<br>Mehr dazu unter ***Wax***
Cannabidiol (CBD)	Cannabidiol ist ein Cannabinoid aus dem weiblichen Hanf. Es sind entkrampfende, entzündungshemmende, angstlösende und gegen Übelkeit gerichtete Wirkungen beschrieben. CBD hat keine psychotrope Wirkung und fällt deshalb nicht unter das Betäubungsmittelgesetz.
Cannabinoide	Cannabinoide sind natürliche Substanzen, die aus indischem Hanf (Cannabis sativa) gewonnen werden, sowie synthetische Analoga (künstlich hergestellte, ähnliche Substanzen). Die wirksamste Substanz ist das Tetrahydrocannabinol (THC).
Cannabis-Edibles	Esswaren, die mit Cannabis versetzt sind. Z.B. Cookies, Kuchen, Fruchtgummi, Brownies, Honig, Getränke, ….<br>Aufgrund der psychotropen Wirkung fallen Edibles unter das BetmG und nicht unter das Lebensmittelgesetz (Erl. BetmVD, S. 9) und benötigen eine kindersichere Verpackung (ebd., Art. 11, S. 10)
Cannabis Social Club	ist ein im Jahr 2005 von der paneuropäischen Organisation ENCOD vorgeschlagenes Modellprojekt, um eine legale Anbau- und Vertriebsmöglichkeit von Cannabis als Rauschmittel an volljährige Personen zu ermöglichen. Ein Cannabis Social Club ist ein nichtkommerzieller Verein, welcher den professionellen, kollektiven Anbau einer limitierten Menge von Cannabis organisiert, um die persönlichen Bedürfnisse der volljährigen Clubmitglieder zu decken. Cannabis Social Clubs gibt es bisher in Spanien, Belgien, Niederlanden, Oesterreich, Frankreich, Deutschland, Italien, Slowenien.<br>https://de.wikipedia.org/wiki/Cannabis_Social_Club
Cannabis-ruderalis	Selbstblühende Hanfpflanzen
CannaTrade	Internationale Cannabis Expo in Bern<br>Über 250 Aussteller aus aller Welt präsentieren bewährte Produkte, Neuheiten und Innovationen.<br>https://www.cannatrade.ch/de/

Charas	eine handgemachte, typische Art des Haschisch, welches aus Indien kommt. Der wird nicht wie im arabischen Raum durch Sieben von trockenem Blütenmaterial gewonnen, sondern aus der frischen Pflanze. Aufwendig und zeitintensiv werden dafür Hanfblüten durch sanftes Massieren solange zwischen den Händen gerieben, bis das Cannabisharz an der Handinnenfläche kleben bleibt und anschliessend zu Knollen/ Taler ab den Händen gerieben. Die Hanfblüten werden 2-3 Wochen bevor sie erntereif sind und der THC Gehalt in der Pflanze am höchsten ist, benutzt, um das heilige Harz zu ernten.<br>https://www.bushplanet.com/Was_ist_Charas
Dabs (Tupf)	Stark THC-haltige Cannabisextrakte. Der Grund für diese Bezeichnung ist, dass schon der Konsum eines Dabs (Tupfens) ausreicht. Dabs gibt es in unterschiedlichen Ausprägungen.<br>***Shatter*** tendiert zu einem hohen THC-Anteil. Es hat die Form einer harten, glasartigen Substanz, die häufig Bernstein ähnelt. Die meisten Shattersorten weisen einen THC-Gehalt zwischen 70 und 90 % auf.<br>***Wax*** (Wachs) besitzt eine wesentlich weichere Textur, etwa wie Knetmasse. Der THC-Gehalt von Wax liegt im Durchschnitt bei 50 bis 80 %.<br>***Sugar Wax*** hat eine härtere Konsistenz als reines Wax. Bei Berührungen bricht es entzwei und hat eine gewisse Ähnlichkeit mit Zucker. Geschmack und Terpenprofil von Sugar Wax sind meistens besser als bei Wax, dennoch hat es einen hohen THC-Gehalt von durchschnittlich 60 bis 90 %.<br>***Crumble*** ist genau das, wonach es sich anhört. Es ist ein Wachs, das bei Berührung zu kleinen Krümeln zerfällt, manchmal sogar zu einer pulverähnlichen Substanz. Crumble wird hergestellt, indem es während des Extraktionsprozesses verschiedenen Vakuummengen und Temperaturen ausgesetzt wird. Crumble besitzt ebenfalls einen durchschnittlichen THC-Gehalt von 60 bis 80 %.<br>***Budder*** hat eine klebrige Konsistenz, die der Butter aus dem Lebensmittelgeschäft ähnelt. Durch sein reiches Aroma und Terpenprofil in Verbindung mit THC-Anteilen zwischen 50 und 70 % ist Budder eine milde Alternative zu Shatter.<br>***Live Resin*** wird mithilfe der Extraktion von frischen statt von getrockneten Cannabisblüten hergestellt. Live Resin ist ein Konzentrat, das unglaublich „terpy" schmeckt (voller Terpen-Geschmack) und für gewöhnlich einen hohen THC-Gehalt zwischen 80 und 90 % oder noch höher hat.<br>***CO2 Sapp*** verfügt über ein reiches Terpenprofil. CO2 Sapp wird größtenteils wie Live Resin hergestellt. Sein THC-Gehalt beträgt im Durchschnitt rund 50 bis 60 %, was es zu einer tollen Alternative für alle macht, die nach einem intensiven Rausch oder High suchen.

	The Pure ist eines der stärksten Formen der Cannabiskonzentrate, die auf dem Markt erhältlich sind. The Pure ist eine klare Flüssigkeit, die oft mit anderen Zutaten wie Apfel-, Trauben- oder Zitronenterpenen angereichert wird, um ihm Geschmack zu verleihen. Der THC-Gehalt liegt bei durchschnittlich 99 %.<br>***Rosin*** ist ein Typ des Cannabisextrakts, für dessen Herstellung gehärtete oder getrocknete Cannabisblüten erhitzt werden. Sobald die Blüten unter Hitzeeinwirkung zusammengepresst werden, wird Harz abgesondert. Dieses Harz wird dann eingesammelt und konsumiert. Im Durchschnitt enthält Rosin einen THC-Gehalt von 60 % oder mehr.
Dabbing	Das Inhalieren von verdampftem Cannabiskonzentrat durch einen als Dab Rig bekannten Glasapparat.
(Delta-9-)Tetrahydrocannabinol (THC)	ist eine psychoaktive Substanz, die zu den Cannabinoiden zählt. Die Substanz kommt in Pflanzen der Gattung Hanf vor und ihr wird der Hauptanteil der berauschenden Wirkung zugesprochen
dunk	Die Cannabissorte Dunk-a-Roos ist ein sativadominierter Hybrid
Edibles	***Edibles*** ist der Ausdruck für Lebensmittel, denen Cannabis-Extrakte beigefügt wurden. Häufig kommen sie in Form von Backwaren, wie Keksen und Brownies, aber auch in Getränken, Broten und Süßigkeiten vor. Beim Konsum von Edibles kann davon ausgegangen werden, dass die aktiven Komponenten aus den Extrakten länger brauchen, um zu wirken, da sie durch das Verdauungssystem absorbiert werden müssen.
El Kif	So heisst Cannabis in Marokko, wo Cannabis hauptsächlich im Rif-Gebirge angebaut wird.
EMCDDA	***E***uropean ***M***onitoring ***C***entre for ***D***rugs and ***D***rug ***A***ddiction zu deutsch<br>Europäische Beobachtungsstelle für Drogen und Drogensucht (EBDD). Dies ist eine Agentur der Europäischen Union mit Sitz in der portugiesischen Hauptstadt Lissabon. Sie gibt einen jährlichen Bericht über den Stand der Drogenproblematik in Europa heraus.
Entourage-Effekt	Der ***Entourage Effekt*** ist die Annahme, dass alle Verbindungen der Pflanze zusammenarbeiten und zusammen eine bessere Wirkung erzielen als alleine. Hanfpflanzen enthalten mehr als 120 verschiedene Phytocannabinoide. Diese wirken auf das Endocannabinoidsystem (ECS) Ihres Körpers, welches eine Reihe von Funktionen und Prozesse reguliert und im Gleichgewicht hält. Cannabidiol (CBD) und Tetrahydrocannabinol (THC) sind zwei der am besten erforschten Phytocannabinoide.<br>Der Begriff ***Entourage-Effekt*** wurde erstmals 1998 von Dr. Ben-Shabat geprägt. Das Konzept wurde vor allem von Dr. Etahan Russo erläutert und erweitert.

	Untersuchungen legen nahe, dass die gemeinsame Einnahme - zusammen mit anderen natürlichen Verbindungen in der Cannabispflanze, die als Terpene oder Terpenoide bekannt sind - wirksamer ist als die alleinige Einnahme von CBD.
Fufi	5g Cannabis = 50 Franken
Gorilla Blue	Gorilla Blue ist eine Cannabis-Pflanze mit medizinischen und heilenden Eigenschaften, da sie ein Verbündeter gegen Schmerzen ist und eine große beruhigende Wirkung auf den Körper ausübt.
Growshop	Bietet alles für die Cannabiszucht zu Hause an<br>Zelte, Bewässerungssysteme, Samen, Beleuchtung, Belüftung, Substrate, Pflanzenschutzmittel, Verpackungsmaterial, …
Haschisch / Hasch / Shit (Harz)	Haschisch ist das gesammelte und meistens gepresste "Harz" der Hanfpflanze. Es kann nicht nur aus den Blüten, sondern auch aus mit Harzen besetzten Blättern gewonnen werden. Je nach Qualität und Herstellungsmethode schwankt seine Farbe von hellem grau-braun bis zu mattem schwarz.
Haze	Haze Sorten haben einen sehr hohen THC-Gehalt, was beim Rauchen eine ausgeprägt starke Wirkung zur Folge hat. Ihren großen Ruhm und die hohe Nachfrage verdankt die Haze allerdings ihrer sehr speziellen Wirkung beim Konsum. Durch den hohen Sativa Anteil ist das High oft sogar psychedelisch und knallt voll in den Kopf.
Headshop	bezeichnet man meist kleine Ladengeschäfte, die Zubehör für den Konsum von Cannabis verkaufen. Verkauft werden etwa Wasserpfeifen, Bongs, Vaporizer oder langes Zigarettenpapier zum Drehen von Joints, aber auch normale Tabakspfeifen.
Hybrid-Hanf	Bei einer Vielzahl der am Markt vertretenen Cannabis-Sorten handelt es sich um sogenannte Hybriden; das bedeutet, sie sind das Produkt einer Kreuzung zwischen mehreren Cannabis-Arten.<br>Obwohl die drei Arten ***Sativa, Indica*** und ***Ruderalis*** unterschiedlichen Cannabisfamilien angehören, lassen sich alle untereinander kreuzen.<br>***Ziel ist*** es neue hoch-potente Sorten mit einer speziellen Genetik und den bestmöglichen Eigenschaften von mehreren Sorten zu nutzen, um diese in einer zu vereinen.
Indoor-Hanf (Indica)	Indoor-Hanf wächst in Räumen, meist bei künstlichem Licht und Bewässerung mit Zugange von Substraten. Sei beliebter, da geschmacksintensiver und höherer THC-Gehalt
Jack Herer	Jack Herer ist einer der kultigsten Cannabis- und Hanfaktivisten. Er ist vor allem für sein Buch ‚Die Wiederentdeckung der Nutzpflanze Hanf' von 1985 bekannt.<br>Nur wenige Namen in der Cannabisindustrie rufen Emotionen hervor wie Jack Herer. Jack ist wohl einer kultigsten Cannabis-/Hanf-Aktivisten in der Geschichte und seine Arbeit hat deutliche Spuren in der

	Cannabisindustrie und der Legalisierungsbewegung hinterlassen.<br>Trotz seiner ihn auszeichnenden radikalen und leidenschaftlichen Ansichten über Cannabis, sowie über das Verbot und das Potenzial der Hanfpflanze, war Jack jedoch nicht immer der Weed-Enthusiast, den wir heute kennen. In der Tat probierte er Cannabis erstmals, als er weit jenseits der 30 war. 2010 verschied er traurigerweise aufgrund von Komplikationen nach einer Herzattacke
Jack Herer Gras	Die Sorte findet man weltweit von Kalifornien bis Amsterdam und darüber hinaus.<br>In den 1990ern ursprünglich in Holland gezüchtet, war das Ziel, eine Pflanze zu entwickeln, die eine erhebende Wirkung liefert, die üblicherweise mit Haze-Sorten in Verbindung gebracht wird, aber mit einer reduzierten Blütezeit.<br>Die genaue Genetik von Jack Herer ist ein streng gehütetes Geheimnis und so etwas wie ein Mysterium, aber es wird allgemein angenommen, dass Jack eine 50%ige Haze-Kreuzung mit etwas Northern Lights und Skunk ist. All diese Sorten sind selbst legendär und stark.
Kalifornischer Hanf	Im online-Handel erhältlich, schön verpackt, mit Zippverschluss
Kief / Keef /Skuff	pudrige Substanz aus den Pflanzenhaaren der Hanfpflanze
Knaster	Alter Name für Nutz-Hanf. Die Blüten davon wurden bereits früher geraucht. Heute wird Knaster auch als Tabakersatz verwendet. Oft enthält Knaster heute weitere pflanzliche Bestandteile wie bspw. Rotklee, Holunderblätter, Löwenzahn, Helmkraut, weitere fermentierte Bestandteile.
Kush	Kush ist eine alte Cannabissorte, die zur Art Cannabis indica gehört. Die Cannabissorte stammt ursprünglich aus Afghanistan, Pakistan, Iran und Nordindien.
Lappe	10g Cannabis = 100 Franken
Marihuana / Weed (Blüten, Gras)	Marihuana oder Gras bezeichnet man die getrockneten Blüten der weiblichen Hanfpflanze. An Drüsenhaaren auf diesen Blüten sitzt das "Harz" der Pflanze, mit seinen hohen Konzentrationen von THC, CBD und anderen Cannabinoiden. Marihuana ist je nach Qualität, Herkunft, Anbaumethode und Trocknungsgrad üblicherweise grün bis bräunlich, teilweise auch weiß oder leicht lila.
Moonrock	Hanfblüte getränkt mit Hanföl und bestäubt mit Hanfblütenstaub<br>Der THC-Gehalt ist himmelhoch. Ein Moonrock ist in der Tat eine Kombination aus Cannabisspitzen, Haschischöl und Kief.
NPS	***N***eue ***p***sychoaktive ***S***ubstanzen (synthetische Cannabinoide)
Outdoor-Hanf (Sativa)	Wächst draussen, auf dem Feld oder in Töpfen. Meist naturbelassener Hanf. Hat an den Blüten viele kleine

	Blättchen, die vor dem Konsum weggezupft werden müssen.
PIED	***p***erformance and ***i***mage ***e***nhancing ***d***rugs<br>Werden wie NPS online gehandelt
«Praliné»	Joint mit Hanfblüten und Harz
Roter Libanese	Ist Haschisch. Die Pflanzen, aus denen „Roter Libanese" gewonnen wird, werden so lange auf dem Feld stehen gelassen, bis die Harzdrüsen voll ausgereift sind und eine goldrote Farbe angenommen haben. Dann werden die ganzen Pflanzen über dem Boden abgeschnitten und in Kisten abgeklopft. Die reifen Harzdrüsen brechen sehr leicht ab, fallen zu Boden und werden dann zu sogenannten "Pucks" gepresst.<br>Roter Libanese ist die Bezeichnung für eine bestimmte Art von Haschisch aus dem Libanon. Es handelt sich dabei nicht um eine Sortenbezeichnung im engeren Sinn: Viele einschlägige Publikationen gehen davon aus, dass die Wortschöpfung „Roter Libanese“ – unabhängig von Herstellungsweise oder Verarbeitung – lediglich das Herkunftsland Libanon. Andere Interpretationen machen die rote Erde im libanesischen Bekaa-Tal für den Namen „Roter Libanese“ verantwortlich. Spezifischer wird der Rote Libanese als Haschisch aus dem Libanon-Gebirge beschrieben, der für gewöhnlich in flachgepressten Leinenbeuteln verpackt wird und sich durch einen hohen Harzgehalt auszeichnet.
Schwarzer Afghane	Ist Haschisch. „Schwarzer Afghane" wird durch Abreiben des Harzes von der wachsenden Pflanze gewonnen. Nach dem Abreiben ist seine Farbe noch grün und wird erst durch langes Kneten schwarz. Schwarzer Afghane stammt aus Afghanistan und ist äußerlich schwarz und innerlich bräunlich. Er hat einen intensiven Geruch, einen hohen THC-Gehalt und wird meist mit Schaf- oder Ziegenfett geschmeidig gemacht. Afghane brennt sehr langsam und schwer und macht schnell müde. Das gleiche gilt für Schwarzen Pakistani, der allerdings eine härtere Konsistenz aufweist.
Space Cookie	Cookie, das mit Cannabis versetzt ist, z.B. mit Cannabisbutter oder Cannabisschokolade
Spice	Synthetische Cannabinoide, ahmen Wirkung von THC nach
Streckmittel von Marihuana	Brix<br>Sand, Quarzsand<br>Speckstein<br>Zucker<br>Haarspray<br>Haze-Spray (Raum-, Duftspray)<br>Glas<br>Pflanzliche Streckmittel, Mehl, Gewürze (selten)<br>Blei (giftigstes Streckmittel)<br>Phosphor / Kaliumdünger
Tabakersatz	Kräuterzigarette ohne Nikotin

Terpene	Terpene kommen in allen Pflanzen vor, besonders in den Blüten sind die Terpen Konzentrationen oft am höchsten. Der Sinn ist ganz einfach: Terpene locken durch ihr Aroma Insekten zur Bestäubung an. Gleichzeitig schützen Terpene aber auch vor Schädlingen, sozusagen als natürliches Insektizid. Die Terpene sind eine stark heterogene und sehr große Gruppe chemischer Verbindungen, die als sekundäre Inhaltsstoffe in Organismen natürlich vorkommen.<br>Terpene = ätherische Öle (ca. 120 verschiedene)
Tweni	2g Cannabis = 20 Franken
Vermischte Cannabisprodukte	Cannabisprodukte, bei denen Betäubungsmittel des Wirkungstyps Cannabis vom Hersteller mit Zusatzstoffen vermischt werden, um beispielsweise deren Aufnahme, Wirkung, Geschmack oder Aussehen zu modifizieren.
Cannabis- ***Wax*** / -Crumbels / -Honeycumb / -Budder	Cannabis-Wax ist eine Form von BHO (Butan Hasch-Öl). Es wird unter der Verwendung von Butan (ein Flüssiggas) als Lösungsmittel für die Extraktion der wichtigsten Cannabinoide und Terpene aus den Cannabisblüten und dem Trim hergestellt.<br><br>Das daraus entstehende Gemisch wird dann zu einem Konzentrat verarbeitet, das ein bisschen wie Bienenwachs aussieht. Der Herstellungsprozess von Cannabis-Wax variiert und kann in einer Vielzahl verschiedener Produkte mit einzigartigen Beschaffenheiten und Farben resultieren.
Wietpas	«Gras-Ausweis»<br>Am 1. Mai 2012 führten drei südliche Provinzen (Limburg, Noord-Brabant und Zeeland) als Modellversuch den „Wietpas“ (dt. „Gras-Ausweis“) ein, um ihn am Ende des Jahres in den ganzen Niederlanden einzuführen. Dies scheiterte aufgrund von Protest und beschränkte sich bereits nach kurzer Zeit wieder nur auf diese drei Provinzen, wobei sich jede Stadt dort auch eigenständig gegen den Wietpas entscheiden darf. Mit dem Wietpas wurde sichergestellt, dass kein Drogentourismus entsteht. Nur wer einen Wietpas hat, kann in Coffeeshops einkaufen.
50er-Säckli	Mit «50ig» ist der Preis gemeint, nicht die Menge des Inhalts/ Produktes
420 / 4:20/ 4/20	Fourtwenty ist in den USA ein gängiges Codewort für den regelmässigen Cannabiskonsum<br>Mehr dazu unter<br>https://de.wikipedia.org/wiki/420_(Cannabis-Kultur)

Auswertung der Gruppendiskussion zu SCRIPT

Die Gruppendiskussion wurde mit Cannabiskonsumierenden Personen geführt

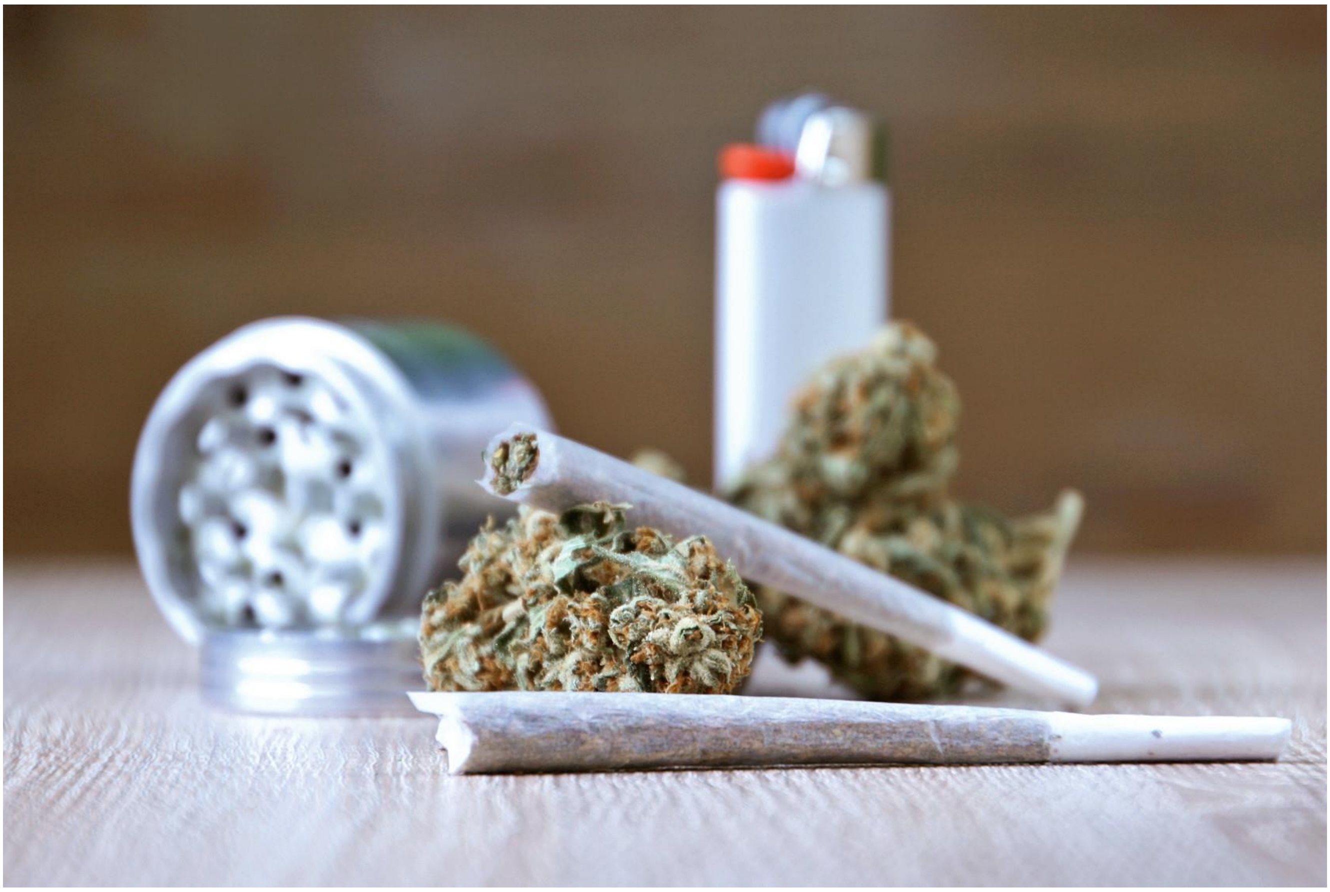

Beatrice Metry

Berner Institut für Hausarztmedizin

Juli 2022

Inhaltsverzeichnis

1 Einleitung 3
2 Stichprobe 3
3 Ergebnisse aus der Gruppendiskussion 4
3.1 Bindung an SCRIPT 4
3.2 Gebrauch von Vaporizer und E-Joints 4
3.3 Cannabisprodukte 5
3.3.1 Blüten 5
3.3.2 Harze 7
3.3.3 E-Liquids 7
3.3.4 Oel 8
3.4 Präsentation der Produkte 8
3.5 Prävention in der Apotheke 10
3.6 Mögliche Verkaufsstellen 11
3.7 Herausforderungen der Forschungsgruppe 12
3.8 Diverses 13
4 Fazit 13
5 Anhang 1: Frageroute Gruppendiskussion 15

1 Einleitung

Ende 2021 und anfangs 2022 wurden Einzelinterviews mit Cannabiskonsumierenden Personen geführt und erste Erkenntnisse für die Umsetzung von SCRIPT daraus gewonnen. Seither hat die Forschungsgruppe weitere Vorbereitungsarbeiten für die Umsetzung der Studie ausgeführt. Aktuelle Themen, zu denen die Perspektive der Endkund:innen in einer Gruppendiskussion eingeholt werden sollten, waren:

- Informationen zum aktuellen Stand der Studie abgeben, insbesondere das Studiendesign vorstellen
- Vaporizer, E-Joints
- Präsentation der vorliegenden Cannabisprodukte
- Prävention in der Apotheke
- Mögliche Verkaufsstellen, ausser die Apotheken
- Herausforderungen für die Forschenden

Das Thema der alternativen Verkaufsstellen wurde durch den parlamentarischen Vorstoss «Keine Cannabis-Pilotversuche in bernischen Apotheken» (RRB-Nr. 674/2022), eingereicht durch die SVP und EDU), sehr aktuell. Der Vorstoss besteht darauf keine Genussmittel in bernischen Apotheken zu verkaufen. Dies steht im Widerspruch zu SCRIPT, denn die Studie richtet sich an Genusskonsument:innen von Cannabis und hat bisher als Vertriebsorte Apotheken avisiert und entsprechend in die Studie involviert. Der Regierungsrat hat am 22. Juni 2022 das Postulat an den Grossen Rat des Kantons Bern weitergereicht, dies mit der Empfehlung das Postulat anzunehmen.

Der Auswertungsbericht skizziert die Stichprobe und wird anschliessend den Themen entlang der Frageroute, welche im Anhang zu finden ist, vorgestellt.

2 Stichprobe

Für die Gruppendiskussion wurden alle neun Personen, welche bereits bei den Einzelinterviews mitgemacht und den entsprechenden Informed consent unterzeichnet haben, angefragt. Via doodle wurde der Termin vereinbart.
Anwesend waren sechs Cannabiskonsumierende Personen, zwei Personen aus der Forschungsgruppe als Zuhörerinnen sowie die Diskussionsgruppenleiterin.

**Jahrgang:**	1957 bis 2002<br>1957, 1975, 1991, 1993, 2001, 2002		
**Geschlecht**	3 männlich	2 weiblich	1 non-binär

3 Ergebnisse aus der Gruppendiskussion

Die Ergebnisse werden nachfolgend entlang der Frageroute aufgeführt. Zahlen in runden Klammern weisen darauf hin, wie viele der Befragten in diese Richtung geantwortet haben. Befindet sich keine Klammer hinter der Aussage, handelt es sich um eine Einzelnennung. In eckigen Klammern wird der Absatz in der MAXQDA-Datei (A), in welchem das genannte Zitat gefunden werden kann, aufgeführt.

3.1 Bindung an SCRIPT

Das Studiendesign wurde vorgestellt und auf die randomisierte Verteilung in Start- und Kontrollgruppe hingewiesen. Die Kontrollgruppe kann 6 Monate später mit dem Bezug von Cannabis beginnen. Auf die Frage, ob die Teilnehmenden warten würden, wenn sie in die Kontrollgruppe kämen und allenfalls von einer anderen Studie angefragt würden, in der sie direkt Cannabis beziehen könnten, wurde wie folgt geantwortet: Eine Stimme äusserte, sie würde in beiden Studien mit machen, statt zu warten. Jedoch schätze sie es als sehr unwahrscheinlich ein, abgeworben zu werden. Die anderen meinten, sie würden der Studie zuliebe warten (5), denn ihr Konsum sei nach wie vor sichergestellt. Weitere Gründe, die fürs Warten sprechen, seien ein persönlicher Beitrag auf dem Weg der Legalisierung bzw. das Geschichte schreiben, auf dem Weg der Entkriminalisierung und Legalisierung von Cannabis in der Schweiz.

> Ich denke auch, kiffen für die Wissenschaft, das ist wie Geschichte schreiben, in dieser Phase. Es geht eine Entkriminalisierungs- und Legalisierungswelle global voran. [A:89]

Eine weitere Stimme legte den Fokus auf die Produktequalität, wie nachfolgendes Zitat unterstreicht:

> Und ich glaube, das ist eine meiner Hauptmotivationen, einfach an eine bessere Qualität ranzukommen, oder eben kontrollierte, gute Qualität. [A:90]

Eine kritische Äusserung betraf den Preis, wenn Cannabis in den Apotheken verkauft werde. Die Vorstellung sei, dass Cannabis teurer werde als auf dem Schwarzmarkt. Da gab eine andere Person zu bedenken, dass es auch genau andersrum sein könnte, dass der Schwarzmarkt teurer werde, weil Cannabis nun auch in Apotheken bezogen werden könne.

3.2 Gebrauch von Vaporizer und E-Joints

Auf die Frage wer von den Anwesenden bereits über Erfahrung mit einem Vaporizer oder einem e-Joint verfüge, gaben alle Personen (6) an, in unterschiedlichem Ausmass Cannabis mit einem Vaporizer konsumiert zu haben bzw. regelmässig auf diese Weise zu konsumieren. Eine Person äusserte, unter der Woche ausschliesslich zu vaporisieren und nur am Wochenende gedrehte Joints zu rauchen. Erfahrung mit e-Joints deklarierte lediglich eine Person. Die Befragten äusserten verschiedene kritische Aspekte der Vaporizer und e-Joints. Es fehle, das Sinnliche, das Genüssliche, das Gemeinsame (3), es habe nicht dieselbe Wirkung und es brauche Strom wie nachfolgendes Zitat untermalt:

> Ja, das hält mich auch davon ab. Etwas, das ich chargen muss. Da kannst du sagen, wir machen etwas für unsere Gesundheit, aber wir zerstören die Umwelt. [A:128]

Eine andere Stimme meinte, sie höre mehr Menschen husten, die Cannabis mit Vaporizer und e-Joints konsumieren würden. Vorteile wurden darin gesehen, dass es wesentlich gesünder sei zu vaporisieren (3), der Geschmack sei besser, intensiver (2), die Umgebung rieche weniger stark nach Cannabis nach dem Vaporisieren (2), es gehe schneller als einen Joint zu drehen und zu rauchen und die Reste nach dem Vaporisieren könnten noch zum Kochen und Backen verwertet werden, wie das folgende Zitat untermalt:

> Das Positive an dem ist, das Gras, das du geraucht hast, kannst du wiederverwerten. Es wird verdampft und du hast dann dieses braune Zeug drin und das kannst du noch in einen Kuchen reintun und das haut dich noch um. [A:130]

Eine Stimme äusserte e-Joints und Vaporizer würde sie lediglich als Ergänzung zu gedrehten Joints ansehen. Auch habe sie noch nie von einer Cannabiskonsumierenden Person gehört, die ganz auf Vaporizer umgestiegen sei.

Auf die Frage, unter welchen Umständen die Anwesenden, doch einen e-Joint dampfen oder Cannabis vaporisieren würden, wurde geäussert e-Joints der Studie zuliebe zu probieren (2) oder allenfalls auf einen langen Flug mitzunehmen. Drei Personen meinten, mit e-Joints gar nicht erst anfangen zu wollen. Mit Vaporisieren, gaben alle Teilnehmenden an, bereits Erfahrungen gemacht zu haben, doch haben nicht alle einen eigenen Vaporizer und sind auch (noch) nicht bereit ein eigenes Gerät zu kaufen. Zwei Personen meinten, sie würden ganz auf einen Vaporizer umstellen und auch einen kaufen, wenn es aus gesundheitlichen Gründen notwendig würde. Das zeigt nachfolgendes Zitat sehr schön:

> Eben, wenn ich ein Lungenproblem hätte. Ja, also schon aus gesundheitlichen Aspekten. Wenn mir ein Arzt/ eine Ärztin nahelegen würde, also jetzt sollten sie wirklich mit Rauchen aufhören. Dann würde ich vielleicht noch ein bisschen, dezent an einem Vaporizer ziehen. [A:399]

3.3 Cannabisprodukte

Den Teilnehmenden wurden die verschiedenen Produkte (Blüten, Harze, E-Liquid, Oel) vorgestellt und jeweils gefragt, ob es sie anspricht und ob sie die Produkte kaufen würden.

3.3.1 Blüten

Die vier vorgestellten Cannabisblütensorten würden von allen Teilnehmenden zumindest zum Ausprobieren gekauft. Die meisten der Befragten fanden die THC-Werte mit maximal 18% absolut ausreichend (5), eine Person äusserte, dass sie Blüten mit höheren THC-Werten gewohnt sei und deshalb etwas ernüchtert. Es ist auch diese Person, welche lieber Indoorblüten konsumiert, alle

anderen fanden Outdoorblüten absprechend. Ebenfalls positiv aufgefallen sind die Schweizer Bioqualität (3) und die ansprechenden Namen der verschiedenen Blüten (2). Der Preis wurde von drei Personen als klar zu teuer angesehen, eine dieser Personen wünschte sich einen Mengenrabatt einzuführen. Und eine weitere dieser drei Personen, meinte, diese Preise seien auf Amsterdam-Niveau. Wie nachfolgende Zitate belegen:

> Also so einmal werde ich das ganz sicher ausprobieren. Oder so, aber sonst sehe ich das im Moment eher so, falls der Schwarzmarkt nicht da ist. Egal, wie hart das jetzt tönt. Aber wenn ich 15 Franken mehr bezahlen muss für Gras, das maximal so gut ist, wie das welches ich eh schon habe und ich eh schon habe testen lassen, und es nicht gestreckt ist, dann sehe ich das nicht so. Vielleicht einfach einmal, weil ich Lust habe. [A:171]

> Es ist relativ teuer, von den Preisen her, würde ich sagen. 13.- Franken, das ist dann schon Amsterdam-Niveau. Outdoor, das bekommst du für einen Fünfliber und da stellt sich nachher wirklich die Frage nach der Effizienz und der Effektivität, wer bezahlt fast das Doppelte? Da habe ich das Gefühl, da holt man nur die Biologen ab, die etwas Gutes machen und den Schwarzmarkt nicht unterstützen wollen. [A:157]

Eine weitere Person fand, sie möchte die Produkte aus dem Schwarzmarkt und der Apotheke vergleichen. Die zeigt folgende Aussage:

> Ich würde es auch kaufen. Ich würde es vielleicht auch direkt in den Vergleich ziehen mit der gleichen Sorte vom Schwarzmarkt. Weil, das würde mich auch noch interessieren. [A:235]

Eine weitere Person fand, der Preis sei okay, sie bezahle aktuell für 5 Gramm auch 50 CHF, sie würde sich nicht jedes Mal den teuersten leisten, wie nachfolgendes Zitat untermalt:

> Also ich weiss jetzt nicht, ob ich immer auf diese 65 CHF gehen würde, ich denke eher nicht, weil es zu fest auf der Tasche liegen würde. Aber 50 CHF bezahle ich jetzt schon und 45 CHF ist sowieso weniger. Also, wenn ich das «tasten» (probieren) könnte, natürlich und es nice wäre, dann ja. [A:167]

Zwei weitere Personen äusserten, eher auf Qualität als den Preis zu achten.

Die Diskussion kam zum Thema Verhältnis THC zu CBD und welche Wirkung dies habe. Eine der teilnehmenden Personen klärte dies sowie die hohen THC-Werte auf dem Schwarzmarkt wie folgt auf:

> Ich weiss einfach, dass CBD THC-hemmend wirkt. Die meisten anderen Cannabinoide dämpfen das THC. Desto purer das Weed ist, also desto mehr THC drin ist, desto stärker wirkt es. [A:214]

> Ich habe das Gefühl, es hat sicher auch mit den Sorten zu tun. Und ja, ich glaube, es ist mehr nach Bereich. In der Medizin wird häufig mit hohen CBD-Anteilen gearbeitet, habe ich

> gemeint. Und ich glaube, es hat sich auf dem Schwarzmarkt in diese Richtung (hohe THC-Werte) entwickelt. Also vom Profitdenken her. Je mehr es "chlepft" desto besser kann man es verkaufen, desto teurer kann man es verkaufen. So würde ich das als Herleitung darlegen. [A:216]

3.3.2 Harze

Alle der Diskussionsrunde äusserten, sie würden die beiden Harzsorten ausprobieren, obwohl auch kritische Aspekte genannt wurden. So wurde der Preis als teuer eingestuft (4), eine Person fand, diese Preise seien ok, denn Hasch reiche weiter als Gras und in Amsterdam oder Barcelona würde man für 1 Gramm Hasch auch zwischen 20 und 30 Euro bezahlen. Eine weitere Person monierte das Verhältnis THC – CBD und fand, auf dem Schwarzmarkt wäre der THC-Gehalt deutlich höher. Eine weitere Stimme meinte, sie würde unbedingt Beratung in Anspruch nehmen, da ihr diese Namen nichts sagen würden. Wie bereits bei den Blüten, sind auch hier die Namen der Harze positiv, ansprechend aufgefallen (2). Die Auswahl von zwei verschiedenen Harzen sei ok, meinten zwei Stimmen, denn auf dem Schwarzmarkt gäbe es meist eine Sorte. Eine Person regte an, den Filterungsgrad des Harzes auszuweisen.

3.3.3 E-Liquids

Mit e-Liquids hat noch niemand der Anwesenden Erfahrung gesammelt. Vier Personen meinten, sie würden E-Liquids mindestens der Studie zuliebe ausprobieren. Zwei Personen fanden e-Liquids unsympathisch. Bereits der Name «e-Liquid» stosse ab. Und die ausgewiesenen «künstlichen» Bestandteile schreckten total ab, da auch unklar sei, wie sich diese auf die Gesundheit auswirken würden. Zwei Personen fanden, den Kauf eines weiteren Gerätes unverhältnismässig und eine dieser Personen würde es begrüssen, während der Studie ein Gerät zum Gebrauch ausgeliehen zu bekommen. Eine weitere Person führte aus, dass sie ganz bewusst keine e-Zigaretten rauche, weil sie im Bekanntenkreis die Beobachtung mache, dass mit e-Zigaretten mehr konsumiert werde. Es würden normale und zusätzlich e-Zigaretten konsumiert, auch seien letztere besser und überall verfügbar. Aus diesem Grund würde sie e-Joints lediglich einmal ausprobieren wollen, um keine Gewohnheit damit aufzubauen. Da entgegnete eine andere Person, dass genau diese Verfügbarkeit sie fasziniere und sich deshalb vorstellen könne e-Joints zu konsumieren. Diese Zwiespältigkeit zeigt das nachstehende Zitat:

> Das tönt nicht gerade sympathisch. Das wegen dem available finde ich noch interessant, weil es schon Situationen gibt, wo ich gerne noch schnell eins paffen würde, aber es ist nicht situationsadäquat. Und daher, könnte das noch interessant sein. [A:347]

3.3.4 Oel

Das in der Studie angebotene Öl ist auf Lebensmittelbasis und zum Verzehr gedacht. Diese Art Öl ist drei teilnehmenden Personen bekannt. Die eine Person nutze dies für alles, also zum Rauchen, in der Salatsauce, im Tee, im Jogurt und bei anderen Gelegenheiten. Eine andere Person gab an, dies zum Einreiben gegen Schmerzen zu nutzen und eine dritte Person zum Rauchen, bzw. anreichern von Joints. Diese Person macht darauf aufmerksam, dass das «übliche» THC-Öl (60-70%) sehr dunkel und klebrig sei und dadurch auch unhandlich bzw. unangenehm zum Wiederverschliessen. Alle Anwesenden gaben an, das vorgestellte THC-CBD-Öl zumindest zum Ausprobieren zu kaufen.

Bis am Schluss blieb unklar, ob die Anwesenden den Unterschied zwischen dem klebrigen THC-Öl und dem hier für den Verzehr angebotenen Öl realisiert haben.

3.4 Präsentation der Produkte

Ein zentrales Thema zeigte sich in der Transparenz der Produkte. Dabei wurde der Vergleich mit Frischtheken (2) und einer Schmuckvitrine geäussert. Bei beiden Varianten würde die Kundschaft persönlich bedient und ein Gespräch zwischen Apotheker:in und Kundschaft ergäbe sich ganz natürlich, dies auch im Hinblick auf eine (Präventions-)Beratung (2). Ein weiterer Vergleich wurde mit der dekorativen Kosmetik gemacht. Denkbar seien Testdöschen, die geöffnet werden können, um daran zu riechen und das Produkt anzusehen (3). Zur Variante Cannabis in «Konfitürengläser» zu präsentieren, äusserten sich alle Anwesenden positiv (6). Doch gilt das *Riechen können* auch als Kaufskriterium? Auch da waren sich alle Anwesenden einig, wenn auch aus anderen Motiven. Nein, es sei kein Kaufskiller, wenn am Produkt nicht gerochen werden dürfe (6). Dies wurde wie folgt begründet:

Während der Studie sei dies ok, werde Cannabis später für alle Menschen zugänglich, sollte dieser Punkt aufgenommen werden (4). Dies zeigt nachfolgendes Zitat:

> Das ist dann vielleicht erst der next level. Oder, wenn wir das hier jetzt etablieren. Aha, das kann man an öffentlichen Verkaufsstellen kaufen. Und dann denke ich, geht es nicht lange und dann ist es eben so, wie in Amsterdam, dass man ein Menu an der Theke hat. Aber dort kann man auch nicht immer daran riechen. [A:499]

Zwei weitere Stimmen äusserten, dass Cannabis häufig nicht so rieche, wie es schmecke.

> Und es kommt auch noch dazu, dass Gras häufig nicht so schmeckt, wie es von der Nase her riecht. Oftmals kann es mega-geil riechen und zum Rauchen mega-scheisse sein. [A:496]

Eine Person meinte, sie sei es gewohnt, dass sie keine Auswahl habe, dies im Sinn von «es gibt, was es hat». In die ähnliche Richtung äusserte sich eine weitere Person. Sie meinte, es hänge mit der

Grösse der Auswahl zusammen, ob das *Riechen können* wichtig sei, bei der aktuell überschaubaren Auswahl, sei dies nicht unbedingt notwendig. Das *Riechen können* wurde gegen Ende der Diskussion als Pluspunkt im Verkaufsprozess genannt und sollte auf Nachfragen möglich sein.

Das *Sehen können* scheint wichtiger zu sein. So äusserten sich mehrere Personen (4), sie möchten das Produkt sehen bevor sie es kaufen. Es wurde als eine Art Qualitätskontrolle beschrieben. So sei es wichtig zu sehen, wie die Blüte aussehe, ob es Blätter und Samen drin habe, wie untenstehendes Zitat aufzeigt:

> Du siehst, hat es höllenviele Blätter dran, hat es Sämchen drin. Und sind es schöne Blüten. [A: 472]

Die Teilnehmenden (4) sprachen von einer transparenten Verpackung, die die Sicht auf das Produkt möglich mache. Eine Person äusserte, sie möchte zusehen, wenn das Produkt in der Apotheke abgewogen und abgepackt werde, das gäbe ihr Sicherheit und fände sie für diesen Preis auch gerechtfertigt. Eine andere Person entgegnete, volles Vertrauen in die Apotheken zu haben, für sie sei wichtiger, dass das Produkt vakuumiert und wiederverschliessbar sei. Ein transparentes Säckli fand diese Person nicht zwingend, jedoch hübscher. In Bezug auf die Sichtbarkeit der Produkte kam das Thema auf, wo in der Apotheke Cannabisprodukte präsentiert werden sollen. Denn während der Studie werde Cannabis lediglich für Studienteilnehmende zu erwerben sein. Werden die Produkte sichtbar gemacht, seien sie auch für cannabisinteressierte Personen, die nicht an der Studie teilnehmen sichtbar (2).

Eine Person brachte das Thema Boveda ein. Boveda reguliere die Feuchtigkeit des Produktes im Glas und unterstützen den Reifungsprozess. Darauf entgegnete eine andere Person, dass dies nur funktioniere, wenn das Glas auch regelmässig - einmal pro Tag - geöffnet werde. Auch würde sich Boveda auf den Duft auswirken. Werde Boveda eingesetzt müsse die Blüte gebrochen werden, um den Duft freizusetzen, wie nachfolgendes Zitat untermalt:

> Also bei den Boveda, riechst du es nachher nicht mehr so. Du musst es immer aufbrechen, dass man es riecht. [A: 441]

Ein weiterer Punkt in Bezug auf die Transparenz betraf die Wirkung des Produktes. Gewünscht wären Informationen von Personen, die das Produkt selbst konsumiert haben. Wie nachfolgendes Zitat zeigt:

> Es könnte Testende geben, die Videos machen, wo sie es testen. Das wäre ein 3-Minuten Video. Da sagen sie, es ist das und das. Du siehst sie ein bisschen rauchen. Sie haben sich so und so gefühlt, so und so ist der Taste. Das könnte man in Verbindung mit einem Scan machen. Also auf dem Tisch ein Plakat "Scan me". Und dann kann man dies einscannen und dann kommen zwei, drei Leute, die das testen und Sachen dazu sagen. […] Aber sonst

> würde es mich schon auch wundernehmen, wie ist es wirklich zum Rauchen, wie riecht es, welche Wirkung hat es und wie fühlt sich eine Person dabei? [A: 557]

Daraufhin meinten zwei Stimmen, die Teilnehmenden der aktuellen Begleitgruppe würden sich bestens dafür eigenen Videos zu Cannabisprodukten zu drehen.

3.5 Prävention in der Apotheke

Apotheken haben ein breites Dienstleistungsangebot, darunter fällt auch die Beratung zu Medikamenten, deren Wirkung, Nebenwirkung sowie das Ansprechen von (Sucht-) Verhalten. Die Anwesenden meinten, bei einem ersten Einkauf durchaus offen für ein (Präventions-) Gespräch zu sein (5), dies insbesondere auch für Fachinformationen zu Cannabisprodukten (4). Ein mehrmaliges Präventionsgespräch, so meinten die Anwesenden (4) würde eher nerven und demotivieren, Cannabisprodukte in der Apotheke zu kaufen. Zwei Stimmen würden es begrüssen, wenn Flyer zu Vaporizer, oralen Cannabiskonsum und Rauchstopp als niederschwellige Angebote aufliegen würden. Eine weitere Person nannte die Variante mit den Informationsvideos von Konsumierenden, die mittels QR-Code abgerufen werden können (s. Abschnitt 3.4). Eine Person nahm in Zusammenhang mit dem Präventionsgespräch in Apotheken, die früher in der Diskussion gezeigte Risikoskala auf. Und äusserte folgendes:

> Ich war recht überrascht über diese rot-grüne Tabelle am Anfang. Wie krass die ist. Und wir haben ja vorher alle auf dieses technische Zeug nicht gut reagiert. Ich könnte mir jetzt vorstellen, wenn ein sympathischer Apotheker oder eine sympathische Apothekerin mir sagt: «Wenn sie manchmal die Lunge ein bisschen spüren und sie das Gefühl haben, ...// Dann wäre es so, dass dieses Gerät, es ist zwar ein technisches Gerät, aber es ist schon krass, der Unterschied für Ihre Lunge. Das würde ich Ihnen empfehlen.» […] …das war ja schon noch krass dieser Unterschied. Und wir haben jetzt alle trotzdem, dass wir das gesehen haben, nicht gefunden, doch das müssen wir ausprobiert haben. Aber wenn man mir das gut verkauft, dann wäre ich vielleicht schon noch (zu haben). Weil ich dann denke: "Ok, doch manchmal spüre ich sie auch." Dann würde ich das vielleicht trotzdem mal probieren mit diesem Ding, auch wenn es mir nicht so sympathisch ist. Aber die Gesundheit ist mir ja trotzdem nicht gerade völlig unbedeutend. [A: 548]

Diese Stimme äusserte weiter, dass es der Ton und die Art ausmache, wie die Fachperson mit ihr spreche und das Gespräch führe, was in der Gruppe auf Zustimmung stiess (4).

3.6 Mögliche Verkaufsstellen

Wie bereits in der Einleitung sichtbar gemacht, geht es darum Ideen zu möglichen Verkaufsstellen ausserhalb der Apotheken zu sammeln und zu prüfen. Die Teilnehmenden wurden eingeladen, ihre Ideen kundzutun. Was sie rege taten.

Coffeeshops wie in den Niederlanden oder Cannabis Social Clubs wie in Spanien oder auch Cannabars wurden von allen Anwesenden als passende Möglichkeit genannt (6), wie nachfolgende Aussagen belegen:

> […] aber zum Beispiel gibt es den Kon-Tiki Coffeeshop. Da kann man unten einkaufen und oben rauchen. Sie verkaufen CBD, aber da raucht niemand CBD. Dass es zum Beispiel in solchen Kafis, wo man sowieso rauchen kann, dass man es dort verkaufen könnte. [A:598]

> Es ist so ähnlich wie bei den Social Clubs in Spanien, da musst du einfach Mitglied sein. Sobald du diese Mitgliederkarte hast, hast du nachher geschlossene Räume, da wird an der Theke Hanf verkauft, du kannst Getränke kaufen, es hat Töggelikästen. Ja, wirklich so wie in einem Coffeeshop. [A:608]

> Eben ich gehe zwischen durch sehr gerne nach Amsterdam. Und ich denke dann immer, es ist so sophisticated, […] Ja, genau, es hat so etwas Edles. Ich sitze im Kaffee, ich habe einen feinen Kaffee und nachher habe ich das (Cannabis-)Menu. Gut wir haben dann nur vier auf unserem Menu, aber das ist egal. [A:601]

Gefolgt von CBD-Shops (5), Quartier-Bioladen (4), Tabakladen (4), Four-Twenty-/ Grow-/Headshops (2), direkt ab Bauernhof (2), extra neu gegründete Stiftung zur Cannabis-Abgabe (2) oder eine Weinhandlung. Auf mässiges Interesse stiess die Idee Cannabis zumindest während der Studie online zu kaufen. Lediglich eine Person äusserte, ihre Cannabisprodukte bereits online zu bestellen. Die anderen gaben an, dies unpersönlich, unsympathisch und auch als gefährlich zu erachten. Eine Person meinte, online bestellen und vor Ort abzuholen, wäre eine für sie machbare Variante, bei der auch eine gewisse soziale Kontrolle sichergestellt wäre. Eine weitere Person führte ins Feld, dass bei der online Variante ein Präventionsgespräch wegfallen würde. Und noch eine andere Stimme fand, allein wegen des laufenden Legalisierungsprozesses wäre die Apotheke, der passende Cannabis-Verkaufsort.

Eine Methadon- und Heroin-Abgabestelle wurde von allen Teilnehmenden klar abgewiesen. Dazu hier einige Stimmen, die das unterstreichen:

> Das gibt eine Stigmatisierung und eine Gleichstellung mit Schwerstabhängigen. Und da habe ich das Gefühl, das finde ich das Paradoxe. Hanf ist ein Naturkraut, ...// Klar kann man auch süchtig werden, aber man spricht nicht von einer körperlichen Sucht. […] Aber, dass man Kiffer und all die anderen Konsumenten zwischendrin - Ecstasy, LSD, Kokain und all diese Sachen - dann wird Kiffen automatisch auf die höchste Stufe gestellt, mit Heroin. […] [A: 652]

> Das wäre der falsche Ort, das könnt ihr nicht machen. Veto! [A: 655]
>
> Wir bezahlen ja schon mehr. Wieso sollte ich das wollen? [A: 656]

3.7 Herausforderungen der Forschungsgruppe

Herausforderungen für die Forschungsgruppe wurden von der Gruppe sehr heterogen gesehen. Einzig dieser Input, dass alle Forschenden dieser SCRIPT-Studie, mindestens einmal Erfahrung mit Cannabis gemacht haben sollten, damit sie wissen von was sie sprechen bzw. um den Forschungsansatz zu erweitern, wurde von mehreren Personen gut geheissen (3). Die belegt folgendes Zitat:

> Ich glaube, dass ich es wichtig finden würde, dass alle, die wissenschaftlich an dieser Studie arbeiten, wirklich einmal eins paffen. Damit ihr wisst - ich meine es total ernst und nicht blöd. Und zwar im Sinn von Albert Hoffmann. Wieso hat Albert Hoffmann zum Beispiel das Psilozybin wissenschaftlich nachweisen können? Der Entdecker des LSD, wieso? Weil, alle anderen haben falsch gesucht. Das ist hochinteressant. Ihr müsst das einmal genau, wissenschaftlich nachlesen, wieso Albert Hofmann nicht nur das LSD, sondern auch das Psilozybin in den Pilzen nachweisen konnte. Es gibt in der Wissenschaft der Punkt, bei dem man nicht mehr das alte, wissenschaftliche Denken brauchen kann, sondern man muss wissen, von was man überhaupt spricht. [A: 672]

Von zwei Personen wurde die Platzierung der Cannabisprodukte als Herausforderung gesehen. Die Produkte stehen ausschliesslich für Teilnehmende der Studie zum Kauf frei, wie wird mit anderen Cannabisinteressierten Personen umgegangen, was wird ihnen in Apotheken gesagt? Zwei weitere Stimmen äusserten, dass das veraltete Bild von Cannabiskonsumierenden (Hippies) bzw. von der Cannabispflanze selbst (Einsteigerdroge) in der Bevölkerung, eine Herausforderung sei und die Studie, dies verändern sollte. Eine Person äusserte, die Prävention «durch die Hintertür», könnte bei den Käufer:innen nicht gut angekommen. Da wäre es wichtig, nicht bevormundend oder belehrend aufzutreten. Dazu folgendes Zitat:

> Ob all die Kiffenden für dieses Präventionsding wirklich offen sind? Einerseits will man es zugänglich machen […] Und trotzdem, so ein bisschen Präventionsarbeit durch die Hintertür oder so. Also so, dass man sich vielleicht ein bisschen bevormundet fühlen könnte, also erfahrene Kiffende. [A: 664]

Eine weitere Person merkte an, dass bei diesen Preisen ein Rabattsystem für «Viel-Konsumierende» die Akzeptanz fördern könnte. Und eine letzte Stimme meinte, sie habe einfach Freude, dass es nun bald so weit sei. Das einzige Risiko, sei, dass die Studie plötzlich von der ändernden Gesetzeslage überholt werde (Entkriminalisierung vor Studienende).

3.8 Diverses

Der Cannabiskonsum mit einem Bong wurde diskutiert und das Vorgehen kurz beschrieben. Es kam die Idee auf, diese Art des Konsums könnte gesund sein. Dies wurde jedoch von einer Person, die einige Bongerfahrung hat, vehement dementiert.

Eine Person äusserte, Interesse an Tabakersatz zu haben, also diesen auch in der Apotheke beziehen zu können. Andere Teilnehmende, die keinen Tabak verwenden, nutzen männliche Cannabispflanzen oder auch Blätter der weiblichen. Minze wurde als Tabakersatz genannt sowie auch das Damianakraut, welches auf dem Markt als Tabakersatz angeboten wird.

Ebenfalls geäussert wurde der Wunsch, als Studienteilnehmende der Begleitgruppe ein Degustationspacket der verschiedenen Produkte zu erhalten.

4 Fazit

Im grossen Ganzen wurde die vorgestellte Cannabisprodukteauswahl von den Teilnehmenden positiv beurteilt. Bis auf die e-Liquids, gaben alle Teilnehmenden an, die Produkte zumindest probieren zu wollen. Die anfängliche, absolute Abneigung gegen die e-Liquids, hat sich während der Diskussion etwas gelockert und es äusserten doch vier Personen, diese Art des Cannabiskonsums ausprobieren zu wollen (vgl. Kap. 3.2 und 3.3.3).

Nach einer längeren Diskussion fand die Mehrheit der Gruppe, dass das *Riechen können* am Cannabisprodukt vor dem Kauf, bei der aktuellen Auswahl gar nicht so zentral sei und in einem nächsten Schritt in den Verkaufsstellen aufgenommen werden könne. Hingegen wurde das *Sehen können* der Produkte als wichtig erachtet, auch weil so ersichtlich werde, ob Blätter oder Samen darin enthalten seien, was etwas zur Produktequalität aussagen würde. Da nicht alle Personen, die in der Apotheke arbeiten kiffen, würden kurze Videos zu den einzelnen Produkten begrüsst (z.B. mit QR-Code abrufbar). Auf diesen Videos sollen Konsumierende etwas zum Geschmack und der Wirkung sagen. Diese Aussagen seien glaubwürdiger als von einer Person, die selbst kein Cannabis konsumiert.

In Bezug auf die Präventionsgespräche in den Apotheken waren die Teilnehmenden zurückhaltend. So wurde bspw. geäussert, dass Cannabisprodukte auf der einen Seite für alle in guter Qualität zugänglich gemacht würden und dann doch durch die Hintertür, die Prävention reingeschlichen käme. Ganz anders sieht es bei der Beratung aus, da möchten die Konsumierenden eine fachkundige Person im Verkaufsgespräch.

Sollten Apotheken doch keine Cannabisprodukte, welche zum Genuss konsumiert werden, verkaufen dürfen, nannten die Anwesenden verschiedene andere Verkaufsstellen. Allen voran Coffeeshops/ Cannabars und CBD-Shops. Der online Verkauf stiess auf Widerstand. Dies sei unpersönlich, unsympathisch und auch gefährlich. Es wurde ins Feld geführt, dass eine soziale Kontrolle fehlen würde

und keine Präventionsgespräche geführt werden könnten. Dies wäre jedoch auch bei den anderen hier genannte Verkaufsstellen ein wunder Punkt.

5 Anhang 1: Frageroute Gruppendiskussion

Vorbereitung Begleitgruppe für Cannabiskonsumierende

SCRIPT

4. Juli 2022 / 18:30 – 20:30

<table>
<tr><td>Min</td><td>Thema</td></tr>
<tr><td>15`</td><td>Einstieg, Vorstellungsrunde (kurz, ev. nur Vorname, Wohnort)
Begrüssen, Rahmen klären
Agenda vorstellen (ppp)
Einladen zu einer kurzen Runde «Wer ist wer» (Name, Wohnort, Interesse an Studie)</td></tr>
<tr><td>15`</td><td>Aktueller Stand von SCRIPT (ppp Folien 3-7)
Hintergrund: Weshalb wird Wert auf einen regulierten Erwerb von Cannabis gelegt
Ablauf der Studie und aktueller Stand
Fokus und Forschungsfrage der Studie: «Schadensminimierung» erklären, Risikoskala zeigen

pppFolie 5
→Würdet ihr auch mitmachen, wenn ihr in der Kontrollgruppe landet und 6 Monate mit dem Kauf von Cannabis in Apotheken warten müsstet?
Falls ja, nehmen wir an, es gäbe eine zweite Studie in Bern. Da könntet ihr sofort Cannabis erwerben. Würdet ihr dann aus «unserer» Studie austreten und bei den anderen mitmachen oder würdet ihr blieben und 6 Monate warten?
Welche Gründe gibt es zu warten bzw. zu wechseln?</td></tr>
</table>

<table>
<tr><td>20`</td><td>Angebot Cannabissorten, Geräte (ppp Folien 8-13)
Sorten und Geräte vorstellen und pro Folie (Blüte, Harz, Öl) fragen:
Wie wirkt das auf euch?
Was fällt euch auf?
Was sagt ihr zum Verhältnis von Preis und Produkt?
Würdet ihr diese Produkte kaufen?
Weshalb ist xy wichtig?
E-Liquids (Folie 14)
Könnt ihr euch vorstellen, Cannabis auch zu dampfen (e-Flüssigkeiten, e-Joints) oder zu vaporisieren (Blüten, Harz)?
Was spricht dafür? Was dagegen?
Wenn ihr es nicht schon macht: Unter welchen Umständen würdet ihr dampfen oder e-Liquids konsumieren?
Was könnte für euch ein Grund sein, Cannabis zu dampfen?
Sprechen euch diese Geräte rein optisch an?
Für welches würdet ihr euch entscheiden?
Auf was würdet ihr bei eurem Auswahlverfahren achten?</td></tr>
<tr><td>20`</td><td>Präsentation von Cannabisprodukten in Apotheken (Folien 16-17)
Wie wichtig ist es euch
Cannabis anschauen zu können?
Daran riechen zu können?
Weshalb ist euch das so wichtig? Auf was achtet ihr da genau?
Wann seht ihr vom Kauf von Cannabis ab?
Eigenschaften
Wenn ihr nicht daran riechen / ihn nicht ansehen könnt</td></tr>
<tr><td></td><td>Beschreibung von Cannabisprodukten (Folien 21-26)
Was erwartet ihr von einer Produktebeschreibung?
Wie wirkt diese Beschreibung auf euch?
Was fällt auf?
Was gefällt besonders?
Was fehlt?
Wie sollte der Geschmack beschreiben werden?</td></tr>
</table>

	○ Wie sprecht ihr über den Geschmack? Welche Worte benutzt ihr oder sind unter Cannabiskonsumierenden gängig? Was kennt ihr?<br>○ Wie seid ihr das gewohnt?
20`	***Prävention in Apotheken (ppp Folien 27-28)***<br>In der SCRIPT-Studie ist vorgesehen, dass Apotheker:innen auch präventiv tätig sein werden. Das bedeutet, dass sie mit ihre Cannabis-Kund:innen beraten (z.B. zur Cannabiskonzentration) oder Gespräche führen, sollte ein auffälliger Cannabis- oder Alkoholkonsum auffallen.<br>• Was haltet ihr grundsätzlich von diesem Umstand, dass Prävention angeboten bzw. aktiv angesprochen wird?<br>• Wie weit dürfte die Fachperson mit ihrer Beratung/ ihrem Ansprechen bei dir/ euch gehen?<br>○ Welche Art von Beratung/ Gespräch würdet ihr schätzen?<br>○ Wo würdet ihr eine Grenze ziehen? (im Sinn von zu nahe, geht ihn/sie nichts an, …)<br>○ Welche Konsequenzen würdet ihr ziehen?<br>▪ Ansprechen, Beratung/ Gespräch nicht erwünscht und Cannabis weiterhin in dieser Apotheke kaufen<br>▪ Cannabis nicht mehr in der Apotheke kaufen.
10`	***Mögliche Verkaufsstellen (ppp Folien 25-27)***<br>• Welche Verkaufsstellen seht ihr nebst den Apotheken, um Cannabisprodukte zu verkaufen?<br>○ Wo würdet ihr hingehen bzw. wo würdet ihr garantiert nicht hingehen?<br>○ Weshalb ja/ nein?<br>○ Welche Art Verkaufsstelle käme für euch garantiert nicht in Frage?<br>• Was würde euch ansprechen / entgegenkommen?

15`	***Herausforderungen aus der Perspektive der TN (ppp Folien 28-29)***<br>Jetzt habt ihr viele Informationen erhalten zu Cannabisprodukten, Verpackung, Geräten, möglichen Interventionen in der Apotheke und der Studie selbst.<br>• Gibt es etwas, wo ihr sagt, da müsst ihr hinschauen? Das könnten für Cannabiskonsumierenden Personen heikel sein?<br>• Gibt es etwas, das euch gerade auffällt und ihr uns noch mit auf den Weg geben möchtet?<br>• Wie schätzt ihr das aktuelle Angebot (Produkte, Verpackung, Preis, Geräte, Interventionen) generell ein?<br>o Was entspricht euch?<br>o Was seht ihr eher kritisch?<br>o Was könnte aus eurer Perspektive anders sein? Wie?
5`	***Aussicht (ppp Folien 30-31)***<br>Es ist vorgesehen, eine weitere Begleitgruppensitzung durchzuführen. Dies wird voraussichtlich Sept. oder Okt 2022.<br>Mögliche Themen vorstellen

Laddering Technik:

Vertiefendes Nachfragen mit:

- Weshalb ist dir das wichtig?
- Was bedeutet dies für dich?
- Was verbindest du mit?
- Was würde dir fehlen, wenn xy nicht gegeben wäre? Was würdest du dann vermissen?
- Wie fühlst du dich bei xy?
- Was erwartest du aufgrund von …?

Auswertung der Gruppendiskussion der partizipativen Begleitgruppe von SCRIPT

Die Gruppendiskussion wurde mit Cannabiskonsumierenden Personen geführt

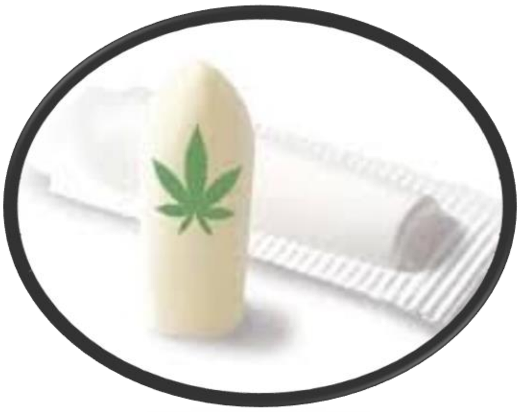

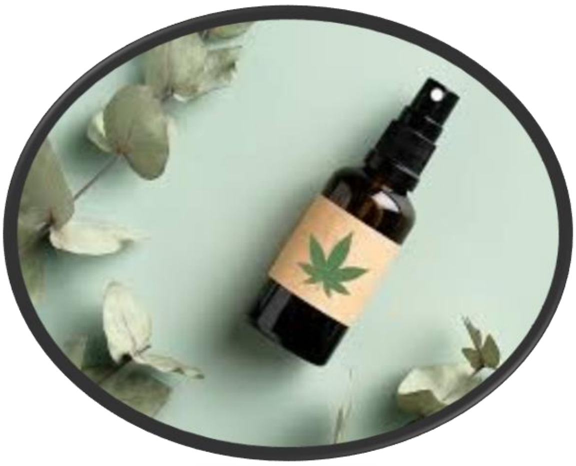

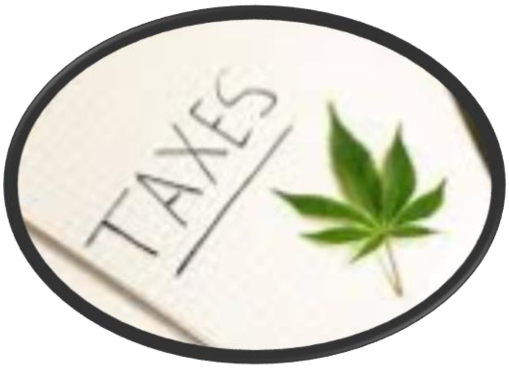

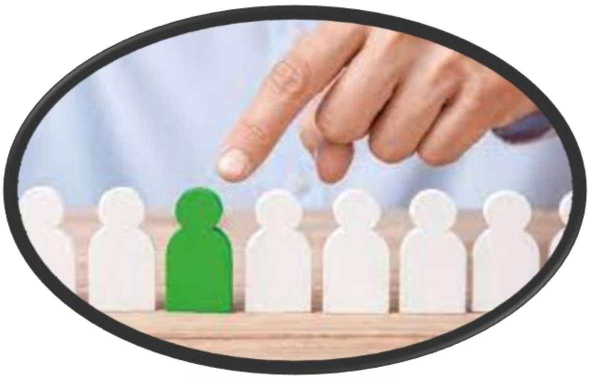

Beatrice Metry

Berner Institut für Hausarztmedizin

März 2023

Inhaltsverzeichnis

1 Einleitung 3
2 Methodisches Vorgehen 4
2.1 Zuständigkeitsabklärung bei der kantonalen Ethikkommission Bern 4
2.2 Erhebungs- und Auswertungsverfahren 4
2.3 Stichprobe 4
3 Ergebnisse aus der Gruppendiskussion 6
3.1 Reaktionen auf weitere Cannabisproduktideen 6
3.2 Gewinnorientierte versus nicht-gewinnorientierter Cannabisverkaufsmarkt 7
3.3 Werbung für Cannabisprodukte 10
3.4 Einsatz der Cannabissteuern 11
3.5 Denkbare Cannabis-Verkaufsstellen 12
3.6 Reaktionen auf die Auswahlkriterien für die Studienteilnehmenden 14
3.7 Ausblick 14
4 Fazit 15
5 Anhang 1: Frageroute Gruppendiskussion 16

1 Einleitung

Im vierten Quartal vom Jahr 2021 wurde mit den neun Mitgliedern der partizipative Begleitgruppe der SCRIPT Studie Einzelinterviews geführt. Im Juli 2022 und im Februar 2023 traf sich die Gruppe je einmal zur Gruppendiskussion in den Räumlichkeiten der Universität Bern. Die Einzelinterviews wie auch diese Gruppendiskussion wurden inhaltsanalytisch ausgewertet und die Resultate in einem Bericht dargestellt.

Bis dato sind folgende Berichte entstanden und erhältlich:

- Auswertung der Einzelinterviews zu SCRIPT vom März 2022
- Auswertung der Gruppendiskussion zu SCRIPT vom Juli 2022
- Auswertung der Gruppendiskussion zu SCRIPT vom März 2023 (vorliegender Bericht)

Die partizipativen Begleitgruppe der SCRIPT-Studie traf sich am 13. Februar 2023 zur zweiten Gruppendiskussion. Von den anfänglich neun Mitglieder der partizipativen Begleitgruppen, waren diesmal fünf cannabiskonsumierende Personen anwesend, die sich unter der Moderation von Beatrice Metry (wissenschaftliche Mitarbeiterin) zu folgenden Themen austauschte:

- weitere Cannabisprodukte (Mundspray, Suppositorien, Crèmes)
- Gewinnorientierter versus nicht-gewinnorientierter Markt
- Cannabis-Verkaufsmarkt-Optionen
- Auswahl der Studienteilnehmenden

Der Auswertungsbericht skizziert die Stichprobe und wird anschliessend den Themen entlang der Frageroute, welche im Anhang zu finden ist, vorgestellt. Im Anhang findet sich die Frageroute.

2 Methodisches Vorgehen

Nachfolgend werden die verschiedenen Teile des Vorgehens beschrieben. Alle befragten Personen haben vor dem Einzelinterinterview (2021) einen Informed consent unterzeichnet und sich durch ihre Unterschrift mit dem Interview sowie den nachfolgenden Gruppendiskussion und deren Auswertung einverstanden erklärt. Darin wurde ihnen Anonymität zugesichert.

2.1 Zuständigkeitsabklärung bei der kantonalen Ethikkommission Bern

Am 31. Mai 2021 wurde das Konzept zur qualitativen Begleitforschung mit dem Titel «Einsatz einer partizipativen Begleitgruppe bestehend aus Cannabiskonsumierenden Erwachsenen als Ergänzung während der Planungsphase des Projekts SCRIPT 2» bei der Kantonalen Ethikkommission (KEK) eingereicht, um deren Zuständigkeit zu prüfen. Die KEK befand, dass sie nicht zuständig sei. Dies bedeutet, dass diese Forschung nicht unter das Humangesetz Artikel 2, Absatz 1 fällt und Einzel- und Gruppeninterviews mit der ausgewählten Zielgruppe durchgeführt werden können, ohne ein Gesuch einzureichen. Nach diesem Bescheid[1] vom 20. Juni 2021 wurde mit der Akquise von Teilnehmenden gestartet.

2.2 Erhebungs- und Auswertungsverfahren

Die Daten wurden durch eine Gruppendiskussion entlang einer definierten Frageroute, während einem Zeitraum von zwei Stunden, durchgeführt und digital aufgezeichnet sowie anschliessend transkribiert.

Das Transkript wurden mittels der Software MAXQDA inhaltsanalytische ausgewertet. Das bedeutet in einem ersten Schritt die Codierung des Textes, anschliessend eine Verdichtung der einzelnen Aussagen sowie das Verschriftlichen der Ergebnisse.

2.3 Stichprobe

Für die Gruppendiskussion wurden alle neun Personen, welche bereits bei den Einzelinterviews mitgemacht und den entsprechenden Informed consent unterzeichnet haben, angefragt. Via doodle wurde der Termin vereinbart.

Ein Gruppenmitglied hat sich abgemeldet, da die Person für ein Jahr im Ausland studiert und deshalb nicht an den Sitzungen teilnehmen kann. Zwei Personen haben sich kurzfristig abgemeldet und eine Person hat sich im Datum geirrt und kam deshalb nicht. Dies führte dazu, dass an dieser Gruppendiskussion fünf cannabiskonsumierende Personen plus die Moderatorin anwesend waren. Die nachfolgende Tabelle gibt eine Übersicht der Stichprobe.

[1] BASEC-Nr Req-2021-00609, Bescheid der Zuständigkeitsabklärung liegt vom 20.6.2021 vor

	**Einzelinterviews 4. Quartal 2021**	**Gruppendiskussion 1 Juli 2022**	**Gruppendiskussion 2 März 2023**
**Anzahl Personen**	9	6	5
**Jahrgang**	1957, 1975, 1990, 1991, 1993, 1999, 2000, 2001, 2000	1957, 1975, 1991, 1993, 2001, 2002	1957, 1991, 1993, 2000, 2002
**Branche Beruf**	Studium (3)<br>Logistik (2)<br>Finanzen (1)<br>Fotografie (1)<br>Grafik (1)<br>Fitness (1)	Studium (1)<br>Logistik (1)<br>Finanzen (1)<br>Fotografie (1)<br>Grafik (1)<br>Fitness (1)	Studium (2)<br>Finanzen (1)<br>Grafik (1)<br>Fitness (1)
**Geschlecht**	weiblich 3<br>männlich 5<br>non-binär 1	weiblich 2<br>männlich 3<br>non-binär 1	weiblich 2<br>männlich 3<br>non-binär 0
**Nationalität**	Schweiz 9	Schweiz 6	Schweiz 5

3 Ergebnisse aus der Gruppendiskussion

Die Ergebnisse werden nachfolgend entlang der Frageroute aufgeführt. Zahlen in runden Klammern weisen darauf hin, wie viele der Befragten in diese Richtung geantwortet haben. Befindet sich keine Klammer hinter der Aussage, handelt es sich um eine Einzelnennung. In eckigen Klammern wird der Absatz in der MAXQDA-Datei (A), in welchem das genannte Zitat gefunden werden kann, aufgeführt.

3.1 Reaktionen auf weitere Cannabisproduktideen

Der Gruppe wurden Suppositorien, Mundspray und (Schleim-)Hautcrème als weitere Cannabis-Produktideen vorgestellt. Drei Personen äusserten, erst Erstaunen über diese Ideen und meinten anschliessend ihnen würde bei diesen Produkten die Sinnlichkeit des Drehens und Rauchens fehlen. Weitere erste Reaktionen waren: die schnelle Aufnahme der Wirkstoffe über die Schleimhäute; die potentere Wirkung als beim Rauchen; weniger Nebenwirkungen, da keine Rauchabfallprodukte eingeatmet werden. Eine weitere Stimme äusserte, dass mit Mundspray, Zäpfchen und Crèmes der Cannabiskonsum verdeckt bleibe. Die letzte Aussage wird mit nachfolgendem Zitat unterlegt:

> Ich habe das Gefühl, mit diesen Rauschkonsumformen, kann man sehr inkognito unterwegs sein. Wenn du kiffst, dann riechst du den Rauch. Es ist auffällig. Und sonst habe ich das Gefühl, du kannst sprayen oder nimmst ein Zäpfchen und da schaut niemand hin. Es ist etwas, wo ich das Gefühl habe, man kann es besser versteckt halten. [A:58]

Die Teilnehmenden waren sich einig, einen Cannabis-Mundspray würden sie ausprobieren (5). Der Mundspray fand Anklang, weil dieser effektiv und einfach zu dosieren sei (4), wie nachfolgendes Zitat belegt:

> Weil beim LSD finde ich den Spray genial. Also das effektive Dosieren. 20/40 oder wie viel. Einfach, dass du es präzis dosieren kannst. Und daher, heute wo dieses Gras einfach so stark ist und die Dosierung wirklich heikel, finde ich das ein Problemthema. [A:32]

Zur Dosierung des Mundsprays gaben zwei Personen an, eine Dosierung würde ausreichen. Der Konsum könne gut mit der Anzahl Stösse reguliert werden. Eine weitere Person gab zu bedenken, dass auch bei einem Cannabis-Mundspray jede Person anders auf die Cannabiswirkstoffe reagiere und dies beim Konsum berücksichtigt werden müsse.

Für eine Person ist klar, dass Mundspray geruchlos sei. Eine andere äusserte, falls dieses Produkt geruchlos sei, dies ein guter Reisebegleiter sein könnte.

> Weil, es würde natürlich ein gewisses Problem, das man beim Reisen hat, lösen. Wenn man etwas hat, das absolut nicht riecht. Dann könnte dies der Begleiter auf Reisen sein. [A:63]

Dagegen äusserte eine andere Stimme, Schmuggeln sei nicht die Idee von neuen Cannabisprodukten. Die (Schleim-)Hautcrèmes würden vier Personen ausprobieren. Eine Stimme äusserte Interesse an einer Nasensalbe zu haben, dies als weitere Produkteidee.

Suppositorien als Verabreichungsform von Cannabis gab in der Gruppe zu reden. Auf der einen Seite äusserten mehrere Personen, Zäpfchen seien mit Kranksein konnotiert (2) bzw. gehöre nicht zum Genusskonsum. Die belegen nachfolgende Zitate:

> Ja. Zäpfchen ist für mich sehr negativ mit Kranksein konnotiert. [A:42]
>
> Die Zäpfchen sind für mich unnötig, eigentlich. Also ich sehe da nur den medizinischen Hintergrund. Für den Genusskonsum ist es nicht praktisch, wenn ich das so sagen kann. [A:45]
>
> Ich habe mir jetzt die Kifferrunde vorgestellt. Statt einen Joint rundumreichen, gibt man sich gegenseitig ein Zäpfchen. [A:24]

Generell zu diesen Produktideen, äusserten die Teilnehmenden (3), diese würden eine neue Zielgruppe ansprechen. Genannt wurden Menschen, die bisher nicht geraucht bzw. gekifft hätten oder auf eine gesündere Konsumform umsteigen möchten. Nachfolgende Aussage untermalt dies:

> Aber ich denke auch, für jemanden, der es nicht kennt und es gerne einmal probieren möchte, und seine Lunge nicht schädigen will, weil er noch nie geraucht hat, dann ist so ein Spray...// Also, dass diese Person eher auf ein Spray oder ein Zäpfchen oder keine Ahnung auf was ansprechen würde. Aber jetzt halt der 0815-Kiffer ist halt einfach, wie schon erwähnt, wir sind gerne mit unseren Joints und unserem Drehen und alle dem. [A:27]

Eine der befragten Personen äusserte, sie fände es an der Zeit mit der Umsetzung der Studie zu beginnen bevor weitere Konsumformen ausgearbeitet würden. Diese Person formulierte ihre Aussage wie folgt:

> Nach jetzt sechs Jahren darüber diskutieren, ob man jetzt darf oder nicht. Und man legt extra einen Gesetzesartikel mit Experimentierartikel fest […] habe ich das Gefühl, ich würde das jetzt lieber mal Rauchen oder Vaporisieren statt x-verschiedene Konsumvariationen zu haben. [A:24]

Es gibt auch kritische Bemerkungen zu den weiteren Darreichungsformen. So äusserte eine Person Bedenken, da Mundspray durch die diskrete Einnahme und steten Verfügbarkeit zu vermehrtem Konsum einladen könne, wie folgendes Zitat aufzeigt:

> Aber das kann auch nach hinten losgehen. Weil, wenn du einen Spray dabeihast, der dich immer anlacht, wo du immer schnell hervornehmen kannst und sprayen ohne, dass irgendjemand etwas mitbekommt. Das kann auch in die andere Richtung laufen. [A:37]

Eine weitere Stimme geht in die ähnliche Richtung und fügt an, dass die oben genannten Eigenschaften der Produkte zu einem versteckten Suchtverhalten führen könnten.

> Ich habe das Gefühl, […] mit diesen Rauschkonsumformen […] kann man sehr inkognito unterwegs sein. Wenn du kiffst, dann riechst du den Rauch. Es ist auffällig. Und sonst habe ich das Gefühl, du kannst sprayen oder nimmst ein Zäpfchen und da schaut niemand hin. [A:58]

3.2 Gewinnorientierte versus nicht-gewinnorientierter Cannabisverkaufsmarkt

Die Teilnehmenden wurden gefragt, welche Art von Verkaufsmarkt sie für Cannabisprodukte generell sehen würden. Vorgestellt wurden der gewinnorientierte und der nicht-gewinnorientierte Markt. Der Grundtenor der Anwesenden tönte gegen den Kapitalismus für den Cannabismarkt. So folgen hier einige Zitate, die dies belegen:

> Überall wo Goldgräberstimmung herrscht, da ist es plötzlich so, dass man nur noch das Positive sieht. Vielleicht auch die Gefahren, die damit einhergehen, denn Risiken gibt es überall. Ja, eben, dass man die Kuh melkt, bis sie stirbt. […] Für mich persönlich steht Hanf für etwas anderes. Es steht für Entschleunigung. Und nicht für das Karussell dreht immer schneller und der Stress nimmt zu. [A:124]
> Ja, eine von den grössten Ängsten, eigentlich, dass es immer nur darum geht, dass mehr Geld damit gemacht wird. Und das alles teurer wird. […] Und, ja, dieses Gewinnorientierte, dass man mit etwas, für mich Schönem, nachher das Schlechte daraus genommen wird. [A:85]

> Auch wenn ich das uncool finde, dass Cannabis so kommerziell ausgestattet wird, denn ich finde, es entspricht nicht dem Naturell der Pflanze. Es ist ein Unkraut, das überall wächst und dafür nachher Geld oder viel Geld zu verlangen, finde ich moralisch, ja, das schneidet sich. [A:89]

> Es wird immer Leute geben, die aus irgendetwas einen Profit schlagen werden. Das kann man nicht abstreiten. Und es war auch so, als man das CBD legalisiert hat. [A:91]

Dagegen hält eine Stimme folgendes:

> Auch wenn der ganze Kapitalismus und alles Scheisse ist, Fakt ist, wenn es Gewinn gibt, dann ist Akzeptanz da. Und das Ziel ist schlussendlich, dass es [das Cannabis] akzeptiert wird. [A:92]

Eine weitere Stimme äusserte, wo Gewinne entstünden, würden sich alle freuen und dies schaffe Akzeptanz in der Gesellschaft. Diese Akzeptanz (3) wurde als Vorteil des gewinnorientierten Marktes gesehen. Auch der offene Umgang zum Beispiel mit Cannabiswerbung schaffe ein anderes Bild und führe zu Akzeptanz in der Gesellschaft.

Eine weitere Person sprach die Konkurrenz auf dem Markt als positiven Effekt an. Denn so würde auch der Schwarzmarkt unter Druck geraten und das fanden mehrere Anwesende ein gutes Argument (3), das für den gewinnorientierten Markt spreche. Nachfolgend zwei Zitate dazu:

> Zu dem sehe ich auch bei der Variante 1 [gewinnorientierter Markt] den ganzen Schwarzmarkt, der eher zusammenbricht, wenn man das so macht. [A:92]

> Auf der einen Seite würde ein kapitalistischer Markt sicher den Preis drücken. Weil einfach ein Konkurrenzdenken ist, sage ich jetzt einmal. [A:89]

Ein weiterer Vorteil des gewinnorientierten Marktes sah eine anwesende Person in der Schaffung von Arbeitsplätzen in unterschiedlichen Gebieten (Anbau, Produktion, Werbung, Verkauf). Diese Arbeitsplätze seien für unsere Gesellschaft wichtig.

Der nicht-gewinnorientierte Ansatz, so äusserten zwei Personen, sei eher für medizinische Produkte sinnvoll. Wenn es um Genuss gehe, «sollte man voll auffahren und richtig dran gehen» [A:127], so eine Stimme.

Als klarer Nachteil im nicht-gewinnorientierten Markt wurden die regulierten Preise genannt (4). Da die Preise staatlich vorgegeben seien, wären sie überteuert und der Schwarzmarkt könne weiterblühen. Eine Person äusserte, dass regulierter Markt nur teilweise die Wünsche der Konsumierenden decken würden, dies formulierte sie wie folgt:

> Ich denke, wenn man es streng reguliert machen will, dann ist es so das Prinzip vom kleinen Finger geben und man nimmt die Hand. Weil es einfach ein bisschen erlaubt wird, aber nicht in diesem Ausmass wie es gewünscht wird. Das ist vielleicht noch ein kritischer Punkt. Ich glaube schon, dass es ein grosser Schritt ist, aber man muss sich bewusst dazu entscheiden, den grossen Schritt zu nehmen und nicht den kleinen. [A:182]

Als Vorteil im nicht-gewinnorientierten Markt wurde die Berücksichtigung von sozialen Institutionen und Vorhaben gesehen (2). Zwei Personen gaben an davon überzeugt zu sein, dass es in der Schweiz ein regulierter Markt wird, da auch der Tabakmarkt stark reguliert sei.

Die Anwesenden äussern, dass wohl eine Mischform der beiden Marktansätze für die Zukunft denkbar wäre. So sollte unbedingt eine Abgabe für Prävention, Suchbehandlung oder psychiatrische Therapien (3) vorgesehen werden.

> Und ich habe das Gefühl, so eine Mischform, wäre etwas Cooles. Auch wenn es kapitalistisch wäre, könnte man sagen, 10 oder 20% vom Umsatz gehen in die Suchtprävention rein. [A:89]

> Ich finde auch, eine Mischform, die einzige gute Lösung ist. [A:91

> Für mich ist es jetzt noch spannend, weil ich recht anti-Werbung bin. Und das war nun recht spannend, diesen Ansatz einmal zu hören. Und ja, es macht definitiv Sinn, dass wir anders denken. Ich sehe jetzt auch die Nachteile im nicht-gewinnorientierten. Aber ich finde, man sollte den richtigen Weg aus beidem finden. Weil, ich nicht sagen kann fix dies oder das. Es ist sicher ein Thema, das noch ein bisschen länger geht, wo ich das Gefühl habe, dass man da noch den richtigen Weg finden muss. [A:147]

Eine weitere Person äussert, sie finde den Begriff «gewinnorientiert» falsch, es sollte eine *Marktorientierung* verfolgt werden, da eines der Ziele sei, den Schwarzmarkt zu eliminieren. Diese Person führt ihre Gedanken wie folgt aus:

> Ich würde es nicht so formulieren "gewinnorientiert", ich würde sagen: "marktorientiert". Weil eines der Ziele ist der Schwarzmarkt zu eliminieren. Das heisst, wenn ich Unternehmer bin und jetzt Cannabis produziere, dann kann ich nicht einfach sagen, ich will jetzt einfach viel verdienen und das ist jetzt teuer. Sondern, ich muss den Schwarzmarkt wegbringen. Das heisst, ich muss phasenweise sehr weitrunter mit dem Preis, damit dies gelingt. Es ist ja so, als würde die eine Gangsterbande gegen die andere kämpfen. […] So, also in diesem Sinn wäre es für mich nicht gewinnorientiert, sondern der Marktansatz. Wie der Markt funktioniert. Und in diesem Sinn, kann ich nicht sagen, es setzt sich aus dem, dem und dem zusammen und das gibt den Preis. Sondern, der Preis ist allein definiert von der Frage: Wie bringen wir den Schwarzmarkt weg? Das habe ich das Gefühl, müsste der Ansatz sein, weil das ja ein Ziel ist. [A:136]

Nachfolgend werden generelle Aussagen zum zukünftigen Cannabismarkt in der Schweiz dargestellt.

> Ich habe das Gefühl, es ist wohl wie eine Evolution, schlussendlich. Mit der Regulierung und der Entkriminalisierung wird es einmal zur Legalisierung kommen. Aber auch da, die Schweiz ist konservativ. Da geht noch viel Wasser die Aare runter, bis wir den ersten Joint rauchen mit diesem legalen Bio-Hanf.

Es wurde das Anliegen einer Lösung auf Bundesebene geäussert, um dem Cannabistourismus zwischen den Kantonen vorzubeugen.

Ebenfalls wurde der Eigenanbau angesprochen. Dieser sollte bei einer Marktöffnung für den Eigengebrauch erlaubt werden (4). Dies mit nachfolgender Begründung:

> Die älteste Ausgrabung in der Schweiz ist bei Egolzwil, bei Wauwil. Dort haben sie Hanfsamen gefunden. Also, die ältesten Sachen in unserem Gebiet sind Hanfsamen. Also, das ist wirklich ein uraltes Kulturgut von hier. Das finde ich wichtig. [A:97]

3.3 Werbung für Cannabisprodukte

Die Anwesenden wurden dazu befragt, ob Cannabisprodukte beworben werden sollen. Dazu äusserten sich drei Personen eher kritisch. So befürchtet eine Person, eine verstärkte Ächtung von Cannabiskonsumierenden in der Gesellschaft. Sie äusserte dies wie folgt:

> Dass sich nachher alle darüber aufregen können. So: Ja, jetzt machen sie noch Werbung dafür. Das kann ja nur schlimmer kommen. [A:85]

Eine andere Person merkte an grundsätzlich soll keine Werbung für Cannabisprodukte gemacht werden, wie nachfolgendes Zitat zeigt:

> Ich finde nicht, dass man Werbung für Cannabis machen sollte. Ich finde auch nicht, dass man für Alkohol Werbung machen sollte. [A:198]

Und die dritte Person äusserte Kiffen sei eine Lebenseinstellung und die könne nicht beworben werden. Ebenso schneide allfällige Cannabiswerbung die aktuellen Auflagen der Tabak- und Alkoholwerbung.

> Ich glaube, für viele ist es nur ein Ausprobieren. Aber Kiffen ist eine Lebenseinstellung. Eben, ich bin auch kritisch eingestellt gegenüber der allgemeinen Werbung. Weil ich das Gefühl habe, es schneidet sich mit dem Alkohol und dem Tabak. [A:188]

Des Weiteren lobte eine dieser drei Personen die mundzumund-Propaganda als gut funktionierendes Werbemittel, wie nachfolgendes Zitat gut unterlegt:

> Aber ich finde schlussendlich schon mundzumund-Propaganda eine gute Werbung und eine die richtig gut funktioniert. [A:202]

Eine weitere Person gab an, sie fände es sinnvoll, wenn Cannabiswerbung denselben Vorschriften unterliege wie die Alkohol- und Tabakwerbung. Und noch eine weitere Stimme fand, gerade das Angebot von sauberem Cannabis, sollte unbedingt beworben werden, wie nachfolgendes Zitat gut zeigt.

> Also das fände ich, dass man dies unbedingt bewerben sollte. […] Also, wenn du nachher einem halbwegs anständigen Kiffer sagst, was da auf dem Schwarzmarkt passiert, und das willst du doch nicht. Bezieh es doch anders. Die Leute kaufen Havelaar Produkte. Argumente funktionieren. Viele Leute hören auf Argumente. Nicht alle, aber viele. Und in diesem Sinn zu sagen, was die Konsequenz ist, wenn du es auf dem Schwarzmarkt kaufst. [A:207]

Dieselbe Person erläuterte, dass Zigarettenwerbung ebenfalls darauf abziele, dass die Konsumenten die Marke wechselten und es nicht darum gehe, Nichtraucher:innen zu Raucher:innen zu machen.

3.4 Einsatz der Cannabissteuern

Dass über kurz oder lang auf Cannabisprodukten eine Steuer erhoben werde, war allen Anwesenden klar. Die Cannabissteuer wurde mit der Tabak- und der Alkoholsteuer in Zusammenhang gesetzt. Dabei äusserten zwei Personen, dass die Steuer auf allen drei politischen Ebenen (Gemeinde, Kanton, Bund) verteilt werden soll. Eine Stimme bevorzugte die Gemeinde, da diese auch die Infrastruktur genehmige und dort die Wirkung der Steuer am besten sichtbar werden. Der Einsatz der Steuergelder, wurde von den Anwesenden in der Suchtprävention, Jugendarbeit, sozialen Institutionen und der Notfallpsychiatrie gesehen. Nachfolgendes Zitat gibt darüber Aufschluss, wie das aussehen könnte:

> Also, jetzt einmal so aus der Hüfte geschossen, wenn man sagt, 20% der Einnahmen werden als Steuer abgegeben. Da könnte man eine Aufteilung machen. Ein Viertel von diesen 20% geht in die Prävention, ein Viertel geht in die Notfall-Psychiatrie oder sonst

> Psychiatrie, ein Viertel geht in die AHV und ein Viertel geht in die restlichen Sozialwerke. Und dann könnte man schauen, wie wird es verwendet. Wenn man merkt, in der Psychiatrie braucht es nicht viel, nur ein Bruchteil, dann könnte man das dann auch verschieben. [A:265]

3.5 Denkbare Cannabis-Verkaufsstellen

Die Ideen für weitere Verkaufsstellen – nebst den Apotheken – sind in dieser Runde vielfältig. Was deutlich hervorkam ist, eine Stiftung, die mit harten Drogen oder der Methadonabgabe in Verbindung gebracht wird, geht für die Anwesenden gar nicht. Das bedeutet auf dem Markt Bern, dass die Stiftung Contact für den Verkauf von Cannabisprodukten von Cannabiskonsumierenden nicht akzeptiert würde, was nachfolgendes Zitat unterstreicht:

> Also ich finde alles, was [die] Herkunft hat von harten Drogen, finde ich ein absolutes No-Go! Weil, diese Scheissbehauptung, es sei eine Einsteigerdroge, einfach nährt. Und da ist wirklich null Toleranz. Einfach nicht. [A:214]

> Ja, genau. Denn das ist eine der ganz grossen Lügen und die müssen wir nicht nähren. Also wirklich nicht. [A:216]

Das Cannabisangebot würde gut in die Bio-Läden passen, fanden die Anwesenden (5). Diese seien in die Quartieren, also Nahe der Kundschaft und den Leuten vertraut, das wurde als Vorteil gesehen. Die Bio-Läden hätten oft pro Abteilung spezialisiertes Personal. Das könnte für die Cannabisecke, genauso sein und würde bedeuten, dass eine Person eine zwei - dreitägige Schulung machen würde. Dafür kämen auch Quereinsteiger ohne Eidgenössischen Fähigkeitszeugnis (EFZ) in Frage. Die folgenden Zitate belegen, diese Aussagen:

> Dann könnte es auch sein, dass wenn jetzt irgendjemand hört oder im Haller-Laden ein Plakätchen ist "Cannabis kann hier gekauft werden" oder so etwas. Dann denkt man: "Aha, hier kann man das beziehen". Das liegt aber nicht einfach beim Gemüse und man kann es in den Plastiksack packen und wägen. Das müsste beim Käsestand oder so sein, wo man sagen kann: "Ich würde gerne ein bisschen Gras kaufen". Und dann wäre die erste Frage: "Hast du bei uns schon einmal gekauft?" Und wenn ja, dann: "Klar, von welchem, wie viel?" Und wenn es das erste Mal ist, dann gibt es ein kurzes Einführungsgespräch. Was man (im Sortiment) hat. [A:235]

> Und vielleicht so zwei oder drei Tage hast. Zum Beispiel, wie wenn du Autofahren lernst. Da machst du den Nothelfer. Da gehst du zwei Tage hin, hast ein paar Stunden Inputs, füllst am Schluss etwas aus und dann ist gut. Und ich finde, dann wäre es nicht einmal wichtig, dass man einen EFZ hat, sondern einfach diese Schulung. Dann macht man diese Schulung, die geht zwei Tage und dann passt das für mich. Vom Stoff her reichen

> zwei Tage, das ist durchaus möglich, eine Person aufzuklären, dass er andere Menschen aufklären kann. [A:316]
>
> Da kommen auch Leute, die eine Ahnung davon haben. Es muss niemand sein, der eine EFZ-Ausbildung hat. Eben, ich kenne Leute, die weiss wie viel Ahnung vom Kiffen haben und vom Anbau bis zu weiss nicht was, aber sonst keine Ausbildung. Und ich meine, ja, wenn du nachher noch ein bisschen mit Leuten umgehen kannst...// Schau an einem anderen Ort und mach eine Ausschreibung so: "Hey, bist du extrovertiert und kannst mit Menschen umgehen und hast eine Ahnung von Cannabis?" Aber, dass man auch Arbeitsplätze schaffen kann für Menschen, die an einem anderen Ort keine Chance hätten. Aber dort blühen sie voll auf. Ja, klar, sie müssen noch so einen Tag in den Unterricht und dann wissen sie auch, was ist gut, was ist nicht gut. Aber eben, auch einmal anderen Leuten eine Chance geben. Auch Quereinsteiger. [A:329]

Weitere Informationen könnten mit einem Aufsteller (Broschüren, Touchscreen oder QR-Code) den Kunden und Kundinnen angeboten werden, ergänzte eine weitere Person, das Bio-Laden-Angebot.

Zustimmung fand auch die Selbstvermarktung der Cannabisprodukte durch die Bio-Bauern selbst. Eine Idee, welche unter den Anwesenden Anklang fand, war der 1.Augst-Brunch, bei welchem das Cannabisangebot des Bauernbetriebes vorgestellt werden dürfe (3). In gleichem Mass Zustimmung fand die Idee, das Angebot in den Head bzw. Growshops auszubauen (3). Da seien die Fachpersonen bereits am Werk und Zubehör könne gekauft werden. Auch die vorgestellten Cannabisvereine (analog den Social Clubs in Spanien) erhielten ein positives Echo (3). Nachfolgendes Zitat verdeutlicht die Idee, dieser lokalen Cannabisvereine:

> Ja, das geht so ein bisschen in diese Richtung. Aus der Region für die Region. Dass ein Club besteht, in dem Leute aktiv Mitglied sind und auch Leute dazu holen können. Und die können dann zum Eigenpreis ihr Cannabis kaufen. Dann findet alle drei Monate eine Ernte statt und dann hat man 1 Gramm für 3-4 Franken. Und das ist schlussendlich auch eine billige Variante. Ich habe das Gefühl, die Leute werden nicht gerne bevormundet, gerade mit Rauschmittel. [A:140]

Vereinsstatuten würden regeln, wer wie viel Cannabis erwerben darf. Eine aktive Mitgliedschaft wäre eine Pflicht. Weitere Ideen waren Tabakläden und Kioske in den Cannabisverkauf einzubinden. Oder Cannabisbars zu eröffnen, in denen Cannabis gekauft und konsumiert werden könnte. Cannabisbars als Verkaufsmarkt einzusetzen hätte den Vorteil, dass sie abends und an den Wochenenden geöffnet sind.

Ein Bedürfnis der Anwesenden war Cannabisrauchzonen (3) festzulegen. Da sollte das Rauchen von Cannabisprodukten möglich sein. Vergleichbar mit den Raucherzonen an den Flughäfen oder Bahnhöfen.

3.6 Reaktionen auf die Auswahlkriterien für die Studienteilnehmenden

Bezüglich der Auswahlkriterien, die definieren, wer an der Studie teilnehmen darf, kamen wenige Reaktionen auf. Eine Person meinte, die Zahlen 55% männlich, 40% weiblich und 5% andere, sei eine unfaire Verteilung, da mehr Männer als Frauen berücksichtigt würden. Eine weitere Person meinte, das sei ok, wenn diese Zahlen das Verhältnis der kiffenden Personen in unserer Gesellschaft abbilde. Und eine dritte Person äusserte, die Parameter würden nüchtern und verständlich wirken, das sei ok.

Mehr als die Parameter interessierte die Teilnehmenden, ob sie sicher an der Umsetzungsstudie teilnehmen könnten, den dies sei ihnen zugesichert worden, als sie sich für das Engagement in der partizipativen Begleitgruppe angemeldet hätten. Den Teilnehmenden wurde von der Moderatorin zugesichert, dass dies so sei, und sie an der Umsetzungsstudie zugelassen würden.

3.7 Ausblick

Eine nächste Begleitgruppensitzung wird für den Mai 2023 vorgesehen. In der Zeit dazwischen könnte es sein, dass die Teilnehmenden etwas zum Lesen und Rückmelden erhalten würden. Die Frage dazu war, ob sie dazu bereit wären. Da stimmten alle fünf Personen zu. Ebenfalls würden alle fünf Personen über einen Rechner verfügen, an welchem sie die Dokumente lesen und direkt Notizen hineinschreiben könnten. Ein Ausdruck der Dokumente wünscht niemand der Anwesenden.

4 Fazit

Die Teilnehmenden dieser Begleitgruppensitzung äusserten Neugierde bezüglich der weiteren Cannabisproduktideen. Besonders der Mundspray und Crèmes für Schleimhäute fänden die Anwesenden interessant. Sie gaben an, zu bedenken, dass diese Konsumform im Verstecken stattfinden könne, anders als das Rauchen und somit ein Suchtverhalten weniger augenfällig werden.

In Bezug auf die Marktform nannten die Befragen einige positive Aspekte des gewinnorientierten Marktes, so sei dieser effektiver, wenn es darum gehe, den Schwarzmarkt zu schwächen und gesellschaftliche Akzeptanz des Cannabiskonsums zu erlangen. Eine Mischform wäre realistisch. Dabei sollten 10-20% des Umsatzes für Soziales (Notfall-Psychiatrie, Suchtprävention, etc.) abgegeben werden. Die beste Werbung für Cannabisprodukte sei die mundzumund Propaganda. Weitere Werbung sei eher unrealistisch, da Tabak und Alkohol auch nicht beworben werden dürften.

Als weitere Cannabisverkaufsstelle wurde der Bio-Laden im Quartier hervorgehoben. Vorstellbar seien auch Head- bzw. Growshops, Cannabisbars, Tabakläden und Kioske. Was für die Teilnehmenden als Verkaufsstelle gar nicht geht, sind Stiftungen oder Stellen, welche mit harten Drogen in Verbindung gebracht werden.

Die nächste Begleitgruppensitzung ist für den Mai 2023 vorgesehen. In der zwischenzeit sind die Teilnehmenden bereit Informationsschreiben, Flyer etc. zu lesen und dazu eine schriftliche Rückmeldung zu geben. Alle Anwesenden verzichten auf einen Ausdruck per Post, sie würden die Unterlagen elektronisch lesen und bearbeiten.

.

5 Anhang 1: Frageroute Gruppendiskussion

Vorbereitung Begleitgruppe für Cannabiskonsumierende

SCRIPT

6. Februar 2023 / 18:30 – 20:30

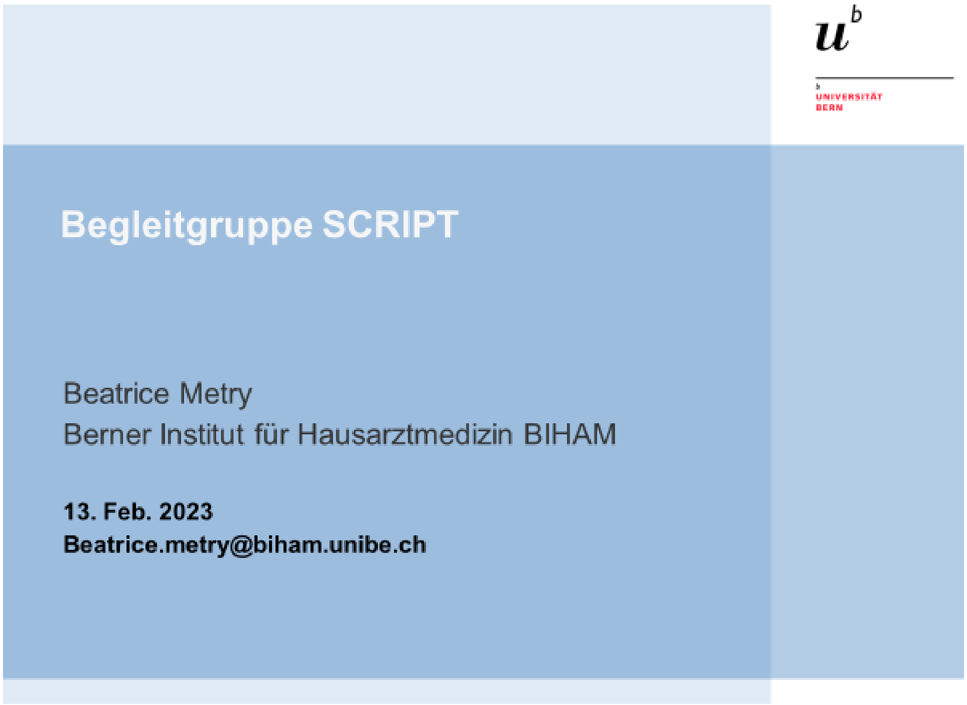

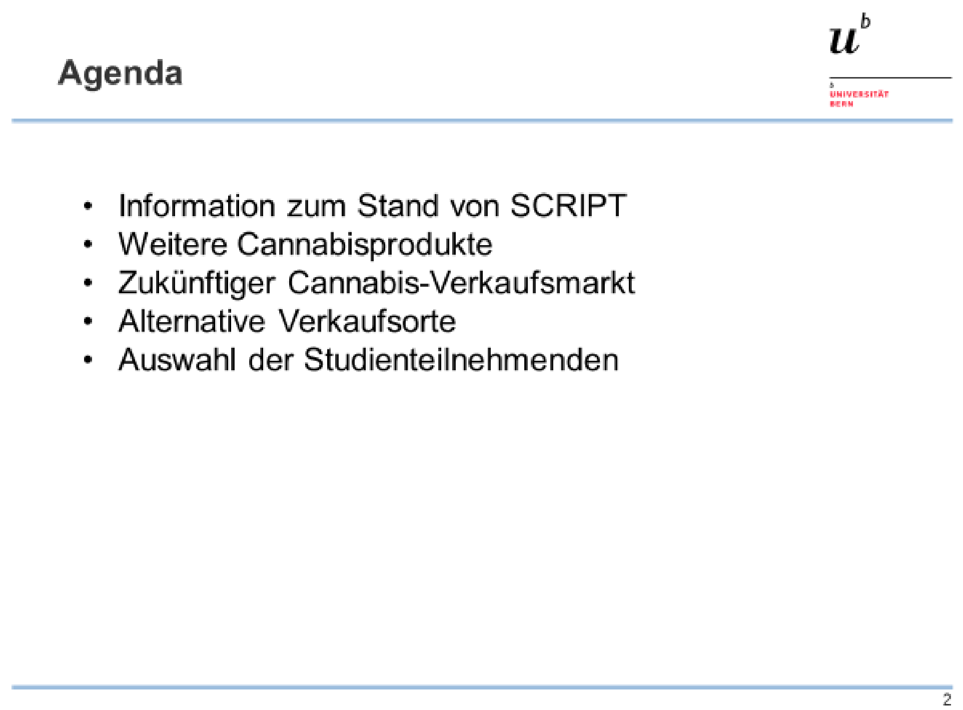

Informationen zu SCRIPT

Der Bewilligungsprozess ist am Laufen
- Der Kanton Bern unterstützt Studie nicht und lehnt Abgabe Cannabis grundsätzlich ab.
  - Genügend Studien um Entscheid treffen
  - negatives Signal für die Jugendlichen

Gespräch mit Kanton wird gesucht
Voraussichtlicher Beginn in Bern im Spätsommer 2023
Bewilligungsprozess Versuche in den Kantonen Zürich, Genf und Waadt laufen.

Cannabisproduzent kann erst ab April aussäen, die Ernte wird im Spätsommer 2023 erwartet.
  - Gras wird von Beginn an zur Verfügung stehen
  - Harz wird wahrscheinlich später kommen

3

Bereits geplante Cannabisprodukte

4x Cannabisblüten

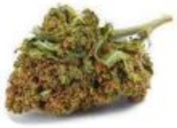

2x Cannabisharz

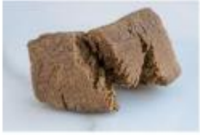

2x Cannabisöl

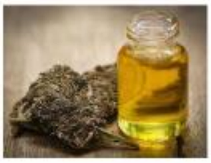

2x Cannabis E-Flüssigkeit

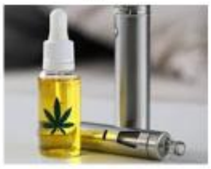

4

Zusätzliche Cannabisprodukte

UNIVERSITÄT BERN

Mundspray

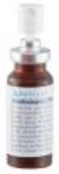

Crème für die Haut oder Schleimhaut (z.B. nasal, vaginal, anal) oder Zäpfchen

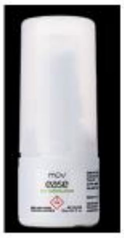

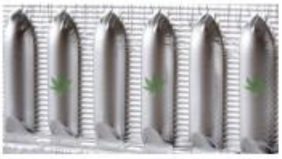

5

Fragen

UNIVERSITÄT BERN

Was kommt Ihnen spontan in den Sinn, wenn Sie daran denken, diese Produkte bei uns erwerben zu können?

Würden Sie solche Produkte kaufen? Welche?

Was müsste sein, dass Sie diese Produkte kaufen würden?
Für welche Gelegenheit würden Sie solche Produkte kaufen?

Was würden Sie garantiert nicht kaufen? Weshalb?

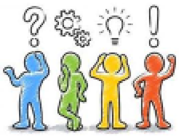

6

Zukünftiger Cannabismarkt

u^b UNIVERSITÄT BERN

Variante 1: gewinnorientiert, kompetitiver Markt

Viele Produktionsbetriebe
Produktevielfalt
Werbung, Aktionen, Promotionen
Ziel = Marktsteigerung, illegaler Markt schnell dämpfen
Diverse Verkaufsstellen
Preisdruck
Gewinne für die Betriebe und deren Aktionäre/ Inhaber
Zunahme von Konsumierenden

7

Zukünftiger Cannabismarkt

u^b UNIVERSITÄT BERN

Variante 2: nicht-gewinnorientiert, strikt regulierter Markt

Produktionsfirmen benötigen eine (kantonale/ eidgenössische) Zulassung/ Lizenz
Produkte sind vordefiniert
Bestimmte Verkaufsstellen
Preise sind vergleichbar/ gleich
Keine Werbung
Gewinne werden in Suchtprävention und zu weitere Zwecke für «community» investiert

8

Zukünftiger Cannabismarkt

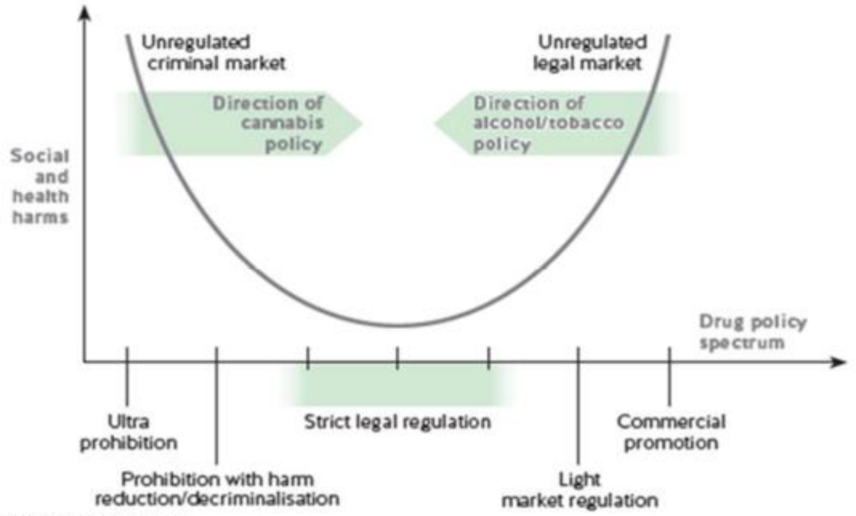

Transform 2013, Marks 2008

9

Zukünftiger Cannabismarkt

Variante 2: nicht-gewinnorientiert, kompetitiver Markt

Studie verfolgt einen ***nicht-gewinnorientierten, strikt regulierter, Ansatz***.

Den Preis, den ihr für das Produkt bezahlen werdet, deckt

1. Den Aufwand der Apotheken,
2. Den Aufwand für die Herstellung
3. Der restliche Betrag fliesst zurück in die Studie (z.B. um Gutscheine zur Aufwandsentschädigung der Teilnehmenden kaufen).

10

Fragen mit Fokus auf den Verkaufsmarkt

UNIVERSITÄT BERN

- Welches sind eure ersten Gedanken, wenn ihr diese Marktvarianten seht?
- Was wäre für euch der optimale zukünftige Cannabis Markt?
- Wo seht ihr in ***gewinnorientierten Ansätze*** Vor- und Nachteile?
- Wo seht ihr in ***nicht-gewinnorientierten Ansätze*** Vor- und Nachteile?
- Was spricht euch an? Weshalb?
- Was denkt ihr wo wird sich der Schweizer Markt einpendeln?

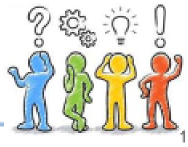

11

Fragen mit Fokus auf den Verkaufsmarkt

UNIVERSITÄT BERN

- Was denkt Ihr, soll Werbung erlaubt sein?
  - Wenn ja, in welcher Form?
  - Wo?
    - Plakate an Strassenränder
    - Fernsehspots
    - Kino
    - Verkaufsstellen
    - Spezifische Zeitschriften
    - Online (social media, influencer, websites, …)

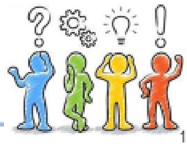

12

Fragen mit Fokus auf den Verkaufsmarkt

UNIVERSITÄT BERN

- Wer soll im Verkaufsmarkt aktiv sein?
  - Apotheken, Cannabisläden, Online Shops, Kiosk
  - Eine Stiftung, ein Verband, andere Non-Profit-Organisationen
- Auf welcher Ebene sollen Steuern erhoben werden?
  - Bund, Kanton, Gemeinde
- Für was sollen die erhobenen Steuern eingesetzt werden?
  - Prävention, Betreuung von Personen mit einer Cannabissucht, soziale Werke, AHV, …

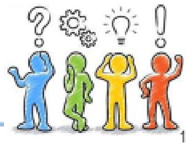

13

Alternativer Verkaufsort

UNIVERSITÄT BERN

Alternativ zu den Apotheken wird eine weitere Verkaufsstellenmöglichkeit evaluiert.

Im Moment ist die Stiftung Contact Bern im Gespräch
Es werden separate Cannabis-Verkaufsstellen sein

Die SCRIPT-Studie hat Interesse zu ergründen, ob, wer und wann diese alternativen Verkaufsstellen aufgesucht werden. So kann ein Vergleich mit den Apotheken gemacht werden.

14

Fragen

Wie steht ihr zu einer alternativen Verkaufsstelle?

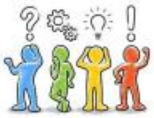

Was kommt euch in den Sinn zu:

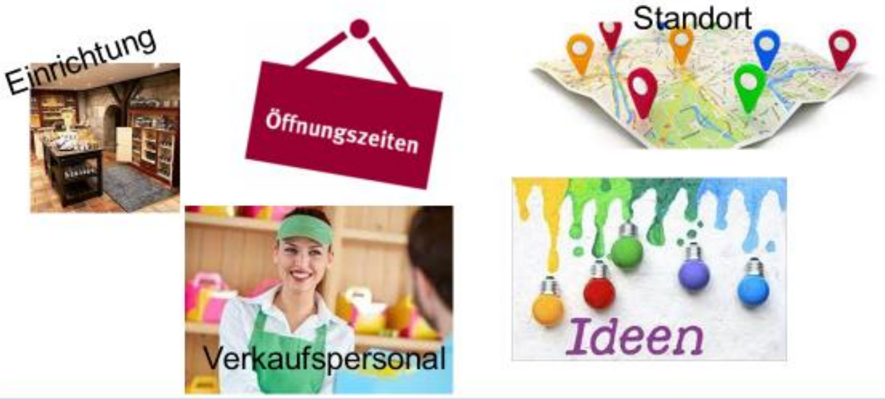

15

Alternativer Verkaufsort

- Gibt es im Vergleich zu den Apotheken eine Reihenfolge, welche anderen Verkaufsstellen ihr bevorzugt?
    - Welche Reihenfolge?
    - Was sind die Gründe für diese Wahl?

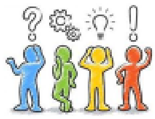

16

Auswahlverfahren Studienteilnehmende

- Es werden viele Anmeldungen erwartet
- Aussagekraft der Studie verbessert sich, wenn die Charakteristika der Teilnehmenden auf die ganze Breite verteilt sind

Überlegung zu Charakteristika

Gender 55% m, 35% w, 5-10% div.
Alter ab 18, gegen oben offen. Anzahl verteilt auf das ganze Altersspektrum
Konsumverhalten täglich oder nicht täglich
Wohnhaft in der Stadt Bern →Anmeldungen aus anderen Gemeinden kommen auf die Warteliste
Registrierung ist ab Sommer 23 möglich, Webseite wird bekannt gegeben

17

Frage

Was sagt ihr zum Auswahlverfahren?

Wie wirken die Kriterien auf euch?

Was erwartet ihr?

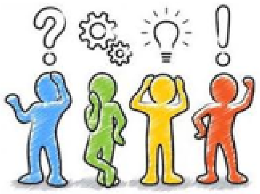

18

Ausblick

UNIVERSITÄT BERN

Die nächste Sitzung ist für den ***Mai 2023*** vorgesehen

Seid ihr bereit zwischenzeitlich ein Informationsschreiben und / oder einen Flyer zu lesen und eine Rückmeldung dazu zugeben?

Wer von euch hat die Möglichkeit, dies elektronisch zu tun?
Wer benötigt einen Ausdruck?

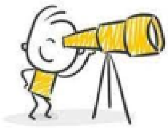

19

Abschluss

UNIVERSITÄT BERN

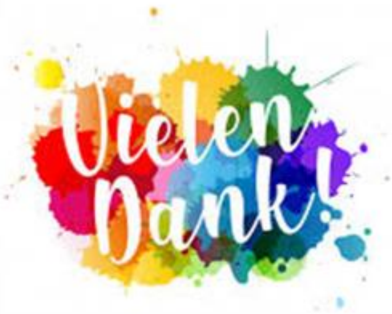

20

Laddering Technik:

Vertiefendes Nachfragen mit:

- Weshalb ist dir das wichtig?
- Was bedeutet dies für dich?
- Was verbindest du mit?
- Was würde dir fehlen, wenn xy nicht gegeben wäre? Was würdest du dann vermissen?
- Wie fühlst du dich bei xy?
- Was erwartest du aufgrund von …?

Auswertung der Gruppendiskussion der partizipativen Begleitgruppe von SCRIPT

Die Gruppendiskussion wurde mit Cannabiskonsumierenden Personen geführt

SCRIPT
The Safer Cannabis – Research In Pharmacies randomized controlled Trial

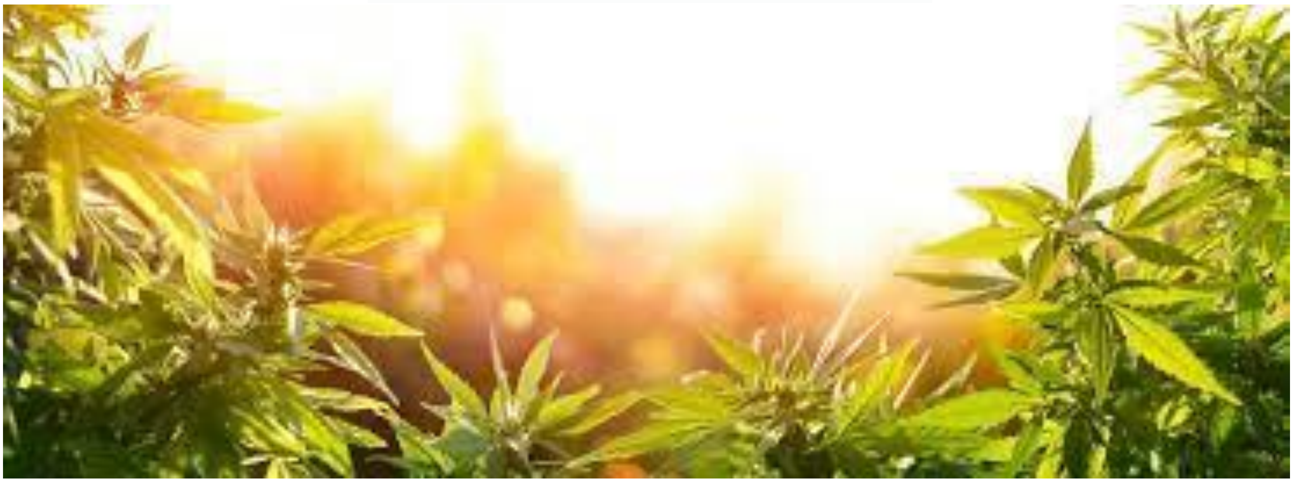

Beatrice Metry

Berner Institut für Hausarztmedizin

Juni 2023

Inhaltsverzeichnis

1 Einleitung 3
2 Methodisches Vorgehen 4
2.1 Zuständigkeitsabklärung bei der kantonalen Ethikkommission Bern 4
2.2 Erhebungs- und Auswertungsverfahren 4
2.3 Stichprobe 4
3 Ergebnisse aus der Gruppendiskussion 6
3.1 Studieninformationen und Einverständniserklärung 6
3.1.1 Verständlichkeit der Studieninformation und der Einverständniserklärung 6
3.1.2 Aussagen zu Inhalten der Studieninformation und Einverständniserklärung 8
3.1.3 Studienteilnahme 8
3.2 Rauchstoppberatung in der Apotheke 10
3.3 Case Report Forms 12
3.4 Website script-studie.ch 14
3.5 Informationen und Perspektive für die Begleitgruppe 15
4 Fazit 17
5 Anhang 1: Frageroute Gruppendiskussion 18
6 Anhang 3: Entscheidungshilfe zur Behandlungsmethode zur Unterstützung der Raucherentwöhnung 21
7 Anhang 2: Studieninformation und Einverständniserklärung 23

1 Einleitung

Im vierten Quartal vom Jahr 2021 wurde mit den neun Mitgliedern der partizipative Begleitgruppe der SCRIPT Studie Einzelinterviews geführt. Im Juli 2022 sowie im Februar und Juni 2023 traf sich die Gruppe je einmal zur Gruppendiskussion in den Räumlichkeiten der Universität Bern. Die Einzelinterviews wie auch diese Gruppendiskussionen wurden inhaltsanalytisch ausgewertet und die Resultate in einem Bericht dargestellt.
Bis dato sind folgende Berichte entstanden und erhältlich:

- Auswertung der Einzelinterviews zu SCRIPT vom März 2022
- Auswertung der Gruppendiskussion zu SCRIPT vom Juli 2022
- Auswertung der Gruppendiskussion zu SCRIPT vom März 2023
- Auswertung der Gruppendiskussion zu SCRIPT vom Juni 2023 (vorliegender Bericht)

Die partizipativen Begleitgruppe der SCRIPT-Studie traf sich am 5. Juni 2023 zur dritten Gruppendiskussion. Von den anfänglich neun Mitglieder der partizipativen Begleitgruppen, waren diesmal vier cannabiskonsumierende Personen anwesend, die sich unter der Moderation von Beatrice Metry (wissenschaftliche Mitarbeiterin) zu folgenden Themen austauschte:

- Die Einverständniserklärung für die Teilnehmenden
- Die Rauchstoppberatung in der Apotheke mit einem neu entwickelten Beratungsinstrument
- Die Case Report Forms
- Die Website www.script-studie.ch

In diesem Auswertungsbericht wird die Stichprobe skizziert und anschliessend die Ergebnisse den Themen entlang der Frageroute, welche im Anhang zu finden ist, vorgestellt. Im Anhang findet sich die Frageroute, das Rauchstoppinstrument und Angaben zu den Case Report Forms.

2 Methodisches Vorgehen

Nachfolgend werden die verschiedenen Teile des Vorgehens beschrieben. Alle befragten Personen haben vor dem Einzelinterinterview (2021) einen Informed consent unterzeichnet und sich durch ihre Unterschrift mit dem Interview sowie den nachfolgenden Gruppendiskussion und deren Auswertung einverstanden erklärt. Darin wurde ihnen Anonymität zugesichert.

2.1 Zuständigkeitsabklärung bei der kantonalen Ethikkommission Bern

Am 31. Mai 2021 wurde das Konzept zur qualitativen Begleitforschung mit dem Titel «Einsatz einer partizipativen Begleitgruppe bestehend aus Cannabiskonsumierenden Erwachsenen als Ergänzung während der Planungsphase des Projekts SCRIPT 2» bei der Kantonalen Ethikkommission (KEK) eingereicht, um deren Zuständigkeit zu prüfen. Die KEK befand, dass sie nicht zuständig sei. Dies bedeutet, dass diese Forschung nicht unter das Humangesetz Artikel 2, Absatz 1 fällt und Einzel- und Gruppeninterviews mit der ausgewählten Zielgruppe durchgeführt werden können, ohne ein Gesuch einzureichen. Nach diesem Bescheid[1] vom 20. Juni 2021 wurde mit der Akquise von Teilnehmenden gestartet.

2.2 Erhebungs- und Auswertungsverfahren

Die Daten wurden durch eine Gruppendiskussion entlang einer definierten Frageroute, während einem Zeitraum von zwei Stunden, durchgeführt und digital aufgezeichnet sowie anschliessend transkribiert.

Das Transkript wurden mittels der Software MAXQDA inhaltsanalytische ausgewertet. Das bedeutet in einem ersten Schritt die Codierung des Textes, anschliessend eine Verdichtung der einzelnen Aussagen sowie das Verschriftlichen der Ergebnisse.

2.3 Stichprobe

Für die Gruppendiskussion wurden alle neun Personen, welche bereits bei den Einzelinterviews mitgemacht und den entsprechenden Informed consent unterzeichnet haben, angefragt. Via doodle wurde der Termin vereinbart.

Sieben Personen hatten sich für diese Gruppendiskussion angemeldet und vier Personen erschienen zur Gruppendiskussion. Die nachfolgende Tabelle gibt eine Übersicht der Stichprobe.

[1] BASEC-Nr Req-2021-00609, Bescheid der Zuständigkeitsabklärung liegt vom 20.6.2021 vor

	**Einzelinterviews 4. Quartal 2021**	**Gruppen-diskussion 1 Juli 2022**	**Gruppen-diskussion 2 März 2023**	**Gruppen-diskussion Juni 2023**
**Anzahl Personen**	9	6	5	4
**Jahrgang**	1957, 1975, 1990, 1991, 1993, 1999, 2000, 2001, 2000	1957, 1975, 1991, 1993, 2001, 2002	1957, 1991, 1993, 2000, 2002	1957, 1975, 2000, 2002
**Branche Beruf**	Studium (3)<br>Logistik (2)<br>Finanzen (1)<br>Fotografie (1)<br>Grafik (1)<br>Fitness (1)	Studium (1)<br>Logistik (1)<br>Finanzen (1)<br>Fotografie (1)<br>Grafik (1)<br>Fitness (1)	Studium (2)<br>Finanzen (1)<br>Grafik (1)<br>Fitness (1)	Studium (2)<br>Grafik (1)<br>Fotografie (1)
**Anrede**	Frau 3<br>Mann 5<br>non-binär 1	Frau 2<br>Mann 3<br>non-binär 1	Frau 2<br>Mann 3<br>non-binär 0	Frau 1<br>Mann 2<br>Non-binär 1
**Nationalität**	Schweiz 9	Schweiz 6	Schweiz 5	Schweiz 4

3 Ergebnisse aus der Gruppendiskussion

Die Ergebnisse werden nachfolgend entlang der Frageroute aufgeführt. Zahlen in runden Klammern weisen darauf hin, wie viele der Befragten in diese Richtung geantwortet haben. Befindet sich keine Klammer hinter der Aussage, handelt es sich um eine Einzelnennung. In eckigen Klammern wird der Absatz in der MAXQDA-Datei (A), in welchem das genannte Zitat gefunden werden kann, aufgeführt.

3.1 Studieninformationen und Einverständniserklärung

Die Studieninformation und die Einverständniserklärung wurden den Teilnehmenden vor der Sitzung elektronisch zu geschickt. Sie erhielten den Auftrag, das Dokument (Studieninformation und Einverständniserklärung) zu lesen und ihre Fragen, Diskrepanzen im Text zu notieren und an die Sitzung mitzubringen.

Eine Person hat bereits vor der Sitzung schriftlich auf orthografische, grammatikalische Mängel sowie Halbsätze aufmerksam gemacht. Diese Erkenntnisse wurden bereits vor der Sitzung an das Forschungsteam weitergeleitet.

3.1.1 Verständlichkeit der Studieninformation und der Einverständniserklärung

Alle anwesenden Personen (4) äusserten, sie hätten die Studieninformation und die Einverständniserklärung verstanden. Was für alle offen blieb, war die Anzahl der Dokumente. Die Teilnehmenden erhielten ein Dokument, in welchem die Studieninformation, die Einverständniserklärung zur SCRIPT-Studie sowie die Einwilligungserklärung zur Weiterverwendung von Daten und Urinproben für spätere Studien beinhaltet. Für die Teilnehmenden waren dies drei Dokumente.

- Ein Dokument von Zeile 1-374: Die Studieninformation.
- Ein Dokument von Zeile 375-446: Die Einverständniserklärung zur Teilnahme an der SCRIPT-Studie.
- Ein Dokument Zeilen 447-479: Die Einwilligungserklärung für die Weiterverwendung von Daten und Urinproben dieser Studie in verschlüsselter Form.

In diesem Zusammenhang kam die Frage auf, ob die Teilnehmenden aus der SCRIPT-Studie ausgeschlossen würden, wenn sie lediglich die Einverständniserklärung zu SCRIPT geben würden (4). Eine Person äusserte folgendes:

> *Vielleicht ist es möglich, […] dass man sagt, jetzt unabhängig davon was man hier entscheidet, dass man trotzdem an der Studie weitermachen kann. Und diese Daten unabhängig von der aktuellen [SCRIPT-] Studie noch weiterverwendet werden können, falls man damit einverstanden ist.* [A 112]

Es wurde angeregt, nach Zeile 380 bzw. 447 folgenden Einschub zu machen (3):

- Unabhängig davon, ob ich mit der Weiterverwendung meiner Daten einverstanden bin oder nicht, bleibe ich bei der SCRIPT Studie dabei.

- Ich bin mit der SCRIPT-Studie einverstanden Ja/Nein
- Ich bin mit der Weiterverwendung meiner Daten und Urinproben für nicht näher definierte Forschungsprojekte auf unbestimmte Zeit auf einverstanden Ja/ Nein

Eine Person äusserte Verständnis für dieses Anliegen, meinte jedoch für sie wäre es auch ohne diesen Zusatz verständlich. Nachfolgend ein Zitat dieser Stimme:

> *Ich würde einfach nicht unterschreiben, wenn ich das nicht möchte. Ich bräuchte jetzt nicht noch eine Klausel, die mir erklärt, dass ich auch dabei bin, unabhängig von diesem zweiten Teil [Einwilligungserklärung zur Weiterverwendung der Daten und Urinproben Anm. der Autorin]. Das ist für mich wie klar. Aber ich sehe euren Punkt. Also fürs Verständnis, dass das einfacher ist. Ist es sowieso.* [A 146]

Eine Person gab an, die Aussage auf den Zeilen 226-228 seien verwirrlich. Sie formulierte dies wie folgt:

> *Ja, ich war verwirrt bezüglich Zeile 226. Wegen dem Besitz von Studiencannabis im öffentlichen Raum. Da steht Studiencannabis wird nicht beschlagnahmt, wenn die Originalpackung noch ungeöffnet ist und man nicht mehr als 10 Gramm auf sich trägt. Das habe ich nicht verstanden. Was heisst es, wenn es nun geöffnet ist, dann können sie es beschlagnahmen?* [A 46][2]

Drei Teilnehmende äusserten, dass sie davon ausgehen eine Kopie der Studieninformation, der Einverständniserklärung zur SCRIPT-Studie und der Einwilligungserklärung zur Weiterverwendung der Daten erhalten. Auf diese Weise würden sie auch sehen, wer die Prüfperson sei, und hätten etwas in den Händen. Dies wird mit nachfolgendem Zitat unterstrichen:

> *Also für mich ist völlig klar, dass ich eine Kopie erhalte.* [A 173]

Die vierte Stimme meinte zu diesem Thema:

> *Also jetzt, wo du das sagst, finde ich, ja why not. Aber es wäre jetzt nicht das Erste, was mir in den Sinn gekommen wäre, dass ich das [eine Kopie Anm. der Autorin] einfordern müsste. Ich glaube, ich habe da ein recht naives Vertrauen.* [A 177]

[2] Formulierungsidee der Autorin: Studiencannabis dürfen Sie bei einer Polizeikontrolle behalten, wenn:
- die originale Packung noch verschlossen/verschweisst ist.
- Sie insgesamt 10 Gramm oder weniger Cannabisblüten oder Cannabisharz und maximal 2 Gramm Gesamt-THC-Gehalt bei vermischten Cannabisprodukten auf sich tragen.
- Sie Ihren gültigen Studienausweis vorweisen können.

Auf den Zeilen 390-391 steht bereits «*Ich behalte die schriftliche Information und erhalte eine Kopie meiner schriftlichen Einwilligungserklärung.*» Dies war den Teilnehmenden während der Diskussion offensichtlich nicht bewusst.

3.1.2 Aussagen zu Inhalten der Studieninformation und Einverständniserklärung

Die Zeile 173 beinhaltet, den Hinweis zum Bern City Geschenkgutschein. Zwei Personen gaben an, nicht zu wissen, was damit gemeint sei. Es wäre deshalb praktisch, wenn direkt ein Link oder ein QR-Code dabei stehen würde.

Das Angebot, studienfremder Cannabis testen lassen zu können, wurde von allen Anwesenden (4) positive aufgenommen. Eine Person regte an, sie möchte das Ergebnis des Tests erhalten, wie nachfolgendes Zitat zeigt:

> *Was ich da noch cool fände oder empfehlenswert, wenn wir diese Ergebnisse auch bekommen könnten. Weil es wäre noch interessant zu sehen, was drin ist, in diesem Zeug, welches man selbst raucht. Angenommen, es sei vom Schwarzmarkt oder selbst gezüchtet.* [A 187]

Darauf konterte eine andere Person mit folgender Aussage:

> *Und lustig, da hatte ich das Selbstverständnis, dass ich da dachte: Höllen gut, dann hat man das ja gerade mal kontrolliert. Also ich hätte jetzt da nichts anderes erwartet, als dass wir da eine Rückmeldung erhalten, welche Schlüsse sie aus der Probe ziehen.* [A 199]

Ob die Ergebnisse zum getesteten Cannabis telefonisch oder schriftlich mitgeteilt werden, sei beides recht, so die Anwesenden.

3.1.3 Studienteilnahme

Alle vier Personen gaben an, sie wären bereit diese Einverständniserklärung zu unterschreiben und unter diesen Bedingungen an der Studie teilzunehmen. Einige Punkte gaben jedoch zu diskutieren. Darunter die Zeilen 429 – 435. Da steht zu Beginn: «Einverständnis für die Erfassung von Kontaktdaten eines Angehörigen und/oder der/des Hausärztin/Hausarztes». Zum Zeitpunkt der Diskussion (und des Lesens) war für die Teilnehmenden offen, weshalb diese Daten erhoben werden sollen. Drei Personen gaben an, dass der Hausarzt oder die Hausärztin für sie keine Option sei, wie nachfolgende Zitate zweier Stimmen belegen:

> *Also, der Hausarzt würde ich nie ankreuzen. Aber, man hat ja eine Wahl. […] [A 17]*

> *Mich hat der Hausarzt auch verwirrt. Also ich kann verstehen, dass du nein sagst. Und sonst, wenn wir darüber sprechen würden, wärest du vielleicht nicht zu einem Nein gekommen. Weil mein Hausarzt, weiss nicht, dass ich baffe. Das geht den auch nichts an. Und ich war ein bisschen irritiert, als ich das gelesen habe. [A 204]*

Ich glaube nicht, dass da jemand den Hausarzt angeben wird. Wenn du niemanden angeben kannst von Freunden und Familie, dann ist, so glaube ich, auch der Hausarzt nicht der richtige. Ich glaube, der ist nie der richtige. Ich glaube nicht, dass da viele den Hausarzt angeben würden. Und dann ist da die Frage: Ist das gescheit, ihn da drauf zu tun? [A 208]

Ich habe keinen Hausarzt. [A 210]

Für alle Teilnehmenden war offen, was unter dem Wort «Studiendaten» (Zeile 432) zu verstehen sei. Was genau, da erhoben werden solle, bleibe vage und dies sei genau zu definieren. Auch auf den Zeilen 140-142 bleibt dieser Aspekt offen. So wurde in der Gruppe diskutiert, ob es darum gehe mit einer Person, die sich nicht mehr melde in Kontakt zu treten oder ob Angaben zum Gesundheitszustand der Person erfragt würden oder ob Gewohnheiten zum Cannabiskonsum und den Erfahrungen mit dem Cannabiserwerb in Apotheken erfragt würden.

In der Diskussion entstand die Idee, lediglich von *Kontaktperson (4)* zu sprechen. Dies beinhalte alle Möglichkeiten, auch den Hausarzt oder die Hausärztin. Dazu folgende Zitate aus der Diskussion:

[…] Und Angehörige ist für mich völlig unproblematisch. Das ist vielleicht ein ungünstiges Wort. Da ist die Frage, könnte es auch ein Freund oder einfach eine Kontaktperson sein? Halt vielleicht nicht Familie, aber sonst jemand. [A 17]

Oder einfach Kontaktperson, das, was XY [andere teilnehmende Person Anm. der Autorin] gesagt hat. Das ist etwas allgemeiner und schliesst niemanden aus. [A 27]

Eine Person äusserte:

Ich finde, man könnte es theoretisch stehen lassen. Denn es tut niemandem weh, wenn es [die Hausärztin, der Hausarzt Anm. der Autorin] da steht. [A 212]

Und eine weitere Person ergänzte die *Kontaktperson* mit folgendem Zitat:

Oder man schreibt einfach "es darf auch der Hausarzt sein". So schliesst man ihn nicht aus. Man impliziert ihn indirekt. [A 215]

Ebenfalls zu diesem Punkt wurde in der Gruppe diskutiert, weshalb nicht direkt nach den Kontaktdaten der Kontaktperson gefragt werde (3). Nachfolgendes Zitat unterstreicht diese Ansicht:

Ich finde allgemein auf Zeile 436 sollte direkt die Angabemöglichkeit stehen. Mit der Bemerkung, wieso man diese Person ausgewählt hat. Das fand ich ein bisschen komisch.

> *Dann kreuze ich hier "ja" an und im Nachhinein kommt dann noch, welche Kontaktperson ich will. Das kannst du ja gerade direkt da angeben. [A 42]*

Im Zusammenhang mit diesen verschiedenen Einverständnissen und der Einwilligung zur Weiterverwertung der Studiendaten und Urinproben kam die Frage auf, ob diese Antworten einen Einfluss auf die Zulassung zur SCRIPT-Studie haben werden. Wie bereits unter Kapitel 3.1 erwähnt, wünscht sich die Mehrheit der Gruppe (3) Klarheit über die Studien-Ausschlusskriterien. Unter Absatz 9 (Zeilen 254-265) werden lediglich Kriterien zum Ausschluss vom Cannabisbezug aufgelistet.

3.2 Rauchstoppberatung in der Apotheke

Alle Befragten gaben an, es sei gut, dass in den Apotheken eine Rauchstoppberatung angeboten werde. Diese sollte jedoch gezielt, diskret und freundlich angesprochen und ein *Nein* des Kunden/ der Kundin sofort akzeptiert werden. Die Anwesenden fanden, ein Angebot zur Rauchstoppberatung bei einem Folgeeinkauf, wäre genug. Eine Person äusserte sie fände es gut, wenn an der Tür der Apotheke ein Schild angehängt würde, um sichtbar zu machen, dass hier Rauchstoppberatungen angeboten würden. So könnten die interessierten Kunden und Kundinnen selbst danach fragen. Nachfolgend das entsprechende Zitat:

> *Oder, vielleicht hat es an der Tür der Apotheke ein Schildchen, wo draufsteht: «Hier Rauchstoppberatung». Und dass sich die Leute angesprochen fühlen. Und nicht, dass sie [die Apotheker und Apothekerinnen Anm. der Autorin] bei dem der etwas in der Apotheke kaufen geht, fragen: Darf ich Ihnen noch eine Rauchstoppberatung geben? Das wäre dann so wie jene am Kiosk, die immer sagen: Darf ich Ihnen noch ein Schoggistängeli anbieten. Ja, sie können das machen. Aber ich denke, es sollte eher so sein, dass die Leute selbst danach fragen. So fühlen sie sich angesprochen. Das würde ich jetzt behaupten. Und das aber auch publik machen in der Apotheke, dass man das hier haben kann (eine Rauchstoppberatung). [A 251]*

Dieser Aussage konterte eine andere Person wie folgt:

> *Das kann aber auch den umgekehrten Effekt haben. Wenn jemand, der raucht zur Apotheke geht und dann sieht er da dieses Schild. Dann denkt der: Nein, ich gehe in eine andere Apotheke. Weil, ich kenne viele, die Rauchen und nicht damit aufhören wollen. Und denen geht es dann auch schnell einmal auf die Nerven, wenn dir das so aufgezwungen wird. Also ich finde es eher so diskret, wenn man eine Nebenfrage stellt. So: Ja, rauchen Sie? Das muss ja nur eine Nebenfrage sein. Nicht, dass dies gerade das Hauptthema wird.* [A 252]

Von den anwesenden vier Personen würden zwei Personen keine Rauchstoppberatung in Anspruch nehmen, weil sie sich als Gelegenheitsrauchende deklarieren und sie hätten keine

Nikotinabhängigkeit. Eine Person gab an, eine Rauchstoppberatung anzunehmen, da sie interessiert sei und gerne dazu lerne. Diese Person formulierte dies so:

> Ja. Ich denke, ich würde sicher einmal zuhören. Ich rauche, zwar nicht mega viel. Manchmal mehr, manchmal weniger. Jetzt heute zum Beispiel habe ich noch nicht geraucht. Aber ich bin immer daran interessiert, welche Optionen es gibt. Ich habe mir jetzt erst gerade eine E-Zigarette ohne Nikotin gekauft. Einfach aus Interesse. Und das hat mir eigentlich sehr gut gepasst. […] Aber ja, ich würde sicher reinhören. [A 271]

Die vierte Person meinte, sie würde zuhören, hätte jedoch im Moment ein geringes Interesse an einem Raucherausstieg.

Aktuell wird am BIHAM von einer Gruppe Pharmazeuten und Pharmazeutinnen an einer Entscheidungshilfe zur Wahl der Behandlungsmethode zur Unterstützung der Raucherentwöhnung (Anhang 3, Kapitel 6) gearbeitet. Diese Entscheidungshilfe wurde der Begleitgruppe vorgelegt mit der Frage, wie sie darauf reagieren würden, wenn ihnen in der Apotheke diese Entscheidungshilfe während der Rauchstopp-Beratung gezeigt würde.

Die befragten Personen gaben an, die Entscheidungshilfe sei übersichtlich (3), jedoch sei die Grafik simpler zu gestalten (3) ohne dies näher auszuführen. Eine weitere Stimme merkte an, dass alle wichtigen und interessanten Informationen auf dieser Übersicht zu finden seien. Einzig pflanzliche Alternativen wie Damiana würde darauf fehlen.

Eine Person äusserte, dass es zu dieser Übersicht auf alle Fälle eine persönliche Beratung brauche.

Auf die Frage, wie sie zu den beiden aufgeführten Produkten E-Zigarette und Nikotinbeutel stehen würden, antworteten sie unterschiedlich. Eine Person fand E-Zigaretten mit Nikotin zum Ausschleichen sei zwar akzeptabel, jedoch unverständlich Nikotinprodukte anzubieten. Denn grundsätzlich würden E-Zigaretten viele Giftstoffe enthalten und seien schädlicher als Tabakzigaretten. Diese Person machte dies am Verbot von Juuls fest, wie nachstehende Zitat aufzeigt:

> *Aber da sind sonst giftige Stoffe. Also mein erster Gedanke, wenn ich so etwas sehe, sind Juuls. Sind ja auch so E-Zigaretten gewesen, mit Liquids. Das waren von den ersten e-Zigaretten, die jetzt verboten sind.* [A 316]

Und drei weitere Personen äusseren, dass eine E-Zigarette für sie als Ersatz von Tabakzigaretten nicht in Frage käme.

Eine Person gab an, dass Rauchen für Zigarettenkonsumierende mehr sei als Nikotinkonsum. Es sei das Rauchen an und für sich, ein Ritual mit festem Ablauf. Dieselbe Person äusserte, sie sei erschrocken, ab der Suchtgefahr der E-Zigarette, welche auf dieser Übersicht ausgewiesen werde.

Eine weitere Stimme sagte, es sei gut gäbe es Alternativen zum Tabakrauchen. Diese Aussage wird mit nachstehendem Zitat untermalt.

> *Also ich glaube, man hat jetzt so die Meinung, dass Nikotin mega schädlich ist. Was in hohen Mengen stimmt. Es kann auch tödlich sein. Aber ich glaube, das Rauchen selbst mehr Krankheiten verursacht. Wie zum Beispiel COPD[3] oder so.* [A 353]

Und eine andere Person erwiderte darauf:

> *Es ist gut, es gibt Alternativen.* [A 360]

Die Nikotinbeutel fanden bei den Anwesenden (4) keine Zustimmung für den persönlichen Gebraucht, jedoch als Nikotinersatzprodukt für Menschen mit gesundheitlichen Beschwerden (2).

3.3 Case Report Forms

Eine Person gab gleich zu Beginn zu bedenken, dass Menschen ihr Erinnerungsvermögen meistens überschätzen würden und ein Fragebogen, welcher Fragen zu Ereignissen, die 6 Monate zurückliegen würden enthalte, kaum realitätsgetreue Antworten erheben würde. Diese Person regte deshalb, die Fragen zum Beratungsgespräch deutlich früher zu stellen.

> *1-2 Wochen nach dem Beratungsgespräch oder nach einer Woche. Dann kann ich noch sagen, wie ich das gefunden habe. Vor allem, wenn es dann noch in die Details gehen soll. Aber nach einem halben Jahr weiss ich noch, dass es stattgefunden hat.* [A 459]

Es wurde angeregt, Fragen zum Preis-Leistungsverhältnis (4), den Öffnungszeiten und zum erweiterten Angebot (E-Dampfer, E-Zigaretten, Papier, Filter etc.) aufzunehmen. Eine Person regte an, die Bedürfnisse der Kunden und Kundinnen anders abzufragen. Dazu wird folgendes Zitat eingefügt:

> *Geht es darum, dass man das Angebot noch mehr auf die Bedürfnisse ausrichten kann. Ich würde nicht nach jedem einzelnen fragen, sondern mich eher fragen: Hast du dein Cannabis in den letzten 6 Monaten ausschliesslich aus der Apotheke bezogen? Oder Apotheke und Schwarzmarkt? Und wenn du nicht ausschliesslich in der Apotheke gekauft hast, was sind deine Gründe. Dann habe ich genau das, was ich will. Glaube ich, wenn das die einzige Absicht ist. Weil lange Fragebogen, wo ich jedes (Produkte) schon vergessen habe, dass es das überhaupt gibt. Und so habe ich nachher sehr präzise deine Antwort. Wenn das das Einzige ist, was du wissen willst.* [A 436]

Darauf konterte eine andere Stimme wie folgt:

> *Ich glaube es ist schon wichtig, dass man alle Produkte abfragt. Damit kann man auch evaluieren, braucht es mehr? Braucht es weniger? Braucht es andere Sachen? Ich glaube*

[3] Chronic obstructiv pulmonary disease, zu Deutsch chronisch obstruktive Lungenerkrankung

> *für das Angebot ist das wichtig. Aber es ist sicher noch eine spannende Frage zum dazunehmen. Zum Fragen, hat man es nur in der Apotheke gekauft oder nicht oder beides?* [A 437]

Die Frage zu den Warnhinweisen[4] bzw. die Art der Warnhinweise wurde rege diskutiert. Den Anwesenden war bisher nicht bewusst, dass es auf der Verpackung einen Warnhinweis geben werde. So antwortet eine Person auf die Frage, ob sie Cannabis auch mit einem Warnhinweis kaufen würde, folgendes:

> *Es kommt ein bisschen darauf an, wie es aussieht. Also wenn es so grauenhaft ist wie bei der EU-Zigarette, dann würde ich das nicht kaufen. Nein, das mache ich nicht. Es ist einfach nur dramatisch. Da siehst du den Namen der Zigarette gar nicht mehr. […] Grauenhafte Bilder. Und das ist eine Gemeinheit. Das ist eine Frechheit.* [A 401 + 402]

Eine andere Person unterstreicht diese Aussage mit nachfolgendem Zitat:

> *Ja, weisst du so ein grusiges Militärgrün. Und nachher hat es so grosse (Bilder) von offenen Herzen und Raucherlunge und so. In der EU. Oder du hast nicht ein Display von allen verschiedenen Marken. Sondern es ist nur noch eine Einheit. Du musst haargenau wissen, was du kaufen willst*. [A 403]

Diese beiden Personen gaben an, lediglich eine sprachlichen Warnhinweis auf der Verpackung zu wünschen. Und falls doch ein Bild, dann nur eines vom Happy Hanfbauer.

Die anderen beiden Personen äusserten, nicht auf die Warnhinweise zu reagieren. Ihnen sei es egal, was da geschrieben sei, sie hätten Interesse am Inhalt.

Die Fragen zur Beratung wurden von zwei Personen als offen und allgemein gehalten bezeichnet. Eine dieser Personen regte an, vertiefter zu fragen.

> *Also ich finde, das ist im Vergleich zum oberen Fragebogen [zu den Produkten], ist das hier mega offen. Da kann man jetzt eigentlich fragen: War die Beratung informativ? Also mega einfache Fragen. Und wenn nicht, was hat Ihnen gefehlt. Ich finde diese Frage[5] ist mega allgemein und so offen gefragt.* [A 440]

Darauf reagierte eine Person wie folgt:

> *Also ich finde es gut, so wie es ist. Ich finde, wenn es noch mehr Fragen sind, dann bin ich dankbar, wenn es ja/nein Fragen sind. Weil an diesem Punkt ist man schon lange am Beantworten und ich hätte jetzt nicht mega Bock noch ausführlich über die Beratung zu schreiben. Deshalb ja/nein Fragen wären für mich am praktischsten. Zum Beispiel da*

[4] Die Warnhinweise auf der Verpackung vom Studiencannabis sind verständlich.
[5] Ich war mit der Beratung zufrieden.

> *finde ich es extrem gut, dass man einfach die Themen ankreuzen kann und man das nicht selbst aufschreiben muss. […] Und ich könnte jetzt auch nach einem halben Jahr noch sagen zu welchen Themen ich Informationen erhalten haben. Da fragen sie ja nicht nach welchen Informationen im Sinn von ausführlichen Details. Sie fragen nur: Ist dieses Thema angesprochen worden? Ja, nein. Das könnte ich durchaus sagen, auch nach einem halben Jahr noch.* [A 464]

Zu den Fragen zu Informationsquellen für einen risikoärmeren Cannabiskonsum, meinten zwei Stimmen, «BeGes[6]» sei nicht verständlich, dies sollte ausgeschrieben werden.

3.4 Website script-studie.ch

Zur Website äusserten die Befragten einige Komplimente. Alle fanden, die Website erfülle den Zweck (4). Die Website wurde als klar (2) und übersichtlich (2), schön und hinterlässt einen guten Eindruck, beschrieben. Ebenfalls wurden der Informationsgehalt (2) und die Umsetzung der Usability (2) positiv erwähnt. Die News (4) sowie die Studieninformationen (4) wurden übersichtlich wahrgenommen. Als besonders cool, wurde die Animation des Bildes von zwei Stimmen erwähnt.

Die Anwesenden hatten verschiedene Ideen und Anregungen, was an der Website noch verändert werden könnte. So wurde bemerkt, dass wer direkt auf Log In klickt und nicht das Dropdown-Menu nutzt, auf das Wordpress Log In gelangt. Da sind sich die Anwesenden einige, dies sollte unbedingt behoben werden.

Die FAQs könnten mit folgenden Fragen und Antworten ergänzt werden: Wie viel THC enthält ein Durchschnittsjoint? (Als Referenzwert. Mit 10 Gramm THC pro Monat können die wenigsten etwas anfangen) (3) Wer kann an der Studie teilnehmen? (3) Wer wird aus der Studie ausgeschlossen? (3). Ein Feld: Hast du noch eine Frage? Und da direkt zur E-Mail-Adresse oder zum Kontaktformular verweisen (2).

Wer mit der Maus über «Über uns» fährt, erhält das Dropdown-Menu, in welchem das Kontaktformular enthalten ist. Wer auf «Über uns» klickt, kommt lediglich zur Beschreibung der SCRIPT-Studie. Da gibt es kein Kontaktformular, was als Mangel angesehen wurde (3). Dazu merkt eine Person folgendes an:

> *Du darfst es nicht suchen. Du musst es gleich finden*. [A 584]

Eine Idee war, «Kontakt» als eigenen Ritter in die obere Leiste zu stellen und dafür, das «Über uns» als Unterseite unter Pilotstudie aufzuführen. Unter «Über uns» fänden es drei Personen schön, Bilder von Personen zu sehen, die diese Studie durchführen. Folgende Zitate zeigt dies auf:

[6] Berner Gesundheit

> *Unter "Über uns" wäre es nice ein paar Leute zu sehen, die da mitmachen*. [A 623]

> *Also, da bin ich auch deiner Meinung. Das ist doch irgendwie immer so, dass es unter "Über uns", immer ein Foto von irgendjemandem gibt.* [A 626]

Eine Person regte an, generell mehr Bilder auf die Website zu stellen. Da widersprach eine andere Person wie folgt:

> *[…] Ich muss dir widersprechen. Ich würde nicht mehr Bilder nehmen. Weil, was sind das für Bilder? […] Es muss nüchtern sein. Du willst ja keinen versifften Raum zeigen. Du erzählst eine Geschichte über das Kiffen, sobald du ein Bild nimmst.* [A 620]

Es entstand eine längere Diskussion, ob «News» so prominent und mit eigenem Ritter dargestellt werden solle. Dieselben Informationen erscheinen auf Home. Eine Person bezog sich auf die Personen, die sich anmelden würden, die würden sich nicht für News interessieren, gab sich diese Person überzeugt. Zwei andere Person entgegneten, dass es andere an der SCRIPT-Studie Interessierte gäbe. Für diejenigen seien die News zum Stand der Studie sehr wohl wichtig. Nachfolgendes Zitat unterlegt diese Aussage:

> *Aber es ist nicht nur fürs Anmelden. Es ist klar, dass man sich auf der Webseite anmelden kann. Aber ich finde trotzdem, es ist ein Anhaltspunkt, erstens Leute zu kontaktieren und zweitens zu Informieren. Also, wenn ich auf die Webseite gehe, dann will ich laufend informiert werden, was da gerade abgeht. Wir sind ja wie Teilnehmer der Studie. Wir wollen jetzt unser Zeug beziehen und so. Aber, wenn es die Möglichkeit gibt, mich auf der Webseite fortlaufend zu informieren, also: wir sind jetzt an diesem Schritt. Irgendwie in den anderen Kantonen gibt es jetzt auch Anläufe für ähnliche Studien. Etc. Das finde ich mega wichtig*. [A 629]

Ein weiterer Aspekt war das Impressum der Website. Eine Stimme äusserte sich wie folgt dazu:

> *Ja, das Impressum ist ein bisschen schlank. Beim Impressum hat es sonst noch e-Mail oder andere Kontaktangaben. Eben, es hat ja einen Kontakt da. Es ist mir einfach aufgefallen, dass das Impressum relativ schlank ist. Einfach diese 3 Unis. Und das Copyright. Aber das ist ok.* [A 640]

3.5 Informationen und Perspektive für die Begleitgruppe

Die Anwesenden wurden darüber informiert, dass die Forschungsgruppe daran arbeitet, eine Pilotstudie für die SCRIPT-Umsetzung zu lancieren. Während der Pilotstudie sollen die Personen der Begleitgruppe als Pilotgruppe fungieren und die Abläufe vom Cannabiserwerb in Apotheken zu prüfen und ihre Erfahrungen erneut in die Begleitgruppe miteinzubringen. Alle Anwesenden freuen sich darauf, wenn sie in einer Pilotgruppe mitwirken können.

Ebenfalls wurde die Gruppe darüber informiert, dass die Forschungsgruppe nach einem Weg sucht die Teilnehmenden der Begleitgruppe in die Gruppe aufzunehmen, die ab Studienstart Cannabisprodukte in der Apotheke beziehen kann.

4 Fazit

Die Teilnehmenden dieser Begleitgruppensitzung äusserten sich grundsätzlich positiv zur Einverständniserklärung, zum Rauchstoppberatungsinstrument, zu den Case Report Forms und zur Website www.script-studie.ch.

Die ***Einverständniserklärung*** würden alle vier sofort sowie sie unterschreiben. Zu diskutieren gab vor allem die Angabe eines Angehörigen oder des Hausarztes. Da äusserte die Gruppe eine *Kontaktperson* anzugeben, die auch der Hausarzt/ die Hausärztin sein kann, wäre zeitgemässer. Die Angaben zur Kontaktperson könnten aus auf dieser Einverständniserklärung erhoben werden.

Die ***Rauchstoppberatung*** erntete mässige Begeisterung bei den vier befragten Personen. Zwei Personen rauchen keinen Tabak. Eine Person fühlt sich wohl mit ihrem Zigarettenkonsum und eine weitere Person würde aus Interesse neues zu erfahren, eine Rauchstoppberatung annehmen. Das Instrument zur Rauchstoppberatung wurde als übersichtlich und informativ eingestuft.

Die ***Case Report Forms***, welche die Teilnehmenden nach 6 Monaten ausfüllen werden, ergänzten die Befragten mit weiteren Fragen zu Preis-Leistung der Produkte. Intensiv wurde über den Warnhinweis, welcher auf den Produkten stehen wird gesprochen. Dabei verglichen die Anwesenden, den Warnhinweis mit jenem der EU-Zigaretten.

Die ***Website*** der SCRIPT-Studie gefällt den Befragten. Nebst den positiven Aspekten gaben sie konkrete Hinweise zu Mängeln in der Websitestruktur. So öffnet zum Beispiel der Klick auf «über uns» das Wordpress-LogIn und das dürfe nicht sein, fand die Gruppe.

Die Begleitgruppe wurde darüber informiert, dass die Forschungsgruppe daran arbeitet, eine Pilotstudie vor der Umsetzungsphase zu lancieren und falls diese zustande kommt, die Begleitgruppenmitglieder als Pilotgruppe mit dabei sein wird.

Die nächste Begleitgruppensitzung ist für den September 2023 vorgesehen.

.

5 Anhang 1: Frageroute Gruppendiskussion

Vorbereitung Begleitgruppe für Cannabiskonsumierende

SCRIPT

05. Juni 2023 / 18:30 – 20:30

Begrüssung, Agenda, Dauer (5`)

Studieninformation und Einverständniserklärung (45`)

Die TN haben am 19.5.2023 eine Mail erhalten. Darin war das Dokument «Studieninformation und Einverständniserklärung» enthalten.

Der ***Vorbereitungsauftrag*** an die Gruppe lautete:

Ihr findet ein pdf im Anhang. Da sind am Rand die Zeilennummern, damit wir uns im Austausch gut orientieren können. Im Original werden diese gelöscht. Bitte lest das Dokument aufmerksam durch und haltet nachfolgende Fragen im Blick.

1) Verstehst du die Information und die Einverständniserklärung? Gibt es Stolpersteine oder verschwommene, missverständliche Formulierungen? Wenn ja, welche?
2) Bleiben nach dem Durchlesen für dich Fragen offen? Falls ja, welche? Bitte halte folgendes im Blick: Rechte, Pflichten und Ablauf. Deine Fragen bringst du bitte an Sitzung vom 5. Juni 23 mit.
3) Jetzt nach der Lektüre der Information und Einverständniserklärung, gibst du deine Zustimmung an der Studie teilzunehmen? Falls ja, was hat dich überzeugt? Falls nein, was sind deine Überlegungen dazu?
4) Ab Seite 10 beginnt die Einwilligungserklärung. Diese beinhaltet unterschiedliche Teile der Studie.
5) Studienteilname generell
    - Teilnahme an Interview während der Studie
    - Mitbringen von Cannabisproben zur Analyse (Eigenanbau oder Schwarzmarkt)
    - Erfassung von Kontaktdaten (Hausarzt / Hausärztin oder Angehörige) falls du selbst nicht mehr in der Lage wärst
    - Weiterverwendung von Daten und Urinproben □Dabei handelt es sich um zusätzliche Urinproben, die während der Studie an Studienvisiten mitgebracht werden. (Eine Urinprobe an 1. Studienvisite ist für alle obligatorisch. Siehe Absatz 4 Ablauf der Studienvisite).
        - Zu welchen dieser Studienteile sagst du ja? Probandeninformation

Vorgehen an der Sitzung:

Fragen 1- 5 aus der Vorbereitung aufgreifen und besprechen.

Weitere Fragen: Stigmatisierende Sätze? Fehlendes? Überflüssiges?

Rauchstoppberatung (30`) ppt

Cannabis TN dürfen Rauchstoppberatung in Anspruch nehmen. Die Apotheker haben dazu Instrumente, die eine Entscheidung von alternativen Produkten unterstützen sollen. Dazu habe ich folgende Fragen:

- Wie steht ihr zur Rauchstoppberatung in den Apotheken?
  - Würdet ihr das Angebot annehmen? Unter welchen Umständen?
- Wie wirkt diese Übersicht von Nikotinprodukten und alternativen Angeboten auf euch?
  - Wie würdet ihr reagieren, wenn ein Apotheker euch solche Unterlagen vorlegen würde?
  - Ist diese Übersicht für euch hilfreich? Weshalb?
  - Würdet ihr diese Auswahl schätzen?
  - Habt ihr Vorbehalte? Welche? Weshalb?
- Was denkt ihr zu den Angeboten E-Dampfer und Nikotinbeutel??

Case Report Forms (20`)

Das sind Formulare, die an den Studienvisiten von den Teilnehmenden ausgefüllt werden. Dabei geht es auch um die Zufriedenheit mit den Bezugsstellen. Diese Fragen dienen dazu, das Angebot und die Beratung während der Studie zu verbessern.

- Welche Fragen würdet ihr stellen, um Aussagen zur Zufriedenheit mit den Bezugsstellen zu erhalten?
- Welche Fragen würdet ihr stellen, um Aussagen zur Zufriedenheit der Beratung zu erhalten?
- Was ist euch als Konsumenten an der Beratung/ Bezugsstellen am wichtigsten?
- Was würdet ihr explizit fragen, um die Zufriedenheit mit Angebot und Beratung während der Studie zu verbessern?

Website www.script-studie.ch (10`) → Seiten auf Screen schalten

- Was ist euch aufgefallen?
- Was hat euch gefallen? Was weniger?

Information und Ausblick (5`)

Teilnehmende an der PBG können an der Studie mitmachen.

Versucht wird: Die TN PBG als ***Pilotgruppe*** ab Oktober 2023 einzuführen, um die Prozesse zu prüfen.

Falls Pilot abgelehnt wird:

- Alle TN kommen in Studie 50% in Kontrollgruppe, 50% in Interventionsgruppe
- Es wird angestrebt, dass alle PBG-TN in Interventionsgruppe kommen

Nächste Sitzung im Sept. 2023

6 Anhang 3: Entscheidungshilfe zur Behandlungsmethode zur Unterstützung der Raucherentwöhnung

Wahl einer Behandlungsmethode zur Unterstützung des Raucherentwöhnung

	NIKOTINERSATZPRODUKTE						KOMBINATION
**Produkt**	Nicorette® Sublingual Tablette<br>Liste D	Nicorette® / Nicotinell® Lutschtablette<br>Liste D	Nicorette® Kaudepot Nicotinell® Kaugummi<br>Liste D	Nicorette® Inhaler<br>Liste D	Nicorette® Spray zur Anwendung in der Mundhöhle<br>Liste D	Nicorette® Depotpflaster Nicotinell® Pflaster<br>Liste D	Pflaster & Nikotinersatztherapie mehrere Kombimöglichkeiten<br>Liste D
**Darreichungsform**	Sublingual-tabletten	Lutschtabletten	Kaugummi	Inhaler	Mundspray	Pflaster	Pflaster & anderes NEP
**Verfügbare Dosis**	2 mg	1 mg / 2 mg	2 mg / 4 mg	10 mg	1 mg / Sprühstoss	21, 14 und 7 mg / 25, 15 und 10 mg	siehe Pflaster & andere NEP
**Anwendung**	8-12x / Tag nach Bedarf	8-12x / Tag nach Bedarf	8-12x / Tag nach Bedarf	8-12x / Tag nach Bedarf	12-25x / Tag nach Bedarf	1x / Tag während 16 oder 24h oder nach Bedarf	Pflaster 1x / Tag + anderes Ersatzprodukt nach Bedarf
**Vorteil für Nutzer:innen**	• Unauffällig mit dem Rauchen aufhören	• Unauffällig mit dem Rauchen aufhören	• Erhältlich in verschiedenen Wirkstoffstärken und Geschmacks-richtungen<br>• aktiv aufhören	• «Hand-zu-Mund»-Bewegung wird simuliert	• Erhältlich in verschiedenen Geschmacks-richtungen<br>• Rasche Hilfe nach 30 s	• Erhältlich in verschiedenen Wirkstoffstärken<br>• 1x am Tag<br>• Nikotinabgabe rund um die Uhr<br>• Nicotinell®: 24h Pflaster	• Bei starkem oder unkontrollierbarem Rauchverlangen<br>• Wenn bei Monotherapie rückfällig
**Preis pro Schachtel**	~ 65.– CHF<br>100 Tabletten à 2 mg	~ 25.– CHF<br>36 Tabletten à 2 mg	~ 20.– CHF<br>30 Kaugummis à 2 mg	~ 27.– CHF<br>18 Patronen à 10 mg	~ 60.– CHF<br>150 Sprühstösse	~ 120.– CHF<br>14 Pflaster à 15 mg	~ 120.– CHF<br>14 Pflaster und 1 Schachtel schnell-wirksames Ersatzprodukt
**Preis pro Tag (im Vergleich zu einer Zigarettenschachtel*)**	~ 6.– CHF / Tag	~ 6.– CHF / Tag	~ 5.– CHF / Tag	~ 9.– CHF / Tag	~ 6.– CHF / Tag	~ 7.– CHF / Tag	~ 11.– CHF / Tag
**Wirksamkeit**	++	++	++	++	+++	++	+++
**Abhängigkeit**	+	+	+	+	+	+	+
**Hauptnebenwirkungen**	• Irritationen im Mund- und Rachenbereich<br>• Schluckauf<br>• Übelkeit	• Irritationen im Mund- und Rachenbereich<br>• Schluckauf<br>• Übelkeit	• Irritationen im Mund- und Rachenbereich<br>• Schluckauf<br>• Übelkeit	• Irritationen im Mund- und Rachenbereich<br>• Schluckauf<br>• Übelkeit	• Irritationen im Mund- und Rachenbereich<br>• Schluckauf<br>• Übelkeit	• Hautirritationen	• Hautirritationen, Irritationen im Mund- und Rachenbereich<br>• Schluckauf<br>• Übelkeit

Wahl einer Behandlungsmethode zur Unterstützung des Raucherentwöhnung

	OHNE MEDIKAMENTE			MEDIKAMENTÖSE THERAPIE			NEUARTIGE NIKOTINPRODUKTE (NICHT IN DER APOTHEKE ERHÄLTLICH)	
**Produkt**	Kognitive Verhaltenstherapie (KVT)<br>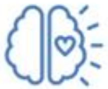	Motivational Interviewing<br>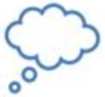	Raucherentwöhnung ohne Unterstützung (Reduzierung der gerauchten Zigaretten)<br>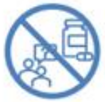	Vareniclin<br>Champix®<br>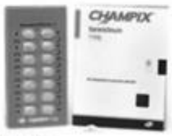<br>Liste B+	Bupropion®<br>Zyban®<br>*nur auf ärztliche Verschreibung*<br>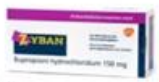<br>Liste A	Cytisin®<br>Tabex®, Desmoxan®, Cravv®, Asmoken®<br>*nur auf ärztliche Verschreibung*<br>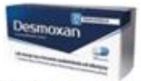<br>**In der CH nicht verfügbar**	Nikotinhaltiger Verdampfer<br>mehrere Marken<br>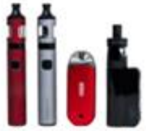<br>In Fachgeschäften verfügbar	Nikotinhaltige Beutel<br>mehrere Marken<br>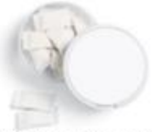<br>In Fachgeschäften verfügbar
**Darreichungsform**	Psychotherapie	Psychotherapie	Keine	Tablettten	Tabletten	Keine	E-Zigarette	Nikotinbeutel
**Verfügbare Dosis**	-	-	-	0.5 mg / 1 mg	150 mg	1.5 mg	verschiedene Dosierungen	verschiedene Dosierungen
**Anwendung**	-	-	-	2x / Tag	2x / Tag	6x / Tag, dann 2x / Tag	Wenn Medikamente nicht genug wirksam sind, nach Bedarf	Wenn Medikamente nicht genug wirksam sind, nach Bedarf
**Vorteil für Nutzer:innen**	• Keine medikamentöse Therapie<br>• Strategien lernen zur Bewältigung des Rauchdrangs<br>• Vertrauen in der eigenen Abstinenzfähigkeit stärken	• Keine medikamentöse Therapie<br>• Verhaltens-änderung durch Argumente auskommend von Patient:in, nicht von therapierender Person	• Keine medikamentöse Therapie<br>• Keine Sprechstunden nötig<br>• Verschiedene Methoden: Schlusspunkt- oder Reduktionsmethode	• Unauffällig mit dem Rauchen aufhören<br>• Kann unter bestimmten Bedingungen von der Grundversicherung übernommen werden	• Unauffällig mit dem Rauchen aufhören<br>• Kann unter bestimmten Bedingungen von der Grundversicherun g übernommen werden	• Unauffällig mit dem Rauchen aufhören<br>• Kann zur Tabakentwöhnung angewendet werden, wenn andere Therapieformen nicht zum Erfolg geführt haben	• «Hand-zu-Mund»-Bewegung wird simuliert	• Tabakfreies Produkt<br>• Erhältlich in verschiedenen Geschmacks-richtungen
**Preis pro Schachtel**	individuell<br>je nach Tarif / Anzahl Sitzungen	individuell<br>je nach Tarif / Anzahl Sitzungen	Keine Kosten	~120.– CHF<br>56 Tabletten à 1 mg	~60.– CHF<br>30 Tabletten à 150 mg	~90.– CHF<br>100 Tabletten à 1.5 mg	~50.– CHF<br>Starterkit – diverse Marken	~7.– CHF<br>1 Dose à 21 Beuteln
**Preis pro Tag (im Vergleich zu einer Zigarettenschachtel*)**	**individuell**<br>je nach Tarif / Anzahl Sitzungen	**individuell**<br>je nach Tarif / Anzahl Sitzungen	**Keine Kosten**	~ 4.– CHF / Tag	~4.– CHF / Tag	~3.60.– CHF / Tag	~2 bis 3.– CHF / Tag	~2 bis 3.– CHF / Tag
**Wirksamkeit**	++	+	(+)	+++	++	++	+++(+)	++
**Abhängigkeit**	-	-	-	-	-	-	+++	++
**Hauptnebenwirkungen**	• Keine	• Keine	• Reizbarkeit, Wut, Nervosität<br>• Müdigkeit<br>• Ängstlichkeit<br>• Schlechte Laune<br>• Craving	• Übelkeit<br>• Schlafstörungen<br>• Stimmungs-schwankungen<br>• Albträume	• Schlafstörungen<br>• Trockener Mund<br>• Kopfschmerzen<br>• Stimmungs-schwankungen<br>• Verdauungsstörungen	• Kopfschmerzen<br>• Übelkeit<br>• Schlafstörungen<br>• Magen-Darm-Beschwerden	• Irritationen im Mund- und Rachenbereich<br>• Mögliche Exposition gegenüber giftigen Verbindungen<br>• Husten	• Schluckauf<br>• Übelkeit<br>• Suchtgefahr

7 Anhang 2: Studieninformation und Einverständniserklärung

Einfügen der Information zur Teilnahme an einer Pilotstudie: The SCRIPT TRIAL

Information zur Teilnahme an einer Pilotstudie:

THE SCRIPT TRIAL

Eine randomisierte kontrollierte Pilotstudie über den regulierten Cannabisverkauf in Apotheken

Offizieller Titel: The Safer Cannabis – Research In Pharmacies randomized controlled Trial

---

Guten Tag

Vielen Dank für Ihr Interesse, an dieser Studie teilzunehmen.

Die Studie ist durch ein Team von Forschenden organisiert, die am Berner Institut für Hausarztmedizin (BIHAM) der Universität Bern arbeiten. Sie unterliegt strengen Regelungen und Datenschutzvorschriften. Eine Studienteilnahme ist freiwillig und kann jederzeit ohne Angaben von Gründen zurückgezogen werden.

Diese schriftliche Information beinhaltet nachfolgend eine kurze Zusammenfassung über die wichtigsten Punkte der Studie. Danach folgt ein detailliertes Informationsschreiben. Zusätzlich klären wir Sie mündlich über das Vorhaben auf und beantworten Ihre Fragen.

Wenn Sie sich dazu entscheiden, an dieser Studie teilzunehmen, bitten wir Sie, die schriftliche Einwilligungserklärung am Ende dieses Dokuments durchzulesen und mit Ihrer Unterschrift zu bestätigen, dass Sie mit den Anforderungen an die Studienteilnahme einverstanden sind.

Zusammenfassung

Warum führen wir diese Studie durch?

Der Cannabiskonsum ist in der Schweiz verbreitet und eine Regulierung wird seit Jahren diskutiert. Mit dieser Studie möchten wir die Auswirkungen auf die Konsumierenden untersuchen, wenn sie Cannabisprodukte in einem kontrollierten Rahmen beziehen können. Zusätzlich erhalten die Teilnehmenden Informationen über Vor- und Nachteile von unterschiedlichen Konsumformen, dabei steht die Aufklärung über Alternativen zum Rauchen im Vordergrund. Die Erkenntnisse aus dieser Studie sollen eine Wissensgrundlage für die allfällige Umsetzung einer Regulierung schaffen.

Was geschieht bei einer Studienteilnahme?

Die Teilnehmenden werden zufällig in zwei Gruppen eingeteilt. Die eine Gruppe darf von Beginn an Cannabisprodukte beziehen; die andere Gruppe darf dies erst nach 6 Monaten. Für alleTeilnehmenden dauert die Studie mindestens 1 Jahr und maximal 2 Jahre. Zu Beginn der Studie und nach 6 Monaten findet eine Studienvisite vor Ort statt , die restlichen Termine werden am Telefon oder per Mail durchgeführt. Im Fokus der Datensammlung stehen verschiedene Fragebögen zum Thema Gesundheit und Substanzkonsum.

Wie funktioniert der regulierte Cannabisverkauf?

Sie werden einen Studienausweis erhalten und können damit in einer ausgewählten Apotheke Cannabisprodukte beziehen. Das Angebot ist limitiert und beinhaltet Cannabisblüten, Cannabisharz (Haschisch), E-Flüssigkeiten und Cannabisöle. Die Abgabe und Information über Vor- und Nachteile findet in der Apotheke statt.

Habe ich einen Nutzen bei der Studienteilnahme oder setze ich mich einem Risiko aus?

Durch die Studienteilnahme entstehen für Sie keine zusätzlichen Risiken und es wird kein direkter persönlicher Nutzen erwartet. Alle Cannabisprodukte, die im Rahmen dieser Studie angeboten werden, unterliegen strengen Sicherheitsvorschriften und wurden im Labor geprüft. Ebenfalls haben Sie in der Apotheke eine Ansprechsperson bezüglich Fragen zu Gesundheit, Sicherheit und Abhängigkeit bei Substanzkonsum.

Detaillierte Studieninformation

1. Ziel der Studie

Das Hauptziel dieser Pilotstudie ist es, den Effekt der geregelten Cannabisabgabe, kombiniert mit Informationen rund um das Thema Cannabis- und Substanzkonsum, auf die Konsumierenden zu untersuchen. Im Vordergrund steht dabei die individuelle Gesundheit und das Thema Rauchen und alternative Konsumationsformen. Die Erkenntnisse aus dieser Studie sollen eine Wissensgrundlage für die allfällige Umsetzung einer Regulierung schaffen.

2. Auswahl der Studienteilnehmenden

An der Studie teilnehmen können Personen die

- bereits regelmässig Cannabis konsumieren,
- mindestens 18 Jahre alt sind und
- ihren Wohnsitz im Kanton Bern haben
- nicht bereits an einer anderen Studie mit reguliertem Cannabisverkauf teilnehmen oder teilgenommen haben

3. Allgemeine Informationen

Cannabis ist die in der Schweiz am häufigsten konsumierte illegale Substanz. Der illegale Konsum bringt jedoch negative Aspekte mit sich wie ungewisse Produkteverfügbarkeit und -Qualität sowie limitierte Anlaufstellen für Aufklärung und gegebenenfalls Hilfe. Mit der Änderung des Betäubungsmittelgesetzes, die im Mai 2021 in Kraft getreten ist, ist es nun möglich, die regulierte Cannabisabgabe wissenschaftlich zu untersuchen und zu analysieren, ob sich die Situation der Konsumierenden dadurch verbessern könnte.

In dieser Studie können Teilnehmende verschiedene Cannabisprodukte in einer ausgewählten Apotheke beziehen: Cannabisblüten, Cannabisharz (Haschisch), E-Flüssigkeiten und Cannabisöle. Sie erhalten zu Beginn der Studie eine separate Liste mit einer genauen Beschreibung der Produkte und deren Inhaltsstoffen. Alle Produkte werden in der Schweiz hergestellt, unterliegen strengen Qualitätsprüfungen und werden nach den Vorschriften der Bio-Verordnung produziert. Die Verkaufspreise berücksichtigen den Wirkstoffgehalt und die Preise auf dem Schwarzmarkt.

Gleichzeitig dient die Apotheke als Anlaufstelle für Informationen über Gesundheit und Cannabis sowie bei weiterem Substanzkonsum wie beispielsweise Tabak oder Alkohol. Im Vordergrund steht dabei die Beratung über den Konsum von Cannabis, insbesondere über Rauchen als wichtiger Risikofaktor für viele Krankheiten, und alternative Konsumformen wie beispielsweise das Vaporisieren, Verdampfen oder Essen von Cannabisprodukten

Damit die Effekte des regulierten Cannabisbezugs eindeutig bestimmt werden können, ist es wichtig, zu Beginn der Studie die Teilnehmenden zufällig in eine von zwei Gruppen einzuteilen. Die erste Gruppe (sog. „bezugsberechtigte Gruppe“) kann von Beginn weg Cannabisprodukte beziehen. Für die zweite Gruppe (sog. „Wartelistegruppe“) ist dies erst sechs Monate nach Studienbeginn erlaubt (siehe Abb. 1). Die Studie dauert für die Teilnehmenden mindestens 1 Jahr und maximal 2 Jahre. Dies ist vom individuellen Teilnahmestart abhängig. Insgesamt möchten wir mindestens 1‘000 Personen für diese Studie gewinnen können.

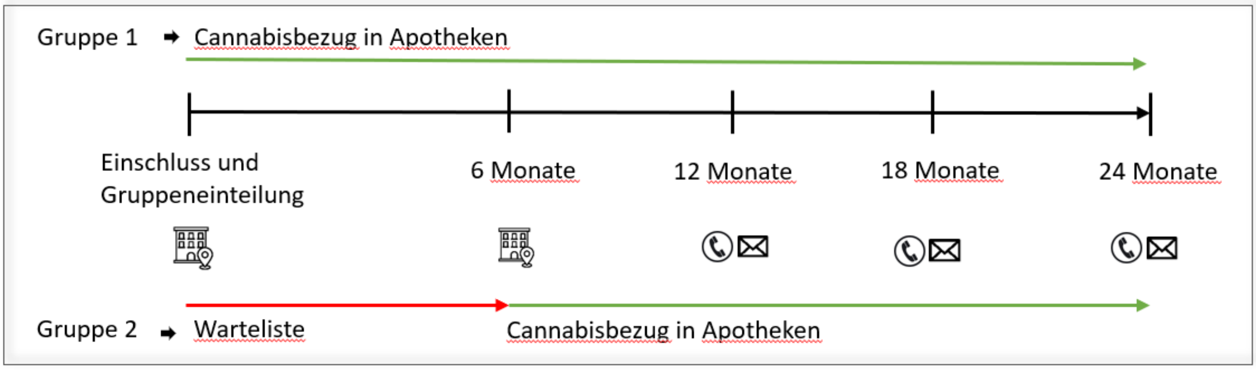

Abb. 1: Studiendesign; = Studienvisite vor Ort; = Studienvisite am Telefon / per Mail

Die Anzahl der Cannabisbezüge in der Apotheke ist nicht limitiert. Per Gesetz ist jedoch vorgeschrieben, dass pro Person und Bezug nicht mehr als total 10 Gramm Cannabis bei unvermischten Produkten und bei vermischten Produkten nicht mehr als 2 Gramm THC erworben werden darf. Weiter ist die Abgabe gesetzlich limitiert auf gesamthaft 10 Gramm THC pro Monat. Für den Besitz und Konsum von Studiencannabis gelten weitere gesetzliche Bestimmungen(siehe dazu Kapitel 8).

Wir führen diese Studie so durch, wie es die Gesetze in der Schweiz vorschreiben und beachten ausserdem alle international anerkannten Richtlinien. Die zuständige Ethikkommission und das Bundesamt für Gesundheit (BAG) haben die Studie geprüft und bewilligt. Eine Beschreibung dieser Studie finden Sie auch auf der Internetseite des BAG unter www.kofam.ch.

4. Ablauf der Studienvisiten

Für zwei Studienvisiten werden Sie gebeten, persönlich an das Studienzentrum zu kommen. An der ersten Visite vor Beginn der Teilnahme besprechen wir den Ablauf und unterschreiben die Einverständniserklärung. Zusätzlich prüfen wir mittels einer Urinprobe, ob sie bereits Cannabis konsumieren. Bitte beachten Sie, dass aus diesem Grund der letzte Cannabiskonsum nicht länger als eine Woche zurückliegen sollte. Da die Verordnung über Pilotversuche die Teilnahme von schwangeren Frauen ausschliesst, wird mit der Urinprobe bei Frauen auch noch eine Schwangerschaft ausgeschlossen. Danach machen wir eine kurze körperliche Untersuchung, die folgende Messungen beinhaltet: Grösse, Gewicht, Bauchumfang, Blutdruck und Ausatmungsluft. Die Ausatmungsluft brauchen wir zur Bestimmung von Kohlenmonoxid, welches einen Hinweis darauf gibt, wie stark Sie Rauch ausgesetzt waren. Zudem bitten wir Sie, einen Fragekatalog zu den Themen Substanzkonsum, insbesondere Cannabis, und Gesundheit zu beantworten. Schliesslich teilen wir Sie mittels Computer und mit einer Wahrscheinlichkeit vom 1:1 zufällig in eine der beiden Gruppen ein. Kommen Sie in die „bezugsberechtigte Gruppe“, erhalten Sie umgehend alle Informationen zum Cannabisbezug in einer ausgewählten Apotheke sowie einen Studienausweis.

An der zweiten Visite nach 6 Monaten machen wir wiederum die körperliche Untersuchung und bitten Sie erneut, einen Fragekatalog zu beantworten. Falls Sie in der „Wartelistengruppe“ waren, erhalten Sie nun auch die Informationen und den Studienausweis für den Bezug von Cannabisprodukten in einer Apotheke. Falls Sie für die Visite nach 6 Monaten nicht zur Verfügung stehen, möchten wir Sie bitten, eine Angehörige/ein Angehöriger oder Ihre Hausärztin/Ihr Hausarzt kontaktieren zu dürfen, um Ihre Studiendaten zu erfassen. In der Einverständniserklärung am

Ende dieses Dokuments können Sie angeben, ob Sie damit einverstanden sind, diese Kontakdaten anzugeben.

Die beiden Visiten vor Ort dauern für Sie je ca. 60 Minuten.

Nach 12 Monaten und je nach Teilnahmedauer auch nach 18 und 24 Monaten werden wir Sie nur noch per Telefon oder E-mail bitten, den Fragekatalog zu beantworten, wobei dies ca. 30 Minuten beanspruchen wird.

Urin-Stichprobe für Schadstoffbestimmung

Wir werden einige zufällig ausgewählte Teilnehmende bitten, eine Urinprobe an die erste Studienvisite und an die Visite nach 6 Monaten mitzubringen. Das Material sowie eine Anleitung werden zur Verfügung gestellt. Diese Proben werden wir brauchen, um verschiedene Schadstoffe die durch das Rauchen von Cannabis oder Tabak entstehen im Labor zu bestimmen.

Interviews über Cannabis und Cannabiskonsum

In der Einverständniserklärung am Ende dieses Dokuments können Sie angeben, ob Sie bereit wären, zusätzlich an einem Interview (möglicheriwese aufgeteilt in bis zu drei Terminen)teilzunehmen. Für uns ist es wichtig zu erfahren, was Sie über die regulierte Cannabisabgabe denken und wie sie das Angebot der Produkte einschätzen. Ein Interviewtermin kann telefonisch oder vor Ort gemacht werden und dauert ca. 60 Minuten. Sie erhalten für Ihre Teilnahme eine Vergütung (siehe Kapitel 14).

Cannabis-Stichproben für Laboruntersuchungen

In der Einverständniserklärung am Ende dieses Dokuments können Sie ebenfalls angeben, ob Sie bereit wären, an die Studienvisite nach 6 Monaten eine Cannabisprobe mitzubringen, die Sie auf dem Schwarzmarkt erworben oder selber gezüchtet haben und die wir im Labor auf ihre Inhaltsstoffe hin untersuchen könnten. Das Ziel der Laboruntersuchung ist es, den Gehalt von THC sowie verschiedenen Schadstoffen (Pestiziden und synthetische Cannabinoïde) zu analysieren und mit dem kontrollierten Cannabis aus der Apotheke zu vergleichen. Für eine Stichprobe benötigen wir 1 Gramm Cannabis, welches wir mit einer BERNcity Geschenkkarte im Wert von CHF 10.- rückvergüten. Bei Interesse stellen wir Ihnen die Resultate der Untersuchung gerne zur Verfügung.

5. Ablauf des Cannabisbezugs

Das Studiencannabis können Sie in einer ausgewählten Apotheke beziehen. Wenn Sie möchten, können Sie dafür das Beratungszimmer der Apotheke nutzen. Wir bitten Sie, sich für Ihren ersten Besuch per Telefon anzumelden und einen amtlichen Ausweis mitzubringen.

In der Apotheke werden Sie zuerst gebeten, einige Fragen betreffend Substanzkonsum und Gesundheit zu beantworten. Das Ausfüllen dieses Fragebogens dauert ca. 10 Minuten. Basierend auf Ihren Angaben kann Ihnen die Apothekerin/der Apotheker Informationen über Gesundheit und Cannabiskonsum, aber auch zu Tabak- oder Alkoholkonsum zur Verfügung stellen. Gleichzeitig werden Ihnen die unterschiedlichen Cannabisprodukte präsentiert und bei Bedarf werden Sie über die Vorteile von alternativen Konsumationsarten wie beispielsweise das Vaporisieren oder Verdampfen von Cannabisprodukten aufgeklärt. Sie können dabei jederzeit Fragen stellen. Anschliessend können Sie Ihr gewünschtes Produkt bzw. Ihre gewünschten Produkte auswählen und direkt vor Ort in bar oder mit Karte bezahlen. Bitte beachten Sie, dass Sie den Studienausweis für den Cannabisbezug nicht vorzuweisen brauchen. Wir empfehlen Ihnen aber, diesen bei sich zu

tragen, damit Sie bei einer allfälligen Polizeikontrolle den Besitz von Studiencannabis rechtfertigen können.

6. Nutzen

Durch das Mitmachen an dieser Studie wird kein direkter persönlicher Nutzen erwartet. . Ein Vorteil der regulierten Cannabisabgabe in dieser Studie im Vergleich mit der Beschaffung auf dem Schwarzmarkt, ist die Qualitätssicherheit der Produkte. Zudem kann der Stress, den die illegale Beschaffung unter Umständen mit sich bringt, vermieden werden. In der Apotheke können Sie sich zudem bezüglich Gesundheit, Sicherheit und Abhängigkeit von Cannabis und weiteren Substanzen wie z.B. Alkohol aufklären lassen. Wenn Sie Cannabis hauptsächlich rauchen, können Sie alternative, weniger gesundheitsschädigende Konsumformen ausprobieren. Auch wird Unterstützung bei der Rauchentwöhnung angeboten. All diese Angebote sind auf freiwilliger Basis. Neben dem persönlichen Nutzen ist in dieser Studie der gesellschaftliche Nutzen zu nennen. So sollen die gesammelten Erkenntnisse aus dieser Studie eine Wissensgrundlage für eine allfällige Umsetzung einer Cannabisregulierung schaffen.

7. Freiwilligkeit und Pflichten

Die Teilnahme an dieser Studie ist freiwillig. Wenn Sie nicht teilnehmen möchten oder später Ihre Teilnahme zurückziehen wollen, müssen Sie dies nicht begründen.
Wenn Sie teilnehmen, möchten wir Sie bitten, dass sie für die Studienvisiten zur Verfügung stehen und die Termine einhalten. Bitte beachten Sie, dass die Bewilligung für den Cannabisbezug von der Teilnahme an den Studienvisiten abhängt (vgl. Kapitel 9). Zudem möchten wir Sie bitten, dass Sie die Fragen über Ihren Cannabiskonsum und Ihre Gesundheit wahrheitsgetreu beantworten. Nur so können wir wichtige Erkenntnisse aus der Studie ziehen.
Schliesslich möchten wir Sie bitten, dass Sie sich an die unten beschriebenen Sicherheitsbestimmungen in Bezug auf den Besitz von Studiencannabis halten, welches sich nach den aktuell geltenden Vorschriften in der Schweiz richtet.

8. Sicherheitsbestimmungen

Zusammen mit der Polizei und weiteren städtischen Behörden haben wir Sicherheitsbestimmungen erarbeitet, welches die Durchführung der Studie im Hinblick auf den öffentlichen Raum regelt:

<u>Besitz von Studiencannabis im öffentlichen Raum</u>

Studiencannabis wird von der Polizei <u>nicht</u> beschlagnahmt, wenn:

- die originale Packung noch ungeöffnet ist
- sie nicht mehr als 10 Gramm Cannabisblüten oder Cannabisharz und nicht mehr als 2 Gramm Gesamt-THC-Gehalt bei vermischten Cannabisprodukten auf sich tragen
- Ihren gültigen Studienausweis vorweisen können.

Wenn Sie keinen Studienausweis vorweisen, kann Ihr originalverpacktes Studiencannabis beschlagnahmt werden, bis Sie als bezugsberechtigt identifiziert werden können. Bitte beachten Sie ebenfalls, dass eine geöffnete Packung beschlagnahmt werden kann. Die Studienleitung empfehlt Ihnen die Cannabisverpackungen nach Einkauf in Ihrem Wohndomizil zu lassen und den Besitz von Studiencannabis an gassenüblichen Drogenumschlagplätzen, im öffentlichen Nachtleben oder an anderen öffentlichen Veranstaltungen zu vermeiden.

<u>Weitergabe von Studiencannabis</u>

Die Weitergabe oder der Verkauf von Studiencannabis an Dritte und Minderjährige ist strafbar. Neben einer Rapportierung an die Staatsanwaltschaft erfolgt durch die Kantonspolizei eine Meldung an die Studienleitung. Wurde Studiencannabis nachweislich weitergegeben, wird die Studienleitung die Person verwarnen und im Wiederholungsfall vom Bezug von Studiencannabis ausschliessen.

<u>Konsum im öffentlichen Raum</u>
Bei Konsum von Studiencannabis im öffentlichen Raum gelten die üblichen strafrechtlichen Bestimmungen. Konsum im öffentlichen Raum kann durch eine Ordnungsbusse bestraft werden. Als nicht-öffentlicher Raum gelten private Räumlichkeiten und private, nicht gemeinschaftlich genutzte Aussenbereiche, wo der Konsum von Studiencannabis gestattet ist.

<u>Strassenverkehr</u>
Im Srassenverkehr gelten die üblichen strafrechtlichen Bestimmungen, unabhängig davon, ob Sie an der Studie teilnehmen oder nicht.

9. Ausschluss vom Cannabisbezug

Es kann sein, dass wir Ihnen das Recht auf den Bezug von Studiencannabis entziehen müssen. Dies kann sein, wenn

a) Sie schwanger werden
b) Sie Studiencannabis weitergeben oder verkaufen
c) Sie einen Gesundheitszustand entwickeln, bei dem der Studienarzt bestätigt, dass der Cannabiskonsum nicht ratsam ist (z. B. akute schwere Psychose);
d) Sie, trotz mehrmahligem Aufbieten, nicht innerhalb von drei Monaten an einer geplanten Studienvisite teilnehmen.
e) Sie während der Studie in einen Kanton ziehen, in dem die Studie nicht durchgeführt wird.

Falls Sie aus den Gründen a), b) oder c) vom Cannabisbezug ausgeschlossen werden, werden wir Sie trotzdem bitten, an den Studienvisiten teilzunehmen.

10. Risiken und Belastungen

Durch die Studienteilnahme entstehen für Sie keine zusätzlichen Risiken oder Belastungen, abgesehen vom Aufwand, den Sie für die Studienvisiten auf sich nehmen. Wie sie dem Kapitel 12 entnehmen können, handhaben wir Ihre persönlichen Daten vertraulich. Es besteht kein Austausch von persönlichen Daten mit der Polizei oder mit anderen (behördlichen) Einrichtungen, ausser, um Sie bei einer Kontrolle im öffentlichen Raum als Person, die an der Studie teilnimmt, zu identifizieren, sollten Sie Studiencannabis, aber keinen Studienausweis auf sich tragen.

11. Ergebnisse

Aus dieser Studie gibt individuelle Ergebnisse, die Sie direkt betreffen. Sie werden im Verlauf der Studie über alle für Sie persönlich wichtigen, neuen Ergebnisse und Erkenntnisse informiert. Möglicherweise gibt es auch individuelle Ergebnisse, die zufällig entstehen (sog. Zufallsbefunde), beispielsweise bei einer Urinanalyse. Bei Zufallsbefunden werden Sie informiert, wenn diese Befunde relevant für Ihre Gesundheit sind.

Damit sich aus der Studie aussagekräftige Resultate ergeben, werten wir nicht Ihre individuellen Ergebnisse aus, sondern fassen die Ergebnisse der Teilnahmegruppen zusammen und bilden sogenannte objektive End-Ergebnisse. Gerne lassen wir Ihnen am Ende der Studie eine Zusammenfassung dieser End-Ergebnissen zukommen.

12. Vertraulichkeit von Daten und Proben

12.1. Datenverarbeitung von Verschlüsselung

Für diese Studie werden Daten zu Ihrer Person und Gesundheit in einer online Datebank erfasst und bearbeitet, teilweise in automatisierter Form. Bei der Datenerhebung werden Ihre Daten verschlüsselt. Verschlüsselung bedeutet, dass alle Bezugsdaten, anhand derer Sie identifiziert werden könnten (Name, Geburtsdatum etc.), durch einen Code ersetzt werden. Ihre persönlichen Kontaktdaten werden in einer separaten Datenbank aufbewahrt, welche mittels technischen Vorkehrungen sicher von der Datenbank mit Ihren Studiendaten getrennt ist. Dies bedeutet, dass für nicht-authorisierte Personen aus Ihren codierten Studiendaten keine Rückschlüsse auf Ihre Person gezogen werden können. Zugang zu Ihren Studiendaten haben nur sehr wenige, authorisierte Fachpersonen. Dies schliesst das Studienpersonal in der Apotheke mit ein, in der Sie Cannabis beziehen, oder das Studienpersonal, welches die Studienvisiten durchführt, und zwar nur, um Aufgaben in Bezug auf die Studie wahrzunehmen. Auch Ihre Kontaktdaten dürfen nur vom Studienpersonal eingesehen werden, um Aufgaben im Rahmen der Studie zu erfüllen, beispielsweise um Ihre Identität beim Bezug von Cannabis zu überprüfen oder Sie für eine Studienvisite anzurufen. Alle Personen mit Zugang zu Ihren Daten unterliegen der Schweigepflicht. Sie als teilnehmende Person haben das Recht auf Einsicht in Ihre Studiendaten.

12.2. Datenschutz und Schutz der Proben

Alle Vorgaben des Datenschutzes werden streng eingehalten. Die online Datenbank wurde eigens für die Studie eröffnet, um Ihre Forschungsdaten sicher und verschlüsselt zu lagern. Es ist möglich, dass die Studiendaten aus der Datenbank übermittelt werden müssen, zum Beispiel für eine Publikation. Die Daten bleiben dabei verschlüsselt und Rückschlüsse auf Ihre Person sind nicht möglich.
Die Urinproben, falls Sie solche abgegeben haben, werden ebenfalls verschlüsselt und an das Labor der Unisanté in Lausanne zur Analyse übermittelt. Falls Sie einer Weiterverwendung der Urinproben zugestimmt haben, werden die Proben im Labor der Unisanté in einer Biobank gelagert. Ansonsten werden die Proben nach der Analyse vernichtet.
Die Cannabisprobe, falls sie eine solche abgegeben haben, wird ebenfalls verschlüsselt und an das Institut für Rechtsmedizin der Universität Bern zur Analyse übermittelt. Diese Probe wird nach der Analyse vernichtet.

12.3. Datenschutz bei Weiterverwendung

Ihre Daten und gegebenenfalls Urinproben, falls Sie solche abgegeben haben, könnten für die Beantwortung von weiteren Fragestellungen in der Cannabisforschung wichtig sein.
Für diese Weiterverwendung bitten wir Sie, ganz am Ende dieses Dokuments eine weitere Einwilligungserklärung zu unterzeichnen. Diese zweite Einwilligung ist unabhängig von der Teilnahme an dieser Studie.

12.4. Einsichtsrechte bei Kontrollen

Diese Studie kann durch die Bewilligungsbehörden (Ethikkommission) oder durch einen studien-internen Monitor überprüft werden. Das Studienpersonal muss dann Ihre Daten für solche Kontrollen offenlegen. Alle an diesen Kontrollen beteiligten Personen müssen absolute Vertraulichkeit wahren.

13. Rücktritt

Sie können jederzeit von der Studie zurücktreten. Im Falle eines Rücktritts werden Ihre bis dahin gesammelten Daten und gegebenenfalls Proben allerdings noch verschlüsselt in die Auswertung der Studie miteinbezogen. Die Daten und gegebenenfalls Proben werden nicht anonymisiert. Dies dient vorrangig zur Sicherung der Datenqualität. Prüfen Sie bitte, ob Sie damit einverstanden sind, bevor Sie bei der Studie mitmachen.

14. Entschädigung

Wenn Sie bei dieser Studie mitmachen, werden wir Ihnen Ihren Aufwand für die zwei Studienvisiten vor Ort mit je CHF 30.- und die Telefon- oder Onlinevisiten mit je CHF 20.- in Form einer BERNcity Geschenkkarte vergüten.
Die Teilnahme an einem Interview werden wir Ihnen mit CHF 30.- pro Stunde, ebenfalls in Form einer BERNcity Geschenkkarte, vergüten.
Es entstehen Ihnen oder Ihrer Krankenkasse keine Kosten durch die Teilnahme.

15. Haftung

Obwohl diese Studie kein vorhersehbares Risiko beinhaltet, haftet das Berner Institut für Hausarztmedizin (BIHAM) der Universität Bern nach den gesetzlichen Bestimmungen für alle Schäden, die im Rahmen dieser Studie entstehen könnten. Das BIHAM der Universität Bern hat für diese Studie eine Haftpflichtversicherung bei der Basler Versicherung AG (Aeschergraben 21, 4002 Basel) abgeschlossen, um in einem möglichen Schadenfall für die Haftung aufkommen zu können. Die Voraussetzungen und das Vorgehen dazu sind gesetzlich geregelt. Sollten Sie durch die Teilnahme an dieser Studie einen Schaden erleiden, so wenden Sie sich bitte an die im Kapitel 17 aufgeführte Kontaktperson.

16. Finanzierung

Einen Teil der Studie wird von der Abteilung „Substanzkonsum“ des Berner Instituts für Hausarztmedizin (Universität Bern) finanziert. Darüber hinaus beteiligen sich die teilnehmenden Gemeinden finanziell an den Kosten für die Planung und Durchführung der Studie.

17. Kontaktperson(en)

Sie dürfen jederzeit Fragen zur Studienteilnahme stellen. Bitte wenden Sie sich dafür an:

Prof. Dr. med. Reto Auer (Studienleitung)
Berner Institut für Hausarztmedizin (BIHAM)
Universität Bern
Mittelstrasse 43
3012 Bern

script@biham.unibe.ch
031 684 58 79 (Mo-Fr 8-17)

Einwilligungserklärung

Schriftliche Einwilligungserklärung zur Teilnahme an der SCRIPT Pilotstudie
Bitte lesen Sie dieses Formular sorgfältig durch. Bitte fragen Sie, wenn Sie etwas nicht verstehen oder wissen möchten. Für die Teilnahme ist Ihre schriftliche Einwilligung notwendig.

**BASEC-Nummer:**	2022-00733
**Titel der Studie**	Eine randomisierte kontrollierte Pilotstudie über den regulierten Cannabisverkauf in Apotheken<br>*Offizieller Titel: The Safer Cannabis – Research In Pharmacies randomized controlled Trial*
**Verantwortliche Institution**	Berner Institut für Hausarztmedizin (BIHAM)<br>Universität Bern<br>Mittelstrasse 43<br>3012 Bern<br>Schweiz
**Ort der Durchführung**	Kanton Bern
**Haupt-Prüfperson am Studienort**	Prof. Dr. med. Reto Auer
<u>**Teilnehmende Person**</u>	
Name und Vorname:	
Geburtsdatum:	

- Ich wurde von der unterzeichnenden Prüfperson (Studienassistenin/-assistent) mündlich und schriftlich über den Zweck und den Ablauf der Studie sowie mögliche Vor- und Nachteile informiert.
- Ich nehme an dieser Studie freiwillig teil und akzeptiere den Inhalt der mir ausgehändigten schriftlichen Information. Ich hatte genügend Zeit, meine Entscheidung zu treffen.
- Meine Fragen im Zusammenhang mit der Teilnahme an dieser Studie sind mir beantwortet worden.
- Ich behalte die schriftliche Information und erhalte eine Kopie meiner schriftlichen Einwilligungserklärung.
- Ich bin einverstanden, dass zuständige Fachleute wie studien-interne Monitore und Personen von der Ethikkommission zu Prüf- und Kontrollzwecken in meine unverschlüsselten Daten Einsicht nehmen dürfen, jedoch unter strikter Einhaltung der Vertraulichkeit und des Datenschutzes.
- Ich weiss, dass meine gesundheitsbezogenen und persönlichen Daten (und gegebenenfalls Urinproben) nur in verschlüsselter Form zu Forschungszwecken für diese Studie weitergegeben werden können. Die Studienverantwortlichen gewährleisten, dass der Datenschutz nach Schweizer Standard eingehalten wird.

- Ich kann jederzeit und ohne Angabe von Gründen von der Studienteilnahme zurücktreten. Die bis zum Rücktritt erhobenen Daten (und gegebenenfalls Urinproben) werden noch im Rahmen der Studie ausgewertet.
- Bei Ergebnissen und/oder Zufallsbefunden, die direkt meine Gesundheit betreffen, werde ich informiert.
- Ich bin darüber informiert, dass das Berner Institut für Hausarztmedizin (BIHAM) eine Versicherung abgeschlossen hat, welche Schäden, die auf das Forschungsprojekt zurückzuführen sind, deckt.
- Ich bin mir bewusst, dass mir im Interesse meiner Gesundheit das Recht auf den Bezug von Studiencannabis entzogen werden kann.
- Ich kenne die studienspezifische Sicherheitsbestimmungen (Kapitel 8) und halte mich an die Vorgaben.
- Ich weiss, dass Weitergabe und Verkauf von Studiencannabis strafbar sind und mir im Wiederholungsfall das Recht auf den Bezug von Studiencannabis entzogen werden kann.
- Ich werde für die Studienvisiten zur Verfügung stehen und die Termine einhalten. Die mir gestellten Fragen werde ich wahrheitsgetreu beantworten.

<u>Einverständnis für die Teilnahme an Interview</u>

Ich bin damit einverstanden, dass ich als teilnehmende Person für ein Interview (möglicheriwese aufgeteilt in bis zu drei Termine) ausgewählt werden kann (bitte ankreuzen):

☐ Ja
☐ Nein

<u>Einverständnis für das Mitbringen von Cannabisproben</u>

Ich bin damit einverstanden, dass ich angefragt werden kann, eine Cannabisstichprobe (*nicht Studiencannabis*) an die Studienvisite nach 6 Monaten mitzubringen (bitte ankreuzen):

☐ Ja
☐ Nein

<u>Einverständnis für die Erfassung von Kontaktdaten eines Angehörigen und/oder der/des Hausärztin/Hausarztes</u>

Ich bin damit einverstanden, dass nach 6 Monaten eine Angehörige/ein Angehöriger oder meine/mein Hausärztin/Hausarzt kontaktiert werden können, um Studiendaten von mir zu erfassen, falls ich selbst nicht dazu in der Lage bin (bitte ankreuzen):

☐ Ja → ☐ Angehöriger & Hausarzt ☐ nur Angehöriger ☐ nur Hausarzt
☐ Nein

Ort, Datum	Unterschrift teilnehmende Person

Bestätigung der Prüfperson: Hiermit bestätige ich, dass ich dieser teilnehmenden Person Wesen, Bedeutung und Tragweite der Studie erläutert habe. Ich versichere, alle im Zusammenhang mit dieser Studie stehenden Verpflichtungen gemäss des in der Schweiz geltenden Rechts zu erfüllen. Sollte ich im Verlauf der Studie von Aspekten erfahren, welche die

Bereitschaft der teilnehmenden Person zur Studienteilnahme beeinflussen könnten, werde ich diese Person umgehend darüber informieren.

Ort, Datum	Name und Vorname der Prüfperson in Druckbuchstaben<br><br><br>Unterschrift der Prüfperson

Einwilligungserklärung für Weiterverwendung von Daten und Urinproben dieser Studie in verschlüsselter Form

**BASEC-Nummer:**	2022-00733
**Titel der Studie**	Eine randomisierte kontrollierte Pilotstudie über den regulierten Cannabisverkauf in Apotheken<br>*Offizieller Titel: The Safer Cannabis – Research In Pharmacies randomized controlled Trial*
**<u>Teilnehmende Person</u>**	
Name und Vorname:	
Geburtsdatum:	

Ich erlaube, dass meine verschlüsselten Studiendaten und Urinproben, falls ich Proben abgegeben habe, für die Forschung weiterverwendet werden dürfen. Die Urinproben werden in einer Biobank der Universitätsklinik Unisanté in Lausanne gelagert und für zukünftige, noch nicht näher definierte Forschungsprojekte auf unbestimmte Zeitdauer verwendet.

Ich habe verstanden, dass die Daten und Proben weiterhin verschlüsselt bleiben und der Schlüssel sicher aufbewahrt wird. Die Daten und Proben können im In- und Ausland an andere Daten- und Biobanken zur Analyse gesendet werden, wenn diese dieselben Standards wie in der Schweiz einhalten. Alle rechtlichen Vorgaben zum Datenschutz werden eingehalten.

Normalerweise werden alle Daten und Proben gesamthaft ausgewertet und die Ergebnisse zusammenfassend publiziert. Sollte sich ein für meine Gesundheit wichtiges Ergebnis ergeben, ist es möglich, dass ich kontaktiert werde.

Ich entscheide freiwillig und kann diesen Entscheid zu jedem Zeitpunkt wiederzurücknehmen. Wenn ich zurücktrete, werden meine Daten verschlüsselt archiviert und meine Urinproben, falls ich Proben abgegeben habe, vernichtet. Ich informiere lediglich die Prüfperson am Studienzentrum und muss diesen Entscheid nicht begründen.

Ort, Datum	Unterschrift teilnehmende Person

Bestätigung der Prüfperson: Hiermit bestätige ich, dass ich dieser teilnehmenden Person Wesen, Bedeutung und Tragweite der Weiterverwendung von ihren Daten und gegebenenfalls Proben erläutert habe.

Ort, Datum	Name und Vorname der Prüfperson in Druckbuchstaben<br><br><br>Unterschrift der Prüfperson

Auswertung der Gruppendiskussion der partizipativen Begleitgruppe während der Aufbauphase der SCRIPT Studie

Die Gruppendiskussion wurde mit cannabiskonsumierenden Personen geführt

SCRIPT
The Safer Cannabis – Research In Pharmacies randomized controlled Trial

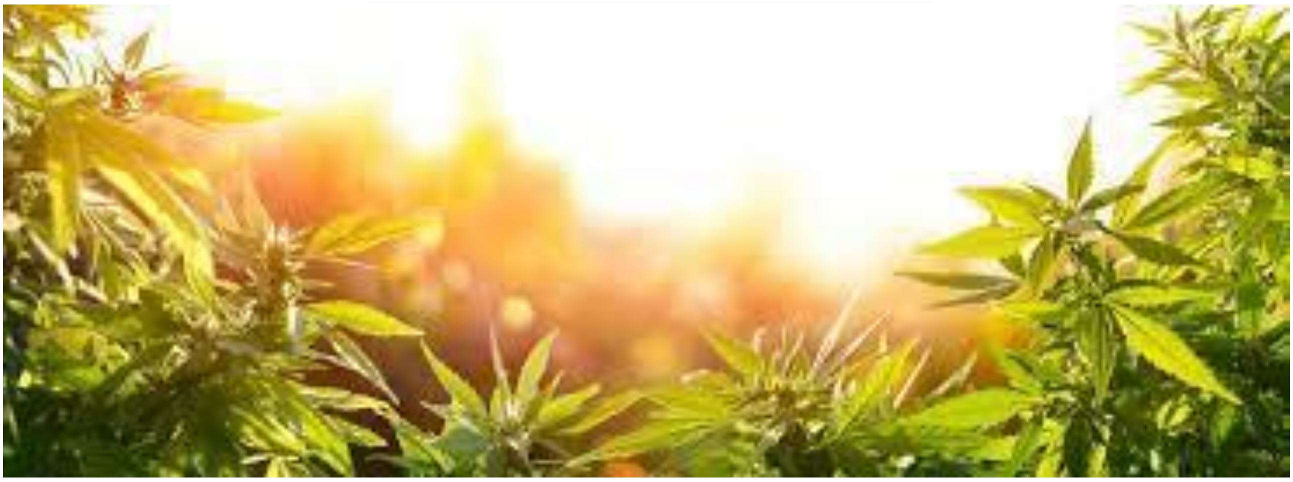

Beatrice Metry

Berner Institut für Hausarztmedizin

November 2023

Inhaltsverzeichnis

1 Einleitung 3
2 Methodisches Vorgehen 4
2.1 Zuständigkeitsabklärung bei der kantonalen Ethikkommission Bern 4
2.2 Erhebungs- und Auswertungsverfahren 4
2.3 Stichprobe 4
3 Ergebnisse aus der Gruppendiskussion 6
3.1 Informationen an die Teilnehmenden der partizipativen Begleitgruppe 6
3.2 Safer Use Rules 6
3.2.1 Inhalte der Safer Use Rules 6
3.2.2 Spalte mit den Links 7
3.2.3 Grafik zur Toxikologie 8
3.3 Video des Berner Instituts für Hausarztmedizin zu Risiken des Cannabiskonsums 9
3.4 Produktevideo Vaporizer 11
3.4.1 Video zu Wolkenkraft von VITA 11
3.5 Medienarbeit und Bereitschaft die Anmeldefragebögen zu prüfen 13
4 Ausblick 14
5 Fazit 15
6 Anhang 1: Frageroute Gruppendiskussion 16
7 Anhang 2: Safer Use Rules mit stehender Grafik 19
8 Anhang 3: Safer Use Rules mit liegender Grafik 20

1 Einleitung

Im vierten Quartal vom Jahr 2021 wurde mit den neun Mitgliedern der partizipative Begleitgruppe der SCRIPT Studie Einzelinterviews geführt. Im Juli 2022 sowie im Februar, Juni und November 2023 traf sich die Gruppe je einmal zur Gruppendiskussion in den Räumlichkeiten der Universität Bern. Die Einzelinterviews wie auch diese Gruppendiskussionen wurden inhaltsanalytisch ausgewertet und die Resultate in einem Bericht dargestellt.
Bis dato sind folgende Berichte entstanden und erhältlich:

- Auswertung der Einzelinterviews zu SCRIPT vom März 2022
- Auswertung der Gruppendiskussion zu SCRIPT vom Juli 2022
- Auswertung der Gruppendiskussion zu SCRIPT vom März 2023
- Auswertung der Gruppendiskussion zu SCRIPT vom Juni 2023
- Auswertung der Gruppendiskussion zu SCRIPT vom November 2023 (vorliegender Bericht)

Die partizipativen Begleitgruppe der SCRIPT-Studie traf sich am 16. November 2023 zur vierten Gruppendiskussion. Von den anfänglich neun Mitglieder der partizipativen Begleitgruppen, waren diesmal fünf cannabiskonsumierende Personen anwesend, die sich unter der Moderation von Beatrice Metry (wissenschaftliche Mitarbeiterin) zu folgenden Themen austauschte:

- Safer Use Rules
- Video zu Fakten des Cannabiskonsums
- Erwartungen an ein Produktevideo z.B. Vaporizer
- Weitere Themen: Medienarbeit, Rückmeldung zu den Fragebögen des Anmeldeverfahrens

In diesem Auswertungsbericht wird die Stichprobe skizziert und anschliessend die Ergebnisse den Themen entlang der Frageroute, welche im Anhang zu finden ist, vorgestellt. Im Anhang findet sich die Frageroute.

2 Methodisches Vorgehen

Nachfolgend werden die verschiedenen Teile des Vorgehens beschrieben. Alle befragten Personen haben vor dem Einzelinterinterview (2021) einen Informed consent unterzeichnet und sich durch ihre Unterschrift mit dem Interview sowie den nachfolgenden Gruppendiskussion und deren Auswertung einverstanden erklärt. Darin wurde ihnen Anonymität zugesichert.

2.1 Zuständigkeitsabklärung bei der kantonalen Ethikkommission Bern

Am 31. Mai 2021 wurde das Konzept zur qualitativen Begleitforschung mit dem Titel «Einsatz einer partizipativen Begleitgruppe bestehend aus Cannabiskonsumierenden Erwachsenen als Ergänzung während der Planungsphase des Projekts SCRIPT 2» bei der Kantonalen Ethikkommission (KEK) eingereicht, um deren Zuständigkeit zu prüfen. Die KEK befand, dass sie nicht zuständig sei. Dies bedeutet, dass diese Forschung nicht unter das Humangesetz Artikel 2, Absatz 1 fällt und Einzel- und Gruppeninterviews mit der ausgewählten Zielgruppe durchgeführt werden können, ohne ein Gesuch einzureichen. Nach diesem Bescheid[1] vom 20. Juni 2021 wurde mit der Akquise von Teilnehmenden gestartet.

2.2 Erhebungs- und Auswertungsverfahren

Die Daten wurden durch eine Gruppendiskussion entlang einer definierten Frageroute, während einem Zeitraum von zwei Stunden, durchgeführt und digital aufgezeichnet sowie anschliessend transkribiert.

Das Transkript wurde mittels der Software MAXQDA inhaltsanalytische ausgewertet. Das bedeutet in einem ersten Schritt die Codierung des Textes, anschliessend eine Verdichtung der einzelnen Aussagen sowie das Verschriftlichen der Ergebnisse.

2.3 Stichprobe

Für die Gruppendiskussion wurden alle neun Personen, welche bereits bei den Einzelinterviews mitgemacht und den entsprechenden Informed consent unterzeichnet haben, angefragt. Via doodle wurde der Termin vereinbart.

Sechs Personen hatten sich für diese Gruppendiskussion angemeldet und fünf Personen erschienen zur Gruppendiskussion. Die nachfolgende Tabelle gibt eine Übersicht der Stichprobe.

[1] BASEC-Nr Req-2021-00609, Bescheid der Zuständigkeitsabklärung liegt vom 20.6.2021 vor

	**Einzelinterviews 4. Quartal2021**	**Gruppen-diskussion 1 Juli 2022**	**Gruppen-diskussion 2 März 2023**	**Gruppen-diskussion 3 Juni 2023**	**Gruppen-diskussion 4 Nov. 2023**
**Anzahl Personen**	9	6	5	4	5
**Jahrgang**	1957, 1975, 1990, 1991, 1993, 1999, 2000, 2001, 2000	1957, 1975, 1991, 1993, 2001, 2002	1957, 1991, 1993, 2000, 2002	1957, 1975, 2000, 2002	1957, 1975, 1991, 1993, 2000,
**Branche Beruf**	Studium (3)<br>Logistik (2)<br>Finanzen (1)<br>Fotografie (1)<br>Grafik (1)<br>Fitness (1)	Studium (1)<br>Logistik (1)<br>Finanzen (1)<br>Fotografie (1)<br>Grafik (1)<br>Fitness (1)	Studium (2)<br>Finanzen (1)<br>Grafik (1)<br>Fitness (1)	Studium (2)<br>Fotografie (1)<br>Grafik (1)	Studium (1)<br>Finanzen (1)<br>Fotografie (1)<br>Grafik (1)<br>Fitness (1)
**Anrede**	Frau 3<br>Mann 5<br>non-binär 1	Frau 2<br>Mann 3<br>non-binär 1	Frau 2<br>Mann 3<br>non-binär 0	Frau 1<br>Mann 2<br>non-binär 1	Frau 1<br>Mann 3<br>Non-binär 1
**Nationalität**	Schweiz 9	Schweiz 6	Schweiz 5	Schweiz 4	Schweiz 5

3 Ergebnisse aus der Gruppendiskussion

Die Ergebnisse werden nachfolgend entlang der Frageroute aufgeführt. Zahlen in runden Klammern weisen darauf hin, wie viele der Befragten in diese Richtung geantwortet haben. Befindet sich keine Klammer hinter der Aussage, handelt es sich um eine Einzelnennung. In eckigen Klammern wird der Absatz in der MAXQDA-Datei (A), in welchem das genannte Zitat gefunden werden kann, aufgeführt.

3.1 Informationen an die Teilnehmenden der partizipativen Begleitgruppe

Zu Beginn der Sitzung wurde darüber orientiert, dass die ersten Studienteilnehmenden voraussichtlich ab Januar 2024 mit der Baseline Visite beginnen werden. Der Cannabisverkauf in Apotheken werde somit Ende Januar 2024 starten.

Des Weiteren wurden die Teilnehmenden darüber informiert, dass demnächst eine Medienmitteilung zur SCRIPT Studie erscheinen werde. Vor der Medienmitteilung werden die Teilnehmenden per Mail darüber orientiert, wann diese publik gemacht wird. In dieser Medienmitteilung werde auch das Anmeldeprozedere beschrieben. Die Begleitgruppenmitglieder wurden aufgefordert, sich offiziell über den ordentlichen Kanal anzumelden. Das bedeutet, sie melden sich an, wie alle anderen Studienteilnehmenden.

3.2 Safer Use Rules

Den Teilnehmenden wurden die Safer Use Rules (siehe Anhänge 7 und 8) als Power-Point-Folie digital gezeigt. Erste Reaktionen darauf waren: Die Informationen seien übersichtlich (2), verständlich und in kurzen Sätzen geschrieben (2). Zwei Stimmen äusserten sich kritisch zum Design. Die eine meinte, der viele Text würde schwerfällig wirken, allerdings gab die Person zu bedenken, sie wüsste nicht, welche Information sie streichen könnte, es sei alles wichtig. Die andere Person fand, diese Folie wirke wie eine Packungsbeilage. Um das Dokument für die Zielgruppe ansprechender zu gestalten, äusserten zwei Personen die Idee, das Bild eines Hanfblattes als Wasserzeichen im Hintergrund einzufügen. Und eine weitere Stimme merkte an, dass praktische Tipps für den Fall einer Überdosierung, die Motivation heben könnte, diese Empfehlungen zu lesen.

3.2.1 Inhalte der Safer Use Rules

Inhaltlich bemängelten vier Personen die Angaben zur Wirkdauer von Cannabis bei oralem Konsum. In den Safer Use Rules steht unter Punkt *3 Überdosierung vermeiden*: «Empfohlene Pausendauer: wenige Minuten bei inhalierter Form, bis eine Stunde bei oraler Einnahme.». Eine Person äusserte sich wie folgt dazu:

> Für mich wäre es sinnvoll, wenn man gerade bei Punkt 3 beim ersten Aufführungszeichen, die Stunden angeben würde. Dies im Sinn von geraucht oder vaporisiert vielleicht 3 Std. Wirkzeit und beim Oralkonsum ist es dann wirklich deutlich länger, 6-9 oder sogar 12 Stunden. […] Es wird potenziell unterschätzt. [A-60]

Vier der Teilnehmenden stimmten dieser Aussage zu. Alle Anwesenden waren sich darüber einig, dass eine Grafik analog jener zur Toxikologie verständliche Informationen zur Wirkdauer jeder Konsumform liefern würde.

Eine Person monierte die fehlende Differenzierung von Tabak- und Cannabisarten und zwei Stimmen äusserten zu diesem Zeitpunkt, eine Differenzierung der Filterqualität fehle. Die Filter wurden in Zusammenhang mit der Grafik zur Toxikologie näher diskutiert (s. Absatz 3.2.3).

Zwei Personen äusserten, ihnen fehle die Information, wie die Tageszeit die Wirkung von Cannabis beeinflusse. Die Anwesenden legten ihre Vermutungen dar. Eine klare Begründung wie die Tageszeit die Wirkung von Cannabis beeinflussen könnte, blieb offen.

Eine Person gab an, ihr fehle der Hinweis, dass bei körperlichen Beschwerden (z.B. Kopfschmerzen, Erkältung) auf den Konsum von Cannabis verzichtet werden solle. Da widersprachen zwei andere Personen, sie würden durchaus Cannabis konsumieren, wenn sie körperliche Beschwerden hätten, und sei es lediglich zur Entspannung des Körpers. Eine Stimme äusserte sich wie folgt dazu:

> Ich habe auch schon, als ich erkältet war […] gekifft, weil es mein Gesamtsystem beruhigt hat. Und ich hatte nicht das Gefühl, dass es die Symptome verschlimmert hätte. Es packt alles in Watte […]. Natürlich kannst du sagen, rauchen ist nicht gut, wenn du Atemwegsbeschwerden hast. Aber im Gesamtsystem kann es schon positiv wirken. [A-95]

Positiv aufgefallen seien die Nachvollziehbarkeit des Textes (3), informativ, sachlich und dass die Entscheidung bei der Leserschaft bleibe (keine Bevormundung) (2).

Des Weiteren kam die Frage auf, ob den Cannabisprodukten einen Beipackzettel beigelegt werde. Falls ja, könnte unter Punkt 3 angefügt werden:

> Für weitere Informationen konsultieren Sie die Packungsbeilage. [A-57]

Eine weitere Stimme äusserte sich wie folgt:

> Ich finde diese farbige Skala mega eindrücklich, weil sie so ein Blickfang ist. Und was es bedeutet, wird klar. Es ist nicht bevormundend, sondern sachlich. Das finde ich gut. Es beschreibt gut, auf was man schauen kann. [A-107]

3.2.2 Spalte mit den Links

Die grüne Spalte mit den Unterstützungsangeboten wurde als passend beurteilt (4). Eine Stimme meinte, es rege sie zur Reflexion des eigenen Cannabiskonsums an. Eine andere Person äusserte, ihr würden Angaben zum konkreten Angebot der jeweiligen Institution fehlen. Sie äusserte sich wie folgt:

> Mich stört es nicht, dass es da ist. Ich hätte jetzt keine Ahnung. Also, wenn ich mich jetzt wegen etwas an jemanden wenden möchte, dann ist mir klar, wenn ich mit dem normalen Rauchen ein Problem habe, dann ist dort "stopsmoking". Und irgendetwas zur Studie, dann ist es das. Bei den ersten drei finde ich, da ist null Information, wann und für was welche Institution steht. Ich habe das Gefühl, da wollte man einfach allen, die es so gibt, gerecht werden und sie erwähnen. Aber für mich als Konsument, der es für einen bestimmten Fall nutzen möchte, habe ich jetzt drei Adressen oder drei URL. Wieso sind es drei und wann wähle ich welche? Wenn ich dich kenne und du kennst diese drei, dann kannst du mir sagen für dein [Problem] würde ich dir diese [Adresse/ Institution] empfehlen. Aber wieso drei? Da ist sehr wenig oder keine Information. Da steht anonym. Sind die anderen nicht anonym? [A-72]

Eine Stimme entgegnete, dass lediglich bei *infodrog* offen sei, für was diese stehe. Dies könnte ergänzt werden mit «Schweizerische Koordinations- und Fachstelle für Drogenfragen/ Drogenkonsum/ Drogensachen. Eine weitere Person ergänzte mit «Drogen und mehr». Diese Ideen mochte jedoch nicht überzeugen. Denn offen bleibe wie diese Auswahl zustande kam und was diese Organisationen auszeichne hier genannt zu werden (s. letztes Zitat).

Es gab auch Anmerkungen zum Design dieser Spalte. So merkten zwei Personen an, dass der QR-Code zur SCRIPT-Studie aus optischen Gründen linksbündig gehalten werden solle. Und es kam die Frage auf, weshalb lediglich zu SCRIPT ein QR-Code gezeigt werde, es wäre einheitlich und für die Leserschaft praktisch, statt Links QR-Codes zu haben.

3.2.3 Grafik zur Toxikologie

Eine Person äusserte spontan, diese Grafik sei einleuchtend und zeige, was er selber erlebe (Unterschied von Rauchen und Vaporisieren). Eine andere Person zeigte sich überrascht, als sie sah, wie schädlich Rauchen mit Filter auf der Grafik eingetragen ist. Für alle Anwesenden war die Filterqualität ein relevantes Thema. Es entstand die Frage, für welche Filterqualität (Filter gerollt aus Karton, Zigarettenfilter, Aktivkohlefilter) die Aussage auf dieser Grafik stimme (5). Eine Stimme äusserte sich wie folgt zu den Filtern:

> Der einzige Kritikpunkt ist der mit den Filtern. Weil, bezüglich der Aktivkohlefilter, werden von den Hersteller Prozentzahlen erwähnt, von Schadstoffausfilterung. Und da habe ich das Gefühl, kann ich dem wirklich glauben? Ich habe schon gehört, dass wenn ich den [Kohleaktivfilter] nutze, ist es besser [gesünder]. Deswegen würde ich das schon nochmal ausschreiben. [A-39]

Die Anwesenden äusserten, eine Differenzierung der Filterqualität sei wichtig und die Nennung für welche Filterqualität die Aussage in der Grafik stimme, werde erwartet. Eine Stimme meinte, die Abbildung der Joints in der Grafik hätte sie verwirrt. Da stand mit und ohne Filter und abgebildet war der Joint jedes Mal mit Filter (s. Abbildung 1).

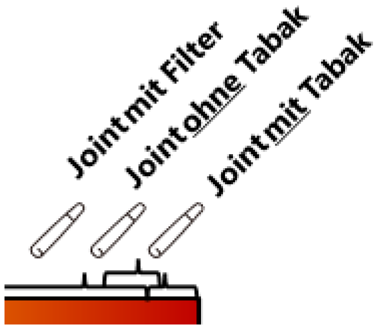

Abbildung 1 Joints aus der Grafik zur Toxikologie von Cannabiskonsumformen

Auf die Frage, wie die horizontale und die vertikale Grafik wirken, äusserten zwei Stimmen, die vertikale Grafik sei dramatischer, einprägender und sie mache die Gefahr deutlich. Das Dokument mit der horizontalen Grafik wurde als hübscher (3) und ansprechender wahrgenommen. Dieses Dokument bilde eine Einheit, ergänzte eine andere Person. Eine weitere Stimme meinte, bei der horizontalen Variante bräuchte es kein Cannabisblatt als Wasserzeichen. Da gab eine Person zu bedenken, dass zu viel Bewegung im Hintergrund, die Lesbarkeit beeinträchtige. Diese Person brachte die Idee ein, links und rechts neben dem Titel «7 Empfehlungen für einen sicheren Umgang mit Cannabis» ein Cannabisblatt abzubilden, das schaffe Verbindung und vermittle «Kiffen ist ok, und hier gibt's noch ein paar Tipps». [A-149] Ein Person merkte an, dass für sie offen sei, ob dramatisch oder hübsch richtig sei. Dazu folgendes Zitat:

> Die Frage ist, ist dramatisierend richtig oder falsch. Und das finde ich nicht klar. Weil das andere [vertikal] ist, die machen da einen auf Abschreckung. Vielleicht ist […] weniger dramatisierend intelligenter. Ich finde, das ist nicht klar. [A-147]

3.3 Video des Berner Instituts für Hausarztmedizin zu Risiken des Cannabiskonsums

Der Begleitgruppe wurde das Video von Dr. Bartlomiej Niznik zu Risiken des Cannabiskonsums gezeigt. Inhaltlich geht es um die Toxikologie, die Bioverfügbarkeit und die Absorptionsgeschwindigkeit der Wirkstoffe von Cannabis bei unterschiedlichen Konsumformen. Das Video dauerte 8 Minuten und 32 Sekunden und gestaltete sich als Power Point Präsentation mit sichtbarem Sprecher unten rechts.

Den ersten Eindruck beschrieben drei Personen als interessant, informativ, etwas Neues gelernt (rektale Anwendung von Cannabis), ein Experte der spreche, die Fakten wären gut, einzig die Angabe zur Wirkdauer bzw. Absorptionsgeschwindigkeit bei oralem Cannabiskonsum wurde bemängelt, wie nachfolgendes Zitat belegt und von allen Anwesenden bestätigt wurde:

> Teilweise hatte ich das Gefühl, es sei nicht praktisch orientiert. Also, eben, so Sachen, die aus meiner Sicht nicht wahr sind. Zum Beispiel das Nachlegen beim oralen Konsum. Also jeder der schon drauf ist oder schon einmal [Cannabis-]Kekse gehabt hat, der würde

> niemandem empfehlen: Nimm nach einer halben Stunde oder Stunde wieder, wenn du noch nichts merkst. [A-163]

> Das ist sehr gefährlich. Weil, es kann eine halbe bis zu vier Stunden dauern, bis man etwas merkt. [A-166]

Die Darstellung der Informationen wirke theoretisch (3) und erinnere an eine Vorlesung (3). Das hohe Anspruchsniveau der Inhalte (3), das Ablesen (3) und die gleichförmige Sprechweise warfen die Frage nach der Zielgruppe auf. Drei Personen meinten, sie hätten beim Sprecher die Leidenschaft zum Thema vermisst. Zwei andere Stimmen äusserten, wenn sie gekonnt hätten, hätten sie dieses Video weggeklickt. Auf die Frage, zu welchem Zeitpunkt sie weggeklickt hätten, meinte die eine Person, als das Wort *Bioverfügbarkeit* kam. Bioverfügbarkeit hätte sie nicht verstanden und eine Erklärung fehlte. Die zweite Stimme äusserte sich wie folgt zum Zeitpunkt des Wegklickens:

> Was soll es mir sagen? Nach einer gewissen Zeit hast du abgehängt, weil zu viele Unbekannte drin waren. [A-184]

Eine weitere Stimme gab an, sie habe etwa in der Mitte des Videos gedanklich abgehängt und nicht mehr zugehört. Zwei weitere Personen fanden das Video interessant, sie konnten den Inhalten folgen und blieben bis am Schluss aufmerksam dabei.

Fremdwörter benötigen eine Definition oder eine Erklärung, verlangten die Anwesenden (5). Drei Personen gaben an, das Wort Bioverfügbarkeit nicht verstanden zu haben. Bei einer anderen Person kam es zu einer Verwirrung zwischen Absorptionsgeschwindigkeit und Wirkungseintritt. Es gab Folien mit Pluszeichen (z.B. Bioverfügbarkeit der Konsumformen) und Folien mit Plus- und Minuszeichen (z.B. Zusammenfassung der Eigenschaften). Da gaben zwei Personen an, die Bedeutung dieser Zeichen nicht verstanden zu haben, wie folgendes Zitat untermalt:

> Eben, das habe ich überhaupt nicht so gelesen. Ich dachte da, hä, warum? Also ich habe nicht verstanden, was es mir sagen wollte über diese Toxikologie. Oder eben, der mit diesen Minus. Da kamen diese Farben hoch und ich dachte, hä? [A-271]

Eine Person merkte an, dass Cannabis, welches mit dem Vaporizer konsumiert werde, ein zweites Mal z.B. zum Backen oder Kochen genutzt werden könne. Also eine Mehrfachnutzung möglich sei. Das nennt sich «already vaped but» kurz AVB, und sei auch käuflich zu erwerben. Diese Information vermisse sie im Video. Wie bereits unter Absatz 3.2.3 ausgeführt, war auch hier die Filterqualität ein Thema, welches differenzierter aufgenommen werden dürfte. Zwei Personen regten an, die Überdosierung von Cannabis nach Konsumform und Cannabisprodukt zu differenzieren. So äusserte eine Person folgendes:

> Ich finde auch, zum Thema Überdosis könnte man es auch noch etwas mehr ausführen. Ich meine Überdosis ist nicht gleich Überdosis. Überdosis Rain wirkt anders als Überdosis

> Karisk. Dass man da noch mehr darauf eingehen könnte. Und was ist eine Überdosis des Kiffens und was ist eine Überdosis durchs Essen? Das finde ich auch anders und auch vom Zeitlichen her, die Wirkung ist anders. Und eben auch die Filter, das gehört auch dahinein. Aber die Facts waren gut. Wie gesagt, man muss nicht alles erläutern, aber dass es dagestanden ist, finde ich gut. [A-264]

Auf die Frage, wie das Video verbessert werden könnte, äusserte eine Stimme, dass sich die erste Frage dem Vermittlungskanal widmen sollte. Also welche Informationen sollen zu wem transportiert werden und welche Form eignet sich dafür. Erst wenn das geklärt sei, könne am Inhalt gearbeitet werden. Ideen dazu lieferten zwei andere Personen. Die eine Stimme nannte ein Erklärvideo als Möglichkeit und die zweite Stimme erinnerte an die animierte Büroklammer von Windows und leitet daraus ein animiertes Cannabisblatt als Sprecher:in ab. Eine weitere Verbesserungsidee war ein Sprecherwechsel (4), z.B. ein charismatischer Hanfbauer (2) oder eine Person mit praktischer Cannabiserfahrung (2), die die Umgangssprache frei nutzen. Das Ziel eines Sprecherwechsels, wäre mehr Praxiserfahrung und Leidenschaft zu vermitteln. Auch eine Stimme aus dem OFF, ohne Bild, wäre eine Variante, äusserte eine Person. Zwei Person meinten, die Folien könnten visueller gestaltet werden, also mehr Bild und weniger Text enthalten. Eine Person monierte die unregelmässige Formatierung der Folien, z.B. die Aufführungszeichen.

3.4 Produktevideo Vaporizer

Die Teilnehmenden wurden gefragt, welche Informationen sie in einem Produktevideo zu einem Vaporizer erfahren möchten. Folgende Aspekte wurden genannt: Vorteile des Vaporisierens (z.B. die Mehrfachnutzung s. Absatz 3.3) (3), Bedienungsanleitung (3), Preis (2), Akkulaufzeit, Materialien (2), insbesondere die Herkunft der Materialien und die Umweltbilanz, regulierbare Erhitzung möglich und wenn ja, mit welcher Wirkung auf den Konsum (2), Stromkabel oder Akkubetrieb, Angaben zur Wartung des Geräts und Informationen zur Garantie. Eine Person gab an, keine Erwartungen an ein Produktevideo zu haben.

Die Frage, wann die Teilnehmenden ein Produktevideo anschauen würde, äusserten sie folgendes: Wenn sie einen Vaporizer kaufen und sich informieren möchten (2), an der CannaTrade oder um Wartezeit zu füllen. Eine Person gab an, keine Produkte- bzw. Werbevideos zu schauen.

3.4.1 Video zu Wolkenkraft von VITA

Der Begleitgruppe wurde ein Video von Fourtwenty gezeigt. Darin wird der Vaporizer Wolkenkraft von VITA vorgestellt. Das Video dauerte 2 Minuten und 26 Sekunden und beinhaltete Werbung für Fourtwenty, den Grow- und Headshop von Bern.

Die Wirkung dieses Videos wurde heterogen wahrgenommen. So meinte eine Person, sie würde diesen Vaporizer sofort kaufen, er sei handlich und die Akkulaufzeit seit gut für unterwegs. Zwei Personen fanden, der Sprecher wirke authentisch und wisse, wovon er spreche. Zwei andere Stimmen fanden, der Sprecher wirke, als fühle er sich nicht wohl in dieser Situation vor der Kamera.

Weiter wurde geäussert, dass die Hintergrundmusik angenehm war (3), sie mit der Vape Wool ein neues Produkt kennengelernt haben (2), die kurzen Sätze gut verständlich waren (2) und das Video geschnitten war, was positiv bewertet wurde. Eine Person merkte zu dem an, sie sei nie abgeschweift oder ausgestiegen und konnte bis am Schluss aufmerksam zu hören.

Zwei Personen taxierten dieses Video als «schlecht». Zum einen spreche sie das Video nicht an und zum anderen werde Nebensächliches wie die Gebrauchsanleitung erwähnt, dafür würden andere Informationen fehlen. Eine Person merkte an, sie würde lieber die Fakten durchlesen.

Die Informationen, welche die Teilnehmenden in einem Produktevideo erfahren möchten (s. oben), wurden lediglich teilweise genannt.

Die Werbung von Fourtwenty im Video störte niemanden (5), da es sich um einen kleinen, lokalen Anbieter handle. Wäre es eine Verkaufskette fänden die Teilnehmenden die Werbung störend (5). Die Teilnehmenden äusserten Vertrauen zu Fourtwenty zu haben (4). Lediglich eine Person gab an, Fourtwenty vor dem Video nicht gekannt zu haben.

Eine Person äusserte Bedenken, ob die Universität Bern während einer Studie Schleichwerbung machen darf. Sie formulierte dies wie folgt:

> Also es ist ja eine Uni-Studie. Und ich glaube, das ist manchmal schon zweischneidig, wenn die Uni im Rahmen der Studie Schleichwerbung für einen Laden macht. Ich finde, man kann Vaporizer vorschlagen, aber die Frage ist, muss es ein bestimmter sein? […] Also ich weiss nicht, ob das ideal ist. Also ich habe auch das Vertrauen in Fourtwenty, aber ob es das wirklich in der Uni-Studie braucht, noch so Schleichwerbung zu machen, da bin ich nicht sicher. [A-376]

Eine andere Stimme bracht die Justiz ins Spiel, dies mit der Frage, ob die Universität überhaupt bestimmte Produkte bewerben darf, ohne sichtbar zu machen, wie es zu dieser Auswahl kam. Nachfolgende Zitate belegen diese Aussagen:

> Dürft ihr als Uni mit einem Geschäft und einem Typ Vaporizer werben? [A-381]

> Also habt ihr konsumentenschutzmässig eine Untersuchung gemacht und die sieben besten dürfen dieses Video reinstellen? Das fände ich super, dann möchte ich das aber auch kommuniziert haben. Dass ihr alle getestet habt und diese sieben sind gut und deshalb gebt ihr da die Plattform, um diese zu bewerben. Dann habe ich eine Erklärung. Aber diese Erklärung möchte ich eigentlich. [A-385]

3.5 Medienarbeit und Bereitschaft die Anmeldefragebögen zu prüfen

Die Medienarbeit findet in der Begleitgruppe Anklang. Vier der Anwesenden sind bereit bei Medienanfragen mitzuwirken. Die Mail-Adressen dieser vier Personen werden an Kathrin Bieri, Koordinatorin SCRIPT, weitergeleitet. Die Anfragen werden direkt von Kathrin Bieri gemacht.

Eine Medienmitteilung steht bevor und darin wird das Anmeldeprozedere beschrieben. Ab diesem Zeitpunkt wird die online Anmeldung für die Teilnahme an der SCRIPT-Studie möglich sein. Um die Anmeldefragebögen zu testen, wurden die Teilnehmenden gefragt, ob sie bereit wären Zeit dafür aufzuwenden. Alle Anwesenden (5) gaben an, die Fragebögen testen zu wollen und dazu eine schriftliche Rückmeldung auf einem vorbereiteten Formular zu geben. Kathrin Bieri wird den fünf Personen einen Link, die Zugangsdaten und das Rückmeldeformular zukommen lassen.

4 Ausblick

Die nächste Begleitgruppensitzung ist für den Februar 2024 geplant. Mögliche Themen werden

- erste Rückmeldungen zur Studien- und Verkaufsabläufe,
- ein Aufzeigen der Wirkung der Begleitgruppe auf das Studienprotokoll und
- eine Retrospektive auf die Arbeit innerhalb der Begleitgruppe sein.

5 Fazit

Es steht eine ***Medienmitteilung*** bevor. Da werde auch das Anmeldeprozedere beschrieben. Ab diesem Zeitpunkt können sich Interessierte anmelden. Die Begleitgruppenmitglieder wurden darüber informiert, dass sie sich auf dem offiziellen Weg anmelden müssen, um an SCRIPT teilzunehmen.

Die ***Safer Use Rules*** wurden von den Befragten mit übersichtlich und verständlich kommentiert. Inhaltlich wurde die fehlende Angabe zur Filterqualität und die als fehlerhaft empfundene Angabe zur Wirkzeit von oral konsumiertem Cannabis moniert. Eine Grafik zur Wirkzeit analog jener der Toxikologie könnte die Verständlichkeit unterstützen. Die Grafik zur Toxikologie zeigt in vertikaler Ausrichtung die Gefahr eindrücklich auf. In horizontaler Ausrichtung wirke die Grafik hübscher und füge sich geschmeidiger ins Dokument ein. Die grüne Spalte am rechten Seitenrand wurde mehrheitlich als hilfreich empfunden.

Das ***Video des Berner Instituts für Hausarztmedizin zu Risiken des Cannabiskonsums*** wurde als interessant, informativ beschrieben. Sie hätten etwas Neues gelernt (rektale Anwendung von Cannabis), ein Experte habe gesprochen und die Fakten wären gut, einzig die Angabe zur Wirkdauer bzw. Absorptionsgeschwindigkeit bei oralem Cannabiskonsum wurde bemängelt. Die Vermittlung der Informationen wurde als theoretisch und auf hohem Niveau empfunden. Die gleichförmige Sprechweise, die benutzen Fremdwörter und die Folien mit Plus- und Minuszeichen forderte (zu) viel Aufmerksamkeit. Die Befragten wünschten sich für das Video die Umgangssprache, Cannabiserfahrung und mehr Leidenschaft für das Thema.
Das ***Produktevideo*** zum Vaporizer Wolkenkraft von VITA wurde heterogen kommentiert. Worin sich die Teilnehmenden einige waren, war der Fakt, dass die erwarteten Produkteinformationen im Video nur teilweise genannt wurden. Die Werbung von Fourtwenty wurde nicht als störend empfunden. Allerdings kam die Frage auf, ob eine Universität in einer offiziellen Studie für einen lokalen Anbieter und bestimmte Produkte Werbung machen dürfe.

Die Anwesenden sind motiviert sich durch die ***Anmeldeformulare*** zu klicken und dazu eine schriftliche Rückmeldung zu geben sowie an zukünftiger ***Medienarbeit*** mitzuwirken.

Die nächste und letzte Begleitgruppensitzung ist für den Februar 2024 vorgesehen.
.

6 Anhang 1: Frageroute Gruppendiskussion

Vorbereitung Begleitgruppe 16. Nov. 2023 18:30 – 20:30

Begrüssung, Agenda, Dauer (5`)

Informationen aus dem Forschungsteam (5`)

- Einige TN beginnen im Januar 2024 mit den Baseline Visiten und geben dazu eine Rückmeldung.
- Die Umsetzung der Studie SCRIPT beginnt *voraussichtlich* Ende Januar 2024 mit dem Verkauf von Cannabis in Apotheken.
- Es wird demnächst eine Medienmitteilung zur Studie geben. Ihr erhaltet eine Mail, wenn diese Medienmitteilung gemacht wird. Gleichzeitig wird darüber informiert, wie die Anmeldung abläuft. Bitte meldet euch offiziell an. Ihr werdet anschliessend manuell aus den Anmeldungen rausgesucht, da ihr unabhängig vom Wohnort mitmachen könnt.

Safer use rules →ppt zeigen und wirken lassen (25`)

Bitte nehmt euch Zeit und lest diese Regeln einmal durch.

- Wie wirken diese Regeln auf euch?
  Neutral, bevormundend, Leserschaft ist in der Verantwortung, andere wissen was gut für mich ist, …
- Was lest ihr daraus? Was nehmt ihr mit?
  - Was ist für euch die wichtigste Botschaft?
- Was sagt ihr zu den Inhalten?
  - Wirken die Inhalte vollständig?
  - Fehlt etwas?
    - Wenn ja, was?
- Wie findet ihr die Grafik?
  - Welche Informationen lest ihr daraus?
    - Ist diese Grafik verständlich?
  - Ist diese Grafik ansprechend?

Video von Bartek zu Toxikologie, Bioverfügbarkeit, Absorptionsgeschwindigkeit (40`)

Video abspielen (9`)

- Wie wirkt dieses Video auf euch?
- Was nehmt ihr mit?
- Was habt ihr verstanden?

    - Was versteht ihr unter Bioverfügbarkeit?
    - Was versteht ihr unter Toxikologie?
    - Was versteht ihr unter Absorptionsgeschwindigkeit
- Welche Elemente versteht ihr nicht?
        - Warum ist ein Filter nicht genügend?
- Gibt es Elemente/ Informationen, die ihr hier ergänzen möchtet?
    - Wenn ja, welche?
    - Bitte begründe deine Antwort.

Videos zu Vaporizer von Fourtwenty (30`)

Bevor ich euch Videos zeige, in denen Vaporizer und ihre Handhabung vorgestellt werden, fragen ich euch:

- **Was erwartet ihr von Produktevideos?**
    - Zu welchen Aspekten eines Vaporizers wollt ihr in einem Produktevideo etwas erfahren?
    - Worauf soll das Video fokussiert sein? Was darf auf keinen Fall fehlen?
    - Wann sind Produktevideos für euch als hilfreich?
    - Bei welchen Gelegenheiten schaut ihr euch Produktevideos an?

Ich zeige Video von Fourtwenty zu Vaporisieren (5`) und zu Wolkenkraft (3`)

- **Wie findet ihr diese Videos generell?**
  verständlich, ansprechend, informativ, hilfreich, überladen,

- **Wirken diese Videos authentisch?**
    - ***Falls ja***, was macht es aus, dass sie authentisch wirken?
    - ***Falls nein***, was fehlt, um authentisch zu wirken? An was macht ihr fest, dass «gespielt» wird?
- **Was gefällt euch besonders? Was fällt euch positiv auf?**
- **Seht ihr Nachteile, wenn Vaporizer mit solchen (Werbe-)Videos vorgestellt werden**?
    - Wenn ja, welche?
- **Seht ihr Vorteile, wenn Vaporizer mit solchen (Werbe-)Videos vorgestellt werden?**
    - Wenn ja, welche?

Falls die TN keine Aussagen zur Werbung machen, aktiv nachfragen:

- **Wie steht ihr zur Werbung in den Videos?**
    - Wie wirkt diese Werbung?
    - Findet ihr Werbung in den Videos ok?

        - Wenn ja, weshalb?
        - Wenn nein, weshalb?
    - Sollten diese Videos neutral gehalten werden?
        - Falls ja, weshalb?

Wer von euch hat Interesse an Medienarbeit? (5`)

- F. hatte bereits einen ersten Kontakt und kann berichten.
- Gerne kann eine 2. Person mit machen.

Wer von euch hat Interesse die online Anmeldung mit allen Fragen zu testen? (5`) Das bedeutet:

- Alle Fragen durchzuklicken und in einem separaten Feedbackformular eine Rückmeldung pro Seite/ Fragenset zu geben.
- Die Fragen können fiktiv beantwortet werden = Test. Die Daten werden anschliessend vernichtet.
- Dauer: ca. 30 Minuten
- Wer die online Fragen ausfüllt und den Feedbackbogen ausgefüllt zurück schickt erhält eine 1 Stunde à CHF 30.- gut geschrieben (wird mit PBG Geld ausbezahlt).
- Dieses Prozedere müsst ihr bei der Anmeldung nochmals wiederholen.

Ich werde eure Mailadresse an Kathrin Bieri weitergeben.

- Link zu den Fragen und das Feedbackformular erhaltet ihr in den nächsten 1-2 Wochen von Kathrin Bieri per Mail.

Nächste und letzte Sitzung *findet im Februar 2024 statt. (5`)*

Voraussichtliche Themen werden sein:

- Eine Retrospektive dazu, was ihr alles ins Studienprotokoll eingebracht habt.
- Einige Gedanken dazu, wie die Arbeit in der Begleitgruppe für euch war.
- Erste Rückmeldungen zu Studien- und Verkaufsabläufen.

7 Anhang 2: Safer Use Rules mit stehender Grafik

7 Empfehlungen für einen sicheren Umgang mit Cannabis

1 Wahl einer weniger schädlichen Konsumform von Cannabis

Die verschiedenen Formen des Cannabiskonsums lösen unterschiedliche Mengen und Arten von Schadstoffen aus.

- Joint, mit Tabak: Rauchen ist die schädlichste Cannabis-Konsumform. Tabak enthält in sich toxische Substanzen, die zu gesundheitlichen Schäden führen. Zudem hat das im Tabak enthaltene Nikotin ein starkes Abhängigkeitspotential.
- Joint, mit Filter: Der Filter bietet nur einen minimalen (Pseudo-)Schutz. Die meisten toxischen Substanzen gelangen durch den Filter hindurch in die Lunge.
- Vaporisator: Harz/Blüten werden um 200°C erhitzt. Es werden weniger Schadstoffe gelöst als beim Verbrennungsprozess in klassischen Joints.
- E-Joint: Mit einem E-Joint wird cannabishaltige Flüssigkeit um 200°C erhitzt. Es werden weniger Schadstoffe gelöst als beim Verbrennungsprozess in klassischen Joints.
- Orale Einnahme: Cannabis wird in unveränderter Form im Körper verabreicht. Schadstoffe durch Erhitzung von Cannabis werden vermieden.

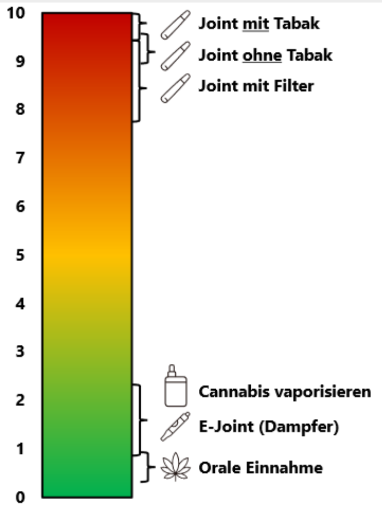

2 Tabak vermeiden

- Tabakrauch - nicht Nikotin - ist die wichtigste vermeidbare Ursache von Erkrankungen bei Personen, die Cannabis konsumieren.
- Falls Sie weiterhin Nikotin konsumieren möchten, wechseln Sie zu weniger schädlichen Konsumformen. (Nikotinersatztherapien, E-Dampfer, Nikotin Pouches)
- Lassen Sie sich in Ihrer SCRIPT Verkaufsstelle zu Tabakkonsum beraten.

3 Überdosierungen vermeiden

- Die Dauer bis die Wirkung von Cannabis gespürt wird, ist abhängig von der Konsumform.
- Cannabiskonsum durch Inhalieren führt zu einer raschen Aufnahme der Wirkstoffe über die Lunge.
- Bei oraler Einnahme ist die Wirkung verzögert und abhängig von Mahlzeiten und Tageszeit.
- Konsumieren Sie kleine Mengen und machen Sie Pause vor weiterem Konsum.
- Empfohlene Pausendauer: wenige Minuten bei inhalierter Form, bis eine Stunde bei oraler Einnahme.

4 Cannabis in der Freizeit konsumieren

- Cannabis beeinträchtigt Merkfähigkeit und psychomotorische Fähigkeiten.
- Beachten Sie die Wirkdauer von Cannabis, insbesondere vor Arbeit, Schule, Teilnahme am Strassenverkehr und Bedienen von Maschinen.
- Konsumieren Sie in einer Umgebung, in der Sie sich wohl fühlen.

5 Vermeiden von Cannabis in Kombination mit anderen psychoaktiven Substanzen und Medikamenten

- Der gleichzeitige Konsum von Cannabis und Substanzen wie z.B. Alkohol oder Drogen kann die Wirkung gegenseitig verstärken.
- Cannabis kann die Wirkung von Medikamenten beeinflussen. Lassen Sie sich durch eine Gesundheitsfachperson beraten, falls Sie Medikamente einnehmen.

6 Umfeld schützen, insbesondere Minderjährige

- Geben Sie Cannabis nicht an Dritte weiter.
- Lagern Sie Cannabis ausser Reichweite von Kindern, an einem kühlen, trockenen und luftdichten Ort.
- Schützen Sie Ihr Umfeld vor Passiv-Rauchen.

7 Hilfe holen bei Problem im Zusammenhang mit Cannabiskonsum

- Holen Sie sich Unterstützung, wenn Sie das Gefühl haben Ihr Cannabiskonsum gerate ausser Kontrolle.
- Holen Sie sich Hilfe, wenn Sie Entzugserscheinungen haben oder wenn Ihr Konsum die Arbeit, Schule oder das Sozial- und Familienleben beeinträchtigt.

Unterstützungs-angebote

www.safezone.ch
Online-Plattform für anonyme Suchtberatung

www.infodrog.ch
Schweizerische Koordinations- und Fachstelle

www.bernergesundheit.ch
Lokale Gesundheitsfachstelle Bern / Biel

www.stopsmoking.ch
Online-Plattform für Rauchstopp

SCRIPT Verkaufsstelle
Persönliche Tabak- und Cannabiskonsumberatung in Ihrer Apotheke

Studienkontakt:
info@script-studie.ch
031 684 67 79 (Mo-Fr 8-17);
Notruf (24h): 112
Prof. Dr. med. Reto Auer
Berner Institut für Hausarztmedizin (BIHAM)
Universität Bern
Mittelstrasse 43
3012 Bern

SCRIPT

Safer Use Regeln | SCRIPT Cannabispilotprojekt | DE | V 1.2 | 07.11.2023

8 Anhang 3: Safer Use Rules mit liegender Grafik

Safer Use Regeln | SCRIPT Cannabispilotprojekt | DE | V 1.2 | 07.11.2023

7 Empfehlungen für einen sicheren Umgang mit Cannabis

1 Wahl einer weniger schädlichen Konsumform von Cannabis

Die verschiedenen Formen des Cannabiskonsums lösen unterschiedliche Mengen und Arten von Schadstoffen aus.

- Joint, mit Tabak: Rauchen ist die schädlichste Cannabis-Konsumform. Tabak enthält in sich toxische Substanzen, die zu gesundheitlichen Schäden führen. Zudem hat das im Tabak enthaltene Nikotin ein starkes Abhängigkeitspotential.
- Joint, mit Filter: Der Filter bietet nur einen minimalen (Pseudo-)Schutz. Die meisten toxischen Substanzen gelangen durch den Filter hindurch in die Lunge.
- Vaporisator: Harz/Blüten werden um 200°C erhitzt. Es werden weniger Schadstoffe gelöst als beim Verbrennungsprozess in klassischen Joints.
- E-Joint: Mit einem E-Joint wird cannabishaltige Flüssigkeit um 200°C erhitzt. Es werden weniger Schadstoffe gelöst als beim Verbrennungsprozess in klassischen Joints.
- Orale Einnahme: Cannabis wird in unveränderter Form im Körper verabreicht. Schadstoffe durch Erhitzung von Cannabis werden vermieden.

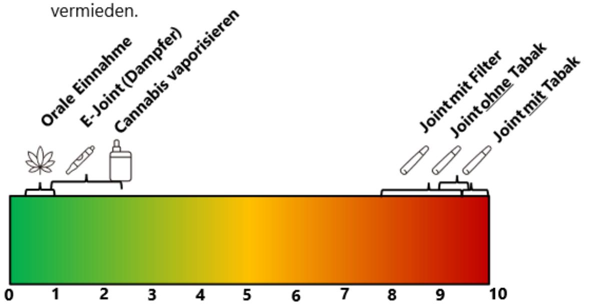

2 Tabak vermeiden

- Tabakrauch - nicht Nikotin - ist die wichtigste vermeidbare Ursache von Erkrankungen bei Personen, die Cannabis konsumieren.
- Falls Sie weiterhin Nikotin konsumieren möchten, wechseln Sie zu weniger schädlichen Konsumformen. (Nikotinersatztherapien, E-Dampfer, Nikotin Pouches)
- Lassen Sie sich in Ihrer SCRIPT Verkaufsstelle zu Tabakkonsum beraten.

3 Überdosierungen vermeiden

- Die Dauer bis die Wirkung von Cannabis gespürt wird, ist abhängig von der Konsumform.
- Cannabiskonsum durch Inhalieren führt zu einer raschen Aufnahme der Wirkstoffe über die Lunge.
- Bei oraler Einnahme ist die Wirkung verzögert und abhängig von Mahlzeiten und Tageszeit.
- Konsumieren Sie kleine Mengen und machen Sie Pause vor weiterem Konsum.
- Empfohlene Pausendauer: wenige Minuten bei inhalierter Form, bis eine Stunde bei oraler Einnahme.

4 Cannabis in der Freizeit konsumieren

- Cannabis beeinträchtigt Merkfähigkeit und psychomotorische Fähigkeiten.
- Beachten Sie die Wirkdauer von Cannabis, insbesondere vor Arbeit, Schule, Teilnahme am Strassenverkehr und Bedienen von Maschinen.
- Konsumieren Sie in einer Umgebung, in der Sie sich wohl fühlen.

5 Vermeiden von Cannabis in Kombination mit anderen psychoaktiven Substanzen und Medikamenten

- Der gleichzeitige Konsum von Cannabis und Substanzen wie z.B. Alkohol oder Drogen kann die Wirkung gegenseitig verstärken.
- Cannabis kann die Wirkung von Medikamenten beeinflussen. Lassen Sie sich durch eine Gesundheitsfachperson beraten, falls Sie Medikamente einnehmen.

6 Umfeld schützen, insbesondere Minderjährige

- Geben Sie Cannabis nicht an Dritte weiter.
- Lagern Sie Cannabis ausser Reichweite von Kindern, an einem kühlen, trockenen und luftdichten Ort.
- Schützen Sie Ihr Umfeld vor Passiv-Rauchen.

7 Hilfe holen bei Problem im Zusammenhang mit Cannabiskonsum

- Holen Sie sich Unterstützung, wenn Sie das Gefühl haben Ihr Cannabiskonsum gerate ausser Kontrolle.
- Holen Sie sich Hilfe, wenn Sie Entzugserscheinungen haben oder wenn Ihr Konsum die Arbeit, Schule oder das Sozial- und Familienleben beeinträchtigt.

Unterstützungs-angebote

www.safezone.ch

Online-Plattform für anonyme Suchtberatung

www.infodrog.ch

Schweizerische Koordinations- und Fachstelle

www.bernergesundheit.ch

Lokale Gesundheitsfachstelle Bern / Biel

www.stopsmoking.ch

Online-Plattform für Rauchstopp

SCRIPT Verkaufsstelle

Persönliche Tabak- und Cannabiskonsumberatung in Ihrer Apotheke

Studienkontakt:
info@script-studie.ch
031 684 67 79 (Mo-Fr 8-17);
Notruf (24h): 112
Prof. Dr. med. Reto Auer
Berner Institut für Hausarztmedizin (BIHAM)
Universität Bern
Mittelstrasse 43
3012 Bern

Auswertung der Gruppendiskussion der partizipativen Begleitgruppe während der Aufbauphase der SCRIPT Studie

Die Gruppendiskussion wurde mit cannabiskonsumierenden Personen geführt

SCRIPT

The Safer Cannabis – Research In Pharmacies randomized controlled Trial

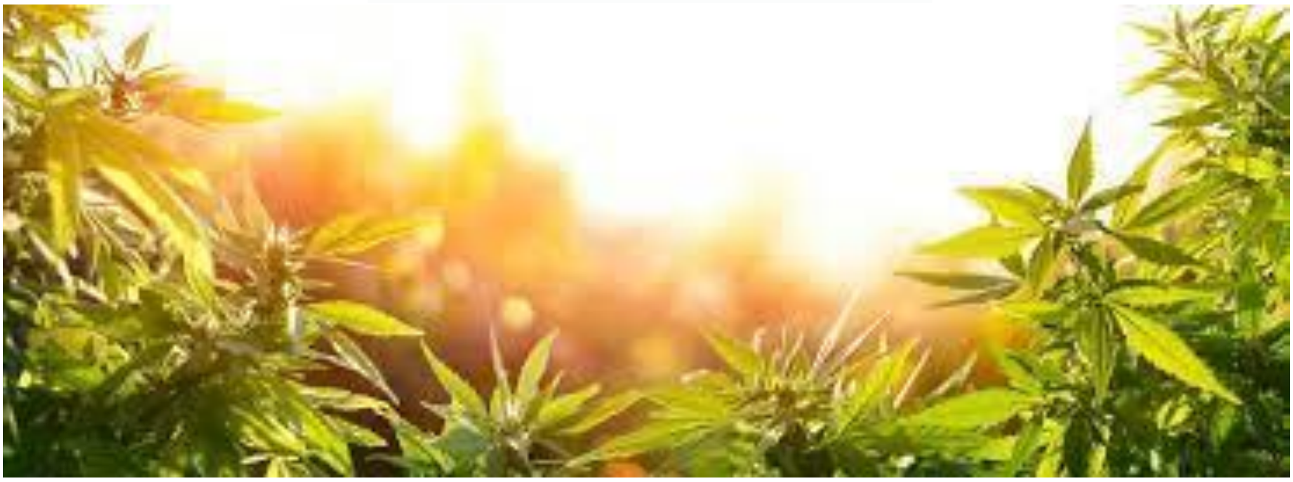

Beatrice Metry

Berner Institut für Hausarztmedizin

März 2024

Inhaltsverzeichnis

1 Einleitung 3
2 Methodisches Vorgehen 4
2.1 Zuständigkeitsabklärung bei der kantonalen Ethikkommission Bern 4
2.2 Erhebungs- und Auswertungsverfahren 4
2.3 Stichprobe 4
3 Ergebnisse aus der Gruppendiskussion 6
3.1 Mitschreiben an einem Artikel zur Arbeit in der Begleitgruppe 6
3.2 Cannabisprodukte 6
3.2.1 Rückmeldungen zu den Cannabisprodukten 7
3.2.2 Rückmeldungen zu den Cannabiskonsum-Geräten 8
3.3 Erkenntnisse aus der Begleitgruppe für die Forschungsgruppe 9
3.4 SCRIPT Intranet 11
3.5 Rückblick auf die Arbeit in der Begleitgruppe 13
3.6 Diverses 15
4 Fazit 16
5 Anhang 1: Frageroute Gruppendiskussion 18
6 Anhang 2: Erkenntnisse aus der Begleitgruppe 20

1 Einleitung

Im vierten Quartal vom Jahr 2021 wurde mit den neun Mitgliedern der partizipative Begleitgruppe der SCRIPT Studie Einzelinterviews geführt. Im Juli 2022, im Februar, Juni und November 2023 sowie im März 2024 traf sich die Gruppe je einmal zur Gruppendiskussion in den Räumlichkeiten der Universität Bern. Die Einzelinterviews wie auch diese Gruppendiskussionen wurden inhaltsanalytisch ausgewertet und die Resultate in einem Bericht dargestellt.
Bis dato sind folgende Berichte entstanden und erhältlich:

- Auswertung der Einzelinterviews zu SCRIPT vom März 2022
- Auswertung der Gruppendiskussion zu SCRIPT vom Juli 2022
- Auswertung der Gruppendiskussion zu SCRIPT vom März 2023
- Auswertung der Gruppendiskussion zu SCRIPT vom Juni 2023
- Auswertung der Gruppendiskussion zu SCRIPT vom November 2023
- Auswertung der Gruppendiskussion zu SCRIPT vom März 2024 (vorliegender Bericht)

Die partizipativen Begleitgruppe der SCRIPT-Studie traf sich am 6. März 2024 zur fünften Gruppendiskussion. Von den anfänglich neun Mitglieder der partizipativen Begleitgruppen, waren diesmal sieben cannabiskonsumierende Personen anwesend, die sich unter der Moderation von Beatrice Metry (wissenschaftliche Mitarbeiterin) zu folgenden Themen austauschte:

- Mitarbeit an einem Artikel über die Arbeit in der Begleitgruppe
- Cannabisprodukte
- Erkenntnisse aus der Begleitgruppe für die Forschungsgruppe
- SCRIPT Intranet für Studienteilnehmer*innen
- Rückblick auf die Arbeit in der Begleitgruppe

In diesem Auswertungsbericht wird die Stichprobe skizziert und anschliessend die Ergebnisse den Themen entlang der Frageroute, welche im Anhang zu finden ist, vorgestellt. Im Anhang findet sich die Frageroute.

2 Methodisches Vorgehen

Nachfolgend werden die verschiedenen Teile des Vorgehens beschrieben. Alle befragten Personen haben vor dem Einzelinterinterview (2021) einen Informed consent unterzeichnet und sich durch ihre Unterschrift mit dem Interview sowie den nachfolgenden Gruppendiskussion und deren Auswertung einverstanden erklärt. Darin wurde ihnen Anonymität zugesichert.

2.1 Zuständigkeitsabklärung bei der kantonalen Ethikkommission Bern

Am 31. Mai 2021 wurde das Konzept zur qualitativen Begleitforschung mit dem Titel «Einsatz einer partizipativen Begleitgruppe bestehend aus Cannabiskonsumierenden Erwachsenen als Ergänzung während der Planungsphase des Projekts SCRIPT 2» bei der Kantonalen Ethikkommission (KEK) eingereicht, um deren Zuständigkeit zu prüfen. Die KEK befand, dass sie nicht zuständig sei. Dies bedeutet, dass diese Forschung nicht unter das Humangesetz Artikel 2, Absatz 1 fällt und Einzel- und Gruppeninterviews mit der ausgewählten Zielgruppe durchgeführt werden können, ohne ein Gesuch einzureichen. Nach diesem Bescheid[1] vom 20. Juni 2021 wurde mit der Akquise von Teilnehmenden gestartet.

2.2 Erhebungs- und Auswertungsverfahren

Die Daten wurden durch eine Gruppendiskussion entlang einer definierten Frageroute, während einem Zeitraum von zwei Stunden, durchgeführt und digital aufgezeichnet sowie anschliessend transkribiert.

Das Transkript wurde mittels der Software MAXQDA inhaltsanalytische ausgewertet. Das bedeutet in einem ersten Schritt die Codierung des Textes, anschliessend eine Verdichtung der einzelnen Aussagen sowie das Verschriftlichen der Ergebnisse.

2.3 Stichprobe

Für die Gruppendiskussion wurden acht Personen, welche bereits bei den Einzelinterviews mitgemacht und den entsprechenden Informed consent unterzeichnet haben, angefragt. Via nuudel wurde der Termin vereinbart. Eine Person hat sich aus der Begleitgruppe zurückgezogen, da sie kein Cannabis mehr konsumiert.

Sieben Personen hatten sich für diese Gruppendiskussion angemeldet und sind zur Gruppendiskussion erschienen. Die nachfolgende Tabelle gibt eine Übersicht der Stichprobe während den Einzelinterviews und den fünf Begleitgruppensitzungen.

[1] BASEC-Nr Req-2021-00609, Bescheid der Zuständigkeitsabklärung liegt vom 20.6.2021 vor

Tabelle 1 Übersicht der Stichprobe in den verschiedenen Erhebungsmomenten

	**Einzelinterviews 4. Quartal 2021**	**Gruppen-diskussion 1 Juli 2022**	**Gruppen-diskussion 2 März 2023**	**Gruppen-diskussion 3 Juni 2023**	**Gruppen-diskussion 4 Nov. 2023**	**Gruppen-diskussion 5 März 2024**
**Anzahl Personen**	9	6	5	4	5	7
**Jahrgang**	1957, 1975, 1990, 1991, 1993, 1999, 2000, 2001, 2000	1957, 1975, 1991, 1993, 2001, 2002	1957, 1991, 1993, 2000, 2002	1957, 1975, 2000, 2002	1957, 1975, 1991, 1993, 2000,	1957, 1975, 1990, 1991, 1993, 2000, 2002,
**Branche Beruf**	Studium (3)<br>Logistik (2)<br>Finanzen (1)<br>Fotografie (1)<br>Grafik (1)<br>Fitness (1)	Studium (1)<br>Logistik (1)<br>Finanzen (1)<br>Fotografie (1)<br>Grafik (1)<br>Fitness (1)	Studium (2)<br>Finanzen (1)<br>Grafik (1)<br>Fitness (1)	Studium (2)<br>Fotografie (1)<br>Grafik (1)	Studium (1)<br>Finanzen (1)<br>Fotografie (1)<br>Grafik (1)<br>Fitness (1)	Studium (2)<br>Logistik (1)<br>Finanzen (1)<br>Fotografie (1)<br>Grafik (1)<br>Fitness (1)
**Anrede**	Frau 3<br>Herr 5<br>non-binär 1	Frau 2<br>Herr 3<br>non-binär 1	Frau 2<br>Herr 3<br>non-binär 0	Frau 1<br>Herr 2<br>non-binär 1	Frau 1<br>Herr 3<br>non-binär 1	Frau 2<br>Herr 4<br>non-binär 1
**Nationalität**	Schweiz 9	Schweiz 6	Schweiz 5	Schweiz 4	Schweiz 5	Schweiz 7

3 Ergebnisse aus der Gruppendiskussion

Die Ergebnisse werden nachfolgend entlang der Frageroute aufgeführt. Zahlen in runden Klammern weisen darauf hin, wie viele der Befragten in diese Richtung geantwortet haben. Befindet sich keine Klammer hinter der Aussage, handelt es sich um eine Einzelnennung. In eckigen Klammern wird der Absatz in der MAXQDA-Datei (z.B. [A-56]) in welchem das genannte Zitat gefunden werden kann, aufgeführt.
Die anwesenden Personen wurden begrüsst und die Agenda vorgestellt. Die Agenda beinhaltete folgende sechs Punkte: Anna Schibli vorstellen (Doktorandin und SCRIPT-Studienärztin), Artikel zur Arbeit in der Begleitgruppe mitschreiben, Cannabisprodukte, Erkenntnisse aus der Begleitgruppenarbeit, SCRIPT-Intranet und Rückblick auf die Begleitgruppen(zusammen-)arbeit.

3.1 Mitschreiben an einem Artikel zur Arbeit in der Begleitgruppe

Anna Schibli, SCRIPT-Studienärztin und Doktorandin, stellte sich der Begleitgruppe vor. Sie befasst sich in ihrer Dissertation mit folgender Frage: Wie stark beeinflusste die Begleitgruppe mit ihren Inputs das Studienprotokoll bzw. die Intervention? Sie fragt die Begleitgruppenmitglieder, ob es für sie in Ordnung sei, wenn sie über dieses Thema – die Arbeit der Anwesenden – schreibe. Die Begleitgruppenmitglieder äusserten damit einverstanden zu sein. Anna Schibli fragte weiter, ob ein bis zwei Personen bereit wären am Artikel mitzuschreiben. Es meldeten sich zwei Personen zum aktiven Mitschreiben. Anna Schibli führte weiter aus, dass es möglich sei als Mitautor*in auf dem wissenschaftlichen Artikel aufgeführt zu werden oder anonym zu bleiben und lediglich als stille Rückmelder*innen zu wirken. Nebst den beiden Interessierten melden sich nun noch zwei Personen, die als Reserve fungieren werden sowie zwei weitere Personen, die den Artikel vorgängig lesen möchten und allenfalls eine Rückmeldung dazu geben werden.

Eine erste Version stellte Anna Schibli für den Mai/ Juni 2024 in Aussicht. Der Zeitaufwand benannte sie mit ein bis zwei Arbeitstagen, doch könne das Engagement individuell bestimmt werden. Als Entgelt werden die Teilnehmer*innen CHF 30.- pro geleistete Stunde erhalten. Alle anwesenden Begleitgruppenmitglieder werden von Anna Schibli den Artikel erhalten.

3.2 Cannabisprodukte

Die anwesenden Personen durften die Cannabisprodukte näher betrachten. Das bedeutete, sie erhielt die Cannabisprodukte in der offiziellen Verkaufsverpackung sowie das ausgepackte Cannabisprodukt in einem Glas. Dieses Gläser haben eine integrierte Lupe und die Gläser, in denen Blüten gezeigt wurden, hatten zusätzlich eine Lasche, die geöffnet werden kann, um das Produkt auch riechen zu können. Es wurden drei Cannabisblüten und zwei Haschischprodukte gezeigt. Zur gleichen Zeit wurden den Begleitgruppenmitglieder drei Vaporizer und einen E-Joint vorgestellt und

zum näheren Betrachten in die Runde gegeben. Anschliessend wurden die Produkte wieder eingesammelt. Erst danach wurde über diese Produkte gesprochen.

3.2.1 Rückmeldungen zu den Cannabisprodukten

In einer ersten Runde brachten die anwesenden Personen (7) ihre Begeisterung vom ***Cannabisangebot*** zum Ausdruck. Fünf Personen äusserten, sie fänden das Produkte-Demo-Glas mit Lupe und Riechlasche super, wie mit nachfolgendem Zitat untermalt wird:

> Das sieht richtig professionell aus. Die Verpackung ist so ähnlich wie andere CBD-Produkte. Das Produkt selbst sieht super aus. Auch mit dieser Lupe, das finde ich super, zum Reinschauen. [A-86]

Gut die Hälfte der anwesenden Personen äusserte, die Produkte sähen ansprechen aus (4). Es gab weitere Einzelstimmen. So merkte eine Person an, bei ihr käme ein VIP-Gefühl auf, weil sie als Begleitgruppenmitglieder nun diese Cannabisprodukte vor allen anderen Studienteilnehmer*innen zu Gesicht bekämen. Die Gegebenheiten, dass die Cannabisprodukte aus einer Outdoor-Produktion kämen und in der Schweiz produziert würden, sowie die Auswahl der Cannabisprodukte kamen ebenfalls gut an.

Drei Personen betonten, dass die ***Blüten*** edel aussähen und es sich offensichtlich um eine gute Produktion handle. Nachfolgendes Zitat unterstreicht diese Aussage.

> Es [Anmerkung der Autorin: die Blüte] hat wirklich edel und schön ausgesehen. […] Ich würde das gerne am Kiosk kaufen. [A-138]

Eine Person merkte an, der intensive Lemonduft des einen Produktes sage ihr nicht zu.

Die ***Haschisch***-Produkte würden wie gepresster Blütenstaub aussehen, waren sich drei Personen einig. Dies wurde wie folgt formuliert:

> Eben, das ist eigentlich kein Hasch, das ist gepresster Blütenstaub. [A-133]

> Das sieht schon so aus, als wäre es gepresster Blütenstaub, definitiv. [A-136]

Eine Stimme merkte an, dass den Produkte-Demo-Gläser für die Haschisch-Produkte die Riechlasche fehle, wie nachfolgendes Zitat belegt:

> Das mit dem Hasch finde ich immer noch, dass man schauen könnte, ob man nicht auch da eine Lasche haben könnte, damit man auch noch daran riechen könnte. [A-88]

Die ***Verpackung*** wurde von drei Personen als ansprechend bzw. professionell beschrieben. Das nachfolgende Zitat belegt diese Aussage:

> Ich finde es schön, dass auf den Plastikverpackungen keine schlimmen Bilder drauf sind, wie wir das von den Zigarettenverpackungen kennen. Das wäre möglich gewesen, dass

> das hier auch in dieser Art aussehen könnte. Aber, es ist nice, dass es so clean aussieht. [A-87]

Zwei Personen monierten den Link hinter dem ***QR-Code,*** der auf der Verpackung aufgedruckt ist. Dieser führe zur offiziellen SCRIPT-Studien-Website. Für die Konsument*innen wäre es sinnvoll, wenn der QR-Code direkt zur Produktebeschreibung führen würde. Eine andere Person äusserte, die Angaben auf den Verpackungen gut zu finden.

Die ***Preise*** der Cannabisprodukte wurde generell als hoch empfunden (4). Eine Person entgegnete, dass die Produktequalität und -sicherheit der angebotenen Studien-Cannabisprodukte ihren Preis habe. Dies untermalte eine andere Person mit ihrer aktuellen Erfahrung auf dem Schwarzmarkt, wie folgendes Zitat belegt:

> Ja, das ist schon so. Und inzwischen ist es auch gefährlich mit dem ganzen Sprayshit, der angeboten wird. Also [ich] habe selbst auch schon davon erwischt und das ist gar nicht lustig. Diese synthetischen Cannabinoiden, die sie haben. Da nehmen sie CBD in China, grosse Felder, wo sie mit der Spritzpistole drüber gehen. Und das ist gefährlich. Auf der einen Blüte ist viel [THC] drauf und auf der anderen Blüte nebendran fast nichts. [A-116]

Zwei Personen gaben an, auch zukünftig bei ihren Bekannten Cannabisprodukte zu kaufen, insbesondere weil es günstiger und auch von guter Qualität sei.

Zwei Personen äusserten, die Preislegung sei widersprüchlich und verleite Kund*innen dazu, die Produkte mit dem höchsten THC-Gehalt zu kaufen, weil da die THC-Einheit am günstigsten sei.

+ airzier air max

3.2.2 Rückmeldungen zu den Cannabiskonsum-Geräten

Nebst den Cannabisprodukten hatten die anwesenden Personen die Möglichkeit, zwei Vaporizer (Wolkenkraft Vita und Volcano Mighty) und einen E-Joint (OBY Aspire) zu begutachten.

Vier Personen gaben an, bereits Erfahrung mit Vaporizern zu haben und selbst ein Gerät zu besitzen. Drei dieser vier Personen äusserten, einen Volcano Mighty zu besitzen und zwei dieser Personen nutzen ihn regelmässig. Eine dieser Personen äusserte sich wie folgt:

> Ja, ich brauche ihn oft. […] Also Joint rauchen finde ich etwas vom schönsten. Aber zu viel Rauchen habe ich dann mit der Zeit auf der Lunge bemerkt. Und ja, mittlerweile habe ich so aufgerüstet, dass ich zu Hause einen Volcano habe, also ein Supergerät, aber das ist an zu Hause gebunden, also an den Strom. Und für unterwegs habe ich den Venti, dass wenn ich an einem Ort hingehe, dass ich den brauchen kann. [A-167]

Eine Person äusserte zu Beginn der Sitzung Skepsis gegenüber Vaporizern. Während dem aktiven Betrachten der Geräte und Austausch mit den anderen Begleitgruppenmitglieder änderte diese Person ihre Meinung, was in nachfolgendem Zitat zur Geltung kommt.

> Weil, das letzte Mal, das weiss ich noch, da habe ich mich lustig gemacht über diese Vaporizer. Weil, mich regt es auf, dass es mit dem Strom verbunden ist und es immer laden musst. Aber nun dieser kleine da, den Wolkenkraft Vita mit Holz. Das war jetzt hier meine Aussage: Aha, das ist jetzt wohl das nächste, dass ich mich einmal mit diesen [Vaporizern] auseinandersetzen muss und dann vielleicht diesen Wolkenkraft mal (ausprobiere). Und dieses grosse Gerät [Volcano Mighty], also, ich verstehe was du [anderes Begleitgruppenmitglied] erzählst. Und einerseits gluschtet es mich. Aber ich sage jetzt mal, für das urchige Kiffer-Gefühl, ist mir das schon fast eben...// Wenn ich so mit dir [anderes Begleitgruppenmitglied] spreche, dann ist mir das schon ein bisschen zu Highend. Ich sehe den Sinn, weshalb man das macht. Aber für mein Kiffer-Ritual, wenn ich zuerst ein Gerät laden muss und programmieren...// Das ist jetzt halt vielleicht die moderne Welt, wie man heute die Gesundheit fördert. Aber so in meinem Kiffer-Dasein ist das noch nicht angekommen. Aber, ja. Aber mit diesem einen (Wolkenkraft) probiere ich dann einmal. [A-170]

Der E-Joint stösst bei einigen anwesenden Personen (3) auf Skepsis. Eine Person äusserte, davon abzusehen einen E-Joint zu probieren, da dies billige Wegwerfprodukte seien. Da konterte eine andere Person. Lediglich das Coil werde ausgewechselt und das Liquid aufgefüllt (Pod wechseln oder nachfüllen). Dieselbe Person merkte an, mit den in der SCRIPT-Studie angebotenen Pods sei der/ die Konsument*in an dieses Gerät gebunden. Mit Liquids wie sie bei E-Zigaretten angeboten würden, könne der Pod selbst nachgefüllt werden, dies gäbe Produktefreiheit.

3.3 Erkenntnisse aus der Begleitgruppe für die Forschungsgruppe

Die Begleitgruppenmitglieder wurden gefragt, was sie denken, welche ihrer Rückmeldungen an die Forschungsgruppe wichtig waren. Oft machten sie die Wichtigkeit daran fest, ob ihr Input vom Forschungsteam aufgenommen und umgesetzt wurde. In einem ersten Schritt wurde mündlich gesammelt, was den anwesenden Personen aus dem Stegreif einfiel. In einem zweiten Schritt wurde ihnen eine Liste vorgelegt (6 Anhang 2) mit der Bitte in Einzelarbeit, die für sie fünf wichtigsten Aspekte anzukreuzen. Die Darstellung der Ergebnisse folgt in dieser Reihenfolge.

In der ***offenen Runde*** wurden viele Einzelnennungen aufgezählt.

Zum Verkauf und dem Verkaufsraum:

- Verkaufspersonal, die Fachkenntnisse haben (2).
- Der Verkaufsablauf in der Apotheke
- Ein separater Beratungsraum in der Apotheke, falls der/ die Kund*in, dies wünscht.
- Produkte-Demo-Glas, so wird riechen und betrachten der Produkte möglich

Zu den Produkten:

- Einfluss auf die Produkteauswahl

    - Haschisch wurde in die Produktpalette aufgenommen
    - Von Cannabis-Analzäpfchen wurde abgesehen
- Die Verpackung ist neutral gehalten.

Gefahrenübersicht:

- Der Aktivkohlefilter wurde aufgenommen

Einer Person äusserte, die inklusive Geschlechterform sei auf Anregung aus der Begleitgruppe in die Dokumente und die Texte der SCRIPT-Website aufgenommen worden, dies fiel positiv auf.

Die Ergebnisse aus der ***schriftlichen Einzelbefragung*** (s. Anhang 2) werden nachfolgend in der numerischen Reihenfolge dargestellt. Zusätzliche schriftliche Kommentare der befragten Personen, finden sich jeweils direkt beim aufgeführten Aspekt.

- Nicht nur Cannabisblüten, auch Haschisch in verschiedenen Sorten (5)
    - Kommentar: Gepresster Blütenstaub sei eher das, was hier angeboten werde.
- Verkaufsperson soll eine kompetente Beratung anbieten (fachkundige Auskunft, Liste mit Sorten, Preisen) (5)
    - Kommentar: Vermittelt Sicherheit; wenn möglich, sollte die Verkaufsperson die Produkte probiert haben
- Produkte sollen sichtbar und im besten Fall auch riechbar sein (4)
    - Kommentar: Auch Hasch soll riechbar sein.
- Auftreten der Fachperson (Sympathie, Ton) ist wichtig (4)
    - Kommentar: Vertrauen aufbauen ist wichtig.
- Mögliche Verkaufsoptionen abgesehen von Apotheken: Cannabis Social Clubs, CBD-Shops, Quartier-Bioladen, Tabakladen. (3)
- Transparenz auf Inhaltsstoffe, Sorte, Wirkung, THC-/ CBD-Gehalte. (2)
    - Kommentar: Die Beschreibung der Wirkung könnte besser sein.
- Cannabisverkauf durch Contact [Stiftung für Suchthilfe. Anmerkung der Autorin] nicht attraktiv, da stigmatisierend (2)
- Rauchstoppberatung nur auf Wunsch, unaufdringlich (2)
- Outdoor- und Indoor-Produkte in guter Qualität
- Nicht mehr als CHF 10.-/Gramm
- Bio-Qualität ansprechend, Preis von Blüten und Haschisch eher an der oberen Grenze.
    - Kommentar: Der Preis sei zu stark an der oberen Grenze. Der Preis sollte zwischen CHF 5 -10.-/ Gramm sein.
- Wichtig, dass jede*r aus der Forschungsgruppe mindestens einmal Cannabis geraucht hat.
- Mundspray und Cremes interessant, Zäpfchen nicht interessant
- Einverstanden mit der Einverständniserklärung

- Hausarzt/ -ärztin als Kontaktperson wird kritisch gesehen.
- Anpassung auf Safer Use Flyer: Orale Wirkzeit, Position der Grafik.

Der Aspekt «THC-Werte mit maximal 18% für die Blüten ausreichend» wurde mit folgendem Kommentar versehen: Tiefere [THC-Werte Anmerkung der Autorin] könnten auch gut sein.

3.4 SCRIPT Intranet

Das SCRIPT-Intranet für Studienteilnehmer*innen wurde von allen anwesenden Personen (7) als gut befunden. Ganz besonders positiv betonten die befragen Personen die ausklappbaren Empfehlungen für einen sicheren Umgang mit Cannabis (7). Die Leserschaft könne auf diese Weise selbst entscheiden zu welchem Thema sie mehr Informationen haben möchte (7). Die Seite sei übersichtlich strukturiert (7), verständlich und einfach aufgebaut (2), informativ (2), einladend, schön, sec, das waren weitere Worte, mit der das Intranet von den Begleitgruppenmitglieder beschrieben wurde. Ein Votum zum Intranet, welches die obigen Aussagen stützt:

> Ich finde es sehr übersichtlich, […]. Es ist einfach gemacht. Ich finde das gut. Ich finde das wirklich gut. Es ist einfach aufgebaut, man checkt sehr schnell was man wo findet. Man sieht die Produkte gut. Sie haben diese schön aufgelistet. [279]

Alle Informationen, die es brauche, seien vorhanden, äusserten zwei Personen.

Die Informationen unter dem Ritter ***Safer Use Empfehlungen*** gab am meisten zu Diskutieren. Der Aspekt «Wählen Sie eine weniger schädliche Konsumform», erfreute drei Personen, da darin der Aktivkohlefilter aufgenommen und dargestellt wurde. Eine Person monierte, dass die Aktivkohlefilter nicht besser dargestellt würden. Die Person äusserte, es wichtig zu finden, dass Cannabiskonsument*innen darauf aufmerksam gemacht würden, es sei gesünder mit einem anständigen Filter zu rauchen als ohne. Mit der aktuellen Darstellung unter den Safer Use Regeln vergebe sich die Studie die Chance, diesen Aspekt deutlich zu machen.

Generell wurde von den anwesenden Personen (7) betont, sich vertieftere Informationen zu den einzelnen Fragen oder Aspekten zu wünschen. Das Wort «Überdosierung» sei negativ behaftet, monierten drei Personen. Sie nannten keine Alternative zum Wort Überdosierung. Des Weiteren bemerkten die Befragten, unter dem Aspekt «Vermeiden Sie die Kombination von Cannabis mit anderen psychoaktiven Substanden und Alkohol» werde auf die psychoaktiven Substanzen gar nicht eingegangen (3). Nachfolgende Zitate verdeutlichen, was gemeint ist.

> Ich finde das nun noch spannend. Sie sprechen von psychoaktiven Substanzen und Medikamente. Aber auf die psychoaktiven Substanzen wird gar nicht eingegangen. [A-349]

> Das kann man sicher noch etwas ausschmücken, unter den jeweiligen Punkten. Sonst generell finde ich es einen guten Überblick und eine gute Einsicht. Es ist nicht überladen.

> Aber eben, wenn man das Plus öffnet, gerade für die psychoaktiven Substanzen, dass man das vielleicht auch die Drogen auflistet, die besonders .../ Oder eben auch Mischkonsum besonders schwierig sein kann, sei es mit Uppers und Downers. [A-351]

Die Informationen unter Mischkonsum sei deutlich zu knapp (4). Allein die Kombination von Cannabis und Alkohol (3) bedürfe mehr Informationen, da es auf die Reihenfolge des Konsums ankomme, wie nachstehendes Zitat zeigt:

> Also, wenn du so fragst, ich finde es eher sehr, sehr knappgehalten. Ich weiss auch nicht, aber zum Beispiel das mit dem Alkohol, das kann man noch ausführen. Ich finde, es ist immer ein grosser Unterschied, ob man zuerst Alkohol trinkt und dann noch eines raucht oder ob man eines geraucht hat und dann noch Alkohol trinkt. [A-333]

Dasselbe gelte für die Kombination Cannabis und Medikamente oder Drogen, fügten zwei weitere Stimmen hinzu. Auch hier fehle ein weiterer Aspekt nämlich: Was kann ich tun, wenn ich Mischkonsum hatte (2)? Bei diesen kargen Informationen sei die Wahrscheinlichkeit gross, dass sich die Menschen auf dem Internet orientieren würden. Das Votum, statt das SCRIPT-Intranet mit mehr Informationen zu füllen, auf passende Websites zu verweisen, fand bei allen anwesenden Personen Zustimmung. Als mögliche Seiten wurden eve & rave[2], Contact[3] und Saferparty[4] genannt.

Vier Personen äusserten, eine Ergänzung zur Überdosierung wäre hilfreich mit der Frage: «Was kann ich bei Überdosierung tun?» Und an dieser Stelle Verhaltensmöglichkeiten und Tipps aufführen, die bei einer Überdosierung helfen können. Von den Befragten wurden Orangensaft, Vitamin C, Duschen und frische Luft genannt. Eine Person erwähnte die Idee, zu diesem Aspekt Erfahrungsberichte von Betroffenen an dieser Stelle zu veröffentlichen. Eine andere Person meinte, der Titel müsste lauten: «Überdosierung bei oraler Einnahme vermeiden», da sich die beiden aufgeführten Aspekte lediglich auf die orale Einnahme beziehe. Darauf konterte eine weitere Person, das Feld könne auch geöffnet und mit Punkten für eine Überdosierung durch Kiffen ergänzt werden.

Eine Person äusserte, ihr fehle ein Button «Feedback an die Studienleitung», damit sie Beobachtungen deponieren könne. Ebenfalls fehle innerhalb des Intranets eine Möglichkeit Fragen zu stellen bzw. die Verlinkung zum Studienkontakt.

[2] https://www.eve-rave.org/drogen-abc/ oder https://eve-rave.ch/Forum/
[3] www.contact-suchthilfe.ch
[4] https://www.saferparty.ch/

3.5 Rückblick auf die Arbeit in der Begleitgruppe

Auf die Frage, wie es für die anwesenden Personen war, aktiver Teil einer Studie zu sein, gaben sechs Personen an, es sei eine interessante, gute ***Erfahrung*** gewesen, insbesondere zu sehen, dass sie als Kiffer Einfluss auf das Studiendesign hätten, wie nachfolgende Zitate belegt:

> Ich hätte nie gedacht, dass ich einmal so etwas machen würde. Wirklich nicht. Never. Auch mit dem Gedanken: Ich weiss ja gar nicht was ICH dazu sagen kann. Keine Ahnung, ich bin doch nur ein kleiner Kiffer. Aber es war mega spannend. Auch einmal zu sehen, es bewirkt etwas. [A-397]

> Es ist wirklich cool, dass solche Leute auf das Ganze Einfluss nehmen konnten. Dass nicht nur Leute gefragt wurden, die keinen Plan haben. Sondern Leute, die effektiv Kiffen, gefragt wurden. Das sind diejenigen, die eine Ahnung haben. Und das finde ich cool. [A-411]

Weiter gaben die befragten Personen (5) an, für sie sei es schön gewesen andere Kiffer aus unterschiedlichen Umfeldern kennenzulernen. Vier Personen äusserten, sie hätten das Gefühl, sie würden mit ihrer Mitarbeit in der Begleitgruppe (Cannabis-) Geschichte schreiben. Zwei der anwesenden Personen gaben an, bereits seit acht Jahren auf diesen Moment gewartet zu haben. Sie hätten sich bereits bei der ersten Ausschreibung von 2016 angemeldet und wären auf der Liste geblieben. Ebenfalls zwei Personen meinten, Kiffen verbinde, das sei in der Begleitgruppenarbeit spürbar gewesen. Eine Person äusserte, die Mitarbeit in dieser Gruppe habe ihr die Möglichkeit gegeben, eine neue Perspektive von Studien kennenzulernen, nämlich als Teilnehmer*in. Weitere Einzelnennungen waren: die Anzahl und Abstände der Begleitgruppensitzungen wäre gerade richtig gewesen; es sei cool gewesen, dass die Cannabisprodukte gezeigt wurden; die Diskussion sei informativ gewesen. Des Weiteren äusserte eine Person, sie fand die Erfahrung und die Unterschiede von Einzelinterview und Gruppendiskussionen spannend, wie folgendes Zitat aufzeigt:

> […] das erste Mal, da hatten wir noch ein Einzelinterview und da erzählst du nur dein Zeug. Und hier [Gruppendiskussion, Anmerkung der Autorin] ist es mega krass. Du sagst irgendetwas und zuerst etwas verhalten und plötzlich ist man da mega am Diskutieren. […] Es ist ein mega Unterschied zwischen Einzelgespräch oder ob du in einer Gruppe bist und angeregt über etwas diskutierst. Das finde ich wirklich ein krasser Unterschied und mega cool. [A-427]

Die meisten der anwesenden Personen (5) äusserten, die ***Atmosphäre*** während den Sitzungen als friedlich und respektvoll wahrgenommen zu haben. Dies machten sie daran fest, dass durch die Illegalität des Cannabiskonsums automatisch eine Verbundenheit, eine eingeschworene Gemeinschaft zwischen Gleichgesinnten entstehe. Ebenfalls eine positive Auswirkung auf die Atmosphäre hätten die neutrale Haltung, die Offenheit und der Respekt der beteiligten Nicht-Kiffer (Moderatorin, Forschungsgruppe) gehabt, betonten drei Personen.

Als ***ungeschriebene Regeln*** innerhalb der Begleitgruppen nannten die befragten Personen, Anstand und gegenseitiger Respekt (5), der sich im gegenseitigen Ausreden lassen (3) und auch darin, andere Meinungen gelten zu lassen (3) zeigte. Eine Stimme fasste die so zusammen:

> Einander ausreden lassen. Nicht jemand von der eigenen Meinung überzeugen wollen. Sondern jeder darf seine Meinung haben und diese ist auch gerechtfertigt. [A-445]

Die Begleitgruppenmitglieder wurden gefragt, wie sie ***aussenstehenden Personen erklären,*** was sie in dieser Begleitgruppe machen. Fünf Personen gaben an, zu sagen, dass sie an Gruppendiskussionen teilnehmen würden, in denen sie ihre Erfahrung als Cannabiskonsument*innen einbrächten und auf diese Weise die Forschungsgruppe in Bezug auf das Studiendesign beratend unterstützen würden. Zwei Personen gaben an, es gehe um die Legalisierung von Cannabisprodukten und eine Person ergänzte, es gehe um Gesundheitsbeobachtungen und um was Cannabis mit Konsument*innen mache.

Ebenfalls wurden die anwesenden Personen gefragt, ob sich ihre ***Einstellung zu Cannabis*** und Cannabiskonsum während der Arbeit in der Begleitgruppe verändert habe. Alle Personen gaben an, ihre grundlegende Einstellung gegenüber Cannabis und Cannabiskonsum habe sich während der Zeit in der Begleitgruppe nicht verändert. Doch äusserte eine Person, ihr Konsum habe sich in dieser Zeit verändert, da in ihrem Umfeld weniger Cannabis konsumiert werde. Eine Person gab an, sie dampfe vermehrt, da dies besser für die Gesundheit sei. Eine andere Person merkte an, dass die Diskussion über die alternativen Konsumformen sie anrege, einen Vaporizer auszuprobieren und eine weitere Person äusserte, sie sei interessiert an den E-Joints, wie nachfolgendes Zitat belegt:

> Ich habe mich schon vorher mit der Gesundheit auseinandergesetzt und vorher mit dem Vapen begonnen. Ich habe das schon gekannt. Es hat mich ein bisschen gluschtig gemacht auf diese E-Flüssigkeiten. Also auf den E-Joint. [A-218]

Eine weitere Person äusserte, es cool zu finden, dass der Fokus der Studie auf der ***Gesundheitsförderung*** (gesündere Konsumform) liege statt wie bisher auf einer Abstinenz.

Eine Person merkte folgendes an:

> Wir sind mega gut ausgeglichen. Männer, Frauen, Alter, Konsumtechnisch. Viel Konsum, weniger Konsum, starker Konsum, schwacher Konsum. Ich glaube diese Mischung hat es ausgemacht, dass wir alle hier (Produkteangebot) etwas finden, das uns passt. [A-417]

3.6 Diverses

Eine Person äusserte sich zu den erhaltenen Unterlagen als Vorbereitung auf die Erstvisite. Darin stände, dass der Studiencannabis lediglich die Person konsumieren dürfe, die in der Studie eingeschrieben sei. Die Person merkte an, dies sei realitätsfremd, da kiffen ein sozialer Akt sei und die wenigsten Personen allein kiffen würden. Sie werde ihre Konsumgewohnheit auch mit dem Studiencannabis beibehalten und mit anderen Leuten kiffen.

Eine andere Person merkte an, sie hätte folgende Idee für die Gesellschaft:

> Jetzt schlagen sie sich alle so die Köpfe ein wegen dieser AHV-Geschichte. Legalisiert Cannabis, besteuert das anständig und wir haben die AHV gerettet. [A-419]

Zwei Fragen blieben für die Begleitgruppenmitglieder offen. Die eine war, in wie vielen Apotheken sie Cannabis beziehen dürften. Und die zweite Frage: Haben die Apotheken die Geräte, die auf der SCRIPT-Intranetseite aufgeführt sind, jederzeit im Angebot?

Auf die Frage, was sie sich in der Begleitgruppenarbeit ***anders gewünscht*** hätten, meinte eine Person, mehr Personen in der Begleitgruppe wäre interessant gewesen. Der Umstand, dass sich keine weiteren Personen finden liessen, verblüffte einen Teil der Gruppe (3). Den anderen Teil der Gruppe (4) konnte dies nachvollziehen, da sie in ihrem Bekanntenkreis Personen hätten, die im Moment mit ihrem Cannabiskonsum anonym bleiben, da sie Nachteile (z.B. im Beruf) befürchten würden. Eine Person merkte an, dass für die Mitarbeit in der Begleitgruppe Werbung hätte gemacht werden können. Ideen zum Auflegen von Flyer waren: Hanfshop, Hausarztpraxen, Fachstellen, Teststelle für Cannabis.

4 Fazit

Die Begleitgruppenmitglieder waren einverstanden damit, dass Anna Schibli über die Begleitgruppenarbeit einen ***Artikel*** schreiben wird. Zwei Personen waren interessiert aktiv am Artikel mitzuschreiben, weitere Personen gaben an, bereit zu sein ein Feedback zum Artikel zu geben.

Die anwesenden Personen zeigten sich begeistert über die ***Cannabisprodukte*** und die Produkte-Demo-Gläser mit Lupe und Riechlasche. Die Cannabisblüten sähen edel aus. Das Haschisch sähe eher wie gepresster Blütenstaub aus und nicht wie echtes Haschisch. Die Preise der Cannabisprodukte wurde generell als hoch eingestuft. Die Preislegung wurde als widersprüchlich empfunden. Sie verleite Kund*innen dazu, die Produkte mit dem höchsten THC-Gehalt zu kaufen, weil da die THC-Einheit am günstigsten sei.

Auf die Frage, was sie denken, welche ***Erkenntnisse aus der Begleitgruppe für die Forschungsgruppe*** wichtig waren, wurden folgende fünf Erkenntnisse am meisten genannt:

- Nicht nur Cannabisblüten, auch Haschisch in verschiedenen Sorten
- Verkaufsperson soll eine kompetente Beratung anbieten (fachkundig Auskunft, Liste mit Sorten, Preise)
- Produkte sollen sichtbar und im besten Fall auch riechbar sein
- Auftreten der Fachperson (Sympathie, Ton) ist wichtig
- Alternative Verkaufsstellen abgesehen von Apotheken: Cannabis Social Club, CBD-Shop, Quartier-Bioladen, Tabakladen

Das ***SCRIPT-Intranet*** für Studienteilnehmer*innen wurde von allen anwesenden Personen als gut und übersichtlich strukturiert befunden. Ganz besonders positiv betonten die befragen Personen die ausklappbaren Empfehlungen für einen sicheren Umgang mit Cannabis. Die Leserschaft könne auf diese Weise selbst entscheiden zu welchem Thema sie mehr Informationen haben möchte. Bei den Safer Use Empfehlungen wünschten sich die Befragten vertieftere Informationen zu den einzelnen Fragen und Aspekten, denn wer auf das «plus» klicke, wolle mehr Informationen als aktuell darunter zu finden seien. Dies könne auch mit einem Link zu anerkannten Fachseiten wie eve & rave, Conact und Saferparty aufgenommen werden. Des Weiteren bemerkten die Befragten, unter dem Aspekt «Vermeiden Sie die Kombination von Cannabis mit anderen psychoaktiven Substanden und Alkohol» werde auf die psychoaktiven Substanzen gar nicht eingegangen, dies sei zu ergänzen. Ebenfalls seien die Informationen zu Mischkonsum zu knappgehalten. Des Weiteren sei dieser Seitenabschnitt mit folgenden Fragen und Antworten zu ergänzen: Was kann ich bei Überdosierung tun? Was mache ich, wenn ich Mischkonsum hatte? Ein Button «Feedback an die Studienleitung» wie auch eine Verlinkung zum Studienkontakt wären an dieser Stelle ebenso erwünscht.

Die Begleitgruppenmitglieder gaben an, die ***Arbeit in dieser Begleitgruppe*** sei für sie eine interessante Erfahrung gewesen. Insbesondere zu merken, dass sie mit ihrer Cannabiskonsum-Erfahrung auf das Studiendesign Einfluss nehmen konnten, hat sie bewegt. Die Atmosphäre innerhalb der Begleitgruppensitzungen empfanden sie als angenehm und respektvoll. Sie äusserten, sie fühlten

sich auch von Nicht-Kiffern (Moderatorin, Forschungsgruppe) respektvoll behandelt. Ihre Einstellung zu Cannabis und ihrem Cannabiskonsum habe sich durch die Arbeit in der Begleitgruppe nicht verändert. Doch gibt es Personen, die vermehrt vapen oder vapen ausprobieren wollen, da diese Konsumform für die Gesundheit besser sei.

5 Anhang 1: Frageroute Gruppendiskussion

Vorbereitung PBG-Abschlusssitzung

Mittwoch, 6. März 2024 18.:30 – 20:30

Einstieg, Überblick der Themen (5`)

- Anna Schibli Artikel zu PBG-Arbeit in SCRIPT (15`)
  - Thema: Auswirkung der Ergebnisse aus der Begleitgruppe auf das Studiendesign von SCRIPT
  - Anna Schibli freut sich, wenn 1-2 Personen am Artikel mitschreiben. Anna führt aus, was das bedeutet.
    - Wer von euch arbeitet mit?

- Cannabisprodukte und Geräte präsentieren (25`)
  - Reaktionen auf Produkte einholen
    - Was gefällt / missfällt
    - Was an den Produkten entspricht euren Erwartungen?
    - Was an den Produkten unterscheidet sich von euren Erwartungen?
  - Reaktionen auf die Geräte einholen
    - Wer von euch hat bereits Erfahrung mit einem dieser Geräte?
      - Falls ja, welche?

- Erkenntnisse aus der PBG-Arbeit (20`)
  - Ihr habt im Jahr 2021 am Einzelinterview teilgenommen und anschliessend an 4 Begleitgruppensitzungen. Was sind aus eurer Sicht die wichtigsten Erkenntnisse, die ihr eingebracht habt? → Sammeln an Flip oder auf ppt
  - Verteilen der Themenliste von Anna Schibli: Schaut euch diese Liste an. Kreuzt anschliessend, die für euch fünf wichtigsten Themen an.
  - Hat sich eure Haltung gegenüber Cannabis und Cannabiskonsum während dieser Zeit verändert? Falls ja, wie?

- Intranet SCRIPT (15`)
  - Seite vorstellen (participant, PW cannabis1000 →ohne Leerschlag)
    - Wie wirkt diese Seite auf euch?
    - Was fehlt? / Welche Ergänzung wäre für euch hilfreich?
    - Was ist aus eurer Sicht zu viel auf dieser Seite? Weshalb?

- Rückblick auf die PBG-Arbeit (30`)
    - Ihr habt, mit heute, an fünf Begleitgruppensitzungen mitgewirkt. Wie war diese Arbeit für euch?
        - Was hat eure Zusammenarbeit ausgemacht?
        - Welche ungeschriebenen Regeln habt ihr eingehalten, damit diese Zusammenarbeit funktionierte?
        - Was hätte eine Fliege an der Decke beobachten können, wenn sie uns in der Begleitgruppensitzungen beobachtet hätte?
        - Wie beschreibt ihr die Atmosphäre, die während den Begleitgruppensitzungen wahrzunehmen war?
        - Gab es etwas an den Begleitgruppensitzungen, was ihr ein nächstes Mal anders haben möchtet?

- Hinweis zu Gruppendiskussion von Marie-Noëlle McGarrity, Sucht Schweiz, 26.3.24 18:30 – 20:30
    - Ausdrucke mitbringen!

Abschluss und Verabschiedung (5`)

6 Anhang 2: Erkenntnisse aus der Begleitgruppe

Erkenntnisse aus dem Einzelinterview und den Begleitgruppensitzungen von 2021-2023

Einzelinterviews

- Nicht nur Cannabisblüten, auch Haschisch in verschiedenen Sorten.
- Outdoor- und Indoor-Produkte in guter Qualität.
- Verkaufsperson soll eine kompetente Beratung anbieten (fachkundige Auskunft, Liste mit Sorten/Preisen).
- Separater Beratungsraum für Verkauf auf Wunsch.
- Transparenz in Bezug auf Inhaltsstoffe, Sorte, Wirkung, THC-/CBD-Gehalte.
- Nicht mehr als 10.-/Gramm.

Gruppeinterview 1

- THC-Werte mit maximal 18% für die Blüten ausreichend.
- Bio-Qualität ansprechend, Preis von Blüten und Haschisch eher an der oberen Grenze.
- Etwas skeptisch gegenüber E-Liquids, die meisten würden es jedoch einmal ausprobieren.
- Produkte sollen sichtbar und im Besten Fall auch riechbar sein.
- Offen gegenüber einem Präventionsgespräch, jedoch nicht aufdringlich. Flyer und Informationsangebote niederschwellig beziehbar.
- Auftreten der Fachperson (Sympathie, Ton) wichtig.
- Wichtig, dass jede*r aus Forschungsgruppe mindestens 1x Cannabis geraucht hat.

Gruppeinterview 2

- Mundsprays und Cremes interessant. Zäpfchen nicht interessant.
- Mögliche Verkaufsoptionen abgesehen von Apotheken: Cannabis Social Clubs, CBD-Shops, Quartier-Bioladen, Tabakladen.
- Cannabisverkauf durch Contact nicht attraktiv da stigmatisiert.

Gruppeinterview 3

- Einverstanden mit der Einverständniserklärung.
- Webseite erfüllt den Zweck.
- Hausarzt als Kontaktperson wird kritisch gesehen.
- Rauchstoppberatung nur auf Wunsch, unaufdringlich.

Gruppeinterview 4

- Anpassungen auf Safer Use Flyer: Orale Wirkzeit, Position der Grafik.
- Video von Forschungsmitglied über Bioverfügbarkeit etc. zu langweilig.
- Videos von 4/20 grundsätzlich sympathisch, für eine Universität jedoch zu unprofessionell.
