# Supplementary material for: Consulting people who use cannabis to plan a regulatory trial on non-medical cannabis sales in pharmacies
Source: Res Involv Engagem. 2025 Oct 24;11:124. doi: 10.1186/s40900-025-00791-3 (PMC12553168; doi:10.1186/s40900-025-00791-3)
Supplement: Supplementary file 3 — Supplementary Material 3 [file 40900_2025_791_MOESM3_ESM.pdf]

Evaluation of the individual interviews on SCRIPT-2

The interviews were conducted with cannabis users

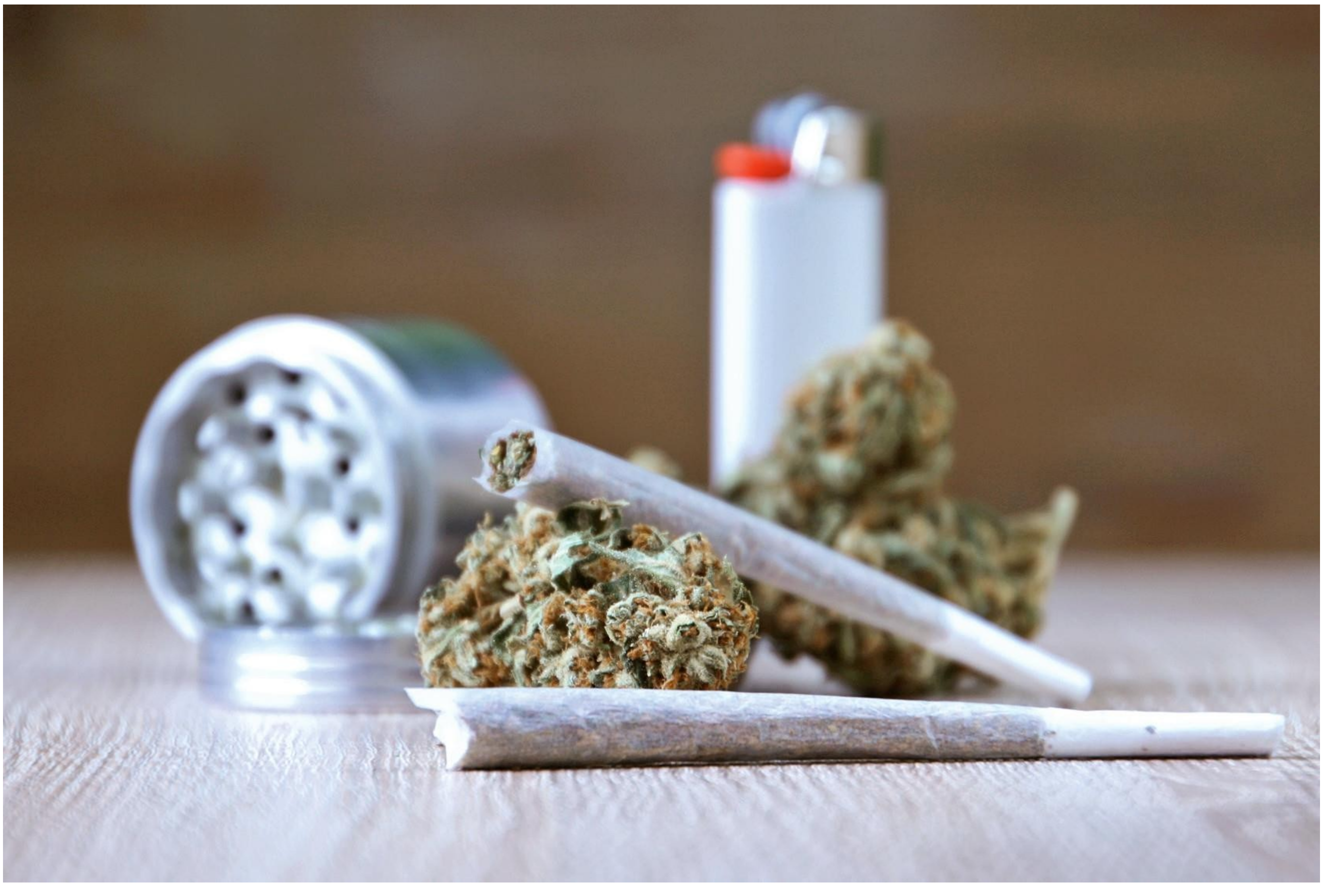

Beatrice Metry

Bern Institute of Family Medicine March

2022

Table of contents

1 Introduction ... 3
2 Methodological approach ... 4
2.1 Clarification of competence with the cantonal ethics committee ... 4
2.2 Survey and evaluation ... 4
2.3 Sample ... 4
2.4 Interview content ... 5
3 Results ... 5
3.1 Questionnaire on cannabis use ... 6
3.2 Interviews ... 6
3.2.1 What is associated with cannabis use and time of day of use ... 6
3.2.2 Current situation of cannabis acquisition (black market) ... 8
3.2.3 Initial thoughts on the regulated purchase of cannabis in pharmacies ... 9
3.2.4 Sales process in pharmacies ... 9
3.2.5 Cannabis products in pharmacies ... 10
3.2.6 Advantages and disadvantages of purchasing cannabis in pharmacies ... 12
3.2.7 Mandatory criteria ... 13
3.2.8 Differences in cannabis supply in relation to the age of users ... 14
3.2.9 The price ... 14
3.2.10 Data protection ... 15
3.2.11 A different perspective ... 16
4 Conclusion ... 17
5 Appendix 1: Questionnaire ... 19
6 Appendix 2: Informed Consent ... 23
7 Appendix 3: Glossary ... 26

1 Introduction

With the amendment to the Narcotics Act, which came into force in May 2021, it is now possible to conduct scientific studies on non-medical cannabis use.

A study on the regulated purchase and consumption of cannabis products in Bern pharmacies is planned for fall 2022 and will be conducted by the University of Bern. The overall aim of the study is to evaluate how the regulated purchase of cannabis affects the consumption behavior, health and social life of people who already use cannabis compared to people who continue to purchase their cannabis on the black market.

In order to prepare and set up sales processes and cannabis products as customer-friendly as possible, people who already regularly consume cannabis for pleasure were interviewed.

The aim of these interviews, as well as future group discussions, is to create a customer-friendly offering in pharmacies for the upcoming study. The start of the implementation study is planned for fall 2022.

This evaluation report is structured in such a way that the methodological approach is described first and then the results are presented along the question route. At the end, there is a brief conclusion and ideas as to which topics could be included in the next group discussions.

2 Methodological approach

The various parts of the procedure are described below. All interviewees signed an Informed Consent (see Appendix 2) before the interview and agreed to the interview and its evaluation by signing it. This assured them of anonymity.

2.1 Clarification of competence with the cantonal ethics committee

On May 31, 2021, the concept for the qualitative accompanying research entitled "Use of a participatory support group consisting of cannabis-using adults as a supplement during the planning phase of the SCRIPT 2 project" was submitted to the Cantonal Ethics Commission (KEK) to check its competence. The CEC found that it was *not* responsible. This means that this research does not fall under Article 2, paragraph 1 of the Human Rights Act and that individual and group interviews with the selected target group can be conducted without submitting an application. After this decision of June 20, 2021, the acquisition of participants was started.

The acquisition of cannabis-using adults who wanted to take part in individual and group interviews took some time. Initially, the focus was placed exclusively on participation in group interviews, and only one person responded. It was only when the focus shifted to individual interviews that several interested people came forward and the study could begin.

2.2 Survey and evaluation

The data was collected by means of individual interviews via Zoom along a defined question route in the period between November 1, 2022 and December 13, 2022. The interviews lasted 60 minutes on average and were digitally recorded and then transcribed.

The transcripts were analyzed using MAXQDA software. The first step involved coding the texts, followed by summarizing the individual statements and writing down the results.

2.3 Sample

Participation in the SCRTIP-2 study was advertised in various ways and cannabis users were actively sought out. Attention was drawn to this by e-mail and by means of a notice board. The advertisement only offered the prospect of group interviews or work in an active support group. Only one person responded. Only when individual interviews were conducted instead of group interviews was it possible to recruit enough people. At the end of the individual interviews, all interviewees stated that they would agree to group discussions.

A total of nine people were interviewed individually via zoom. Five of them live in the city of Bern and four in the Bern agglomeration. All interviewees stated that they were currently working, in training or studying. The sectors in which the interviewees work are diverse; they named the following: Studies (3, 2 of which were multimedia production), logistics (2), finance, graphics, photography and fitness. At the time of the survey, the interviewees stated that they lived alone (3), in a partnership (3), with their family of origin (2) and in a shared flat. All respondents stated that they were Swiss citizens. The sample is presented in table form below.

Table 1 Overview of the sample

**Year of birth:**	1957 to 2002<br>1957, 1975, 1990, 1991, 1993, 1999, 2000, 2001, 2000		
**Gender**	5 male	3 female	1 non-binary
**Occupational sector:**	Studies (3)<br>Logistics (2)<br>Finance (1)	Photography (1)<br>Graphics (1)<br>Fitness (1)	
**Living situation**	Living alone (3)<br>Living in a partnership (3)	Living in the family of origin (2)<br>Living in a shared flat (1)	
**Nationality**	Switzerland (9)		

2.4 Interview content

The interview covered five thematic blocks, which are briefly described below. As an introduction, the interviewees were asked what they associate with the consumption of cannabis and at what time of day they consume cannabis. The current situation of cannabis procurement, i.e. the advantages and disadvantages of the black market and home cultivation. Another block of topics deals with the sale of cannabis in pharmacies. The optimal sales process, the desired cannabis products, the advantages and disadvantages of purchasing cannabis in pharmacies and data protection were examined in more detail. The interviewees were then invited to take the perspective of other cannabis users they know and to contribute their opinions. Another important topic was the price per gram of cannabis on the black market and in pharmacies. Finally, questions on socio-demographic aspects were asked in order to describe the sample.

3 Results

The results are listed below along the question route. Numbers in round brackets indicate how many respondents answered in that direction. If there are no brackets after the statement, it is an individual answer. In square brackets

the number of the transcript (T) and the paragraph in the MAXQDA file (A) in which the quote can be found. The participants had already filled out a short questionnaire on their cannabis use with the informed consent. These results are presented first. This is followed by the results of the interviews.

3.1 Questionnaire on cannabis use

All nine people completed this short questionnaire on their current cannabis use.

The respondents stated that they used tetrahydrocannabinol (>1%), i.e. illegal cannabis (9). According to their statements, six people also use tetrahydrocannabinol (<1%) and two people also use cannabinol products (CBD). Most of the respondents use cannabis daily (6), weekly or weekly - monthly (2). All respondents stated that they smoked cannabis flowers. Four people stated that they also used resin. Four people stated that they vaporize cannabis flowers, one person stated that they vape e-liquid. When asked why they would use cannabis, "for pleasure, just because" was ticked the most (9), followed by "for relaxation" (7), "for nervousness" (5), "for illness/pain" (2) and "other reasons".

"other reasons".

It is possible that the information from the previous questionnaire does not match the interview results. This is due to the nature of the different types of survey and also to the fact that the interviews did not ask as explicitly about consumption habits as the questionnaire.

3.2 Interviews

The results of the interviews are presented below.

3.2.1 What is associated with cannabis use and time of day of use

When asked what cannabis users associate with the use of cannabis, they expressed different opinions. However, one thing seems to be the same for everyone, with all respondents stating that cannabis meant relaxation for them (9). The quote below illustrates this very well.

> "Generally with relaxation, winding down, also relaxation. Yes, creativity. It's something therapeutic-medical for relaxation and also loosening up. Yes, these are such good things." [T5-A3]

Furthermore, enjoyment (6), attitude to life (5), ritual (5), passion (3), serenity (2), togetherness, sensuality, intense perception (e.g. of music) and spontaneity were mentioned as associations with their own cannabis use. From this diversity of what cannabis means to the interviewees, two quotes are given below to underline these statements. The first quote refers to the ritual and the second to the passion associated with this plant.

> "I get up in the morning, have a coffee and make myself a joint and enjoy it afterwards with a coffee on the balcony. So it's actually in the morning. And when I work, it's before work, of course. I get up in the morning, smoke one afterwards and then go to work, comfortably." [T8-A9]
>
> "It is a versatile plant and I am fascinated by this plant." [T8-A13]

In addition to these enjoyment aspects, three people also mentioned medication with cannabis, mainly in connection with pain (e.g. menstrual pain, back pain) or to treat hyperactivity.

Most of the respondents use cannabis in the evening (7). The following quote is an example of this.

> "It's actually always towards the evening, about 2-3 hours before I go to sleep. This has to do with the fact that I can sleep off the after-effect and I don't really feel anything the next day." [T6-A7]

Two people stated that they also used cannabis in the morning *before*, but not *during,* work. Three people stated that they did not use cannabis before or during work, firstly because concentration was less pronounced (2) and secondly because cannabis and its use is still stigmatized by society (2), and therefore no one at work should know that cannabis is used in leisure time.

> "No, I don't use weed at work. But at work, nobody else knows that I smoke weed in my free time. That's because there's a stigma attached to it, which makes it a bit of a scapegoat, maybe if you're ill or forget something or otherwise lack concentration. Then it's very quickly said: "Aha, he's just smoking pot."" [T5-A53]

With regard to the effects of cannabis at work, one person said that their concentration or focus was better after using cannabis. One person said that they worked better after using cannabis because they were less nervous.

All respondents stated that they currently mainly smoked cannabis flowers. Two people each emphasized that they prefer indoor and outdoor cannabis. Resin was also consumed (4), sometimes mixed with flowers instead of tobacco. Three people stated that they regularly use CBD hemp (smoke (3), in beauty products (1).

3.2.2 Current situation of cannabis consumption (black market)

The main sources of supply mentioned were acquaintances (7), with the reasoning that the quality was safe. If the acquaintances had nothing more to sell, they would buy cannabis from acquaintances of acquaintances. One person remarked that this was unpleasant, that she felt like a supplicant in this situation and that it caused her stress. Several people said that when they went out and smoked with others, the source of the weed might not be known and that it was more of a spontaneous thing (3). If a cannabis source disappears, e.g. because the indoor facility has been discovered by the police or because the cannabis user has moved house, the search for a new, trustworthy dealer begins. This is exhausting and time-consuming (3). The majority of respondents expressed the conviction that the black market had no advantages (6). The following advantages were mentioned by the others: there are more exotic varieties on the market (with the price that the origin is not known); the community among cannabis users was positively emphasized; anonymity, if desired, is given; no cannabis tax is levied on the black market, as is to be expected in regulated distribution (analogous to alcohol or tobacco tax). All interviewees said that the criminalization of cannabis use was one of the most stressful aspects of the black market. This is succinctly illustrated by the following quote:

> "It's always the case that the police could catch you at any moment. And then you get a fine and the weed is gone. Yes, that's just sad. I find it sad that a plant can be illegal. It really is a plant. I think it's absolutely fine for something chemical to be illegal. No problem. But the plant itself, ...// Because they're not evil." [T8-A37]

The following further disadvantages were mentioned: there is a health risk from cannabis that has been cut (3); you have to take what is on the market (2); the quantity bought is usually less than agreed (e.g. 4.7 grams instead of 5 grams); pure cannabis is hard to get on the black market; the quality varies; hanging out with criminals and the unreliability of some dealers (as a buyer you have the short end of the stick) is stressful.

Several people stated that they grew their own cannabis (4), one of these people described having had a very rich harvest and perceived this as a disadvantage, as the harvest clearly exceeded their own needs. Home-growing is also illegal and there is always a risk of being caught (3). The advantages of home-growing are seen in the quality of the end product; home-grown weed is also not as strong as purchased weed. One person noted that growing one or two cannabis plants in the garden is a hobby and also fun.

One person mentioned the protection of minors on the black market, as only people over the age of 18 would be allowed to buy cannabis in pharmacies with regulated dispensing, while younger people would continue to buy it on the black market. Another person pointed out that regulated supply could be a thorn in the side of the black market.

3.2.3 Initial thoughts on the regulated purchase of cannabis in pharmacies

Respondents were asked to imagine themselves in the situation of regulated cannabis dispensing in pharmacies and to describe the first things that went through their minds. Several people compared the upcoming setting with cannabis outlets abroad (coffee shops in Amsterdam, social clubs in Spain) (3), with a tea and herb store (2) and also with a Netflix series about a cannabis pharmacy (Disjointed). One person first thought of a separate shelf for cannabis products containing CBD and THC in the pharmacy. Another person expressed the idea of a cannabis assortment with different varieties in safe quality. The cannabis products would be labeled and the product properties described
(2). The following quote visualizes the initial thoughts:

> "I see a counter in front of me, with someone in a lab coat standing behind it, because the whole thing has to remain quite sterile. And yes, that you have different storage jars where you can smell it. And you can touch it [addition: the cannabis] and see what it looks like. And there are these different jars at the back. That you can still buy accessories, papers, filters. That there might also be a fumoir, so that you can try it [addition: the cannabis], that wouldn't be bad either, of course. Maybe some comfortable sofas so that you can sit together and talk. Because stoners like to talk to each other." [T8-A43]

Another interviewee first thought about the advantages of cannabis being available in pharmacies. For example, they described no longer having the stress of procuring cannabis and being able to obtain cannabis in good, consistent quality and in the agreed quantity. Three people said that their first thoughts were joy about this political and social step. This is underlined by the following quote:

> "A good move by Parliament and society, I would say. I think that's great. I think it also helps a lot of people. And when I think about it later, when I go into the store, I would certainly be interested to see how it is presented in the store. What it might even smell like. How the staff respond to it. Whether they are helpful, whether they can perhaps give advice. I would be very interested - if there was a stall - to go past the cannabis stall and have a look. And maybe read through a brochure. I would be very interested in that. [T3-A39]

One person said that this thought felt special. She thought of people who pick up their methadone to get rid of their addiction.

3.2.4 Sales process in pharmacies

Most of the interviewees said that they could imagine the sales talk in the public sales area of the pharmacy (7) and that they would openly express their product wishes. Two people expressed certain reservations about buying cannabis products in the public area of a pharmacy.

One person said that, especially at the beginning of regulated cannabis use, it would be helpful to have an adjoining room to conduct a cannabis sales talk until cannabis is seen as "normal" in society. And the second person said that it should always be possible to hold a sales talk on personal topics in the adjoining room. Two other people pointed out that selling cannabis in the public area of the pharmacy would contribute to social acceptance and that side events should therefore be avoided.

In the interviews conducted, it became clear that cannabis users wanted competent advice in pharmacies (6). Pharmacy staff should also be able to advise customers on cannabis according to their needs, as they do with medicines (6). They should be able to provide expert information (effects, properties, ...) on various cannabis varieties and products (4). Furthermore, an overview of the product range would be desirable, either verbally (3) or in writing in the form of a product list similar to the map of the coffee shops in Amsterdam (2), as the following quote makes clear:

> "Maybe there would also be a list. [...] There are these varieties, the prices. Some kind of map, that would be good. You can quickly read through it, it has this, it has that. Or that the salesperson could ask: "What do you need it for? Is it more that you want to be high at the weekend, or more for...//" Yes, just really competent advice like I would get for any other medicine or stimulant." [T4-A37]

Furthermore, one voice emphasized that they would like to be informed about cannabis news.

In the salesroom, it is important that the products are on display (2), but only accessible to pharmacy employees. This could take the form of a display case or a shelf. One respondent was convinced that the visibility of cannabis products would also contribute to the social recognition of cannabis and to normality.

3.2.5 Cannabis products in pharmacies

The respondents clearly stated that they expect to find flowers (9) and hashish (8) in pharmacies. Flowers are seen as a standard product (9) and hashish as something special (5).

The respondents stated that they expected a range of flowers from outdoor and indoor production, some said that outdoor in organic quality (3) was very important, others indoor quality (3) and still others found both variants fine (3). Furthermore, the selection of different cannabis varieties (sativa, indica, ruderalis) (4) as well as different CBD and THC contents
(4) was expressed as an expectation.

Diversity was also desired with regard to hashish, with names such as: Charas, black Afghan, red Lebanon and green Maroc as well as a mixed product (Moonrock) were mentioned.

> "Absolutely resin. So the solid ones. Afghan, Lebanon, Maroc, all these fine things. Because you can't make them yourself. So I can only buy them." [T9-A49]

Other cannabis products that would be welcome in a pharmacy in the future: Gummy bears (5), creams (lip, body creams) (4), oil (for rubbing in and for ingestion) (3), syrup (2), cookies (2), tea, cough syrup, throat lozenges, chewing gum and liquids for vaporizers. The respondents rejected tablets because they lacked the pleasure (taste, smell) and the ritual (building, rolling). One person said that cannabis gummies reminded them of Alkopop and that it was unfair to young people to bring them onto the market. Two people thought that edibles should be clearly labeled, especially sweets such as gummy bears, so that it is clear and visible to everyone that cannabis is present and has a delayed effect.

Products related to cannabis use, such as filters, papers, lighters, vaporizers, pens and much more, are not necessarily expected in a pharmacy (3), but there are grow stores and Fourtwenty stores (2).

> "You know, there is this Fourtwenty store. There are a few grow stores or something like that. You can get all these stoner accessories there. I don't necessarily think you need to have all that in the pharmacy. But really the breadth of cannabis products, the substance, so to speak. I would like to see a variety of everything there is." [T1-A47]

One person emphasized that filters should be offered in pharmacies that sell cannabis, and even included in the sales pitch. This is because many cannabis users believe that filters minimize the THC and thus the experience of getting high. The filters should also be sold as prevention and education (health, effects).

> "What I always preach to everyone and what I think the pharmacist should also be able to sell. That would be filters. That she tells young people in particular: you know that THC that you want to get high with will pass through any filter. Only that which is not good for the lungs remains in the filter. Can I give you another packet of filters? I think that's extremely important. I keep seeing guys offering their girls something and they're all coughing like mad. And when you know that it's still *fashionable* to just make a paper filter and not use a real filter. And when I tell the boys that, they say: Yes, but it's so expensive. They simply don't have enough of this cannabis and think that THC remains in the filter and that's not true. That's education, and I think that's important information. The intoxication is exactly the same, even with a good filter. And when you see what's in the filter, then it's clear where else it is when you smoke without a filter. So, this is really very important information that you have to tell all young people. [T9-A47]

Respondents attach particular importance to transparency, be it in terms of ingredients, variety, effect and THC and CBD content (6) as well as the type of growing and fertilizing conditions (e.g. indoor, outdoor) (6). This information should be clearly visible on the packaging. Furthermore, value is placed on the appearance of the flowers and on the smell, which means that the flowers should be clearly visible (5) and smellable (4) (e.g. in the form of a tester). This aspect is underlined by the following quote:

> "I would still find this positive. As with make-up, where there is always a tester, there would also be a tester so that you wouldn't have to buy a pig in a poke. There are people who are much more interested in figures, data and facts and who simply want to have all the details exactly. And there are people who pay more attention to everything else. In other words, they pay more attention to the senses. And I think it should appeal to both." [T1-A74]

Some of the interviewees (4) attach importance to a consultation or sales talk in order to be able to buy a product that meets their expectations as closely as possible. Two respondents said that a variety of products and types of hemp was important to them. Two people said that they would only buy outdoor hemp and two others would only buy indoor hemp. One person said that they did not care whether it was outdoor or indoor hemp, as long as the THC content was not too high. In contrast, two people said that a high THC content was very important to them. Aspects such as regionality and value for money were also mentioned as important.

The interviewees commented on which of the expected products mentioned above they would buy themselves. Eight people mentioned flowers, with one person specifying that they would only buy indoor cannabis. Another person stated that they would not buy any flowers at all, as they would continue to grow their own. However, she would be interested in hashish, as would other respondents (6). Four people said that they would try out the range. Individual mentions were: CBD or THC oil (also for ingestion), cookies and throat lozenges.

When asked what they would think of three products with different CBD and THC contents (CBD high/ THC low/ CBD and THC medium/ CBD low, THC high), five people said that this would be a good start. One of these people went on to say that she was not interested in CBD at all, as it made her nauseous, and would then probably opt for the third option (CBD low, THC high). Another person added that the cannabis plant has much more to offer than these three variants.

The packaging of cannabis products was addressed by some respondents. A few stated that the packaging was not important at all (3). Others, however, thought that the packaging should be neutral, simple and yet elegant (2), for example in a cardboard box with a window (2), in a jar (2) or otherwise in a small box.

3.2.6 Advantages and disadvantages of purchasing cannabis in pharmacies

Many ***advantages*** are seen in the regulated purchase of cannabis in pharmacies. For example, the safe quality (9), the ease of purchase for everyday use (5), the selection of cannabis products (4) and the competent advice (3) were positively emphasized. The transparency of the ingredients (THC, CBD content) (2) and personal safety during the purchasing process (pharmacy versus underpass) were also mentioned. The following quote from a respondent shows how buying cannabis could become part of everyday errands:

> "That it can become part of everyday errands. I walk up Münstergasse through the market, buy potatoes, vegetables and milk. On the way back - I always have the

> I always have the feeling it should be at my local pharmacy...// Yes, just something normal. The way I pick up my packet of Panadol or something...// I don't see anything at all that's offensive about it being in the pharmacy. I think that's where it belongs." [T4-A27]

On a social level, the interviewees expressed the following advantages: Cannabis would be recognized in society (5); illegal structures would be broken up, thus weakening the black market (5); regional farmers would be taken into account and this would generate jobs and free up police resources. Two people stated that they only saw advantages in selling cannabis in pharmacies.

> "I personally don't see any disadvantages. No, I just have the feeling that it's more available, it's closer. You don't have to prepare a week in advance if you want to buy something quickly. I actually only see advantages." [T5-A79]

Disadvantages are seen in the presumed higher prices (5). One of these people stated that it would be particularly difficult for young people or people with a limited budget to pay a higher price in the pharmacy and that these people would be forced back into the black market. These concerns are illustrated by the following quote:

> "A young person struggling with his apprentice's wages, for him two francs more, is just two francs more." [T9-A94]

Another voice assumed that the prices had to be higher than on the black market because the sales staff, quality control and any tax would be passed on to the products. As pharmacies are regulated dispensaries, there is a certain degree of control (of cannabis consumption) and this could also be disruptive (3). Another person said that cannabis use could become mainstream because cannabis is accessible to everyone in this way.

3.2.7 Must criteria

When asked which must-have criteria would have to be met for respondents to buy their cannabis from a pharmacy in future, the following criteria were mentioned: impeccable product quality (8); the price must be right (5); a selection of cannabis products (this makes the difference to the black market) (4); competent advice (3) and specialist knowledge regarding the optimal storage of cannabis. This was followed by individual responses. One voice made it clear that they expected organic quality. Another person demanded transparency with regard to ingredients and fertilization. And a third person said that cannabis should (also) be treated like an everyday product in pharmacies (no stigmatization). Another person said that she would like to be able to see and smell the product before she buys it.

3.2.8 Differences in the supply of cannabis in relation to the age of users

During the interview, respondents were asked what the difference was between younger and older cannabis users, which revealed heterogeneous views. For example, two people said that older people would prefer to consume cannabis with a lower THC content, while younger people tended to prefer a high THC content. One person said that older people tended to prefer hashish, while younger people preferred flower. Older people would prefer quality over quantity and discretion. One person stated that age does not matter, smoking weed brings people together, as the following quote emphasizes:

> "I think that's the feeling of community again. Because it doesn't really matter whether you have an 18-year-old in front of you or an 80-year-old. If you find out who is smoking weed, then you already have a connection with that person." [T1-A128]

3.2.9 Price

Black market

The units in which purchases are made on the black market were specified differently. Some people buy their cannabis in liter containers, others in jam jars, others in francs or grams. The most common sales unit seems to be the franc. Some of the interviewees (5) stated that they bought packets of 20, 50 or 100, with the numbers - 20, 50, 100 - referring to the amount to be paid. Most people stated that they spend 50 francs per purchase (4). Three people said that they liked to buy larger quantities at once so that they would last for a while and the price would be slightly lower (bulk discount). Two of the interviewees said that they grow their own weed and only buy hashish on the black market or sometimes buy weed on the black market when they go out. The prices on the black market for cannabis flowers were stated differently, but are in a range between five and ten francs per gram. The following statement by one interviewee is an example:

> "Well, as far as I know, my friend pays six francs a gram. But that's extremely cheap, it's a friend's price. I think you normally pay between eight and ten francs per gram. But then again, ten francs is really expensive." [T7-A138]

Two people made price differences between indoor and outdoor flowers. They would pay between five and seven francs per gram for outdoor flowers and between eight and twelve francs per gram for indoor flowers, because indoor flowers cost more to grow (room rent, electricity, fertilizer, higher risk of being caught). Hashish is slightly more expensive on the black market than cannabis flowers. The interviewees stated that they would pay between ten and twenty francs for a gram of hashish on the black market, depending on the variety and the exclusivity of the product.

Pharmacy

There were very different answers as to how much cannabis flowers should cost in pharmacies. Some said that cannabis flowers should cost the same or only slightly more in pharmacies than on the black market (3), while one respondent specified that the price in pharmacies should be a maximum of five percent higher. The reason given was that there are also people among cannabis users for whom even a marginal increase in price is not affordable and these people would therefore continue to buy on the black market. Three people also thought that ten francs per gram of cannabis flowers would be a fair price. Some people differentiated the prices. For outdoor cannabis flowers, these voices would find a price of seven to a maximum of eight francs per gram reasonable (3). They would pay up to twelve francs for indoor cannabis flowers, and even up to 15 or 20 francs for very special cannabis varieties. However, the quality would have to be in the exclusive range (2). One person stated that they would pay up to twenty francs per gram for cannabis flowers in pharmacies, on the grounds that pharmacies have a selection, the quality is right and the production, storage and labor costs must also be taken into account. The same person specified that high-priced cannabis must also be exclusive in terms of quality, origin and growth and must therefore clearly stand out from standard products such as those presented in the interview. Two people pointed out that cannabis in pharmacies at a price of ten or twelve francs per gram would act as a deterrent.

Hashish is likely to cost between ten and twenty francs per gram in pharmacies, depending on the variety, origin and quality. Black Afghan, red Lebanon and green Maroc, among others, were raved about. One voice compares the situation to wine, as the following quote shows:

> "If I could get the best quality black Afghan now and I can't get it anywhere else. If I know now that I can get it regularly from the pharmacy, then I would look at the price. But if I can get it once and I have to pay 200 for these 10 grams, then I would do that too. If it's the best ever, then you pay 50 or even 100 francs. If it's the best ever, then you pay 100 for a bottle (of wine) or something. Although, of course, that's an insane amount of money for a bottle of wine, but if you know you can make your loved ones really happy and it's a super, super wine, then yes, you can." [T9- A170]

Online trading was mentioned by one person. She stated that she sometimes bought Californian cannabis flowers and paid a much higher price (~14 francs per gram), but also received exclusive quality.

3.2.10 Data protection

With regard to data protection, the interviewees agreed that the data must not be passed on to third parties (police, road traffic authorities, employers, etc.) and that this must be ensured (9). One person mischievously commented that for anyone who carries a smartphone

on their person, data protection is not guaranteed anyway. Several people said that a separate room should be available for advice and sales (4), so that cannabis users who wish to do so can protect themselves and do not have to give their personal details (name, address) in the sales room. Another protective measure mentioned by one voice was the plain packaging of cannabis products.
Respondents would like to identify themselves using a credit card-sized card (4) or an app (3) with a chip on their key ring. Only one person said that they would prefer to use a corresponding app, as they always have their smartphone with them anyway. Several people stated that they did not have a smartphone (3), which is why only a card would be an option. One person noted that the card should be designed as neutrally as possible. And another person pointed out that an ID check could deter young people from buying their cannabis at the pharmacy. Furthermore, one person suggested that the study card should be checked against an official ID (e.g. identity card). Another person said that study registration should not be done via an online platform, but instead on paper, which they would find more secure.
In connection with data protection, one person also raised the issue of police checks. She suggested that a unique ID number should be noted on the student or cannabis ID card, which the police could use to check the authenticity of the student.

3.2.11 A different perspective

Respondents were asked to take the perspective of someone they know who uses cannabis and to express what might be particularly important to this person in relation to the purchase of cannabis in pharmacies. Data protection and discretion (4) as well as the opportunity to make the purchase in a separate room (3) were mentioned. These statements are underpinned by the following quotes:

> "The first thing I think about is my partner. She also uses cannabis. [...] Well, she grew up in a religious home. And cannabis is not something that people like to talk about in religion itself. It's forbidden in that sense. And, maybe in the store itself she wouldn't have a problem buying it (cannabis), but if the family found out, then I can imagine that the family wouldn't..." [T6-A157]
>
> "And if that came out. I can imagine that wouldn't come out so well. [T6-A159]

Furthermore, the respondents mentioned the price (2), easy and regular accessibility (pharmacies have store opening hours) (2), advice and information from specialists, a regular, differentiated range and the transition to an everyday product as important aspects from the point of view of their acquaintances. Another respondent also said that it was important to offer hashish products as well as cannabis flowers.

4 Conclusion

The people surveyed welcome the regulated sale of cannabis in pharmacies. They expect to be able to buy cannabis flowers as well as hashish and, if necessary, edibles. They attach particular importance to the quality and purity of the cannabis products, with some raving about organic quality products. They consider a planned selection in terms of THC and CBD content (THC high - CBD low/ THC and CBD medium/ THC low and CBD high) to be a good start in the right direction. Safe cannabis quality and the recognition of cannabis in society are seen as major advantages. A possible disadvantage is thought to be the higher prices. The current price on the black market was stated to be in the range of five to ten francs per gram of cannabis flowers. In pharmacies, most respondents do not want to pay more than ten francs per gram for cannabis flowers. If taxes are incurred, these should be invested in addiction prevention. In addition to competent advice, the optimal sales process also includes the possibility of smelling the products or being able to inspect the product; the idea of working with test vials came up. It is important to the interviewees that pharmacies offer a selection of cannabis products (cannabis flowers, hashish, etc.), as this would clearly distinguish pharmacies from the black market. All respondents stated that they themselves buy directly in the sales room of the pharmacy, but that they would like a separate room for the consultation and sale of cannabis, so that those who do not want to "show themselves" can also purchase cannabis in the pharmacy, protected from prying eyes. This separate room was also included in connection with the topic of data protection. With regard to data protection, the interviewees requested that their personal data not be passed on to third parties (police, road traffic office, etc.).

For the group discussion with the same people as interviewed here, the following topics are conceivable for in-depth discussion:

- The discrepancy between the desire for acceptance of cannabis in society and the simultaneous desire for a separate room in pharmacies.
- The optimal sales process with a view to the approach by pharmacy staff. If there is a need for (data) protection, it is important that customers do not talk about their cannabis purchase in front of other customers. How could this be elegantly resolved?
- Address indoor and outdoor to see whether cannabis users can be reached with the outdoor products offered in the study. Or what the outdoor products would have to offer so that indoor cannabis users would also come to the pharmacy.

- How important hashish is as a cannabis product for the target group. If hashish was not offered, how this group of users would still purchase cannabis in the pharmacy.
- Several people stated that they did not consume tobacco, not even to roll joints. It would be interesting to find out what they mix cannabis with, especially hashish, in order to be able to offer these additives (e.g. cannabis leaves) in pharmacies.

5 Appendix 1: Questionnaire

Introduction

Let me introduce myself: Research associate at the BIHM, responsible for the interviews and their evaluation during the Cannabis Trail study

The research project SCRIPT 2 (The Safer Cannabis - Research In Pharmacies randomized controlled Trial 2) deals with the regulated sale of cannabis products in pharmacies in the city of Bern. The aim is to gain insights into the effects on the consumption and purchasing behavior and the well-being of the study participants. The sale of cannabis products in pharmacies is planned for fall 2022.

It is important to us to incorporate the experiences, ideas and views of people who regularly consume cannabis into the planning of the cannabis range and the sales processes in pharmacies as early as possible, which is why we are contacting you.

The topics of the interview are sales processes, cannabis products and data protection

The interview lasts around 60-90 minutes.

The interview is recorded digitally. This enables us to transcribe what is said as accurately as possible into writing so that we can then carry out a computer-aided evaluation. After the evaluation, the recording is deleted so that what you have said is anonymous and cannot be linked to you personally. Your statements will be summarized with the statements of the other interviewees in the final report. Your name will not appear in the report.

You have already signed and submitted the declaration of consent. Thank you very much.

Do you have any questions before we start the interview? Do you agree with the procedure described? (→ Start recording)

Thank you very much for taking the time for the interview. Then we will start the interview.

Getting started

- **What do you associate with the use of cannabis?** *Relaxation, company, music, after work, ...*

- **At what times of day do you use cannabis?**

Current situation, black market

We start with a look at the current situation. At the moment, it is only possible to buy cannabis containing THC on the black market.

- **How do you currently obtain cannabis?** *(from friends, acquaintances, always from the same dealer, on the street, from strangers, sometimes like this, sometimes like that, ...)*
- **Are there any aspects of the current acquisition of cannabis** *(on the black market)* **that you find stressful?**
    - If so, what are these aspects?
    - And what would you like to see instead?
- **Are there aspects of the current situation of cannabis use that you see as an advantage?**
    - Aspects that you would not want to miss in the future?

Pharmacy

Now let's take a mental leap into the future.

- **When you think of buying cannabis in a pharmacy, what is the first thing that comes to mind?**
    - What does it look like?
    - What other details come to mind?

Sales process

Now we come to the actual sales process in the pharmacy.

- **Imagine you walk into the pharmacy. You are greeted, and afterwards, what happens next?**
    - Please describe your idea in as much detail as possible.
    - How do you envision the sales process?

Cannabis products

Now we come to the cannabis products to be offered in the pharmacy.

- **What kind of cannabis products would you expect to find in a pharmacy?** *(e.g. flowers, resin, tincture, liquid for vaporizing, tablets, ...)*
- **Which products do you think you would buy yourself?**
- **Which products would you like to try?**
- **What do you attach particular importance to when choosing a product?** (e.g. *THC concentration, packaging, dosage form, degree of purity, ...)*

Advantages and disadvantages of selling cannabis in pharmacies

- **Do you see advantages in being able to buy cannabis products in pharmacies?**
  - If so, what are they?
  - What are the advantages of buying cannabis in pharmacies?
- **Do you see any disadvantages to buying cannabis in pharmacies?**
  - If so, what are they?
  - What could prevent you from buying cannabis in pharmacies?

Data protection

As the trade in cannabis and the consumption and possession of large quantities of cannabis is still illegal, I would like to address the issue of data protection.

- **Is data protection an issue in relation to the purchase of cannabis in pharmacies?**
  - If so, which aspects are important to you?
  - What other aspects come to mind?
  - What precautions would you welcome?

Must criteria

We have now looked at the acquisition of cannabis in pharmacies from different angles and talked about various things.

- **When you think about acquiring cannabis in pharmacies, what must be taken into account so that you can acquire cannabis in pharmacies in the future?**
  - Possibly take up the previously mentioned advantages of the black market and ask:

Obtain other perspectives

In a further step, we would like to get your opinion on how other cannabis users might view the purchase of cannabis in pharmacies.

- **When you think about cannabis users you know, what do you think is particularly important to them when it comes to purchasing cannabis from dispensaries?**
- **Do you think that the age of cannabis users makes a difference when it comes to purchasing cannabis in pharmacies?**
  - If so, what do you think the differences are? Please provide more details.
  - If not, what do you think are the similarities between cannabis users?

Cost / Price

We have now talked about cannabis products, the sales process in pharmacies and also about the black market. We are also interested in prices.

- **What kind of cannabis do you currently buy on the black market?**
  - In what quantity per purchase?
  - How much do you pay for it? *(In CHF)*
  - *If possible, tell me the price per gram?*
- ***What would you be willing to pay in a pharmacy for a comparable amount of cannabis?***
  - *If possible, tell me the price per gram?*

Socio-demographic data

So that we can describe the sample as well as possible, I will ask you a few more questions about yourself.

- **What is your current occupation?**
  - To what extent?
- **How would you describe your current living situation?**

alone / with my family of origin / in a shared flat / in a partnership

Thanks and conclusion

We have almost reached the end of the interview.

- **Is there anything else we haven't talked about that you think is important in connection with this topic?**
  - Or is there anything else you would like to tell us?

Thank you very much for the interview and your valuable answers, as well as for the time you have taken. I would be happy to contact you again for further interviews.

6 Appendix 2: Informed Consent

Your experiences with cannabis are in demand!

Dear interested parties

With the amendment to the Narcotics Act, which came into force in May 2021, it is now possible to conduct scientific studies on non-medical cannabis use.

A study on the regulated purchase and consumption of cannabis products in Bernese pharmacies is planned for fall 2022 and will be conducted by the University of Bern. The overall aim of the study is to evaluate how the regulated purchase of cannabis affects the consumption behavior, health and social life of people who already use cannabis compared to people who continue to purchase their cannabis on the black market.

In order to prepare and set up sales processes and cannabis products as customer-oriented as possible, we conduct individual interviews and, if necessary, set up a so-called participatory support group. For the individual interviews and the participatory support group, we are looking for people who already regularly consume cannabis for pleasure.

The aim of the work is to create a customer-oriented offer in pharmacies for the upcoming study with people who already regularly consume cannabis for pleasure.

Procedure
Information can be collected in two ways. On the one hand with individual interviews, on the other hand in a so-called participatory support group, where topics are discussed in a group discussion. At present, it is still unclear whether both data collection methods will be used. We will start with the individual interviews and may invite you to a group discussion at a later date.

Basic information on the individual interviews
- Individual interviews last around 60-90 minutes and are conducted digitally (e.g. via Zoom).
- The interviews are digitally recorded (audio), then transcribed and analyzed and anonymized.
- The interviews are remunerated at CHF 30 per hour.

Work in the participatory support group
- A meeting of the support group lasts around 2-3 hours and takes place on the premises of the University of Bern
- Eight to ten participants
- The group meeting(s) will be digitally recorded (audio), then written down and analyzed and evaluated anonymously.
- The confidentiality of all personal information relating to the participants, the content developed and discussed, as well as the different opinions within the group is also binding beyond the joint work and applies to both participants and researchers.
- The sessions are remunerated at CHF 30 per hour.

What you need to bring:

- Interest in actively engaging with the topic of cannabis acquisition in pharmacies
- Willingness to engage in discussion, with the ability to accept other opinions
- Regular cannabis use (at least monthly, with THC content >1% for enjoyment)
- Minimum age of 18 years

Exclusion criteria
Association activity or a profession associated with cannabis or its use. Persons who use cannabis exclusively for medical reasons.
Age, gender and consumption behavior are taken into account when selecting participants.

Do you have any questions? Then please contact either the person who asked you or Beatrice Metry atbeatrice.metry@biham.unibe.ch or Anna Schöni at anna.schoeni@biham.unibe.ch

We look forward to hearing from you! Best regards

Head of the individual interviews and the participatory support group

Beatrice Metry

From the research group

Anna Schöni

Declaration of consent

I have read the above information for participants in the individual interviews and the participatory support group on the subject of *cannabis use* and understand what the upcoming discussions will be about. I accept the information as well as the responsibilities and form of cooperation described therein. If I have any further questions, I will contact Anna Schöni from the research group or the head of the individual interviews and the participatory support group Beatrice Metry directly.

I know that I can withdraw my participation in the interviews or the participatory support group at any time without reason and without negative consequences. I know that the data collected will be treated with absolute confidentiality and that it cannot be linked to me personally.

I confirm with my signature that I have understood the explanations and that I agree with the procedure described.

Surname and first name: ____

Address ____

Telephone number: ____

Place, date: ____

Signature: ____

7 Appendix 3: Glossary

Term	Explanation
Blue cookie	Blue Cookies is an indica-dominant hybrid cross of two heavyweight cannabis strains, Girl Scout Cookies and Blueberry. Blue Cookies puts users in a state of euphoria. The effect begins in the head, then spreads throughout the body and leads to relaxation. Sweet berry flavors blend with earthy cherry notes. Beginners should approach Blue Cookies with caution. For those accustomed to cannabis, the potency of this strain is perfect.<br>You may also come across another variation of Blue Cookies, a selected phenotype of Girl Scout Cookies that lacks Blueberry genetics.
BHO	Butane hash oil Read more under ***Wax***
Cannabidiol (CBD)	Cannabidiol is a cannabinoid from female hemp. It has been described as having anticonvulsant, anti-inflammatory, anti-anxiety and anti-nausea effects. CBD has no psychotropic effect and therefore does not fall under the Narcotics Act.
Cannabinoids	Cannabinoids are natural substances obtained from Indian hemp (Cannabis sativa), as well as synthetic analogs (artificially produced, similar substances). The most effective substance is tetrahydrocannabinol (THC).
Cannabis edibles	Edibles that are mixed with cannabis. E.g. cookies, cakes, fruit gums, brownies, honey, drinks, ...<br>Due to their psychotropic effect, edibles fall under the Narcotics Act and not under the Foodstuffs Act (Erl. BetmVD, p. 9) and require child-resistant packaging (ibid., Art. 11, p. 10)
Cannabis Social Club	is a model project proposed in 2005 by the pan-European organization ENCOD to enable the legal cultivation and distribution of cannabis as an intoxicant to persons of legal age. A Cannabis Social Club is a non-commercial association that organizes the professional, collective cultivation of a limited amount of cannabis to meet the personal needs of adult club members. Cannabis Social Clubs exist so far in Spain, Belgium, the Netherlands, Austria, France, Germany, Italy, Slovenia.<br>https://de.wikipedia.org/wiki/Cannabis_Social_Club
Cannabis-ruderalis	Autoflowering cannabis plants
CannaTrade	International Cannabis Expo in Bern<br>Over 250 exhibitors from all over the world present proven products, novelties and innovations.<br>https://www.cannatrade.ch/de/

Charas	a handmade, typical type of hashish that comes from India. It is not obtained by sieving dry flower material as in the Arab world, but from the fresh plant. Hemp flowers are rubbed between the hands in a laborious and time-consuming process until the cannabis resin sticks to the palm of the hand and is then rubbed off the hands to form tubers. The hemp flowers are used 2-3 weeks before they are ready for harvest and the THC content in the plant is at its highest, in order to harvest the sacred resin.<br>harvest.<br>https://www.bushplanet.com/Was_ist_Charas
Dabs (dab)	Cannabis extracts with a high THC content. The reason for this name is that the consumption of one dab is sufficient. Dabs come in different forms.<br>***Shatter*** tends to have a high THC content. It takes the form of a hard, glassy substance that often resembles amber. Most shatter varieties have a THC content of between 70 and 90 %. ***Wax*** has a much softer texture, similar to plasticine. The THC content of wax averages between 50 and 80 %.<br>***Sugar wax*** has a harder consistency than pure wax. It breaks in two when touched and has a certain resemblance to sugar. The taste and terpene profile of sugar wax is usually better than wax, but it still has a high THC content of 60 to 90 % on average.<br>***Crumble*** is exactly what it sounds like. It is a wax that breaks down into small crumbs when touched, sometimes even into a powder-like substance. Crumble is made by exposing it to various amounts of vacuum and temperatures during the extraction process. Crumble also has an average THC content of 60 to 80 %.<br>***Budder*** has a sticky consistency similar to grocery store butter. Its rich aroma and terpene profile combined with THC levels between 50 and 70 % make Budder a mild alternative to Shatter.<br>***Live Resin*** is made by extracting fresh cannabis flowers instead of dried ones. Live Resin is a concentrate that tastes incredibly 'terpy' (full of terpene flavor) and usually has a high THC content of between 80 and 90% or even higher.<br>***CO2 Sapp*** has a rich terpene profile. CO2 Sapp is mostly produced like Live Resin.<br>Its THC content is on average around 50 to 60 %, making it a great alternative for anyone looking for an intense high.

	The Pure is one of the strongest forms of cannabis concentrates available on the market. The Pure is a clear liquid that is often enriched with other ingredients such as apple, grape or lemon terpenes to give it flavor. The THC content averages 99%.<br>***Rosin*** is a type of cannabis extract that is made by heating cured or dried cannabis flowers. As soon as the flowers are compressed under the effect of heat, resin is secreted. This resin is then collected and consumed. On average, rosin contains a THC content of 60 % or more.
Dabbing	The inhalation of vaporized cannabis concentrate through a glass apparatus known as a dab rig.
(Delta-9-)Tetrahydrocannabinol (THC)	is a psychoactive substance that belongs to the cannabinoids. The substance is found in plants of the hemp genus and is said to be the main cause of the intoxicating effect attributed to it
dark	The cannabis strain Dunk-a-Roos is a sativa-dominant hybrid
Edibles	***Edibles*** is the term for foods to which cannabis extracts have been added. They are often found in baked goods such as cookies and brownies, but also in drinks, breads and sweets. When consuming edibles, it can be assumed that the active components from the extracts take longer to take effect, as they have to be absorbed by the digestive system.<br>must be absorbed by the digestive system.
El Kif	This is the name of cannabis in Morocco, where cannabis is mainly grown in the Rif Mountains.
EMCDDA	European Monitoring Center for Drugs and Drug Addiction<br>European Monitoring Center for Drugs and Drug Addiction (EMCDDA). This is an agency of the European Union based in the Portuguese capital Lisbon. It publishes an annual report<br>on the state of the drug problem in Europe.
Entourage effect	The ***entourage effect*** is the assumption that all of the plant's compounds work together and achieve a better effect than they would on their own. Hemp plants contain more than 120 different phytocannabinoids. These act on your body's endocannabinoid system (ECS), which regulates and balances a number of functions and processes. Cannabidiol (CBD) and tetrahydrocannabinol (THC) are two of the best researched phytocannabinoids.<br>The term ***entourage effect*** was first coined by Dr. Ben-Shabat in 1998. The concept was explained and expanded upon primarily by Dr. Etahan Russo.

	Research suggests that taking them together - along with other natural compounds in the cannabis plant known as terpenes or terpenoids - is more effective than taking CBD alone.
Fufi	5g cannabis= 50 francs
Gorilla Blue	Gorilla Blue is a cannabis plant with medicinal and healing properties, as it is an ally against pain and has a great<br>has a calming effect on the body.
Growshop	Offers everything for growing cannabis at home Tents, irrigation systems, seeds, lighting, ventilation, substrates, pesticides,<br>packaging material, ...
Hashish / Hash / Shit (resin)	Hashish is the collected and usually pressed "resin" of the hemp plant. It can be obtained not only from the flowers, but also from leaves covered in resin. Depending on the<br>quality and production method, its color varies from light grey-brown to matt black.
Haze	Haze strains have a very high THC content, which results in a distinctly strong effect when smoked. However, Haze owes its great fame and high demand to its very special effect when consumed. Due to the high sativa content, the high is often even psychedelic and hits the head.
Headshop	Head shops are usually small stores that sell accessories for the consumption of cannabis. They sell water pipes, bongs,<br>vaporizers or long cigarette papers for rolling joints, but also normal tobacco pipes.
Hybrid hemp	Many of the cannabis varieties on the market are so-called hybrids, which means that they are the product of a cross between several cannabis species.<br>Although the three species ***Sativa, Indica*** and ***Ruderalis*** belong to different cannabis families, they can all be crossed with each other.<br>***The aim is to*** use new, highly potent varieties with special genetics and the best possible<br>characteristics of several varieties in order to combine them in one.
Indoor hemp (Indica)	Indoor hemp grows indoors, usually under artificial light and irrigation with the addition of substrates. It is more popular because of its more intense flavor and higher THC content
Jack Herer	Jack Herer is one of the most iconic cannabis and hemp activists. He is best known for his 1985 book 'The Rediscovery of Hemp'.<br>Few names in the cannabis industry evoke emotion like Jack Herer. Jack is arguably one of<br>most iconic cannabis/hemp activist in history and his work has left a significant mark on the cannabis

	cannabis industry and the legalization movement. However, despite his characteristically radical and passionate views on cannabis, as well as the prohibition and potential of the hemp plant, Jack was not always the weed enthusiast we know today. In fact, he first tried cannabis when he was well into his 30s. In 2010, he sadly passed away due to complications following a heart attack
Jack Herer weed	The strain can be found worldwide from California to Amsterdam and beyond.<br>Originally bred in Holland in the 1990s, the aim was to develop a plant that would deliver an uplifting effect commonly associated with<br>Haze strains, but with a reduced flowering time.<br>The exact genetics of Jack Herer are a closely guarded secret and something of a mystery, but it is widely believed that Jack is a 50% Haze cross with some Northern Lights and Skunk. All of these strains are legendary and potent in their own right.
California hemp	Available from online retailers, nicely packaged, zip-tied
Kief / Keef /Skuff	powdery substance made from the plant hairs of the hemp plant
Knaster	Old name for industrial hemp. The flowers were smoked in the past. Today, knaster is also used as a tobacco substitute. Today, Knaster often contains other herbal ingredients such as red clover, elderberry leaves, dandelion, skullcap and other fermented components. fermented ingredients.
Kush	Kush is an old cannabis strain that belongs to the Cannabis indica species. The cannabis strain originally comes from Afghanistan, Pakistan, Iran and northern India.
Lappe	10g cannabis= 100 francs
Marijuana / Weed (flowers, grass)	Marijuana or weed refers to the dried flowers of the female hemp plant. The "resin" of the plant, with its high concentrations of THC, CBD and other cannabinoids, is found on glandular hairs on these flowers. Depending on the quality, origin, cultivation method and degree of degree of drying, marijuana is usually green to brownish, sometimes also white or slightly purple.
Moonrock	Hemp flower soaked with hemp oil and dusted with hemp pollen<br>The THC content is sky-high. A Moonrock is in fact a combination of cannabis tips,<br>hash oil and kief.
NPS	New psychoactive substances (synthetic cannabinoids)
Outdoor hemp (sativa)	Grows outdoors, in the field or in pots. Mostly natural hemp. Has many small leaves on the flowers

	leaves on the flowers, which must be plucked away before consumption.
PIED	performance and image enhancing drugs Traded online like NPS
"Praliné"	Joint with hemp flowers and resin
Red Lebanese	Is hashish. The plants from which "Red Lebanese" is obtained are left in the field until the resin glands are fully mature and have taken on a golden red color. The whole plants are then cut off above the ground and tapped into boxes. The mature resin glands break off very easily, fall to the ground and are then pressed into so-called "pucks".<br>Red Lebanese is the name for a particular type of hashish from Lebanon. It is not a variety designation in the strict sense: many relevant publications assume that the term "Red Lebanese" - regardless of the production method or processing - merely refers to the country of origin, Lebanon. Other interpretations make the red earth in the Lebanese Bekaa Valley responsible for the name "Red Lebanese". Red Lebanese is more specifically described as hashish from the Lebanese mountains, which is usually packaged in flat-pressed linen bags and is characterized by a high resin content. high resin content.
Black Afghan	Is hashish. "Black Afghan" is obtained by rubbing the resin off the growing plant. After rubbing, its color is still green and only turns black through prolonged kneading. Black Afghan originates from Afghanistan and is black on the outside and brownish on the inside. It has an intense odor, a high THC content and is usually softened with sheep or goat fat. Afghani burns very slowly and heavily and makes you tired quickly. The same applies to Black Pakistani, although it has a harder consistency. consistency.
Space Cookie	Cookie that is infused with cannabis, e.g. with cannabis butter or cannabis chocolate
Spice	Synthetic cannabinoids that mimic the effects of THC
Extender of marijuana	Brix<br>Sand, quartz sand<br>Soapstone Sugar<br>Hairspray<br>Haze spray (room spray, fragrance<br>spray) Glass<br>Vegetable extenders, flour, spices (rare) Lead<br>(most toxic extender)<br>Phosphorus / potassium fertilizer
Tobacco substitute	Herbal cigarette without nicotine

Terpenes	Terpenes are found in all plants, and terpene concentrations are often highest in flowers. The purpose is quite simple: terpenes attract insects for pollination through their aroma.<br>At the same time, terpenes also protect against pests, acting as a natural insecticide, so to speak. Terpenes are a highly heterogeneous and very large group of chemical compounds that occur naturally in organisms as secondary constituents.<br>Terpenes= essential oils (approx. 120 different ones)
Tweni	2g cannabis= 20 francs
Mixed cannabis products	Cannabis products in which narcotics of the cannabis effect type are mixed with additives by the manufacturer, for example to modify their absorption, effect, taste or appearance.<br>appearance.
Cannabis ***wax*** / crumbles / honeycumb / budder	Cannabis wax is a form of BHO (butane hash oil). It is produced using butane (a liquefied gas) as a solvent for the extraction of the main cannabinoids and terpenes from the cannabis flowers and trim.<br><br>The resulting mixture is then processed into a concentrate that looks a bit like beeswax. The cannabis wax manufacturing process varies and can result in a variety of different products with unique textures and colors.<br>textures and colors.
Wietpas	"Weed ID card"<br>On May 1, 2012, three southern provinces (Limburg, Noord-Brabant and Zeeland) introduced the "Wietpas" (weed card) as a pilot project with the aim of introducing it throughout the Netherlands at the end of the year. This failed due to protests and was soon restricted to these three provinces again, with each city being allowed to decide independently against the Wietpas. The Wietpas ensured that there was no drug tourism.<br>Only those who have a Wietpas can buy in coffee shops.
Bag of 50	"50ig" refers to the price, not the quantity of the contents/product
420 / 4:20/ 4/20	Fourtwenty is a common code word in the USA for regular cannabis consumption<br>Find out more at<br>https://de.wikipedia.org/wiki/420_(cannabis culture)

Evaluation of the group discussion on SCRIPT

The group discussion was conducted with cannabis users

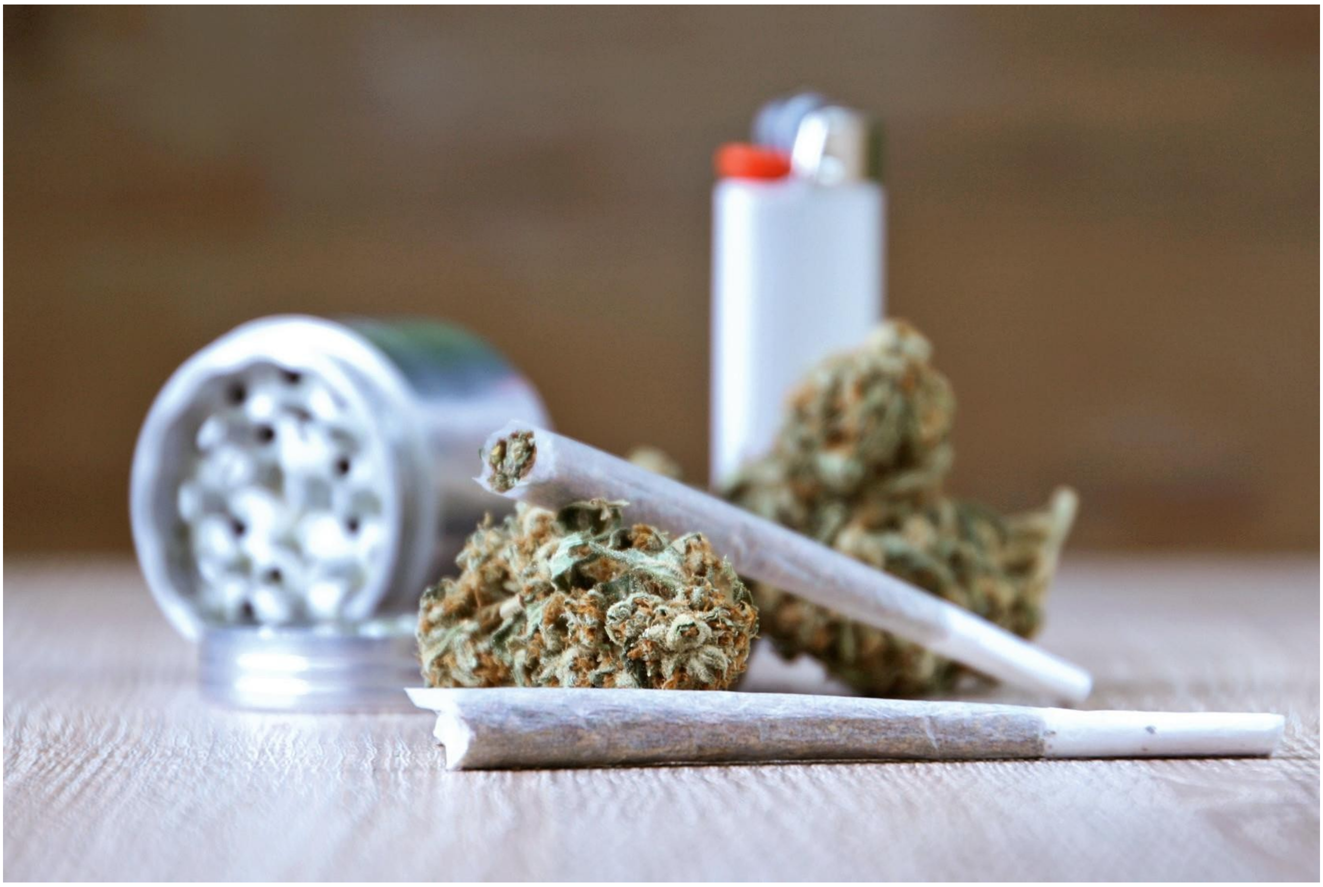

Beatrice Metry

Bern Institute of Family Medicine July

2022

Table of contents

1 Introduction 3
2 Sample 3
3 Results from the group discussion 4
3.1 Binding to SCRIPT 4
3.2 Use of vaporizers and e-joints 4
3.3 Cannabis products 5
3.3.1 Flowers 5
3.3.2 Resins 7
3.3.3 E-liquids 7
3.3.4 Oil 8
3.4 Presentation of the products 8
3.5 Prevention in the pharmacy 10
3.6 Possible points of sale 11
3.7 Challenges of the research group 12
3.8 Miscellaneous 13
4 Conclusion 13
5 Appendix 1: Group discussion questionnaire 15

1 Introduction

At the end of 2021 and beginning of 2022, individual interviews were conducted with cannabis users and initial findings were obtained for the implementation of SCRIPT. Since then, the research group has carried out further preparatory work for the implementation of the study. Current topics on which the perspectives of the end customers were to be obtained in a group discussion were

- Provide information on the current status of the study, in particular present the study design
- Vaporizers, e-joints
- Presentation of the available cannabis products
- Prevention in the pharmacy
- Possible points of sale, except pharmacies
- Challenges for the researchers

The issue of alternative points of sale became very topical following the parliamentary motion "No cannabis pilot trials in Bernese pharmacies" (RRB no. 674/2022), submitted by the SVP and EDU). The motion insists that no stimulants be sold in Bernese pharmacies. This contradicts SCRIPT, as the study is aimed at recreational cannabis users and has so far targeted pharmacies as sales outlets and involved them in the study accordingly. On June 22, 2022, the Government Council forwarded the postulate to the Grand Council of the Canton of Bern with the recommendation to accept the postulate.

The evaluation report outlines the sample and is then presented to the subjects along the question route, which can be found in the appendix.

2 Sample

All nine people who had already taken part in the individual interviews and signed the corresponding informed consent were requested for the group discussion. The appointment was arranged via doodle.
Six cannabis users, two people from the research group as listeners and the discussion group leader were present.

**Year of birth:**	1957 to 2002<br>1957, 1975, 1991, 1993, 2001, 2002		
**Gender: male**	3 male	2 female	1 non-binary

3 Results from the group discussion

The results are listed below along the question route. Numbers in round brackets indicate how many respondents answered in this direction. If there are no brackets after the statement, it is an individual answer. The paragraph in the MAXQDA file (A) in which the citation can be found is listed in square brackets.

3.1 Binding to SCRIPT

The study design was presented and the randomized distribution into start and control group was pointed out. The control group can start using cannabis 6 months later. When asked whether the participants would wait if they joined the control group and were asked to participate in another study in which they could obtain cannabis directly, the answer was as follows: One person said that she would participate in both studies instead of waiting. However, she considered it very unlikely that she would be recruited. The others said that they would wait for the sake of the study (5), as their consumption was still guaranteed. Other reasons for waiting were a personal contribution on the road to legalization or making history on the road to decriminalization and legalization of cannabis in Switzerland.

> I also think that smoking weed for science is like writing history at this stage. There is a wave of decriminalization and legalization going on globally. [A:89]

Another voice focused on product quality, as the following quote underlines: "And I think that's one of my main motivations, just to get better quality, or controlled, good quality. [A:90]

One critical comment concerned the price when cannabis is sold in pharmacies. The idea was that cannabis would be more expensive than on the black market. Another person pointed out that it could also be the other way around, that the black market would become more expensive because cannabis could now also be purchased in pharmacies.

3.2 Use of vaporizers and e-joints

When asked who of those present already had experience with a vaporizer or an e-joint, all persons (6) stated that they had used cannabis with a vaporizer to varying degrees or that they regularly used it in this way. One person stated that they vaporized exclusively during the week and only smoked rolled joints at the weekend. Only one person declared experience with e-joints. The respondents expressed various critical aspects of vaporizers and e-joints. It lacks the sensual, the pleasurable, the shared (3), it does not have the same effect and it requires electricity, as the following quote underlines:

> Yes, that stops me too. Something I have to batch. You can say we're doing something for our health, but we're destroying the environment. [A:128]

Another voice said that she hears more people coughing who consume cannabis with vaporizers and e-joints. Advantages were seen in the fact that it is much healthier to vape (3), the taste is better, more intense (2), the environment smells less strongly of cannabis after vaping (2), it is quicker than rolling and smoking a joint and the leftovers after vaping can still be used for cooking and baking, as the following quote underlines:

> 'The positive thing about that is you can recycle the weed you've smoked. It's vaporized and then you have this brown stuff in it and you can put it in a cake and it'll blow your mind. [A:130]

One voice said she would only consider e-joints and vaporizers as a supplement to rolling joints. She had also never heard of a cannabis user who had switched completely to vaporizers.

When asked under what circumstances the attendees would vape an e-joint or vaporize cannabis, they said they would try e-joints for the sake of the study (2) or take them on a long flight. Three people said they would not even start using e-joints. All participants stated that they had already had experience with vaping, but not all of them had their own vaporizer and were not (yet) prepared to buy their own device. Two people said that they would switch to a vaporizer completely and would also buy one if it became necessary for health reasons. The following quote illustrates this very well:

> Exactly, if I had a lung problem. Yes, from a health point of view. If a doctor suggested that I should really stop smoking now.
> Then I would perhaps take a little, discreet drag on a vaporizer. [A:399]

3.3 Cannabis products

The participants were presented with the various products (flowers, resins, e-liquid, oil) and asked whether they liked them and whether they would buy them.

3.3.1 Flowers

All participants would buy the four cannabis flower varieties presented, at least to try them out. Most of the respondents found the THC levels to be absolutely sufficient at a maximum of 18% (5), one person said that they were used to flowers with higher THC levels and were therefore somewhat disillusioned. It is also this person who prefers to consume indoor flowers, all

others found outdoor flowers appealing. The Swiss organic quality (3) and the appealing names of the various flowers (2) were also positively noted. The price was considered clearly too expensive by three people, one of whom would like to see a volume discount introduced. And another of these three people thought that these prices were at Amsterdam level. As the following quotes prove:

> So one day I will definitely try it out. Or something, but otherwise I see it more like this at the moment, if the black market isn't there. No matter how harsh that sounds now. But if I have to pay 15 francs more for weed that's no more than as good as the weed I already have and I've already had it tested anyway, and it's not stretched, then I don't see it that way. Maybe just once, because I feel like it. [A:171]

> It's relatively expensive in terms of price, I would say. 13 francs, that's already Amsterdam level. Outdoor, you can get that for five francs and then the question of efficiency and effectiveness really arises, who pays almost double that? I have the feeling that you only pick up the biologists who want to do something good and don't want to support the black market. [A:157]

Another person found that she would like to compare the products from the black market and the pharmacy. The shows the following statement:

> I would buy it too. I would perhaps also compare it directly with the same variety from the black market. Because that would also interest me. [A:235]

Another person thought the price was okay, she currently pays CHF 50 for 5 grams, she wouldn't afford the most expensive one every time, as the following quote underlines:

> So I don't know now if I would always go for that 65 CHF, I rather think not because it would be too tight on the pocket. But I'm already paying CHF 50 and CHF 45 is less anyway. So, if I could "feel" (try) it, of course, and it would be nice, then yes. [A:167]

Two other people said that they paid more attention to quality than price.

The discussion turned to the ratio of THC to CBD and what effect this has. One of the participants clarified this and the high THC levels on the black market as follows:

> 'I just know that CBD has a THC-inhibiting effect. Most other cannabinoids attenuate THC. The purer the weed is, i.e. the more THC it contains, the stronger its effect. [A:214]

> I have the feeling that it certainly has something to do with the strains. And yes, I think it's more by range. In medicine, they often work with high CBD levels, I've heard.

> meant. And I believe it has developed in this direction (high THC values) on the black market. So from a profit point of view. The more it "chleps" the better you can sell it, the more expensive you can sell it. That's how I would explain it as a derivation. [A:216]

3.3.2 Resins

Everyone in the discussion group said they would try the two types of resin, although critical aspects were also mentioned. For example, the price was rated as expensive (4), one person thought that these prices were ok, because hash goes further than weed and in Amsterdam or Barcelona you would also pay between 20 and 30 euros for 1 gram of hash. Another person criticized the THC - CBD ratio and thought that the THC content would be significantly higher on the black market. Another person said that she would definitely seek advice, as these names meant nothing to her. As with the flowers, the names of the resins were positive and appealing (2). The choice of two different resins was ok, said two voices, because on the black market there is usually only one variety. One person suggested indicating the degree of filtering of the resin.

3.3.3 E-liquids

None of those present had any experience with e-liquids. Four people said they would try e-liquids at least for the sake of the study. Two people found e-liquids unappealing. Even the name "e-liquid" was off-putting. And the "artificial" ingredients were totally off-putting, as it was also unclear what effect they would have on health. Two people found the purchase of an additional device disproportionate and one of these people would welcome being lent a device to use during the study. Another person stated that she deliberately did not smoke e-cigarettes because she had observed among her friends that more was consumed with e-cigarettes. Normal and additional e-cigarettes are consumed, and the latter are better and available everywhere. For this reason, she would only want to try out e-cigarettes once so as not to build up a habit. Another person replied that it was precisely this availability that fascinated her and that she could therefore imagine using e-cigarettes. The following quote illustrates this ambivalence:

> That doesn't sound very appealing. I still find the available thing interesting, because there are situations where I would like to have a quick puff, but it's not appropriate for the situation. And so that could be interesting. [A:347]

3.3.4 Oil

The oil offered in the study is food-based and intended for consumption. Three of the participants were familiar with this type of oil. One person used it for everything, i.e. for smoking, in salad dressing, in tea, in yoghurt and on other occasions. Another person stated that they use it for rubbing against pain and a third person for smoking or enriching joints. This person pointed out that the "usual" THC oil (60-70%) was very dark and sticky and therefore also unwieldy and unpleasant to reseal. All those present stated that they would buy the THC-CBD oil presented at least to try it out.

It remained unclear until the end whether the attendees realized the difference between the sticky THC oil and the oil offered here for consumption.

3.4 Presentation of the products

A key issue was the transparency of the products. A comparison was made with fresh food counters (2) and a jewelry display case. In both variants, customers would be served personally and a conversation between pharmacist and customer would arise naturally, also with regard to (prevention) advice (2). Another comparison was made with decorative cosmetics. Test jars that can be opened to smell and look at the product are conceivable (3). All those present were positive about the option of presenting cannabis in "jam jars" (6). But *can smelling* also be a purchase criterion? All those present agreed on this too, albeit for different reasons. No, it is not a purchase killer if the product cannot be smelled (6). This was explained as follows:

This is ok during the study, but if cannabis becomes accessible to everyone later, this point should be included (4). This is shown in the following quote:

> That is then perhaps only the next level. Or, if we establish it here now. Aha, you can buy it at public points of sale. And then I think it won't last long and then it will be like in Amsterdam, where you have a menu at the counter. But you can't always smell it there either. [A:499]

Two other voices said that cannabis often doesn't smell like it tastes.

> And there's also the fact that weed often doesn't taste the way it smells from the nose. It can often smell mega-awesome and be mega-shitty to smoke. [A:496]

One person said that they were used to not having a choice, in the sense of "there is what there is". Another person expressed a similar view. She said that it depended on the

She said that whether *being able to smell* was important depended on the size of the selection and that it was not absolutely necessary given the current limited choice. *Being able to smell* was mentioned towards the end of the discussion as a plus point in the sales process and should be possible on request.

Being able to see seems to be more important. Several people (4) said that they would like to see the product before they buy it. It was described as a kind of quality control. It is important to see what the flower looks like, whether it has leaves and seeds inside, as the quote below shows:

> You see, if it's got a hell of a lot of leaves on it, it's got seeds in it. And if it has beautiful flowers. [A: 472]

The participants (4) spoke of transparent packaging that makes it possible to see the product. One person said that she would like to see the product being weighed and packaged in the pharmacy, as this would give her peace of mind and would also be justified for the price. Another person replied that she had full confidence in pharmacies, and that it was more important to her that the product was vacuum-packed and resealable. This person did not think that a transparent bag was essential, but it was prettier. With regard to the visibility of the products, the topic of where cannabis products should be presented in the pharmacy came up. This is because cannabis will only be available for purchase by study participants during the study. If the products are made visible, they will also be visible to people interested in cannabis who are not taking part in the study (2).

One person raised the issue of Boveda. Boveda regulates the moisture of the product in the jar and supports the maturing process. Another person replied that this only works if the jar is opened regularly - once a day. Boveda would also have an effect on the fragrance. If Boveda is used, the flower must be broken in order to release the fragrance, as the following quote underlines:

> So with the Boveda, you don't smell it like that afterwards. You always have to break it open so that you can smell it. [A: 441]

Another point relating to transparency concerned the effect of the product. Information from people who have consumed the product themselves would be desirable. As the following quote shows:

> 'There could be testers who make videos where they test it. That would be a 3-minute video. They say it's this and that. You see them smoking a little bit. They felt so and so, so and so is the button. You could do that in conjunction with a scan. So on the table a poster "Scan me". And then you can scan this and then two or three people come and test it and say things about it. [...] But otherwise

> I would also wonder what it's really like to smoke, what does it smell like, what effect does it have and how does it make a person feel? [A: 557]

In response, two voices said that the participants in the current support group would be ideally suited to making videos about cannabis products.

3.5 Prevention in the pharmacy

Pharmacies offer a wide range of services, including advice on medicines, their effects, side effects and addressing (addictive) behavior. Those present said that they would be open to a (prevention) discussion when making their first purchase (5), especially for specialist information on cannabis products (4). According to those present (4), a repeated prevention discussion would tend to be annoying and demotivate people to buy cannabis products in pharmacies. Two voices would welcome it if flyers on vaporizers, oral cannabis use and smoking cessation were available as low-threshold offers. Another person mentioned the variant with information videos of users that can be accessed using a QR code (see section 3.4). One person referred to the risk scale shown earlier in the discussion in connection with the prevention talk in pharmacies. And said the following:

> 'I was quite surprised by that red-green table at the beginning. How blatant it is. And we all didn't react well to this technical stuff before. I could now imagine a sympathetic pharmacist saying to me: "If they sometimes feel their lungs a bit and they feel ...// Then it would be like this device, it's a technical device but it's rad, the difference for your lungs. I would recommend it to you." [...] ...the difference was still quite stark. And we've all seen it now anyway, we haven't found it, but we must have tried it. But if they sold it to me well, then maybe I would still be (available). Because then I think: "Ok, but sometimes I can feel it too." Then maybe I'd still give this thing a try, even if I don't like it that much. But health isn't completely insignificant to me anyway. [A: 548]

This voice went on to say that it was the tone and the way in which the specialist spoke to her and conducted the conversation that made the difference, which met with approval in the group (4).

3.6 Possible points of sale

As already made clear in the introduction, the aim is to collect and examine ideas for possible points of sale outside pharmacies. The participants were invited to share their ideas. Which they actively did.

Coffeeshops like in the Netherlands or cannabis social clubs like in Spain or even cannabars were mentioned by all those present as a suitable option (6), as the following statements show:

> [...] but for example there is the Kon-Tiki coffeeshop. You can shop downstairs and smoke upstairs. They sell CBD, but nobody smokes CBD there. That it could be sold in coffee shops like that, where you can smoke anyway. [A:598]

> It's similar to the social clubs in Spain, you just have to be a member. As soon as you have this membership card, you have closed rooms afterwards, they sell cannabis at the bar, you can buy drinks, there are ping-pong tables. Yes, it's really like a coffee shop. [A:608]

> I really like going to Amsterdam in between. And I always think it's so sophisticated [...] Yes, exactly, there's something so classy about it. I sit in the coffee, I have a fine coffee and afterwards I have the (cannabis) menu. Well, we only have four on our menu, but that doesn't matter. [A:601]

Followed by CBD stores (5), neighborhood organic store (4), tobacco store (4), four-twenty/grow/head stores (2), directly from the farm (2), newly established foundation for cannabis distribution (2) or a wine shop. The idea of buying cannabis online met with moderate interest, at least during the study. Only one person stated that they already order their cannabis products online. The others stated that they found it impersonal, unappealing and also dangerous. One person said that ordering online and collecting locally would be a feasible option for them, which would also ensure a certain degree of social control. Another person argued that the online option would eliminate the need for a prevention meeting. And yet another voice thought that the pharmacy would be the right place to sell cannabis simply because of the ongoing legalization process.

A methadone and heroin dispensary was clearly rejected by all participants. Here are some voices that underline this:

> That gives a stigmatization and an equality with heavy addicts. And I have the feeling that this is the paradox. Hemp is a natural herb, ...// Of course you can become addicted, but we're not talking about a physical addiction. [...] But the fact that you have stoners and all the other users in between - ecstasy, LSD, cocaine and all these things - then weed is automatically put on the highest level, with heroin. [...] [A: 652]

> That would be the wrong place, you can't do that. Veto! [A: 655] We're
>
> already paying more. Why would I want that? [A: 656]

3.7 Challenges of the research group

Challenges for the research group were viewed very heterogeneously by the group. Only this input, that all researchers of this SCRIPT study should have had at least one experience with cannabis so that they know what they are talking about or to expand the research approach, was approved by several people (3). This is evidenced by the following quote:

> I think I would think it's important that everybody who is working scientifically on this study actually puff one. Just so you know - I'm totally serious and not stupid. And in the sense of Albert Hoffmann. Why was Albert Hoffmann able to scientifically prove psilocybin, for example? The discoverer of LSD, why? Because everyone else was looking in the wrong direction. That's very interesting. You have to read about it in detail, scientifically, why Albert Hofmann was able to detect not only LSD but also psilocybin in mushrooms. There is a point in science where you can no longer use the old, scientific way of thinking, but you have to know what you are talking about. [A: 672]

Two people saw the placement of the cannabis products as a challenge. The products are only available for purchase by participants in the study, how are other people interested in cannabis dealt with, what are they told in pharmacies? Two other voices said that the outdated image of cannabis users (hippies) and of the cannabis plant itself (beginner's drug) in the population was a challenge and that the study should change this. One person said that prevention "through the back door" might not go down well with buyers. It would be important not to appear patronizing or instructive. Here is the following quote:

> Are all the people who smoke weed really open to this prevention thing? On the one hand, you want to make it accessible [...] And yet, a bit of prevention work through the back door or something. So that you might feel a bit patronized, experienced weed smokers. [A: 664]

Another person noted that with these prices, a discount system for "heavy users" could promote acceptance. And a final voice said that they were simply happy that the time had come. The only risk was that the study could suddenly be overtaken by the changing legal situation (decriminalization before the end of the study).

3.8 Miscellaneous

The use of cannabis with a bong was discussed and the procedure briefly described. The idea was raised that this type of consumption could be healthy. However, this was vehemently denied by one person who has some bong experience.

One person expressed an interest in tobacco substitutes, i.e. being able to buy them in pharmacies. Other participants who do not use tobacco use male cannabis plants or female cannabis leaves. Mint was mentioned as a tobacco substitute, as was damiana herb, which is offered on the market as a tobacco substitute.

The wish was also expressed to receive a tasting pack of the various products as study participants in the support group.

4 Conclusion

Overall, the selection of cannabis products presented was rated positively by the participants. With the exception of the e-liquids, all participants stated that they would at least like to try the products. The initial, absolute aversion to the e-liquids eased somewhat during the discussion and four people said that they would like to try this type of cannabis consumption (see Chapters 3.2 and 3.3.3).

After a lengthy discussion, the majority of the group felt that *being able to smell* the cannabis product before buying it was not so central to the current selection and could be included in the next step at the points of sale. On the other hand, *being able to see* the products was considered important, also because this would show whether they contained leaves or seeds, which would say something about the product quality. As not all people who work in pharmacies smoke weed, short videos on the individual products would be welcomed (e.g. accessible via QR code). In these videos, users should say something about the taste and effect. These statements are more credible than from a person who does not consume cannabis themselves.

The participants were cautious with regard to the prevention talks in pharmacies. For example, it was said that cannabis products are made available to everyone in good quality on the one hand and then prevention sneaks in through the back door. The situation is completely different when it comes to advice, where users want a knowledgeable person in the sales talk.

If pharmacies should not be allowed to sell cannabis products that are consumed for pleasure, the attendees mentioned various other points of sale. First and foremost coffee shops/cannabars and CBD stores. Online sales met with resistance. This is impersonal, unappealing and also dangerous. It was argued that there was a lack of social control

and no prevention talks could be held. However, this would also be a sore point for the other outlets mentioned here.

5 Appendix 1: Questionnaire for group discussion

Preparation of support group for cannabis users SCRIPT

July 4, 2022 / 18:30 - 20:30

Min	Topic
15`	***Introduction, round of introductions (short, possibly only first name, place of residence)***<br>Welcome, clarify framework<br>Introduce agenda (ppp)<br>Invite to a short round of "Who is who" (name, place of residence, interest in the study)
15`	***Current status of SCRIPT (ppp slides 3-7)***<br>Background: Why is the regulated acquisition of cannabis important?<br>Course of the study and current status<br>Focus and research question of the study: Explain "harm minimization", show risk scale<br><br>***pppSlide 5***<br>→Would you also take part if you were in the control group and had to wait 6 months before buying cannabis in pharmacies?<br>• ***If yes***, let's assume there was a second study in Bern. You could buy cannabis there immediately. Would you then leave "our" trial and join the others or would you stay and wait 6 months?<br>• What are the reasons for waiting or switching?

<table>
<tr><td>20`</td><td>

Offer cannabis varieties, devices (ppp slides 8-13)

Present strains and equipment and ask per slide (flower, resin, oil):

- How does it affect you?
- What do you notice?
- What do you think of the price/product ratio?
- Would you buy these products?
- Why is xy important?

E-liquids (slide 14)

Can you imagine vaping cannabis (e-liquids, e-joints) or vaporizing it (flowers, resin)?

  - What speaks for it? What's against it?
  - If you don't already do it: Under what circumstances would you vape or consume e-liquids?
  - What could be a reason for you to vape cannabis?

- Do these devices appeal to you visually?
- Which one would you choose?

What would you look for in your selection process?

</td></tr>
<tr><td>20`</td><td>

Presentation of cannabis products in pharmacies (slides 16-17)

- How important is it to you
  - To be able to look at cannabis?
  - To be able to smell it?
- Why is that so important to you? What exactly do you look for?
- When do you refrain from buying cannabis?
  - Characteristics
  - When you can't smell it / can't look at it

</td></tr>
<tr><td></td><td>

Description of cannabis products (slides 21-26)

- What do you expect from a product description?
- How does this description affect you?
- What stands out?
- What do you particularly like?
- What is missing?
- How should the taste be described?

</td></tr>
</table>

<table>
<tr><td></td><td>
o How do you talk about the taste? What words do you use or are common among cannabis users? What do you know?
o How are you used to it?
</td></tr>
<tr><td>20`</td><td>
Prevention in pharmacies (ppp slides 27-28)
In the SCRIPT study, it is planned that pharmacists will also be active in prevention. This means that they will advise their cannabis customers (e.g. on cannabis concentration) or hold discussions if they notice conspicuous cannabis or alcohol use.

What do you think of the fact that prevention is offered or actively addressed?
How far should the specialist go with their advice/approach to you?

o What kind of advice/conversation would you appreciate?
o Where would you draw the line? (in the sense of too close, none of his/her business, ...)
o What consequences would you draw?

Approach, advice/conversation not desired and continue to buy cannabis in this pharmacy
No longer buy cannabis in the pharmacy.

</td></tr>
<tr><td>10`</td><td>
Possible points of sale (ppp slides 25-27)

In addition to pharmacies, which points of sale do you see for selling cannabis products?

o Where would you go or where would you definitely not go?
o Why yes/no?
o What type of retail outlet would you definitely not consider?

What would appeal / suit you?

</td></tr>
</table>

15`	***Challenges from the participants' perspective (ppp slides 28-29)*** Now you have received a lot of information about cannabis products, packaging, devices, possible interventions in the pharmacy and the study itself.<br>• Is there anything you say you need to look at? That could be tricky for people who use cannabis?<br>• Is there anything you've just noticed that you'd like to share with us?<br>• How do you generally rate the current range (products, packaging, price, devices, interventions)?<br>o What appeals to you?<br>o What do you find rather critical?<br>o What could be different from your perspective? How?
5`	***Outlook (ppp slides 30-31)***<br>It is planned to hold another monitoring group meeting. This will probably be Sept. or Oct 2022.<br>Present possible topics

Laddering technique:

In-depth questioning with:

- Why is this important to you?
- What does this mean to you?
- What do you associate with it?
- What would you miss if xy were not there? What would you miss then?
- How does xy make you feel?
- What do you expect because of ...?

Evaluation of the group discussion of the SCRIPT participatory support group

The group discussion was conducted with people who use cannabis

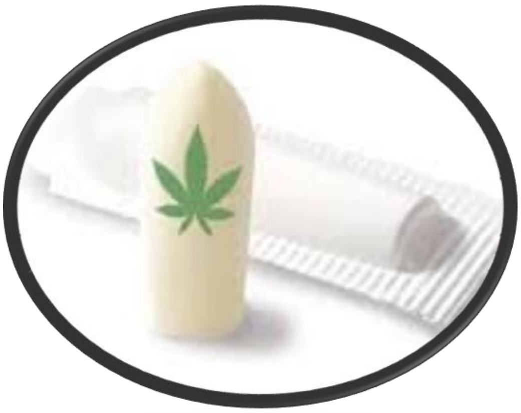

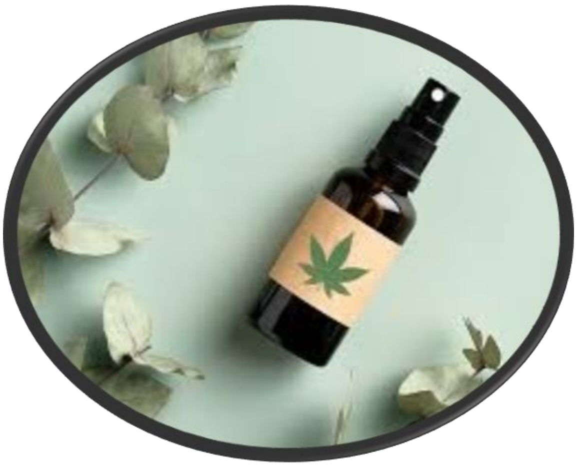

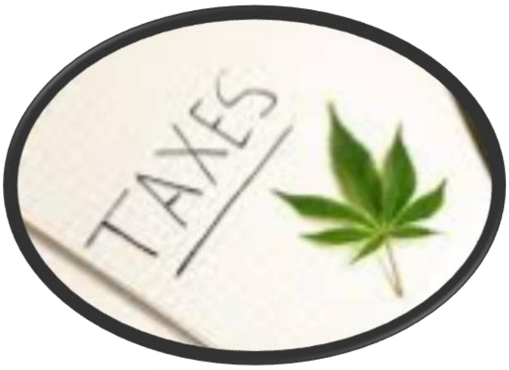

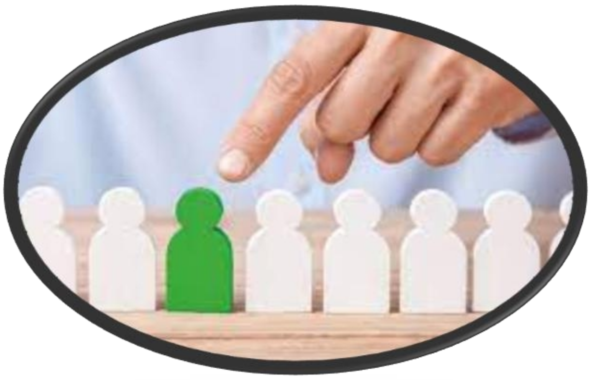

Beatrice Metry

Bern Institute of Family Medicine March

2023

Table of contents

1 Introduction 3

2 Methodological approach 4

2.1 Clarification of competence at the Cantonal Ethics Committee of Bern 4

2.2 Survey and evaluation procedure 4

2.3 Sample 4

3 Results from the group discussion 6

3.1 Reactions to further cannabis product ideas 6

3.2 Profit-oriented versus non-profit-oriented cannabis sales market 7

3.3 Advertising for cannabis products 10

3.4 Use of cannabis taxes 11

3.5 Possible cannabis points of sale 12

3.6 Reactions to the selection criteria for study participants 14

3.7 Outlook 14

4 Conclusion 15

5 Appendix 1: Group discussion questionnaire 16

1 Introduction

In the fourth quarter of 2021, individual interviews were conducted with the nine members of the SCRIPT study's participatory support group. In July 2022 and February 2023, the group met once each for a group discussion on the premises of the University of Bern. The individual interviews and this group discussion were analyzed and the results presented in a report.

To date, the following reports have been produced and are available:

- Evaluation of the individual interviews on SCRIPT from March 2022
- Evaluation of the group discussion on SCRIPT from July 2022
- Evaluation of the group discussion on SCRIPT in March 2023 (present report)

The participatory support group for the SCRIPT study met for the second group discussion on February 13, 2023. Of the initial nine members of the participatory support groups, five cannabis users were present this time, who discussed the following topics under the moderation of Beatrice Metry (research assistant):

- other cannabis products (mouth spray, suppositories, creams)
- Profit-oriented versus non-profit-oriented market
- Cannabis sales market options
- Selection of study participants

The evaluation report outlines the sample and is then presented to the subjects according to the questionnaire, which can be found in the appendix. The questionnaire can be found in the appendix.

2 Methodological approach

The various parts of the procedure are described below. All interviewees signed an informed consent before the individual interview (2021) and agreed to the interview and the subsequent group discussion and its evaluation by signing it. This assured them of anonymity.

2.1 Clarification of competence with the cantonal ethics committee of Bern

On May 31, 2021, the concept for the qualitative accompanying research entitled "Use of a participatory support group consisting of cannabis-using adults as a supplement during the planning phase of the SCRIPT 2 project" was submitted to the Cantonal Ethics Commission (KEK) to check its competence. The CEC found that it was not responsible. This means that this research does not fall under Article 2, paragraph 1 of the Human Rights Act and that individual and group interviews with the selected target group can be conducted without submitting an application. After this decision[1]of June 20, 2021, the acquisition of participants was started.

2.2 Data collection and evaluation procedure

The data was collected through a group discussion along a defined question route over a period of two hours, digitally recorded and then transcribed.

The transcript was analyzed using MAXQDA software. The first step involved coding the text, followed by condensing the individual statements and writing down the results.

2.3 Sample

All nine people who had already taken part in the individual interviews and signed the corresponding informed consent were requested for the group discussion. The appointment was arranged via doodle.
One group member canceled their participation, as this person is studying abroad for a year and is therefore unable to attend the meetings. Two people canceled at short notice and one person made a mistake with the date and therefore did not show up. As a result, five cannabis users plus the moderator were present at this group discussion. The following table provides an overview of the sample.

[1]BASEC no. Req-2021-00609, decision on the clarification of responsibility is available from 20.6.2021

	Individual interviews 4th quarter 2021	Group discussion 1 July 2022	Group discussion 2 March 2023
**Number of persons**	9	6	5
**Year of birth**	1957, 1975, 1990, 1991, 1993, 1999, 2000, 2001, 2000	1957, 1975, 1991, 1993, 2001, 2002	1957, 1991, 1993, 2000, 2002
**Sector Occupation**	Studies (3)<br>Logistics (2)<br>Finance (1)<br>Photography (1)<br>Graphics (1)<br>Fitness (1)	Study (1)<br>Logistics (1)<br>Finance (1)<br>Photography (1)<br>Graphics (1)<br>Fitness (1)	Study (2)<br>Finance (1)<br>Graphics (1)<br>Fitness (1)
**Gender**	female 3<br>male 5<br>non-binary 1	female 2<br>male 3<br>non-binary 1	female 2<br>male 3<br>non-binary 0
**Nationality**	Switzerland 9	Switzerland 6	Switzerland 5

3 Results from the group discussion

The results are listed below along the question route. Numbers in round brackets indicate how many respondents answered in this direction. If there are no brackets after the statement, it is an individual answer. The paragraph in the MAXQDA file (A) in which the quote can be found is listed in square brackets.

3.1 Reactions to further cannabis product ideas

Suppositories, mouth spray and (mucus) skin cream were presented to the group as further cannabis product ideas. Three people first expressed surprise at these ideas and then said that these products lacked the sensuality of rolling and smoking. Other initial reactions were: the rapid absorption of the active ingredients via the mucous membranes; the more potent effect than smoking; fewer side effects, as no smoke waste products are inhaled. Another voice said that mouth spray, suppositories and creams conceal cannabis use. The last statement is underpinned by the following quote:

> I have the feeling that you can be very incognito with these forms of intoxication. When you smoke pot, you can smell the smoke. It's conspicuous. And otherwise I have the feeling that you can spray or take a suppository and nobody is looking. It's something where I feel like you can keep it better hidden. [A:58]

The participants agreed that they would try a cannabis mouth spray (5). The mouth spray was well received because it was effective and easy to dose (4), as the following quote shows:

> Because with LSD, I think the spray is brilliant. So the effective dosing. 20/40 or how much. Simply that you can dose it precisely. And so today, when this weed is just so strong and the dosage is really tricky, I find that a problematic issue. [A:32]

Regarding the dosage of the mouth spray, two people stated that one dose would be sufficient. Consumption could be regulated well with the number of puffs. Another person pointed out that even with a cannabis mouth spray, each person reacts differently to the cannabis active ingredients and this must be taken into account when consuming.

For one person, it is clear that mouth spray is odorless. Another said that if this product was odorless, it could be a good travel companion.

> Because, of course, it would solve a certain problem you have when traveling. If you have something that doesn't smell at all. Then this could be the companion when traveling. [A:63]

In contrast, another voice said that smuggling was not the idea of new cannabis products. Four people would try the (mucilage) skin creams. One voice expressed interest in a nasal ointment as another product idea.

Suppositories as a form of administering cannabis gave rise to discussion in the group. On the one hand, several people said that suppositories were associated with being ill (2) or were not part of recreational use. This is substantiated by the following quotes:

> Yes. For me, suppositories have a very negative connotation with being ill. [A:42]
>
> Suppositories are unnecessary for me, actually. So I only see the medical background. It's not practical for pleasure consumption, if I can put it that way. [A:45]
>
> I've now imagined the stoner round. Instead of passing a joint around, you give each other a suppository. [A:24]

In general, participants (3) said that these product ideas would appeal to a new target group. They mentioned people who had not previously smoked or smoked pot or who would like to switch to a healthier form of consumption. The following statement underlines this:

> But I also think for someone who doesn't know it and would like to try it, and doesn't want to damage their lungs because they've never smoked before, then a spray like that...// So that person would be more likely to respond to a spray or a suppository or I don't know what. But now the 0815 stoner is simply, as already mentioned, we like to be with our joints and our rolling and all that. [A:27]

One of the interviewees said that she thought it was time to start implementing the study before other forms of consumption were developed. This person formulated her statement as follows:

> 'After six years now, discussing whether or not you are allowed now. And there is an extra article in the law with an experimental article [...] I have the feeling that I would rather smoke or vaporize now than have x different consumption variations. [A:24]

There are also critical comments on the other forms of administration. For example, one person expressed concerns that mouth spray could invite increased consumption due to its discreet intake and constant availability, as the following quote shows:

> But that can also backfire. Because if you have a spray with you that's always smiling at you, where you can always take it out quickly and spray without anyone noticing. It can also go the other way. [A:37]

Another voice goes in a similar direction and adds that the above-mentioned properties of the products could lead to hidden addictive behavior.

> I have the feeling [...] with these forms of intoxication [...] you can be very incognito. When you smoke pot, you can smell the smoke. It's conspicuous. And otherwise I have the feeling that you can spray or take a suppository and nobody is looking. [A:58]

3.2 Profit-oriented versus non-profit-oriented cannabis sales market

The participants were asked what type of sales market they would generally see for cannabis products. The profit-oriented and non-profit-oriented markets were presented. The basic tenor of those present was against capitalism for the cannabis market. Here are a few quotes to back this up:

> Wherever there is a gold-rush atmosphere, people suddenly only see the positive. Perhaps also the dangers that come with it, because there are risks everywhere. Yes, you milk the cow until it dies. [...] For me personally, hemp stands for something else. It stands for slowing down. And not for the carousel spinning faster and faster and the stress increasing. [A:124]
> Yes, one of the biggest fears, actually, is that it's always just about making more money with it. And that everything becomes more expensive. [...] And, yes, this profit-oriented thing, that something that's beautiful for me will be taken away from it afterwards. [A:85]

> Even if I don't think it's cool that cannabis is so commercialized, because I don't think it corresponds to the nature of the plant. It's a weed that grows everywhere and to charge money or a lot of money for it afterwards, I think that's moral, yes, that cuts it. [A:89]

> There will always be people who will make a profit from something. You can't deny that. And it was the same when they legalized CBD. [A:91]

On the other hand, one voice says the following:

> Even if the whole capitalism and everything is shit, the fact is, if there is profit, then there is acceptance. And the ultimate goal is for it [cannabis] to be accepted. [A:92]

Another voice said that where profits are made, everyone is happy and this creates acceptance in society. This acceptance (3) was seen as an advantage of the profit-oriented market. The open approach to cannabis advertising, for example, also creates a different image and leads to acceptance in society.

Another person mentioned the competition on the market as a positive effect. This would also put pressure on the black market and several people present thought this was a good argument (3) in favor of the profit-oriented market. Below are two quotes on this:

> To which I also see in variant 1 [profit-oriented market] the whole black market, which is more likely to collapse if you do it this way. [A:92]

> On the one hand, a capitalist market would certainly depress the price. Because there is simply a competitive mindset, I'll say that now. [A:89]

Another advantage of the profit-oriented market was seen by one person present in the creation of jobs in various areas (cultivation, production, advertising, sales). These jobs are important for our society.

The non-profit-oriented approach, two people said, was more useful for medical products. When it comes to enjoyment, "you should go all out and really go for it" [A:127], said one person.

Regulated prices were mentioned as a clear disadvantage in the non-profit market (4). As the prices are set by the state, they are overpriced and the black market can continue to flourish. One person stated that the regulated market would only partially meet the wishes of consumers, which she formulated as follows:

> I think if you want to make it strictly regulated, then it's like the principle of giving the little finger and you take the hand. Because it is simply allowed a little, but not to the extent that is desired. That is perhaps another critical point. I do believe that it is a big step, but you have to make a conscious decision to take the big step and not the small one. [A:182]

The consideration of social institutions and projects was seen as an advantage in the non-profit market (2). Two people stated that they were convinced that it would become a regulated market in Switzerland, as the tobacco market is also heavily regulated.

Those present said that a hybrid of the two market approaches would be conceivable in the future. A levy for prevention, addiction treatment or psychiatric therapies (3) should definitely be envisaged.

> And I have the feeling that such a hybrid form would be something cool. Even if it were capitalistic, you could say that 10 or 20% of sales would go towards addiction prevention. [A:89]

> I also think a mixed form is the only good solution. [A:91

> It's exciting for me now because I'm quite anti-advertising. And it was really exciting to hear this approach. And yes, it definitely makes sense for us to think differently. I can also see the disadvantages of the non-profit approach. But I think we need to find the right balance between the two. Because I can't say fix this or fix that. It's certainly an issue that will go on for a bit longer, where I have the feeling that we still have to find the right way. [A:147]

Another person said that they thought the term "profit-oriented" was wrong, and that a *market orientation* should be pursued, as one of the aims was to eliminate the black market. This person explains their thoughts as follows:

> I wouldn't phrase it as "profit-oriented", I would say "market-oriented". Because one of the goals is to eliminate the black market. In other words, if I am an entrepreneur and now produce cannabis, then I can't just say that I want to earn a lot and that's expensive. I have to get rid of the black market. In other words, I have to lower the price a lot at times in order to achieve this. It's as if one gangster gang is fighting against another. [...] So, in this sense, it wouldn't be profit-oriented for me, but the market approach. How the market works. And in that sense, I can't say it's made up of this, this and this and that gives the price. Rather, the price is defined solely by the question: How do we get rid of the black market? I have the feeling that should be the approach, because that is the goal. [A:136]

The following are general statements on the future cannabis market in Switzerland.

> I have the feeling it's probably like an evolution, ultimately. With regulation and decriminalization, legalization will come one day. But even there, Switzerland is conservative. There's still a lot of water going down the Aare before we smoke the first joint with this legal organic hemp.

The need for a solution at federal level was expressed in order to prevent cannabis tourism between the cantons.

Home cultivation was also addressed. This should be permitted if the market is opened up for personal use (4). This with the following justification:

> The oldest excavation in Switzerland is near Egolzwil, near Wauwil. They found hemp seeds there. So, the oldest things in our area are hemp seeds. So that really is an ancient cultural asset from here. I think that's important. [A:97]

3.3 Advertising for cannabis products

Those present were asked whether cannabis products should be advertised. Three people were rather critical of this. One person feared an increased ostracism of cannabis users in society. She expressed this as follows:

> That everyone can get upset about it afterwards. Like this: Yes, now they're advertising it. That can only get worse. [A:85]

Another person commented that, in principle, cannabis products should not be advertised, as the following quote shows:

> I don't think cannabis should be advertised. I don't think you should advertise alcohol either. [A:198]

And the third person said that smoking weed is a way of life and should not be advertised. Likewise, any cannabis advertising cuts across the current requirements for tobacco and alcohol advertising.

> I think for many people it's just a trial. But smoking weed is a way of life. I'm also critical of advertising in general. Because I have the feeling that it intersects with alcohol and tobacco. [A:188]

Furthermore, one of these three people praised the mouth-to-mouth propaganda as a well-functioning advertising medium, as the following quote well underlines:

> 'But in the end I think mouth-to-mouth propaganda is already a good advertisement and one that works really well.' [A:202]

Another person stated that it would make sense for cannabis advertising to be subject to the same regulations as alcohol and tobacco advertising. And yet another voice thought that the supply of clean cannabis in particular should definitely be advertised, as the following quote shows.

> So I think that this should definitely be advertised. [...] So, if you tell a halfway decent stoner afterwards what's happening on the black market, and you don't want that. You have to do it differently. People buy Havelaar products.
> Arguments work. Many people listen to arguments. Not all, but many. And in that sense, to say what the consequence is if you buy it on the black market. [A:207]

The same person explained that cigarette advertising also aims to get consumers to switch brands and is not about turning non-smokers into smokers.

3.4 Use of cannabis taxes

It was clear to everyone present that sooner or later a tax would be levied on cannabis products. The cannabis tax was linked to the tobacco and alcohol taxes.
Two people said that the tax should be distributed across all three political levels (municipality, canton, federal government). One vote favored the municipality, as it also approves the infrastructure and this is where the effect of the tax is most visible. The use of tax money was seen by those present in addiction prevention, youth work, social institutions and emergency psychiatry. The following quote gives an idea of what this could look like:

> So, shooting from the hip, if you say 20% of revenue is given as tax. You could make a breakdown. A quarter of this 20% goes to prevention, a quarter goes to emergency psychiatry or other

> psychiatry, a quarter goes to the AHV and a quarter goes to the other social services. And then you could look at how it is used. If you realize that not much is needed in psychiatry, only a fraction, then you could also shift it. [A:265]

3.5 Possible cannabis sales outlets

The ideas for further sales outlets - in addition to pharmacies - are varied in this round. What emerged clearly is that a foundation that is associated with hard drugs or methadone dispensing is not an option for those present. On the Bern market, this means that the Contact Foundation would not be accepted for the sale of cannabis products by cannabis users, which is underlined by the following quote:

> 'So I find anything that [has the] origin of hard drugs, I find an absolute no-go! Because this bullshit claim that it's a gateway drug simply feeds. And there really is zero tolerance. Just not. [A:214]

> Yes, exactly. Because that's one of the big lies and we don't need to feed it. So really not. [A:216]

The cannabis offer would fit in well with the organic stores, those present thought (5). These are in the neighborhoods, close to the customers and familiar to the people, which was seen as an advantage. Organic stores often have specialized staff for each department. This could be the same for the cannabis corner and would mean that one person would undergo two to three days of training. Lateral entrants without a Federal Certificate of Competence (EFZ) could also be considered for this. The following quotes support these statements:

> Then it could also be that if someone hears or sees a poster in the Haller store saying "Cannabis can be bought here" or something like that. Then you think: "Aha, you can get it here". But it's not just next to the vegetables and you can put it in a plastic bag and weigh it. It should be at the cheese stall or something like that, where you can say: "I'd like to buy some grass". And then the first question would be: "Have you ever bought from us before?" And if so, then: "Sure, which one, how much?" And if it's the first time, then there's a short introductory talk. What you have (in the range). [A:235]

> And maybe you have two or three days. For example, like when you're learning to drive. You do the emergency helper. You go there for two days, have a few hours of input, fill something out at the end and then you're good. And I don't even think it's important to have an EFZ, just this training. Then you do this training course, which lasts two days, and then it's fine for me. In terms of the material

> two days, it's quite possible to educate a person so that they can educate other people. [A:316]

> There are also people who have an idea about it. It doesn't have to be someone who has an EFZ qualification. Exactly, I know people who know a lot about weed, from cultivation to I don't know what, but no other training. And I mean, yes, if you can deal with people a bit afterwards...// Look in another place and put out an advertisement like this: "Hey, are you extroverted and can deal with people and do you know anything about cannabis?" But that you can also create jobs for people who wouldn't have a chance in another place. But they thrive there. Yes, of course, they have to go to class for another day and then they know what's good and what's not good. But you also have to give other people a chance. Even career changers. [A:329]

Further information could be offered to customers with a display (brochures, touchscreen or QR code), added another person, the organic store offer.

The self-marketing of cannabis products by the organic farmers themselves also met with approval. One idea that met with approval among those present was the 1st August brunch, at which the cannabis products offered by the farm could be presented (3). The idea of expanding the range in the head and grow stores was equally well received (3). The specialists are already at work there and accessories can be purchased. The cannabis clubs presented (similar to the social clubs in Spain) also received a positive response (3).
The following quote illustrates the idea of these local cannabis clubs:

> Yes, it goes a bit in that direction. From the region for the region. That there is a club in which people are active members and can also bring people in. And they can then buy their cannabis at their own price. Then there is a harvest every three months and then you have 1 gram for 3-4 francs. And in the end, that's also a cheap option. I have the feeling that people don't like being patronized, especially with narcotics. [A:140]

Association statutes would regulate who is allowed to purchase how much cannabis. Active membership would be compulsory. Other ideas were to include tobacco stores and kiosks in the sale of cannabis. Or to open cannabis bars where cannabis could be bought and consumed. Using cannabis bars as a sales market would have the advantage of being open in the evenings and at weekends.

One need expressed by those present was to establish cannabis smoking zones (3). It should be possible to smoke cannabis products there. Comparable to the smoking areas at airports or train stations.

3.6 Reactions to the selection criteria for the study participants

There were few reactions to the selection criteria that define who is allowed to take part in the study. One person said that the figures 55% male, 40% female and 5% other was an unfair distribution, as more men than women were included. Another person said it was okay if these figures reflected the proportion of people who smoke weed in our society. And a third person said that the parameters appeared sober and understandable, which was ok.

The participants were more interested in whether they could safely take part in the implementation study than in the parameters, as they had been assured of this when they signed up to join the participatory support group. The participants were assured by the moderator that this was the case and that they would be allowed to take part in the implementation study.

3.7 Outlook

A next monitoring group meeting is planned for May 2023. In the time in between, participants could be given something to read and give feedback on. The question was whether they would be willing to do this. All five people agreed. All five people would also have a computer on which they could read the documents and write notes directly. None of those present wanted a printout of the documents.

4 Conclusion

The participants in this support group meeting expressed curiosity about further cannabis product ideas. They were particularly interested in the mouth spray and creams for mucous membranes. They stated that they were concerned that this form of consumption could take place in concealment, unlike smoking, making addictive behavior less obvious. With regard to the market form, the interviewees mentioned some positive aspects of the profit-oriented market, such as that it is more effective when it comes to weakening the black market and gaining social acceptance of cannabis use. A mixed form would be realistic. In this case, 10-20% of sales should be given to social causes (emergency psychiatry, addiction prevention, etc.). The best advertising for cannabis products is word-of-mouth propaganda. Further advertising is rather unrealistic, as tobacco and alcohol may not be advertised either. The organic store in the neighborhood was highlighted as another cannabis outlet. Head or grow stores, cannabis bars, tobacco stores and kiosks are also conceivable. The participants did not consider foundations or places associated with hard drugs as sales outlets.

The next monitoring group meeting is scheduled for May 2023. In the meantime, the participants are prepared to read information letters, flyers etc. and provide written feedback. All attendees waive a printout by post, they would read and process the documents electronically.

.

5 Appendix 1: Questionnaire for group discussion

Preparation of support group for cannabis users SCRIPT

February 6, 2023 / 18:30 - 20:30

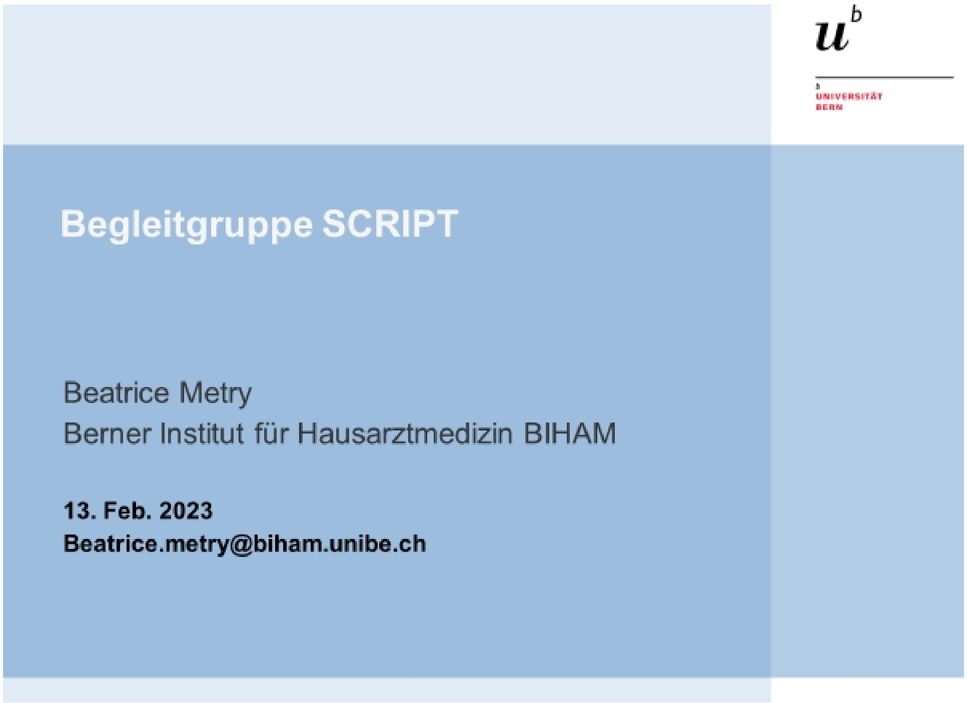

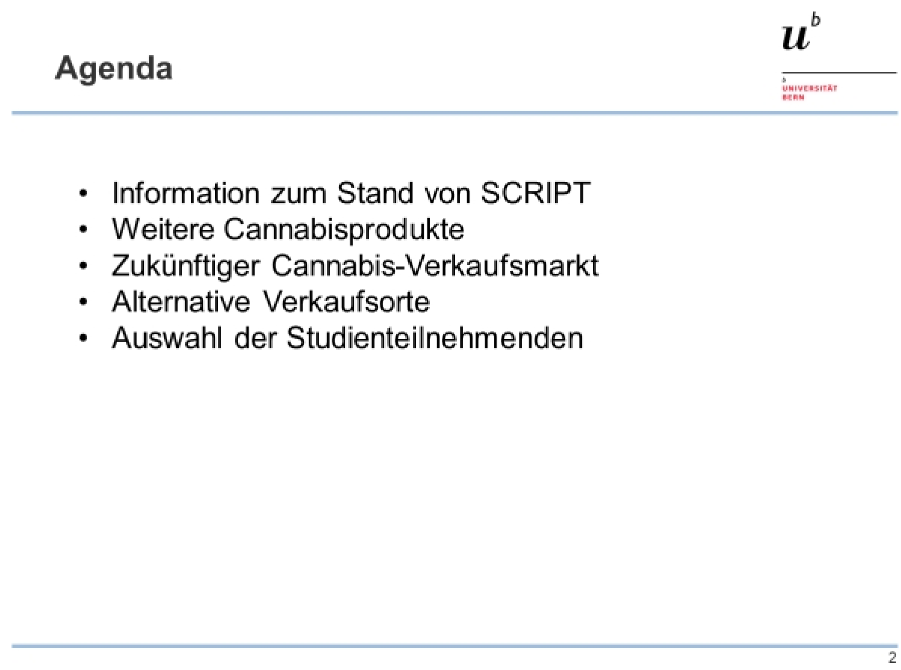

English translation
Agenda
• Information on the current status of SCRIPT
• Additional cannabis products
• Future cannabis sales market
• Alternative points of sale
• Selection of study participants

Informationen zu SCRIPT

Der Bewilligungsprozess ist am Laufen
- Der Kanton Bern unterstützt Studie nicht und lehnt Abgabe Cannabis grundsätzlich ab.
    - Genügend Studien um Entscheid treffen
    - negatives Signal für die Jugendlichen

Gespräch mit Kanton wird gesucht
Voraussichtlicher Beginn in Bern im Spätsommer 2023
Bewilligungsprozess Versuche in den Kantonen Zürich, Genf und Waadt laufen.

Cannabisproduzent kann erst ab April aussäen, die Ernte wird im Spätsommer 2023 erwartet.
- Gras wird von Beginn an zur Verfügung stehen
- Harz wird wahrscheinlich später kommen

3

Information on SCRIPT
The approval process is ongoing.

The Canton of Bern does not support the study and generally rejects the distribution of cannabis.
There are enough studies available to make a decision.
Negative signal for youth.
Talks with the canton are being sought.
The expected start in Bern is late summer 2023.
Approval processes are also underway in the cantons of Zurich, Geneva, and Vaud.
The cannabis producer can only begin sowing in April, and the harvest is expected in late summer 2023.
• Cannabis flower will be available from the beginning
• Resin will likely come later

Bereits geplante Cannabisprodukte

4x Cannabisblüten

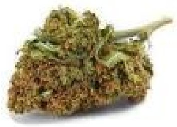

EN: cannabis flowers

2x Cannabisharz

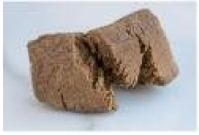

EN: 2x cannabis resin

2x Cannabisöl

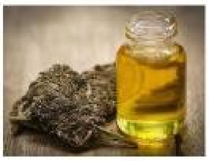

EN: 2x cannabis oil

2x Cannabis E-Flüssigkeit

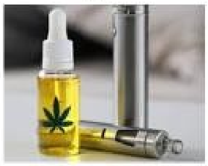

EN: 2x cannabis e-liquid

4

Zusätzliche Cannabisprodukte

Mundspray

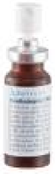

EN: oral spray

Crème für die Haut oder Schleimhaut (z.B. nasal, vaginal, anal) oder Zäpfchen

EN: English translation
Cream for the skin or mucous membranes (e.g. nasal, vaginal, anal) or suppositories

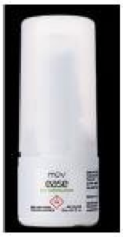

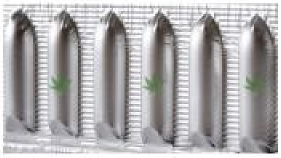

5

Fragen

Was kommt Ihnen spontan in den Sinn, wenn Sie daran denken, diese Produkte bei uns erwerben zu können?

Würden Sie solche Produkte kaufen? Welche?

Was müsste sein, dass Sie diese Produkte kaufen würden?
Für welche Gelegenheit würden Sie solche Produkte kaufen?

Was würden Sie garantiert nicht kaufen? Weshalb?

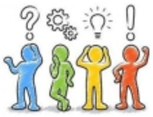

6

English translation
Questions
What comes to mind spontaneously when you think about being able to buy these products from us?
Would you buy such products? Which ones?
What would have to be the case for you to buy these products?
For what occasion would you buy such products?
What would you definitely not buy? Why?

Zukünftiger Cannabismarkt

Variante 1: gewinnorientiert, kompetitiver Markt

Viele Produktionsbetriebe
Produktevielfalt
Werbung, Aktionen, Promotionen
Ziel = Marktsteigerung, illegaler Markt schnell dämpfen
Diverse Verkaufsstellen
Preisdruck
Gewinne für die Betriebe und deren Aktionäre/ Inhaber
Zunahme von Konsumierenden

7

English translation
Future Cannabis Market
Option 1: Profit-oriented, competitive market
Many production businesses
Wide variety of products
Advertising, special offers, promotions
Goal = market growth, quickly suppress the illegal market
Various points of sale
Price pressure
Profits for businesses and their shareholders/owners
Increase in number of consumers

Zukünftiger Cannabismarkt

Variante 2: nicht-gewinnorientiert, strikt regulierter Markt

Produktionsfirmen benötigen eine (kantonale/ eidgenössische) Zulassung/ Lizenz
Produkte sind vordefiniert
Bestimmte Verkaufsstellen
Preise sind vergleichbar/ gleich
Keine Werbung
Gewinne werden in Suchtprävention und zu weitere Zwecke für «community» investiert

8

English translation
Future Cannabis Market
Option 2: Non-profit, strictly regulated market
Production companies require a (cantonal/federal) permit/licence
Products are predefined
Certain authorised points of sale
Prices are comparable/ identical
No advertising
Profits are invested in addiction prevention and other purposes for the community

Zukünftiger Cannabismarkt

UNIVERSITÄT BERN

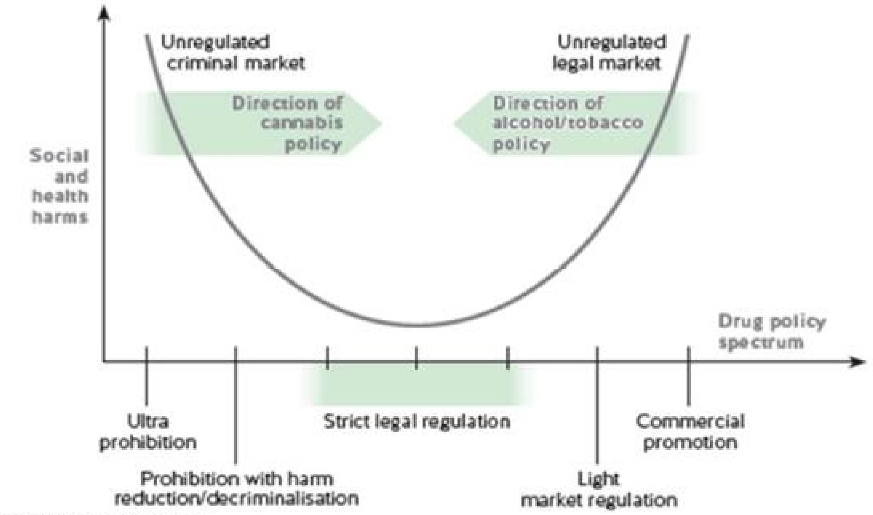

Transform 2013, Marks 2008

9

Zukünftiger Cannabismarkt

UNIVERSITÄT BERN

Variante 2: nicht-gewinnorientiert, kompetitiver Markt

Studie verfolgt einen ***nicht-gewinnorientierten, strikt regulierter, Ansatz***.

Den Preis, den ihr für das Produkt bezahlen werdet, deckt

1. Den Aufwand der Apotheken,
2. Den Aufwand für die Herstellung
3. Der restliche Betrag fliesst zurück in die Studie (z.B. um Gutscheine zur Aufwandsentschädigung der Teilnehmenden kaufen).

10

English translation
Future Cannabis Market
Option 2: Non-profit, competitive market
The study follows a non-profit, strictly regulated approach. The price you will pay for the product covers:

The effort and costs incurred by the pharmacies

The cost of production

The remaining amount flows back into the study (e.g. to buy vouchers as compensation for participants)

Fragen mit Fokus auf den Verkaufsmarkt

UNIVERSITÄT BERN

- Welches sind eure ersten Gedanken, wenn ihr diese Marktvarianten seht?
- Was wäre für euch der optimale zukünftige Cannabis Markt?
- Wo seht ihr in ***gewinnorientierten Ansätze*** Vor- und Nachteile?
- Wo seht ihr in ***nicht-gewinnorientierten Ansätze*** Vor- und Nachteile?
- Was spricht euch an? Weshalb?
- Was denkt ihr wo wird sich der Schweizer Markt einpendeln?

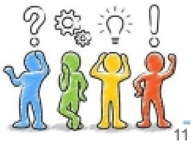

11

English translation
Questions focused on the sales market
• What are your first thoughts when you see these market options?
• What would be the ideal future cannabis market for you?
• What do you see as the advantages and disadvantages of profit-oriented approaches?
• What do you see as the advantages and disadvantages of non-profit approaches?
• What appeals to you? Why?
• Where do you think the Swiss market will eventually settle?

Fragen mit Fokus auf den Verkaufsmarkt

UNIVERSITÄT BERN

- Was denkt Ihr, soll Werbung erlaubt sein?
  - Wenn ja, in welcher Form?
  - Wo?
    - Plakate an Strassenränder
    - Fernsehspots
    - Kino
    - Verkaufsstellen
    - Spezifische Zeitschriften
    - Online (social media, influencer, websites, …)

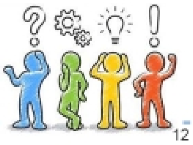

12

English translation
Questions focused on the sales market
• What do you think — should advertising be allowed?
• If yes, in what form?
• Where?
• Posters along roadsides
• TV commercials
• Cinema
• Points of sale
• Specific magazines
• Online (social media, influencers, websites, etc.)

Fragen mit Fokus auf den Verkaufsmarkt

- Wer soll im Verkaufsmarkt aktiv sein?
  - Apotheken, Cannabisläden, Online Shops, Kiosk
  - Eine Stiftung, ein Verband, andere Non-Profit-Organisationen
- Auf welcher Ebene sollen Steuern erhoben werden?
  - Bund, Kanton, Gemeinde
- Für was sollen die erhobenen Steuern eingesetzt werden?
  - Prävention, Betreuung von Personen mit einer Cannabissucht, soziale Werke, AHV, …

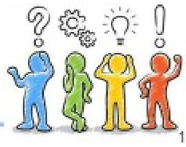

13

English translation
Questions focused on the sales market

- Who should be active in the cannabis sales market?
- Pharmacies, cannabis shops, online shops, kiosks
- A foundation, an association, other non-profit organisations
- At what level should taxes be collected?
- Federal, cantonal, municipal
- What should the collected taxes be used for?
- Prevention, support for people with cannabis addiction, social services, pension system (AHV), etc.

Alternativer Verkaufsort

Alternativ zu den Apotheken wird eine weitere Verkaufsstellenmöglichkeit evaluiert.

Im Moment ist die Stiftung Contact Bern im Gespräch
Es werden separate Cannabis-Verkaufsstellen sein

Die SCRIPT-Studie hat Interesse zu ergründen, ob, wer und wann diese alternativen Verkaufsstellen aufgesucht werden. So kann ein Vergleich mit den Apotheken gemacht werden.

14

English translation
Alternative Point of Sale
An additional point of sale is being considered as an alternative to pharmacies.
Currently, the Contact Foundation Bern is under discussion.
These will be separate cannabis sales locations.
The SCRIPT study is interested in exploring whether, who, and when these alternative sales points are used. This will allow a comparison with pharmacies.

Fragen

Wie steht ihr zu einer alternativen Verkaufsstelle?

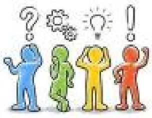

Was kommt euch in den Sinn zu:

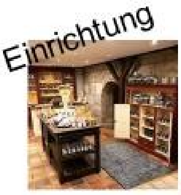

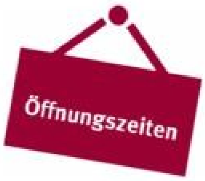

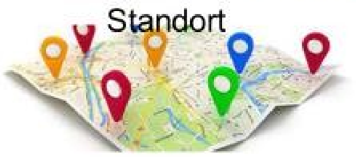

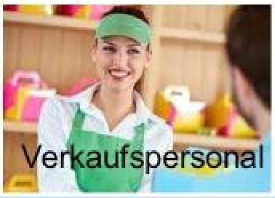

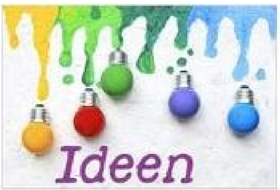

15

English translation
Questions
What are your thoughts on an alternative point of sale?
What comes to mind when you think of:

Alternativer Verkaufsort

- Gibt es im Vergleich zu den Apotheken eine Reihenfolge, welche anderen Verkaufsstellen ihr bevorzugt?
  - Welche Reihenfolge?
  - Was sind die Gründe für diese Wahl?

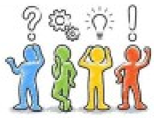

16

English translation
Alternative Point of Sale
• Compared to pharmacies, is there an order of preference for other sales locations?
• What is that order?
• What are the reasons for your choice?

Auswahlverfahren Studienteilnehmende

u^b UNIVERSITÄT BERN

- Es werden viele Anmeldungen erwartet
- Aussagekraft der Studie verbessert sich, wenn die Charakteristika der Teilnehmenden auf die ganze Breite verteilt sind

Überlegung zu Charakteristika

Gender 55% m, 35% w, 5-10% div.
Alter ab 18, gegen oben offen. Anzahl verteilt auf das ganze Altersspektrum
Konsumverhalten täglich oder nicht täglich
Wohnhaft in der Stadt Bern →Anmeldungen aus anderen Gemeinden kommen auf die Warteliste
Registrierung ist ab Sommer 23 möglich, Webseite wird bekannt gegeben

17

English translation
Selection Process for Study Participants
• A high number of registrations is expected
• The study's validity improves if participant characteristics are spread across the full spectrum
Consideration of characteristics:
Gender: 55% male, 35% female, 5–10% diverse
Age: from 18 upwards, with numbers evenly distributed across all age groups
Cannabis use: daily or non-daily
Must live in the city of Bern
Registrations from other municipalities will be placed on a waiting list
Registration will be possible from summer 2023; the website will be announced

Frage

u^b UNIVERSITÄT BERN

Was sagt ihr zum Auswahlverfahren?

Wie wirken die Kriterien auf euch?

Was erwartet ihr?

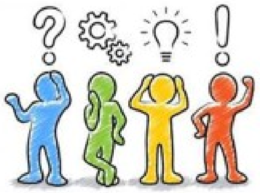

18

English translation
Question
What do you think about the selection process?
How do the criteria affect you?
What are your expectations?

Ausblick

Die nächste Sitzung ist für den ***Mai 2023*** vorgesehen

Seid ihr bereit zwischenzeitlich ein Informationsschreiben und / oder einen Flyer zu lesen und eine Rückmeldung dazu zugeben?

Wer von euch hat die Möglichkeit, dies elektronisch zu tun?
Wer benötigt einen Ausdruck?

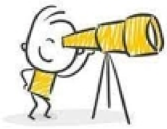

19

English translation
Outlook
The next meeting is planned for May 2023.
Are you willing to read an information letter and/or a flyer in the meantime and give feedback on it?
Who among you is able to do this electronically?
Who needs a printed copy?

Abschluss

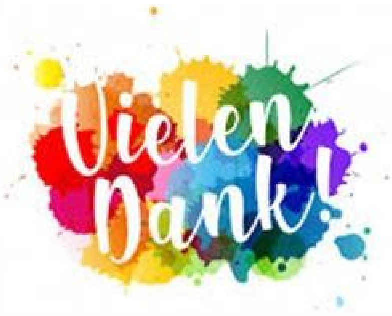

20

English translation
Conclusion
Thank you!

Laddering technique:

In-depth questions with:

- Why is this important to you?
- What does it mean to you?
- What do you associate with it?
- What would you miss if xy were not there? What would you miss then?
- How does xy make you feel?
- What do you expect because of ...?

Evaluation of the group discussion of the SCRIPT participatory support group

The group discussion was conducted with people who use cannabis

SCRIPT
The Safer Cannabis – Research In Pharmacies randomized controlled Trial

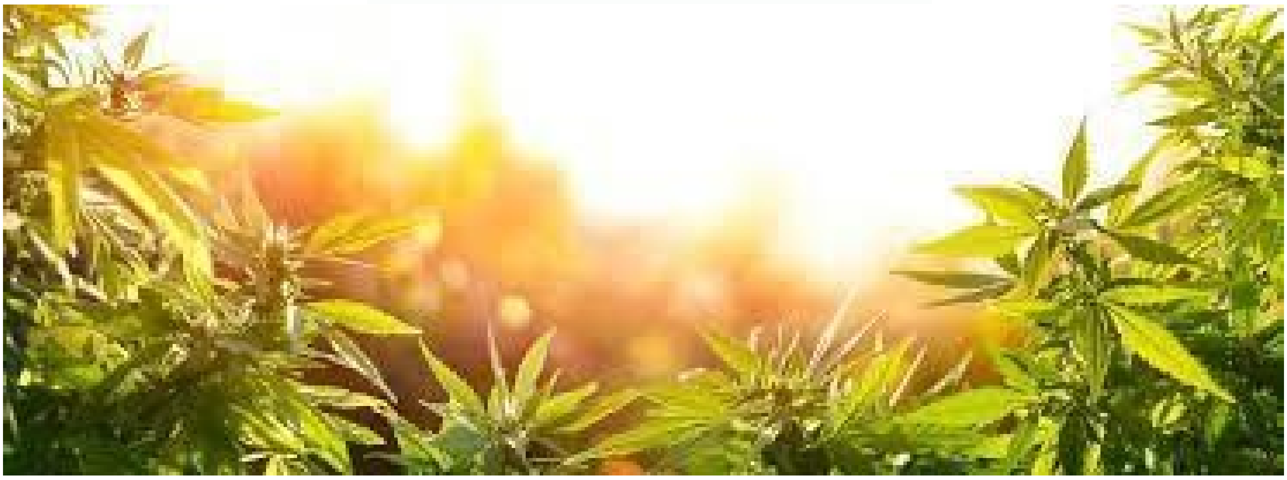

Beatrice Metry

Bern Institute of Family Medicine June 2023

Table of contents

1 Introduction 3
2 Methodological approach 4
2.1 Clarification of competence at the Cantonal Ethics Committee of Bern 4
2.2 Survey and evaluation procedure 4
2.3 Sample 4
3 Results from the group discussion 6
3.1 Study information and informed consent 6
3.1.1 Comprehensibility of the study information and informed consent form 6
3.1.2 Statements on the contents of the study information and informed consent form 8
3.1.3 Study participation 8
3.2 Stop smoking advice in the pharmacy 10
3.3 Case Report Forms 12
3.4 Website script-study.ch 14
3.5 Information and perspective for the monitoring group 15
4 Conclusion 17
5 Appendix 1: Group discussion questionnaire 18
6 Appendix 3: Decision aid for the treatment method to support smoking cessation 21
7 Appendix 2: Study information and informed consent form 23

1 Introduction

In the fourth quarter of 2021, individual interviews were conducted with the nine members of the participatory support group of the SCRIPT study. In July 2022 and in February and June 2023, the group met once each for a group discussion on the premises of the University of Bern. The individual interviews and these group discussions were analyzed and the results presented in a report.
To date, the following reports have been produced and are available:

- Evaluation of the individual interviews on SCRIPT from March 2022
- Evaluation of the group discussion on SCRIPT from July 2022
- Evaluation of the group discussion on SCRIPT from March 2023
- Evaluation of the group discussion on SCRIPT in June 2023 (present report)

The participatory support group for the SCRIPT study met for the third group discussion on June 5, 2023. Of the initial nine members of the participatory support groups, four cannabis users were present this time, who discussed the following topics under the moderation of Beatrice Metry (research assistant):

- The consent form for participants
- Stop smoking advice in pharmacies with a newly developed advice tool
- The case report forms
- The website www.script-studie.ch

In this evaluation report, the sample is outlined and the results are then presented according to the topics in the questionnaire, which can be found in the appendix. The appendix contains the questionnaire, the stop smoking tool and information on the case report forms.

2 Methodological approach

The various parts of the procedure are described below. All interviewees signed an informed consent before the individual interview (2021) and agreed to the interview and the subsequent group discussion and its evaluation by signing it. This assured them of anonymity.

2.1 Clarification of responsibilities with the Bern cantonal ethics committee

On May 31, 2021, the concept for the qualitative accompanying research entitled "Use of a participatory support group consisting of cannabis-using adults as a supplement during the planning phase of the SCRIPT 2 project" was submitted to the Cantonal Ethics Commission (KEK) to check its competence. The CEC found that it was not responsible. This means that this research does not fall under Article 2, paragraph 1 of the Human Rights Act and that individual and group interviews with the selected target group can be conducted without submitting an application. After this decision[1]of June 20, 2021, the acquisition of participants was started.

2.2 Data collection and evaluation procedure

The data was collected through a group discussion along a defined question route over a period of two hours, digitally recorded and then transcribed.

The transcript was analyzed using MAXQDA software. The first step involved coding the text, followed by condensing the individual statements and writing down the results.

2.3 Sample

All nine people who had already taken part in the individual interviews and signed the corresponding informed consent were requested for the group discussion. The appointment was arranged via doodle. Seven people registered for this group discussion and four people showed up for the group discussion. The following table provides an overview of the sample.

[1]BASEC no. Req-2021-00609, notification of the clarification of responsibility is available from 20.6.2021

	Individual interviews 4th quarter 2021	Group discussion 1 July 2022	Group discussion 2 March 2023	Group discussion June 2023
**Number of persons**	9	6	5	4
**Year of birth**	1957, 1975, 1990, 1991, 1993, 1999, 2000, 2001, 2000	1957, 1975, 1991, 1993, 2001, 2002	1957, 1991, 1993, 2000, 2002	1957, 1975, 2000, 2002
**Sector Occupation**	Studies (3)<br>Logistics (2)<br>Finance (1)<br>Photography (1)<br>Graphics (1)<br>Fitness (1)	Study (1)<br>Logistics (1)<br>Finance (1)<br>Photography (1)<br>Graphics (1)<br>Fitness (1)	Study (2)<br>Finance (1)<br>Graphics (1)<br>Fitness (1)	Study (2)<br>Graphics (1)<br>Photography (1)
**Salutation**	Woman 3<br>Man 5<br>non-binary 1	Woman 2<br>Man 3<br>non-binary 1	Woman 2<br>Man 3<br>non-binary 0	Woman 1<br>Man 2<br>Non-binary 1
**Nationality**	Switzerland 9	Switzerland 6	Switzerland 5	Switzerland 4

3 Results from the group discussion

The results are listed below along the question route. Numbers in round brackets indicate how many respondents answered in that direction. If there are no brackets after the statement, it is an individual answer. The paragraph in the MAXQDA file (A) in which the citation can be found is listed in square brackets.

3.1 Study information and informed consent form

The study information and the informed consent form were sent to the participants electronically before the session. They were instructed to read the document (study information and informed consent form) and to note any questions or discrepancies in the text and bring them to the meeting.

One person drew attention to spelling and grammatical errors and half sentences in writing before the meeting. These findings were passed on to the research team before the meeting.

3.1.1 Comprehensibility of the study information and the informed consent form

All persons present (4) stated that they had understood the study information and the informed consent form. What remained open to all was the number of documents. The participants received a document containing the study information, the declaration of consent for the SCRIPT study and the declaration of consent for the further use of data and urine samples for subsequent studies. These were three documents for the participants.

- One document from line 1-374: The study information.
- A document from line 375-446: The declaration of consent for participation in the SCRIPT study.
- A document lines 447-479: The declaration of consent for the further use of data and urine samples from this study in encrypted form.

In this context, the question arose as to whether the participants would be excluded from the SCRIPT study if they only gave informed consent for SCRIPT (4). One person said the following:

> *Maybe it is possible [...] to say, now regardless of what you decide here, that you can still continue in the study. And that these data can still be used independently of the current [SCRIPT] study, if you agree.* [A 112]

It was suggested that the following insertion be made after line 380 or 447 (3):

- Irrespective of whether I agree to the further use of my data or not, I will remain in the SCRIPT Study.

- I agree with the SCRIPT study Yes/No
- I agree to the further use of my data and urine samples for unspecified research projects for an indefinite period of time Yes/ No

One person expressed understanding for this request, but said it would be understandable for them even without this addition. Below is a quote from this voice:

> *I simply wouldn't sign if I didn't want to. I wouldn't need another clause telling me that I'm in, regardless of this second part [declaration of consent for the further use of data and urine samples, author's note]. That's pretty clear to me. But I see your point. So for your understanding, that's easier. It is anyway.* [A 146]

One person stated that the statement on lines 226-228 were confusing. She formulated this as follows:

> *Yes, I was confused about line 226. about possession of study cannabis in public spaces. It says study cannabis will not be confiscated if the original package is still unopened and you have no more than 10 grams on you. I didn't understand that. What does it mean if it's opened, then they can confiscate it? [*A 46][2]

Three participants said that they expected to receive a copy of the study information, the declaration of consent for the SCRIPT study and the declaration of consent for further use of the data. In this way, they would also see who the test person was and would have something in their hands. This is underlined by the following quote:

> *'So for me it is absolutely clear that I will receive a copy. [*A 173]

The fourth voice said on this subject:

> *So now that you say that, I think, yes why not. But it wouldn't be the first thing that would have occurred to me that I would have to demand that [a copy, author's note]. I think I have a rather naïve trust.* [A 177]

[2]Author's formulation idea: You may keep study cannabis at a police check if:

- the original package is still sealed/sealed.
- you are carrying a total of 10 grams or less of cannabis flowers or cannabis resin and a maximum of 2 grams total THC content for mixed cannabis products.
- you can show your valid student ID.

Lines 390-391 already state "*I will keep the written information and receive a copy of my written consent form.*" The participants were obviously not aware of this during the discussion.

3.1.2 Statements on the content of the study information and consent form

Line 173 contains the reference to the Bern City gift voucher. Two people stated that they did not know what this meant. It would therefore be practical if a link or QR code were included.
The offer of being able to have non-student cannabis tested was positively received by all those present (4). One person suggested that they would like to receive the results of the test, as the following quote shows:

> *What else I would think would be cool or recommendable if we could get those results too. Because it would be interesting to see what's in this stuff that you smoke yourself. Assuming it's from the black market or home-grown.* [A 187]

Another person countered with the following statement:

> *And funny, there I had the self-image that I thought: hell good, then you've just controlled it. So I wouldn't have expected anything other than that we would receive feedback on what conclusions they draw from the sample.* [A 199]

Whether the results of the tested cannabis are communicated by telephone or in writing is fine, according to those present.

3.1.3 Study participation

All four people stated that they would be willing to sign this consent form and participate in the study under these conditions. However, some points gave rise to discussion. These included lines 429 - 435, which begin with "Consent for the collection of contact details of a relative and/or the family doctor". At the time of the discussion (and reading), it was unclear to the participants why this data should be collected. Three people stated that the family doctor was not an option for them, as the following quotes from two voices show:

> *So, I would never check the GP box. But, you do have a choice. [...] [A 17]*

> *I was also confused by the GP. So I can understand you saying no. And otherwise, if we had talked about it, you might not have said no. Because my GP doesn't know that I'm gobsmacked. It's none of his business either. And I was a bit irritated when I read that. [A 204]*

I don't think anyone will name the family doctor. If you can't name anyone from your friends and family, then I don't think the family doctor is the right one either. I don't think he's ever the right one. I don't think many people would name their family doctor. And then there's the question: Is it wise to put him on there? [A 208]

I don't have a family doctor. [A 210]

It was unclear to all participants what was meant by the term "study data" (line 432). What exactly was to be collected remained vague and this should be defined precisely. This aspect also remains open on lines 140-142. The group discussed whether the aim was to get in touch with a person who no longer reports, or whether information on the person's state of health would be asked, or whether cannabis consumption habits and experiences with the purchase of cannabis in pharmacies would be asked.

During the discussion, the idea arose to speak only of the *contact person (4).* This would include all possibilities, including the family doctor. The following quotes from the discussion:

[...] And relatives is completely unproblematic for me. That is perhaps an unfavorable word. The question is, could it also be a friend or simply a contact person? Maybe not family, but someone else. [A 17]

Or simply a contact person, what XY [other participating person, author's note] said. That's a bit more general and doesn't exclude anyone. [A 27]

One person said:

I think you could theoretically leave it. Because it doesn't hurt anyone if it [the family doctor, author's note] is there. [A 212]

And another person supplemented the *contact person* with the following quote:

Or you can simply write "it can also be the family doctor". That way you don't exclude him. It is implied indirectly. [A 215]

The group also discussed why the contact details of the contact person were not asked for directly (3). The following quote underlines this view:

I think in general on line 436 there should be a direct indication option. With the comment as to why this person was chosen. I found that a bit strange.

> *Then I tick "yes" here and then afterwards there's the contact person I want. You can specify that right there. [A 42]*

In connection with these various consents and the consent for further use of the study data and urine samples, the question arose as to whether these answers would have an influence on admission to the SCRIPT study. As already mentioned in Chapter 3.1, the majority of the group (3) would like clarity about the study exclusion criteria. Paragraph 9 (lines 254-265) only lists criteria for exclusion from cannabis use.

3.2 Stop smoking advice in the pharmacy

All respondents stated that it is good that pharmacies offer advice on how to stop smoking. However, this should be addressed in a targeted, discreet and friendly manner and a *no* from the customer should be accepted immediately. Those present thought that one offer of stop-smoking advice with a follow-up purchase would be enough. One person said she would like to see a sign on the door of the pharmacy to make it clear that stop-smoking advice is offered here. That way, interested customers could ask for it themselves.
Below is the relevant quote:

> *Or, maybe it has a little sign on the door of the pharmacy that says: "Stop smoking advice here". And that people feel addressed. And not that they [the pharmacists, author's note] ask the person who goes to buy something in the pharmacy: May I give you some advice on how to stop smoking? That would be like those at the kiosk who always say: May I offer you another chocolate bar? Yes, they can do that. But I think it should be more the case that people ask for it themselves. That way they feel addressed. That's what I would say now. And also publicize the fact in the pharmacy that you can have it here (stop smoking advice). [A 251]*

Another person countered this statement as follows:

> *'But that can also have the opposite effect. If someone who smokes goes to the pharmacy and then sees this sign. Then they think: No, I'll go to another pharmacy. Because I know a lot of people who smoke and don't want to stop.*
> *And it quickly gets on their nerves when they force it on you. So I find it more discreet when you ask a side question. Like this: Yes, do you smoke? That only has to be a side question. Not that it becomes the main topic.* [A 252]

Of the four people present, two would not make use of stop-smoking advice because they declare themselves to be occasional smokers and have no nicotine addiction.

nicotine addiction. One person stated that they would accept stop-smoking advice because they were interested and would like to learn more. This person put it like this:

> Yes. I think I would certainly listen once. I smoke, but not a lot. Sometimes more, sometimes less. Today, for example, I haven't smoked yet. But I'm always interested in what options are out there. I've just bought an e-cigarette without nicotine. Simply out of interest. And that actually suited me very well. [...] But yes, I would certainly listen in. [A 271]

The fourth person said she would listen, but had little interest in quitting smoking at the moment.

A group of pharmacists at BIHAM is currently working on a decision aid for the choice of treatment method to support smoking cessation (Appendix 3, Chapter 6). This decision aid was presented to the support group with the question of how they would react if they were shown this decision aid in the pharmacy during the stop-smoking consultation.

The interviewees stated that the decision aid was clear (3), but that the graphic could be made simpler (3) without elaborating on this. Another respondent noted that all important and interesting information could be found on this overview. Only herbal alternatives such as damiana were missing.

One person said that personal advice was definitely needed for this overview.

When asked how they felt about the two products listed, e-cigarettes and nicotine pouches, they gave different answers. One person thought that e-cigarettes with nicotine for vaping were acceptable, but that offering nicotine products was incomprehensible. After all, e-cigarettes contain many toxins and are more harmful than tobacco cigarettes. This person made this clear by banning Juuls, as the following quote shows:

> *But there are otherwise toxic substances. So my first thought when I see something like that is Juuls. They were also e-cigarettes with liquids. They were the first e-cigarettes, which are now banned.* [A 316]

And three other people said that an e-cigarette would not be an option for them as a substitute for tobacco cigarettes.

One person stated that smoking is more than just nicotine consumption for cigarette users. It was smoking in and of itself, a ritual with a fixed routine. The same person said that they were shocked by the addictive nature of e-cigarettes, which is shown on this overview.

Another person said it was good that there were alternatives to tobacco smoking. This statement is supported by the following quote.

> *So I think the opinion now is that nicotine is extremely harmful. Which is true in large quantities. It can also be fatal. But I believe that smoking itself causes more diseases. Like COPD[3]or something like that.* [A 353]

And another person replied:

> *It's good, there are alternatives. [*A 360]

The nicotine pouches were not approved by those present (4) for personal use, but as a nicotine replacement product for people with health problems (2).

3.3 Case report forms

One person pointed out right at the beginning that people usually overestimate their memory and that a questionnaire containing questions about events that happened 6 months ago would hardly elicit realistic answers. This person therefore suggested that the questions for the consultation should be asked much earlier.

> *1-2 weeks after the consultation or after a week. Then I can say how I found it. Especially if you want to go into more detail. But half a year later, I still know that it took place.* [A 459]

It was suggested that questions about the price-performance ratio (4), opening hours and the extended range (e-vaporizers, e-cigarettes, paper, filters, etc.) should be included. One person suggested asking customers about their needs in a different way. The following quote is inserted:

> *Is it about being able to tailor the supply even more to needs? I wouldn't ask about each individual, but rather ask myself: Have you obtained your cannabis exclusively from the pharmacy in the last 6 months? Or pharmacy and black market? And if you haven't bought exclusively from the pharmacy, what are your reasons. Then I have exactly what I want. I think, if that's the only intention. Because long questionnaire where I've already forgotten each (products) even exist. And so afterwards I have your answer very precisely. If that's the only thing you want to know.* [A 436]

Another voice countered as follows:

> *I think it's important to ask about all products. That way you can also evaluate, do you need more? Do we need less? Do we need other things? I think*

[3])Chronic obstructive pulmonary disease

> *This is important for the offer. But it is certainly an interesting question to add. To ask, did you only buy it in the pharmacy or not, or both?* [A 437]

The question about the warnings[4]and the type of warnings was the subject of lively discussion. Those present were not previously aware that there would be a warning on the packaging. One person answered the question of whether they would buy cannabis with a warning label as follows:

> *'It depends a bit on what it looks like. So if it's as horrible as the EU cigarette, then I wouldn't buy it. No, I wouldn't do that. It's just dramatic. You don't even see the name of the cigarette anymore. [...] Horrible pictures. And that's mean. It's a cheek.* [A 401 + 402]

Another person underlines this statement with the following quote:

> *Yes, you know, such a creepy military green. And afterwards it has such big (pictures) of open hearts and smokers' lungs and stuff. In the EU. Or you don't have a display of all the different brands. It's just one unit. You have to know exactly what you want to buy*. [A 403]

These two people stated that they only wanted a language warning on the packaging. And if there is a picture, then only one of the happy hemp farmer.

The other two people stated that they did not react to the warnings. They didn't care what was written there, they were interested in the content.

Two people described the questions about the advice as open and general. One of these people suggested asking more in-depth questions.

> *So I think that compared to the questionnaire above [about the products], this one is mega open. You can actually ask: Was the consultation informative? So mega simple questions. And if not, what was missing? I think this question[5]is very general and so open.* [A 440]

One person responded as follows:

> *Well, I think it's good the way it is. I think if there are more questions, then I'm grateful if they are yes/no questions. Because at this point you've been answering them for a long time and I wouldn't feel like writing in detail about the consultation. So yes/no questions would be the most practical for me. For example*

---

[4]The warnings on the packaging of the study cannabis are understandable.
[5]I was satisfied with the consultation.

I think it's extremely good that you can simply tick the topics and don't have to write them down yourself. [...] And even after six months, I could still say which topics I received information on. They don't ask what information in the sense of extensive details. They only ask: Has this topic been addressed? Yes, no. I could certainly say that, even after six months. [A 464]

In response to the questions about sources of information on low-risk cannabis use, two respondents said that "BeGes[6]" was not comprehensible and should be written out.

3.4 Website script-study.ch

The respondents had a few compliments to say about the website. All found that the website fulfilled its purpose (4). The website was described as clear (2) and easy to navigate (2), beautiful and leaves a good impression. The information content (2) and the implementation of usability (2) were also mentioned positively. The news (4) and the study information (4) were perceived as clear. The animation of the image was mentioned as particularly cool by two voices.

Those present had various ideas and suggestions as to what could be changed on the website. For example, it was noted that clicking directly on Log In and not using the drop-down menu takes you to the Wordpress Log In. All those present agreed that this should definitely be fixed.

The FAQs could be supplemented with the following questions and answers: How much THC does an average joint contain? (As a reference value. Very few people can do anything with 10 grams of THC per month) (3) Who can take part in the study? (3) Who will be excluded from the study? (3). One field: Do you have another question? And link directly to the e-mail address or contact form (2).

If you move the mouse over "About us", you will see the drop-down menu containing the contact form. Clicking on "About us" only takes you to the description of the SCRIPT study. There is no contact form, which was seen as a shortcoming (3). One person notes the following:

You must not look for it. You have to find it right away. [A 584]

One idea was to place "Contact" as a separate tab in the top bar and to list "About us" as a subpage under Pilot study. Under "About us", three people would like to see pictures of people who are carrying out this study. The following quotes illustrate this:

[6] Berner Gesundheit

Under "About us" it would be nice to see a few people who are taking part. [A 623]

Well, I agree with you on that. It's somehow always the case that there's always a photo of someone under "About us". [A 626]

One person suggested putting more pictures on the website in general. Another person disagreed as follows:

[...] I have to disagree with you. I wouldn't take more pictures. Because what kind of pictures are they? [...] It has to be sober. You don't want to show a filthy room. You tell a story about smoking weed as soon as you take a picture. [A 620]

A lengthy discussion ensued as to whether "News" should be featured so prominently and with its own knight. The same information appears on Home. One person referred to the people who would register, who would not be interested in news, this person was convinced. Two other people replied that there were other people interested in the SCRIPT study. For them, the news on the status of the study is very important.
The following quote underlines this statement:

But it's not just for signing up. It's clear that you can register on the website. But I still think it's a point of reference, firstly to contact people and secondly to inform them. So, when I go to the website, I want to be kept informed about what's going on. We are like participants in the study. We want to get our stuff now and all that. But, if there's a way to keep me informed on the website, so: we're at that step now. Somehow in the other cantons there are now also attempts at similar studies. Etc. I think that's really important. [A 629]

Another aspect was the imprint of the website. One person commented on this as follows:

Yes, the imprint is a bit slim. The imprint also has e-mail or other contact details. Exactly, there is a contact there. I just noticed that the legal notice is relatively slim. Just these 3 unis. And the copyright. But that's ok. [A 640]

3.5 Information and perspective for the support group

Those present were informed that the research group is working on launching a pilot study for SCRIPT implementation. During the pilot study, the members of the support group will act as a pilot group and examine the processes of cannabis acquisition in pharmacies and bring their experiences back to the support group. All those present are looking forward to participating in a pilot group.

The group was also informed that the research group is looking for a way to include the participants of the support group in the group, which will be able to obtain cannabis products from pharmacies from the start of the study.

4 Conclusion

The participants in this support group meeting were generally positive about the consent form, the stop smoking advice tool, the case report forms and the website www.script-studie.ch.

All four would sign the ***consent form*** immediately. The main point for discussion was the indication of a relative or family doctor. The group said that specifying a *contact person*, who could also be the family doctor, would be more appropriate. The details of the contact person could be collected from this consent form.

The ***stop smoking advice*** was met with moderate enthusiasm by the four people surveyed. Two people do not smoke tobacco. One person feels comfortable with their cigarette consumption and another person would accept stop-smoking advice out of interest in learning something new. The stop smoking advice tool was rated as clear and informative.

The ***case report forms***, which the participants will complete after 6 months, were supplemented by the respondents with further questions on the price-performance ratio of the products. The warning label that will appear on the products was discussed intensively. The participants compared the warning label with that of EU cigarettes.

Respondents liked the SCRIPT study ***website***. In addition to the positive aspects, they gave specific indications of shortcomings in the website structure. For example, clicking on "about us" opens the Wordpress login, which the group felt should not be the case.

The support group was informed that the research group is working on launching a pilot study before the implementation phase and that if this comes to fruition, the support group members will be involved as a pilot group.

The next monitoring group meeting is scheduled for September 2023.

.

5 Appendix 1: Questionnaire for group discussion

Preparation of SCRIPT support group for cannabis users

June 05, 2023 / 18:30 - 20:30

Welcome, agenda, duration (5`)

Study information and declaration of consent (45`)

The participants received an email on 19.5.2023. This contained the document "Study information and declaration of consent".

The preparation assignment for the group was as follows:

You will find a pdf attached. There are line numbers in the margin so that we can orient ourselves well in the exchange. These will be deleted in the original. Please read the document carefully and keep the following questions in mind.

1) Do you understand the information and the declaration of consent? Are there any stumbling blocks or blurred, misleading formulations? If so, which ones?
2) Do you still have questions after reading through the document? If so, which ones? Please keep the following in mind: Rights, duties and procedure. Please bring your questions to the meeting on June 5, 2013.
3) Now that you have read the information and consent form, do you agree to take part in the study? If yes, what convinced you? If not, what are your thoughts on this?
4) The declaration of consent begins on page 10. This contains different parts of the study.
5) Participation in the study in general
    - Participation in interview during the study
    - Bringing cannabis samples for analysis (home-grown or black market)
    - Recording of contact details (family doctor or relatives) if you are no longer able to do so yourself
    - Further use of data and urine samples These are additional urine samples that are brought to study visits during the study. (A urine sample at the 1st study visit is mandatory for everyone. See paragraph 4 Procedure of the study visit).
        - To which of these study parts do you say yes? Subject information

Procedure at the session:

Take up and discuss questions 1-5 from the preparation. Further

questions: Stigmatizing sentences? What is missing? Superfluous?

Stop smoking advice (30`) ppt

Cannabis participants may take advantage of stop smoking advice. The pharmacists have Instruments to support a decision on alternative products. I have the following questions:

- How do you feel about stop smoking advice in pharmacies?
  - Would you accept the offer? Under what circumstances?
- How does this overview of nicotine products and alternative offers affect you?
  - How would you react if a pharmacist presented you with such documents?
  - Is this overview helpful to you? Why?
  - Would you appreciate this selection?
  - Do you have any reservations? What reservations? Why?
- What do you think about the e-vaporizer and nicotine pouch offers?

Case Report Forms (20`)

These are forms that are filled out by the participants during the study visits. They also deal with satisfaction with the reference centers. These questions are used to improve the services and advice provided during the study.

- What questions would you ask to obtain information on satisfaction with the reference centers?
- What questions would you ask to obtain information on satisfaction with the advice provided?
- As a consumer, what is most important to you about the advice/referral centers?
- What would you explicitly ask to improve satisfaction with the offer and advice during the study?

Website www.script-studie.ch (10`)→ Switch pages to screen

- What did you notice?
- What did you like? What less?

Information and outlook (5`)

Participants in the PBG can take part in the study.

An attempt will be made: Introduce the TN PBG as a ***pilot group*** from October 2023 to test the processes.

If pilot is rejected:

- All TN come into study 50% in control group, 50% in intervention group
- The aim is for all PBG participants to join the intervention group

Next meeting in Sept. 2023

6 Appendix 3: Decision aid for the treatment method to support smoking cessation

Wahl einer Behandlungsmethode zur Unterstützung des Raucherentwöhnung

	NIKOTINERSATZPRODUKTE						KOMBINATION
**Produkt**	Nicorette® Sublingual Tablette<br>Liste D	Nicorette® / Nicotinell® Lutschtablette<br>Liste D	Nicorette® Kaudepot Nicotinell® Kaugummi<br>Liste D	Nicorette® Inhaler<br>Liste D	Nicorette® Spray zur Anwendung in der Mundhöhle<br>Liste D	Nicorette® Depotpflaster Nicotinell® Pflaster<br>Liste D	Pflaster & Nikotinersatztherapie mehrere Kombimöglichkeiten<br>Liste D
**Darreichungsform**	Sublingual-tabletten	Lutschtabletten	Kaugummi	Inhaler	Mundspray	Pflaster	Pflaster & anderes NEP
**Verfügbare Dosis**	2 mg	1 mg / 2 mg	2 mg / 4 mg	10 mg	1 mg / Sprühstoss	21, 14 und 7 mg / 25, 15 und 10 mg	siehe Pflaster & andere NEP
**Anwendung**	8-12x / Tag nach Bedarf	8-12x / Tag nach Bedarf	8-12x / Tag nach Bedarf	8-12x / Tag nach Bedarf	12-25x / Tag nach Bedarf	1x / Tag während 16 oder 24h oder nach Bedarf	Pflaster 1x / Tag + anderes Ersatzprodukt nach Bedarf
**Vorteil für Nutzer:innen**	• Unauffällig mit dem Rauchen aufhören	• Unauffällig mit dem Rauchen aufhören	• Erhältlich in verschiedenen Wirkstoffstärken und Geschmacks-richtungen<br>• aktiv aufhören	• «Hand-zu-Mund»-Bewegung wird simuliert	• Erhältlich in verschiedenen Geschmacks-richtungen<br>• Rasche Hilfe nach 30 s	• Erhältlich in verschiedenen Wirkstoffstärken<br>• 1x am Tag<br>• Nikotinabgabe rund um die Uhr<br>• Nicotinell®: 24h Pflaster	• Bei starkem oder unkontrollierbarem Rauchverlangen<br>• Wenn bei Monotherapie rückfällig
**Preis pro Schachtel**	~ 65.– CHF<br>100 Tabletten à 2 mg	~ 25.– CHF<br>36 Tabletten à 2 mg	~ 20.– CHF<br>30 Kaugummis à 2 mg	~ 27.– CHF<br>18 Patronen à 10 mg	~ 60.– CHF<br>150 Sprühstösse	~ 120.– CHF<br>14 Pflaster à 15 mg	~ 120.– CHF<br>14 Pflaster und 1 Schachtel schnell-wirksames Ersatzprodukt
**Preis pro Tag (im Vergleich zu einer Zigarettenschachtel*)**	~ 6.– CHF / Tag	~ 6.– CHF / Tag	~ 5.– CHF / Tag	~ 9.– CHF / Tag	~ 6.– CHF / Tag	~ 7.– CHF / Tag	~ 11.– CHF / Tag
**Wirksamkeit**	++	++	++	++	+++	++	+++
**Abhängigkeit**	+	+	+	+	+	+	+
**Hauptnebenwirkungen**	• Irritationen im Mund- und Rachenbereich<br>• Schluckauf<br>• Übelkeit	• Irritationen im Mund- und Rachenbereich<br>• Schluckauf<br>• Übelkeit	• Irritationen im Mund- und Rachenbereich<br>• Schluckauf<br>• Übelkeit	• Irritationen im Mund- und Rachenbereich<br>• Schluckauf<br>• Übelkeit	• Irritationen im Mund- und Rachenbereich<br>• Schluckauf<br>• Übelkeit	• Hautirritationen	• Hautirritationen, Irritationen im Mund- und Rachenbereich<br>• Schluckauf<br>• Übelkeit

Wahl einer Behandlungsmethode zur Unterstützung des Raucherentwöhnung

	OHNE MEDIKAMENTE			MEDIKAMENTÖSE THERAPIE		
**Produkt**	Kognitive Verhaltenstherapie (KVT)<br>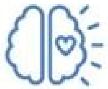	Motivational Interviewing<br>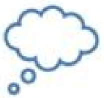	Raucherentwöhnung ohne Unterstützung (Reduzierung der gerauchten Zigaretten)<br>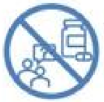	Vareniclin<br>Champix®<br>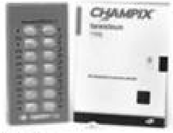<br>Liste B+	Bupropion®<br>Zyban®<br>*nur auf ärztliche Verschreibung*<br>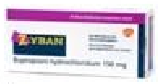<br>Liste A	Cytisin®<br>Tabex®, Desmoxan®, Cravv®, Asmoken®<br>*nur auf ärztliche Verschreibung*<br>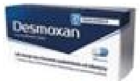<br>**In der CH nicht verfügbar**
**Darreichungsform**	**Psychotherapie**	**Psychotherapie**	**Keine**	**Tablettten**	**Tabletten**	**Keine**
**Verfügbare Dosis**	-	-	-	0.5 mg / 1 mg	150 mg	1.5 mg
**Anwendung**	-	-	-	2x / Tag	2x / Tag	6x / Tag, dann 2x / Tag
**Vorteil für Nutzer:innen**	• Keine medikamentöse Therapie<br>• Strategien lernen zur Bewältigung des Rauchdrangs<br>• Vertrauen in der eigenen Abstinenzfähigkeit stärken	• Keine medikamentöse Therapie<br>• Verhaltens-änderung durch Argumente auskommend von Patient:in, nicht von therapierender Person	• Keine medikamentöse Therapie<br>• Keine Sprechstunden nötig<br>• Verschiedene Methoden: Schlusspunkt- oder Reduktionsmethode	• Unauffällig mit dem Rauchen aufhören<br>• Kann unter bestimmten Bedingungen von der Grundversicherung übernommen werden	• Unauffällig mit dem Rauchen aufhören<br>• Kann unter bestimmten Bedingungen von der Grundversicherun g übernommen werden	• Unauffällig mit dem Rauchen aufhören<br>• Kann zur Tabakentwöhnung angewendet werden, wenn andere Therapieformen nicht zum Erfolg geführt haben
**Preis pro Schachtel**	individuell<br>je nach Tarif / Anzahl Sitzungen	individuell<br>je nach Tarif / Anzahl Sitzungen	Keine Kosten	~120.– CHF<br>56 Tabletten à 1 mg	~60.– CHF<br>30 Tabletten à 150 mg	~90.– CHF<br>100 Tabletten à 1.5 mg
**Preis pro Tag (im Vergleich zu einer Zigarettenschachtel')**	**individuell**<br>je nach Tarif / Anzahl Sitzungen	**individuell**<br>je nach Tarif / Anzahl Sitzungen	**Keine Kosten**	~ 4.– CHF / Tag	~4.– CHF / Tag	~3.60.– CHF / Tag
**Wirksamkeit**	++	+	(+)	+++	++	++
**Abhängigkeit**	-	-	-	-	-	-
**Hauptnebenwirkungen**	• Keine	• Keine	• Reizbarkeit, Wut, Nervosität<br>• Müdigkeit<br>• Ängstlichkeit<br>• Schlechte Laune<br>• Craving	• Übelkeit<br>• Schlafstörungen<br>• Stimmungs-schwankungen<br>• Albträume	• Schlafstörungen<br>• Trockener Mund<br>• Kopfschmerzen<br>• Stimmungs-schwankungen<br>• Verdauungsstörungen	• Kopfschmerzen<br>• Übelkeit<br>• Schlafstörungen<br>• Magen-Darm-Beschwerden

	NEUARTIGE NIKOTINPRODUKTE (NICHT IN DER APOTHEKE ERHÄLTLICH)	
**Produkt**	Nikotinhaltiger Verdampfer<br>mehrere Marken<br>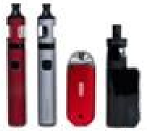<br>In Fachgeschäften verfügbar	Nikotinhaltige Beutel<br>mehrere Marken<br>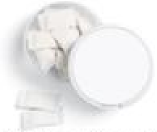<br>In Fachgeschäften verfügbar
**Darreichungsform**	**E-Zigarette**	**Nikotinbeutel**
**Verfügbare Dosis**	verschiedene Dosierungen	verschiedene Dosierungen
**Anwendung**	Wenn Medikamente nicht genug wirksam sind, nach Bedarf	Wenn Medikamente nicht genug wirksam sind, nach Bedarf
**Vorteil für Nutzer:innen**	• «Hand-zu-Mund»-Bewegung wird simuliert	• Tabakfreies Produkt<br>• Erhältlich in verschiedenen Geschmacks-richtungen
**Preis pro Schachtel**	~50.– CHF<br>Starterkit – diverse Marken	~7.– CHF<br>1 Dose à 21 Beuteln
**Preis pro Tag (im Vergleich zu einer Zigarettenschachtel')**	~2 bis 3.– CHF / Tag	~2 bis 3.– CHF / Tag
**Wirksamkeit**	+++(+)	++
**Abhängigkeit**	+++	++
**Hauptnebenwirkungen**	• Irritationen im Mund- und Rachenbereich<br>• Mögliche Exposition gegenüber giftigen Verbindungen<br>• Husten	• Schluckauf<br>• Übelkeit<br>• Suchtgefahr

7 Appendix 2: Study information and informed consent form

Insertion of information on participation in a pilot study: The SCRIPT TRIAL

Information on participation in a pilot study:

THE SCRIPT TRIAL

A randomized controlled pilot study on the regulated cannabis sales in pharmacies

Official title: The Safer Cannabis - Research In Pharmacies randomized controlled trial

---

Good day

Thank you for your interest in participating in this study.

The study is organized by a team of researchers working at the Bern Institute of Family Medicine Medicine (BIHAM) at the University of Bern. It is subject to strict regulations and Data protection regulations. Participation in the study is voluntary and can be withdrawn at any time without giving reasons. reasons.

This written information contains a brief summary of the key points of the study. key points of the study. This is followed by a detailed information letter. In addition we will inform you verbally about the project and answer your questions.

If you decide to take part in this study, we will ask you to complete the written declaration of consent at the end of this document and to confirm it with your signature. confirm that you agree with the requirements for participation in the study.

Summary

Why are we conducting this study?

Cannabis use is widespread in Switzerland and regulation has been under discussion for years. With this study, we want to investigate the effects on users if they are able to obtain cannabis products in a controlled setting. In addition, the participants receive information about the advantages and disadvantages of different forms of consumption, The focus is on providing information about alternatives to smoking. The findings from from this study should provide a knowledge base for the possible implementation of regulation. implementation.

What happens if I take part in a study?

The participants are randomly divided into two groups. One group is allowed to use cannabis cannabis products from the start; the other group may only do so after 6 months. For For all participants, the study lasts at least 1 year and a maximum of 2 years. At the beginning of the study and after 6 months a study visit takes place on site, the remaining appointments are carried out conducted by telephone or e-mail. The focus of the data collection is on various questionnaires on the subject of health and substance use.

How does the regulated sale of cannabis work?

You will receive a study card and can use it to purchase cannabis products in a selected pharmacy. obtain cannabis products. The supply is limited and includes cannabis flowers, cannabis resin (hashish), e-liquids and cannabis oils. Dispensing and information about Advantages and disadvantages take place in the pharmacy.

Do I benefit from participating in the study or am I exposing myself to a risk?

There are no additional risks for you by participating in the study and no direct personal benefit is expected. personal benefit is expected. All cannabis products offered in this study are subject to strict safety are subject to strict safety regulations and have been tested in the laboratory. Likewise you will have a contact person at the pharmacy to answer any questions you may have about health, safety and addiction in the case of substance use.

Detailed study information

1. Aim of the study

The main objective of this pilot study is to assess the effect of regulated cannabis dispensing, combined with information on the subject of cannabis and substance use, on users. users. The focus is on individual health and the topic of smoking and alternative forms of consumption. alternative forms of consumption. The findings from this study should provide a knowledge base for the possible implementation of regulation. 69

2 Selection of study participants

The study is open to people who

- already consume cannabis regularly,
- are at least 18 years old and
- are resident in the Canton of Berne
- are not already participating in another study with regulated cannabis sales or have participated

3. general information

Cannabis is the most commonly consumed illegal substance in Switzerland. However, illegal consumption negative aspects such as uncertain product availability and quality as well as limited limited contact points for information and, if necessary, help. With the amendment of the Narcotics Act, which came into force in May 2021, it is now possible to scientifically investigate the regulated cannabis distribution scientifically and to analyze whether this could improve the situation of users could improve as a result. 85

In this study, participants can obtain various cannabis products from a selected pharmacy pharmacy: Cannabis flowers, cannabis resin (hashish), e-liquids and cannabis oils. At the beginning of the study, they will receive a separate list with a detailed description of the products and their ingredients. All products are manufactured in Switzerland, are subject to strict quality tests and are produced in accordance with the provisions of the Organic Farming Ordinance. The prices take into account the active ingredient content and the prices on the black market. 92

At the same time, the pharmacy serves as a point of contact for information on health and cannabis and other substance use such as tobacco or alcohol. The focus is on advice on the use of cannabis, in particular on smoking as an important risk factor for many risk factor for many diseases, and alternative forms of consumption such as vaporizing vaporizing, vaporizing or eating cannabis products 98

In order to clearly determine the effects of regulated cannabis use, it is important to randomly important to randomly divide the participants into one of two groups at the beginning of the study. The group (known as the "eligible group") can obtain cannabis products from the outset. from the beginning. For the second group (known as the "waiting list group"), this is only permitted six months after the start of the study (see Fig. 1). The study lasts at least 1 year for the participants and a maximum of 2 years. This depends on the individual start of participation. Overall, we would like to at least 1,000 people for this study.

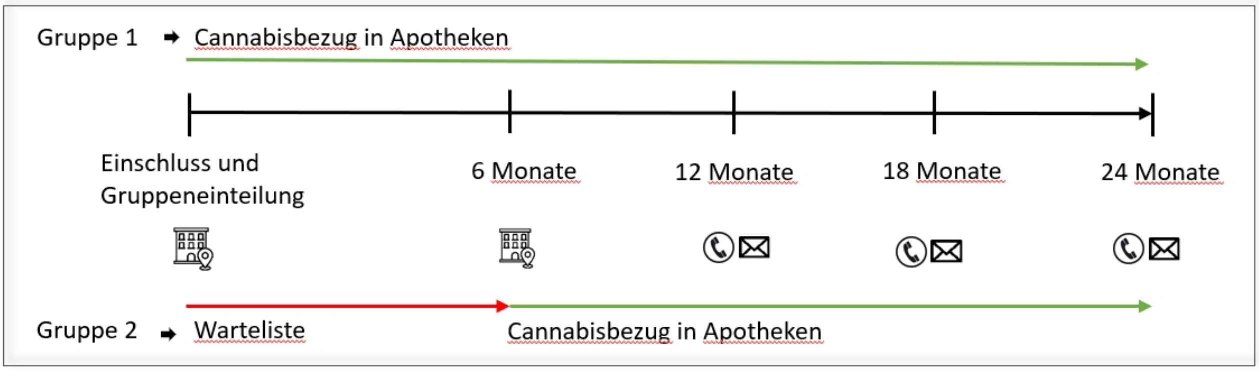

Fig. 1: Study design; = On-site study visit; = Study visit by telephone / email

The number of cannabis withdrawals in pharmacies is not limited. However, the law stipulates law stipulates that no more than a total of 10 grams of cannabis per person per purchase for unmixed products and no more than 2 grams of THC for mixed products. may be used. Furthermore, distribution is limited by law to a total of 10 grams of THC per month. Further legal provisions apply to the possession and consumption of study cannabis (see chapter 8).

We conduct this study in accordance with the laws in Switzerland and comply with all internationally recognized guidelines. all internationally recognized guidelines. The responsible ethics committee and the Federal Office of Public Health (FOPH) have reviewed and approved the study. A description of this study can also be found on the FOPH website at www.kofam.ch.

4 Procedure for the study visits

You will be asked to come to the study center in person for two study visits. At first visit before participation begins, we will discuss the procedure and sign the consent form. declaration of consent. In addition, we will use a urine sample to check whether you already consume cannabis. already consume cannabis. Please note that for this reason the last cannabis use must not be longer than should be more than one week old. As the ordinance on pilot trials excludes the participation of pregnant women, the urine sample for women also excludes pregnancy. pregnancy is also ruled out. We then carry out a brief physical examination, which includes includes the following measurements: Height, weight, abdominal circumference, blood pressure and expired air. We need the exhaled air to determine carbon monoxide, which gives an indication of how much smoke you have been exposed to. We will also ask you to complete a questionnaire on the topics of substance use, especially cannabis, and health. Finally, using a computer and with a probability of 1:1, we randomly assign you to one of the two groups. into one of the two groups. If you are placed in the "eligible group", you will receive information on how to obtain cannabis from a selected pharmacy and a study card. study card.

At the second visit after 6 months, we will again carry out the physical examination and ask you again to answer a questionnaire. If you were in the "waiting list group", you will now also receive the information and the study card for the purchase of cannabis products in a pharmacy. If you are not available for the visit after 6 months months, we would like to ask you to allow a relative or your family doctor to contact you to record your study data. to contact you in order to record your study data. In the consent form at the

the end of this document, you can indicate whether you agree to provide this contact data. contact details.

The two on-site visits each last approx. 60 minutes.

After 12 months and, depending on the length of participation, also after 18 and 24 months, we will only ask you to ask you to answer the questionnaire by telephone or e-mail, which will take approx. 30 minutes. this will take about 30 minutes.

<u>Urine sample for pollutant determination</u>

We will ask a few randomly selected participants to provide a urine sample at the first study visit and the visit after 6 months. The material and instructions are made available. We will need these samples to determine various harmful substances that arise from smoking cannabis or tobacco in the laboratory.

<u>Interviews about cannabis and cannabis use</u>

In the consent form at the end of this document, you can indicate whether you would be willing to to participate in an additional interview (possibly divided into up to three appointments). It is important for us to find out what you think about the regulated cannabis distribution and how they assess the products on offer. An interview appointment can be made by telephone or on site and lasts approx. 60 minutes. You will receive compensation for your participation (see chapter 14).

<u>Cannabis samples for laboratory tests</u>

In the consent form at the end of this document, you can also indicate whether you would be willing to bring a cannabis sample to the study visit after 6 months that you have on the black market or which you have grown yourself and which we can test in the laboratory for its ingredients. The aim of the laboratory test is to analyze the content of THC as well as various harmful substances (pesticides and synthetic cannabinoids) and to compare it with the controlled cannabis from the pharmacy. For a random sample we need 1 gram of cannabis, which we will reimburse with a BERNcity gift card worth CHF 10. If you are interested, we will be happy to provide you with the results of the study. available to you.

5. procedure for obtaining cannabis

You can obtain the study cannabis from a selected pharmacy. If you wish, you can use the pharmacy's consultation room for this purpose. We ask you to register for your first visit by telephone and bring an official ID with you.

At the pharmacy, you will first be asked to answer a few questions about substance use and health. health. It will take about 10 minutes to complete this questionnaire. Based on Based on the information you provide, the pharmacist can give you information about your health and substance use. cannabis use, but also on tobacco and alcohol consumption. At the same time you will be presented with the various cannabis products and, if necessary, you will be informed about the the advantages of alternative consumption methods such as vaporizing or vaporizing cannabis products. vaporizing cannabis products. You can ask questions at any time.

You can then select the product(s) you would like to purchase and pay directly on site in cash or by card. Please note that you will need the student ID card for the purchase of cannabis. However, we recommend that you carry this with you

so that you can justify your possession of study cannabis in the event of a police check. possession of study cannabis.

6. Benefits

No direct personal benefit is expected from participating in this study. . One advantage of regulated cannabis distribution in this study compared to procurement on the black market is the black market is the quality assurance of the products. In addition, the stress caused by the illegal procurement should be avoided. In the pharmacy you can also health, safety and dependency of cannabis and other substances such as alcohol. such as alcohol. If you mainly smoke cannabis, you can try alternative, less harmful forms of consumption. Also Support is also offered for quitting smoking. All these offers are on a voluntary basis. In addition to the personal benefits, the social benefits should also be mentioned in this study. So The findings from this study are intended to provide a knowledge base for the possible implementation of cannabis implementation of cannabis regulation.

7 Voluntariness and obligations

Participation in this study is voluntary. If you do not wish to participate or wish to withdraw your participation later, you do not have to justify this. If you participate, we would ask you to be available for the study visits and to keep the appointments. and keep the appointments. Please note that the authorization for the use of cannabis from participation in the study visits (see Chapter 9). We would also like to ask you to you answer the questions about your cannabis use and your health truthfully. This is the only way we can draw important conclusions from the study. Finally, we would like to ask you to adhere to the safety regulations described below regarding safety regulations regarding the possession of study cannabis described below, which are based on the currently applicable regulations in Switzerland.

8. Safety regulations

Together with the police and other municipal authorities, we have safety regulations, which govern the implementation of the study with regard to the public space. public space:

<u>Possession of study cannabis in public spaces</u>

Study cannabis will <u>not be</u> confiscated by the police if:

- the original package is still unopened
- it contains no more than 10 grams of cannabis flowers or cannabis resin and no more than 2 grams of total THC content for mixed cannabis products
- be able to show your valid student ID.

If you do not present a student ID, your originally packaged study cannabis may be may be confiscated until you can be identified as authorized to obtain it. Please note that an opened package may also be confiscated. The study management recommends that you leave the cannabis packaging at your place of residence after purchase and report the possession of study cannabis in the usual street drug dealing areas, in public nightlife nightlife or at other public events.

<u>Distribution of study cannabis</u>

The transfer or sale of study cannabis to third parties and minors is punishable by law. In addition to a report to the public prosecutor's office, the cantonal police also report to the report to the study management. If it can be proven that study cannabis has been passed on, the will issue the person with a warning and, in the event of a repeat offense, exclude them from from obtaining study cannabis.

<u>Consumption in public places</u>

The usual provisions of criminal law apply to the consumption of study cannabis in public places. provisions apply. Consumption in public spaces may be punished with a fine. Private premises and private, non-communal areas are considered non-public spaces. used outdoor areas where the consumption of study cannabis is permitted.

<u>Road traffic</u>

In road traffic, the usual criminal law provisions apply, regardless of whether you participate in the study or not.

9. exclusion from obtaining cannabis

We may have to withdraw your right to obtain study cannabis. This may be the case if

- a) you become pregnant
- b) you pass on or sell study cannabis
- c) you develop a medical condition where the study doctor confirms that the cannabis use is not advisable (e.g. acute severe psychosis);
- d) you do not attend a planned study visit within three months despite several requests. study visit within three months.
- e) you move during the study to a canton in which the study is not being conducted.

If you are excluded from receiving cannabis for reasons a), b) or c), we will still ask you to attend the study visits. ask you to participate in the study visits anyway.

10 Risks and burdens

There are no additional risks or burdens for you as a result of participating in the study, apart from the time and effort required for the study visits. As you can see in chapter 12 we will handle your personal data confidentially. There is no exchange of personal data with the police or other (official) institutions, except to identify you identify you as a person participating in the study in the event of a check in a public space. you should carry study cannabis, but not a student ID card.

11 Results

There are individual results from this study that affect you directly. In the course of the study you will be You will be informed of any new results and findings that are important to you personally. There may also be individual results that arise by chance (so-called incidental findings), for example in a urinalysis. In the case of incidental findings, you will be informed if these findings are relevant to your health.

In order to obtain meaningful results from the study, we do not evaluate your individual results. results, but summarize the results of the participation groups and form so-called so-called objective final results. We will be happy to send you a summary of these final of these final results at the end of the study.

12. confidentiality of data and samples

12.1. Data processing of encryption

For this study, your personal and health data will be recorded in an online database and processed, partly in automated form. During data collection, your data will be encrypted. Encryption means that all reference data that could be used to identify you (name, date of birth, etc.) are replaced by a code. Your personal contact details are stored in a separate database, which is securely separated from the database is securely separated from the database containing your study data by means of technical precautions. This means that non-authorized persons cannot draw any conclusions from your coded study data about your person can be drawn. Only very few people have access to your study data, authorized specialists. This includes the study personnel at the pharmacy where you you obtain cannabis, or the study personnel who carry out the study visits, and this only to perform tasks related to the study. Your contact details may also only be study personnel in order to fulfill tasks related to the study, for example, to verify your identity when obtaining cannabis or to register you for a to call the study visit. All persons with access to your data are subject to a duty of confidentiality. As a participant, you have the right to access your study data.

12.2. Data protection and protection of samples

All data protection regulations are strictly adhered to. The online database was opened specifically for the study in order to store your research data securely and in encrypted form. It is possible that the study data must be transferred from the database, for example for a publication. The data remains encrypted and it is not possible to identify you personally. are not possible. The urine samples, if you have provided any, are also encrypted and sent to the Unisanté laboratory in Lausanne for analysis. laboratory at Unisanté in Lausanne for analysis. If you have consented to the further use of the urine samples, the samples will be stored in a biobank at the Unisanté laboratory. stored in a biobank. Otherwise, the samples will be destroyed after analysis. The cannabis sample, if you have provided one, will also be coded and sent to to the Institute of Forensic Medicine at the University of Bern for analysis. This sample is destroyed after destroyed after analysis.

12.3. Data protection for further use

Your data and, if applicable, urine samples, if you have provided such samples, could be important for be important for answering further questions in cannabis research. For this further use, we ask you to sign a further declaration of consent at the end of this document. declaration of consent at the end of this document. This second consent is independent of your participation in this study.

12.4. Rights of inspection during controls

This study can be reviewed by the approval authorities (ethics committee) or by a study-internal monitor. internal monitor. The study personnel must then disclose your data for such controls. All persons involved in these controls must maintain absolute confidentiality.

13 Withdrawal

You can withdraw from the study at any time. In the event of withdrawal, your data collected up to that point collected up to that point and any samples will still be included in the evaluation of the of the study. The data and any samples will not be anonymized. This serves primarily to ensure data quality. Please check whether you agree to this, before you take part in the study.

14 Compensation

If you take part in this study, we will reimburse you for your expenses for the two study visits on site with CHF 30 each and the telephone or online visits with CHF 20 each in the form of a BERNcity gift card. in the form of a BERNcity gift card.

We will reimburse you CHF 30 per hour for participating in an interview, also in the form of a BERNcity gift card. a BERNcity gift card.

There are no costs to you or your health insurance company for participating.

15 Liability

Although this study does not involve any foreseeable risk, the Bern Institute of Biomedical (BIHAM) of the University of Bern is liable in accordance with the statutory provisions for any damage that could arise in the context of this study. The BIHAM of the University of Bern has taken out liability liability insurance for this study with Basler Versicherung AG (Aeschergraben 21, 4002 Basel) in order to be able to cover liability in the event of a claim. in the event of a claim. The requirements and procedure for this are regulated by law. Should you damage as a result of participating in this study, please get in touch with the contact person listed in chapter 17 contact person.

16. funding

Part of the study is financed by the "Substance Use" department of the Bern Institute for Family Medicine (University of Bern). In addition, the participating municipalities contributed financially to the costs of planning and conducting the study.

17. contact person(s)

You may ask questions about participating in the study at any time. Please contact:

Prof. Dr. med. Reto Auer (study director)
Bern Institute of Family Medicine (BIHAM)
University of Bern
Mittelstrasse 43
3012 Berne

script@biham.unibe.ch
031 684 58 79 (Mon-Fri 8-17)

Declaration of consent

Written declaration of consent for participation in the SCRIPT pilot study

Please read this form carefully. Please ask if there is anything you do not understand or would like to know. Your written consent is required for participation.

**BASEC number:**	2022-00733
**Title of the study**	A randomized controlled pilot study on the regulated sale of cannabis in pharmacies *Official title: The Safer Cannabis - Research In Pharmacies randomized controlled Trial*
**Responsible institution**	Bern Institute of Family Medicine (BIHAM) University of Bern<br>Mittelstrasse 43<br>3012 Bern<br>Switzerland
**Place of implementation**	Canton of Bern
**Main examiner at the place of study**	Prof. Dr. med. Reto Auer

<u>Participating person</u>

Surname and first

name: Date of

birth:

- I have been informed by the undersigned investigator (study assistant) verbally and in writing about the purpose in writing about the purpose and procedure of the study as well as possible advantages and disadvantages. informed.
- I participate in this study voluntarily and accept the content of the written information given to me. written information given to me. I have had sufficient time to make my decision.
- My questions in connection with participation in this study have been answered. have been answered.
- I will keep the written information and receive a copy of my written consent form. declaration of consent.
- I agree that responsible experts such as study-internal monitors and persons from the ethics committee to access my unencrypted data for testing and control purposes. may inspect them, but under strict observance of confidentiality and data protection. data protection.
- I am aware that my health-related and personal data (and urine samples, if applicable) will only be urine samples) can only be passed on in encrypted form for research purposes for this study. can be passed on. The persons responsible for the study guarantee that data protection in accordance with Swiss standards.

- I can withdraw from participation in the study at any time and without giving reasons. The data collected up to the time of withdrawal (and urine samples, if applicable) will still be evaluated as part of the study.
- I will be informed of any results and/or incidental findings that directly affect my health. informed.
- I have been informed that the Bern Institute of Family Medicine (BIHAM) has taken out an insurance has taken out an insurance policy that covers any damage caused by the research project. attributable to the research project.
- I am aware that, in the interests of my health, my right to obtain study cannabis can be withdrawn.
- I am familiar with the study-specific safety regulations (Chapter 8) and will adhere to the requirements.
- I know that the distribution and sale of study cannabis is a punishable offense and that in the the right to obtain study cannabis may be withdrawn in the event of a repeat offense.
- I will be available for the study visits and keep the appointments. The questions I will answer the questions asked of me truthfully.

<u>Consent for participation in interview</u>

I agree that I may be selected as a participating person for an interview (possibly divided into up to three appointments) (please mark with a cross):

☐ Yes

☐ No

<u>Consent for bringing cannabis samples</u>

I agree that I may be asked to bring a cannabis sample (*not study cannabis*) to the study visit after *study cannabis*) to the study visit after 6 months (please mark with a cross):

☐ Yes

☐ No

<u>Consent for the collection of contact details of a relative and/or the family doctor</u>
<u>family doctor</u>

I agree that after 6 months a relative or my family doctor may be contacted to collect study data about me. my family doctor can be contacted in order to collect study data from me. if I am not able to do this myself (please tick):

☐ Yes →☐ Relative & GP☐ Relative only☐ GP only

☐ No

Place, date	Signature of participating person

Confirmation of the investigator: I hereby confirm that I have explained to this participating person the nature, significance and scope of the study. I confirm that I have fulfilled all obligations in connection with this study in accordance with the Swiss applicable law. Should I become aware of any aspects during the course of the study which

the participant's willingness to take part in the study, I will inform this person immediately.
inform this person immediately.

Place, date	Surname and first name of the investigator in block capitals<br><br>Signature of the investigator

Declaration of consent for further use of data and urine samples from this study in coded form

**BASEC number:**	2022-00733
**Title of the study**	A pilot randomized controlled trial of regulated cannabis sales in pharmacies *Official title: The Safer Cannabis - Research In Pharmacies randomized controlled trial*

<u>Participating person</u>

Surname and first

name: Date of

birth:

I authorize that my coded study data and urine samples, if I provide samples may be used for research purposes. The urine samples will be stored in a biobank of the University Hospital Unisanté in Lausanne and used for future, as yet undefined research projects for an indefinite period of time.

I understand that the data and samples will remain encrypted and that the key is stored securely. The data and samples can be transferred to other data and sample centers in Germany and abroad. biobanks for analysis if they meet the same standards as in Switzerland. in Switzerland. All legal data protection requirements are complied with.

Normally, all data and samples are analyzed as a whole and the results are published in summary form. If there is a result that is important for my health it is possible that I will be contacted.

I decide voluntarily and can withdraw this decision at any time. If I withdraw, my data will be archived in encrypted form and my urine samples, if I samples, will be destroyed. I will only inform the investigator at the study center and do not have to justify this decision.

Place, date	Signature of participating person

Confirmation of the examiner: I hereby confirm that I have given this participating person the nature, significance and scope of the further use of their data and, if applicable I have explained the samples.

Place, date	Surname and first name of the tester in block capitals<br><br><br>Signature of the tester

Evaluation of the group discussion of the participatory support group during the set-up phase of the SCRIPT study

The group discussion was conducted with cannabis users

SCRIPT
The Safer Cannabis – Research In Pharmacies randomized controlled Trial

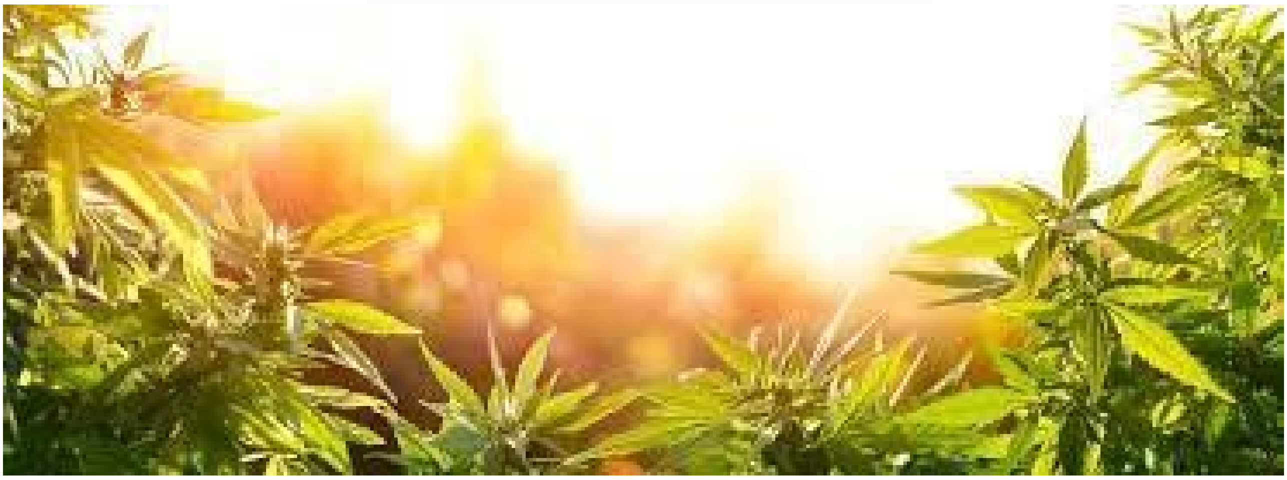

Beatrice Metry

Bern Institute of Family Medicine November

2023

Table of contents

1 Introduction 3
2 Methodological approach 4
2.1 Clarification of competence at the Cantonal Ethics Committee of Bern 4
2.2 Survey and evaluation procedure 4
2.3 Sample 4
3 Results from the group discussion 6
3.1 Information for the participants of the participatory support group 6
3.2 Safer use rules 6
3.2.1 Contents of the Safer Use Rules 6
3.2.2 Column with the links 7
3.2.3 Graphic on toxicology 8
3.3 Video by the Bern Institute of Family Medicine on the risks of cannabis use 9
3.4 Product video vaporizer 11
3.4.1 Video on cloud power from VITA 11
3.5 Media work and willingness to check the registration questionnaires 13
4 Outlook 14
5 Conclusion 15
6 Appendix 1: Group discussion questionnaire 16
7 Appendix 2: Safer Use Rules with standing graphic 19
8 Appendix 3: Safer Use Rules with horizontal graphic 20

1 Introduction

In the fourth quarter of 2021, individual interviews were conducted with the nine members of the SCRIPT study's participatory support group. In July 2022 and in February, June and November 2023, the group met once each for a group discussion on the premises of the University of Bern. The individual interviews and these group discussions were analyzed and the results presented in a report.
To date, the following reports have been produced and are available:

- Evaluation of the individual interviews on SCRIPT from March 2022
- Evaluation of the group discussion on SCRIPT from July 2022
- Evaluation of the group discussion on SCRIPT from March 2023
- Evaluation of the group discussion on SCRIPT from June 2023
- Evaluation of the group discussion on SCRIPT from November 2023 (present report)

The participatory support group for the SCRIPT study met for the fourth group discussion on November 16, 2023. Of the initial nine members of the participatory support groups, five cannabis users were present this time, who discussed the following topics under the moderation of Beatrice Metry (research assistant):

- Safer Use Rules
- Video on the facts of cannabis use
- Expectations of a product video e.g. vaporizer
- Other topics: Media work, feedback on the questionnaires from the registration process This evaluation report outlines the sample and then presents the results for the topics along the question route, which can be found in the appendix. The questionnaire can be found in the appendix.

2 Methodological approach

The various parts of the procedure are described below. All interviewees signed an informed consent before the individual interview (2021) and agreed to the interview and the subsequent group discussion and its evaluation by signing it. This assured them of anonymity.

2.1 Competence clarification with the cantonal ethics committee of Bern

On May 31, 2021, the concept for the qualitative accompanying research entitled "Use of a participatory support group consisting of cannabis-using adults as a supplement during the planning phase of the SCRIPT 2 project" was submitted to the Cantonal Ethics Commission (KEK) to check its competence. The CEC found that it was not responsible. This means that this research does not fall under Article 2, paragraph 1 of the Human Rights Act and that individual and group interviews with the selected target group can be conducted without submitting an application. After this decision[1]of June 20, 2021, the acquisition of participants was started.

2.2 Data collection and evaluation procedure

The data was collected through a group discussion along a defined question route over a period of two hours, digitally recorded and then transcribed.

The transcript was analyzed using MAXQDA software. The first step involved coding the text, followed by summarizing the individual statements and writing down the results.

2.3 Sample

All nine people who had already taken part in the individual interviews and signed the corresponding informed consent were requested for the group discussion. The appointment was arranged via doodle.
Six people registered for this group discussion and five people showed up for the group discussion. The following table provides an overview of the sample.

[1] BASEC no. Req-2021-00609, notification of the clarification of responsibility is available from 20.6.2021

	Individual interviews 4th quarter 2021	Group discussion 1 July 2022	Group discussion 2 March 2023	Group discussion 3 June 2023	Group discussion 4 Nov. 2023
**Number of people**	9	6	5	4	5
**Year of birth**	1957, 1975, 1990, 1991, 1993, 1999, 2000, 2001, 2000	1957, 1975, 1991, 1993, 2001, 2002	1957, 1991, 1993, 2000, 2002	1957, 1975, 2000, 2002	1957, 1975, 1991, 1993, 2000,
**Occupation**	Study (3)<br>Logistics (2)<br>Finance (1)<br>Photography (1)<br>Graphics (1)<br>Fitness (1)	Study (1)<br>Logistics (1)<br>Finance (1)<br>Photography (1)<br>Graphics (1)<br>Fitness (1)	Study (2)<br>Finance (1)<br>Graphics (1)<br>Fitness (1)	Study (2)<br>Photography (1)<br>Graphics (1)	Study (1)<br>Finance (1)<br>Photography (1)<br>Graphics (1)<br>Fitness (1)
**Salutation**	Woman 3<br>Man 5<br>non-binary 1	Woman 2<br>Man 3<br>non-binary 1	Woman 2<br>Man 3<br>non-binary 0	Woman 1<br>Man 2<br>non-binary 1	Woman 1<br>Man 3<br>Non-binary 1
**Nationality**	Switzerland 9	Switzerland 6	Switzerland 5	Switzerland 4	Switzerland 5

3 Results from the group discussion

The results are listed below along the question route. Numbers in round brackets indicate how many respondents answered in this direction. If there are no brackets after the statement, it is an individual answer. The paragraph in the MAXQDA file (A) in which the quote can be found is listed in square brackets.

3.1 Information for the participants of the participatory support group

At the beginning of the meeting, it was announced that the first study participants are expected to start the baseline visit in January 2024. Cannabis sales in pharmacies will therefore start at the end of January 2024.

Participants were also informed that a media release on the SCRIPT study would be published soon. Prior to the media release, the participants will be informed by email about when it will be published. This press release will also describe the registration procedure. The support group members were asked to register officially via the regular channel. This means that they register in the same way as all other study participants.

3.2 Safer Use Rules

The Safer Use Rules (see Appendices 7 and 8) were shown to the participants digitally as a PowerPoint slide. Initial reactions were: The information was clear (2), understandable and written in short sentences (2). Two voices were critical of the design. One said that the large amount of text made it look cumbersome, but the person pointed out that they did not know what information they could delete, as it was all important. The other person thought this slide looked like a leaflet. In order to make the document more appealing to the target group, two people expressed the idea of inserting a picture of a hemp leaf as a watermark in the background. And another person noted that practical tips in the event of an overdose could increase the motivation to read these recommendations.

3.2.1 Content of the safer use rules

In terms of content, four people criticized the information on the duration of effect of cannabis when consumed orally. Point *3* of the Safer Use Rules states: "Recommended pause time: a few minutes for inhaled form, up to one hour for oral use.". One person commented as follows:

> For me it would make sense if, especially in point 3 at the first performance mark, the hours were specified. This in the sense of smoked or vaporized maybe 3 hours of effect time and when consumed orally it is really much longer, 6-9 or even 12 hours. [...] It is potentially underestimated. [A-60]

Four of the participants agreed with this statement. All those present agreed that a chart similar to the one on toxicology would provide comprehensible information on the duration of effect of each form of consumption.

One person criticized the lack of differentiation between tobacco and cannabis types and two voices said at this point that a differentiation of filter quality was missing. The filters were discussed in more detail in connection with the graphic on toxicology (see section 3.2.3).

Two people said that they lacked information on how the time of day influences the effects of cannabis. The attendees explained their assumptions. A clear explanation of how the time of day could influence the effect of cannabis remained unclear.

One person stated that they lacked the information that the consumption of cannabis should be avoided in the event of physical complaints (e.g. headaches, colds). Two other people disagreed, saying that they would certainly use cannabis if they had physical complaints, even if it was only to relax the body. One voice commented as follows:

> I've also smoked weed when I had a cold [...] because it calmed my overall system. And I didn't have the feeling that it made the symptoms worse. It wraps everything up in absorbent cotton [...]. Of course you can say that smoking is not good if you have respiratory problems. But in the overall system it can have a positive effect. [A- 95]

The comprehensibility of the text (3), informative, factual and the fact that the decision remains with the reader (no paternalism) (2) were positively noted.

Furthermore, the question arose as to whether the cannabis products would be accompanied by an instruction leaflet. If so, this could be added under point 3:

> 'For further information, consult the package leaflet. [A-57] Another voice

expressed the following opinion:

> I find this colored scale mega impressive because it's such an eye-catcher. And it makes it clear what it means. It's not patronizing, but factual. I think that's good. It describes well what you can look at. [A-107]

3.2.2 Column with the links

The green column with the support services was rated as appropriate (4). One person said that it encouraged them to reflect on their own cannabis use. Another person said that they lacked information on the specific services offered by the respective institution. She commented as follows:

> It doesn't bother me that it's there. I would have no idea now. So, if I want to contact someone about something now, I realize that if I have a problem with normal smoking, then "stopsmoking" is there. And something about the study, then that's it. With the first three, I think there is zero information about when and for what which institution stands. I have the feeling that they simply wanted to do justice to all of them and mention them. But for me as a consumer who wants to use it for a specific case, I now have three addresses or three URLs. Why are there three and when do I choose which one? If I know you and you know these three, then you can tell me for your [problem] I would recommend this [address/institution]. But why three? There is very little or no information. It says anonymous. Aren't the others anonymous? [A-72]

One voice replied that only *infodrog* is open about what it stands for. This could be supplemented with "Swiss Coordination and Specialist Office for Drug Issues/Drug Consumption/Drug Matters. Another person added "Drugs and more". However, these ideas were not convincing. This is because it remains unclear how this selection came about and what distinguishes these organizations from others (see last quote).

There were also comments on the design of this column. For example, two people noted that the QR code for the SCRIPT study should be left-aligned for visual reasons. And the question arose as to why a QR code was only shown for SCRIPT; it would be consistent and practical for the readership to have QR codes instead of links.

3.2.3 Graphic on toxicology

One person spontaneously commented that this graphic was plausible and showed what he himself experienced (difference between smoking and vaping). Another person was surprised when they saw how harmful smoking with a filter was shown on the graphic. Filter quality was a relevant topic for everyone present. The question arose as to which filter quality (filter rolled out of cardboard, cigarette filter, activated charcoal filter) the statement on this graphic applied to (5). One voice expressed the following opinion about the filters:

> The only point of criticism is with the filters. Because, with regard to the activated carbon filters, the manufacturer mentions percentages of pollutant filtering. And I have the feeling, can I really believe that? I've already heard that if I use the [activated carbon filter], it's better [healthier]. That's why I would write it out again. [A-39]

Those present said that it was important to differentiate between the filter quality and that it was expected to specify which filter quality the statement in the graphic applied to. One voice said that she was confused by the illustration of the joints in the graphic. It said with and without a filter and the joint was always shown with a filter (see Figure 1).

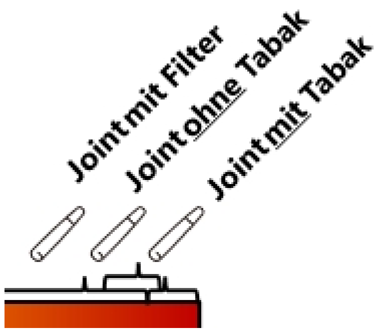

Figure 1 Joints from the graphic on the toxicology of cannabis use

When asked about the effect of the horizontal and vertical graphics, two respondents said that the vertical graphic was more dramatic, more memorable and made the danger clearer. The document with the horizontal graphic was perceived as prettier (3) and more appealing. Another person added that this document formed a unit. Another person said that the horizontal version would not need a cannabis leaf as a watermark. One person pointed out that too much movement in the background would impair legibility. This person put forward the idea of displaying a cannabis leaf to the left and right of the title "7 recommendations for safe cannabis use", which would create a connection and convey "smoking weed is ok, and here are a few more tips". [A-149] One person noted that it was open to them whether dramatic or pretty was right. Here is the quote:

> 'The question is, is dramatizing right or wrong. And I don't think that's clear. Because the other [vertical] is, they're going for deterrence. Maybe [...] less dramatizing is more intelligent. I don't think that's clear. [A-147]

3.3 Video by the Bern Institute of Family Medicine on the risks of cannabis use

The accompanying group was shown the video by Dr. Bartlomiej Niznik on the risks of cannabis use. The content deals with the toxicology, bioavailability and absorption rate of the active ingredients of cannabis in different forms of consumption. The video lasted 8 minutes and 32 seconds and was a Power Point presentation with a visible speaker at the bottom right.

Three people described their first impression as interesting, informative, learned something new (rectal use of cannabis), an expert who spoke, the facts were good, only the information on the duration of action or absorption speed with oral cannabis use was criticized, as the following quote proves and was confirmed by all those present:

> At times I had the feeling that it was not practically oriented. In other words, things that are not true from my point of view. For example, the refilling of oral consumption. So anyone who is already on it or has already had [cannabis] cookies would

> recommend to anyone: 'Take again after half an hour or an hour if you don't feel anything yet. [A-163]

> That is very dangerous. Because it can take from half an hour to four hours before you notice anything. [A-166]

The presentation of the information seems theoretical (3) and is reminiscent of a lecture (3). The high level of difficulty of the content (3), the reading (3) and the uniform way of speaking raised the question of the target group. Three people said that they missed the speaker's passion for the topic. Two other voices said that if they had been able to, they would have clicked off this video. When asked at what point they would have clicked away, one person said when the word *bioavailability* came up. They would not have understood bioavailability and there was no explanation. The second voice said the following about when they clicked away:

> What is it supposed to tell me? After a certain time you clicked off because there were too many unknowns. [A-184]

Another voice stated that they had mentally clicked off in the middle of the video and stopped listening. Two other people found the video interesting, they were able to follow the content and stayed attentive until the end.

Foreign words need a definition or an explanation, demanded those present (5). Three people stated that they did not understand the word bioavailability. Another person was confused between absorption rate and onset of action. There were slides with plus signs (e.g. bioavailability of consumption forms) and slides with plus and minus signs (e.g. summary of properties). Two people stated that they did not understand the meaning of these signs, as the following quote illustrates:

> Eben, I didn't read it that way at all. I thought, huh, why? I didn't understand what it was trying to tell me about this toxicology. Or the one with the minus sign. These colors came up and I thought, huh? [A-271]

One person noted that cannabis consumed with the vaporizer could be used a second time for baking or cooking, for example. In other words, multiple use is possible. This is called "already vaped but" (AVB) and is also available for purchase. She felt this information was missing from the video. As already mentioned in section 3.2.3, the filter quality was also an issue here, which should be received in a more differentiated way. Two people suggested differentiating the overdose of cannabis by form of consumption and cannabis product. One person said the following:

> I also think the topic of overdose could be explained a little more. I mean, not all overdoses are the same. Rain overdose has a different effect than an overdose

> Karisk. That you could go into it more. And what is an overdose from smoking pot and what is an overdose from eating? I think that's also different and also in terms of timing, the effect is different. And also the filters, that's also part of it. But the facts were good. As I said, you don't have to explain everything, but I think it's good that it was there. [A-264]

When asked how the video could be improved, one voice said that the first question should be about the communication channel. In other words, what information should be conveyed to whom and what form is suitable for this. Only once this has been clarified can work be done on the content. Two other people provided ideas on this. One voice mentioned an explanatory video as a possibility and the second voice recalled the animated paper clip from Windows and derived an animated cannabis leaf as a speaker. Another idea for improvement was a change of speaker (4), e.g. a charismatic hemp farmer (2) or a person with practical cannabis experience (2), who freely use colloquial language. The aim of a change of speaker would be to convey more practical experience and passion. A voice from the OFF, without a picture, would also be a variant, one person said. Two people thought that the slides could be made more visual, i.e. contain more images and less text. One person criticized the irregular formatting of the slides, e.g. the performance signs.

3.4 Vaporizer product video

Participants were asked what information they would like to find out about a vaporizer in a product video. The following aspects were mentioned: Advantages of vaporizing (e.g. multiple use see paragraph 3.3) (3), operating instructions (3), price (2), battery life, materials (2), in particular the origin of the materials and the environmental balance, adjustable heating possible and if so, with what effect on consumption (2), power cable or battery operation, information on maintenance of the device and information on the warranty. One person stated that they had no expectations of a product video.

When asked when the participants would watch a product video, they said the following: When they want to buy a vaporizer and get information (2), at the CannaTrade or to fill waiting time. One person stated that they did not watch any product or promotional videos.

3.4.1 Video on cloud power from VITA

The support group was shown a video by Fourtwenty. It introduces the Wolkenkraft vaporizer from VITA. The video lasted 2 minutes and 26 seconds and contained advertising for Fourtwenty, the grow and head store from Bern.

The effect of this video was perceived heterogeneously. One person said they would buy this vaporizer immediately, it was handy and the battery life was good for on the go. Two people thought the speaker seemed authentic and knew what he was talking about. Two other voices thought that the speaker seemed uncomfortable in this situation in front of the camera.

People also commented that the background music was pleasant (3), that they got to know a new product with the vape wool (2), that the short sentences were easy to understand (2) and that the video was edited, which was rated positively. One person commented that they had never wandered off or dropped out and were able to listen attentively until the end.

Two people rated this video as "bad". On the one hand, the video did not appeal to them and on the other hand, irrelevant things such as the instructions for use were mentioned, but other information was missing. One person commented that they would rather read through the facts.

The information that the participants would like to find out in a product video (see above) was only partially mentioned.

No one was bothered by Fourtwenty's advertising in the video (5), as it is a small, local supplier. If it were a retail chain, participants would find the advertising annoying (5). The participants expressed trust in Fourtwenty (4). Only one person stated that they did not know Fourtwenty before the video.

One person expressed concerns as to whether the University of Bern was allowed to use surreptitious advertising during a study. She formulated this as follows:

> 'Well, it is a university study. And I think it's sometimes double-edged when the university does surreptitious advertising for a store as part of the study. I think you can suggest vaporizers, but the question is, does it have to be a specific one? [...] So I don't know if that's ideal. I also have confidence in Fourtwenty, but I'm not sure whether it's really necessary in the university study to do this kind of surreptitious advertising. [A-376]

Another voice brought the judiciary into play with the question of whether the university is allowed to advertise certain products at all without making it clear how this selection was made. The following quotes support these statements:

> Are you as a university allowed to advertise a business and a type of vaporizer? [A-381]

> So you've done a consumer protection study and the seven best are allowed to post this video? I think that would be great, but then I would also like to have that communicated. That you've tested them all and these seven are good and that's why you're giving them the platform to advertise them. Then I have an explanation. But I would actually like this explanation. [A-385]

3.5 Media work and willingness to check the registration questionnaires

The media work is well received by the support group. Four of those present are prepared to help with media inquiries. The e-mail addresses of these four people are forwarded to Kathrin Bieri, SCRIPT coordinator. The inquiries are made directly by Kathrin Bieri.

A media release is forthcoming describing the enrollment procedure. From this date, online registration for participation in the SCRIPT study will be possible. To test the enrollment questionnaires, participants were asked if they would be willing to spend time on them. All those present (5) indicated that they would be willing to test the questionnaires and provide written feedback on a prepared form. Kathrin Bieri will send the five people a link, the access data and the feedback form.

4 Outlook

The next monitoring group meeting is planned for February 2024. Possible topics will be

- initial feedback on the study and sales processes,
- a demonstration of the impact of the monitoring group on the study protocol and
- a retrospective of the work within the monitoring group.

5 Conclusion

A ***media release*** is imminent. This will also describe the registration procedure. Interested parties can register from this point onwards. Monitoring group members have been informed that they must register through the official channels in order to participate in SCRIPT.

Respondents commented that the ***safer use rules*** were clear and easy to understand. In terms of content, the lack of information on filter quality and the information on the effect time of orally consumed cannabis, which was perceived as incorrect, were criticized. A graphic on the effect time similar to that of toxicology could support comprehensibility. The graphic on toxicology shows the danger impressively in a vertical orientation. In horizontal orientation, the graphic looks nicer and fits more smoothly into the document. The green column on the right-hand side of the page was found to be helpful by the majority.

The ***video from the Bern Institute of Family Medicine on the risks of cannabis use*** was described as interesting and informative. They had learned something new (rectal use of cannabis), an expert had spoken and the facts were good, only the information on the duration of effect or absorption speed with oral cannabis use was criticized. The information provided was perceived as theoretical and at a high level. The uniform way of speaking, the use of foreign words and the slides with plus and minus signs demanded (too) much attention. Respondents wanted the video to use colloquial language, cannabis experience and more passion for the topic.

The ***product video*** for VITA's Wolkenkraft vaporizer received mixed comments. What the participants did agree on was the fact that the expected product information was only partially mentioned in the video. The advertising by Fourtwenty was not perceived as disturbing. However, the question arose as to whether a university should be allowed to advertise a local provider and certain products in an official study.

Those present were motivated to click through the ***registration forms*** and provide written feedback and to participate in future ***media work***.

The next and final support group meeting is scheduled for February 2024.

.

6 Appendix 1: Group discussion questionnaire

Preparation Accompanying Group Nov. 16, 2023 18:30 - 20:30

Welcome, agenda, duration (5`)

Information from the research team (5`)

- Some participants will start the baseline visits in January 2024 and provide feedback.
- The implementation of the SCRIPT study *is expected to* begin at the end of January 2024 with the sale of cannabis in pharmacies.
- There will be a media release on the study soon. You will receive an e-mail when this media release is made. At the same time, you will be informed about how to register. Please register officially. You will then be selected manually from the registrations, as you can take part regardless of where you live.

Safer use rules→ show ppt and let it work (25`)

Please take some time to read through these rules.

- How do these rules affect you?
  Neutral, patronizing, readers are responsible, others know what is good for me, ...
- What do you read from them? What do you take away?
  - What is the most important message for you?
- What do you think of the content?
  - Is the content complete?
  - Is something missing?
    - If so, what?
- What do you think of the graphics?
  - What information do you read from it?
    - Is this graphic easy to understand?
  - Is this graphic appealing?

Video by Bartek on toxicology, bioavailability, absorption rate (40`)

Play video (9`)

- How does this video affect you?
- What do you take away?
- What did you understand?

- What do you understand by bioavailability?
- What do you understand by toxicology?
- What do you understand by absorption rate?

- Which elements do you not understand?
    - Why is a filter not enough?
- Are there any elements/information you would like to add here?
    - If so, which ones?
    - Please explain your answer.

Videos about vaporizers from Fourtwenty (30`)

Before I show you videos in which vaporizers and their handling are presented, I ask you:

- **What do you expect from product videos?**
    - What aspects of a vaporizer do you want to learn about in a product video?
    - What should the video focus on? What should not be missing under any circumstances?
    - When are product videos helpful for you?
    - On what occasions do you watch product videos?

I show video from Fourtwenty about vaporizing (5`) and about cloud power (3`)

- **How do you find these videos in general?**
  Comprehensible, appealing, informative, helpful, overloaded,

- **Do these videos seem authentic?**
    - ***If so***, what makes them seem authentic?
    - ***If not***, what is missing to appear authentic? How do you determine that is being "played"?
- **What do you particularly like? What do you notice positively?**
- **Do you see any disadvantages when vaporizers are presented in such (promotional) videos**?
    - If so, what are they?
- **Do you see advantages when vaporizers are presented with such (advertising) videos?**
    - If so, what are they?

If the participants do not make any statements about advertising, ask actively:

- **What do you think about the advertising in the videos?**
    - What effect does this advertising have?
    - Do you think advertising in the videos is ok?

        - If yes, why?
        - If not, why?
    - Should these videos be kept neutral?
        - If so, why?

Who of you is interested in media work? (5`)

- F. has already had an initial contact and can report back.
- A 2nd person is welcome to join in.

Who of you is interested in testing the online registration with all the questions? (5`) That means:

- Clicking through all questions and giving feedback per page/question set in a separate feedback form.
- The questions can be answered fictitiously= Test. The data is then destroyed.
- Duration: approx. 30 minutes
- Anyone who completes the online questions and returns the completed feedback form will be credited with 1 hour at CHF 30 (paid out with PBG money).
- You will have to repeat this procedure when you register. I will pass on

your e-mail address to Kathrin Bieri.

- You will receive the link to the questions and the feedback form from Kathrin Bieri by e-mail in the next 1-2 weeks.

Next and last session *will take place in February 2024. (5`)*

Expected topics will be:

- A retrospective on what you all contributed to the study protocol.
- Some thoughts on how the work in the monitoring group was for you.
- Initial feedback on study and sales processes.

7 Appendix 2: Safer Use Rules with standing graphic 7 Recommendations for Safer Cannabis Use

7 Empfehlungen für einen sicheren Umgang mit Cannabis

1 Wahl einer weniger schädlichen Konsumform von Cannabis

Die verschiedenen Formen des Cannabiskonsums lösen unterschiedliche Mengen und Arten von Schadstoffen aus.

- Joint, mit Tabak: Rauchen ist die schädlichste Cannabis-Konsumform. Tabak enthält in sich toxische Substanzen, die zu gesundheitlichen Schäden führen. Zudem hat das im Tabak enthaltene Nikotin ein starkes Abhängigkeitspotential.
- Joint, mit Filter: Der Filter bietet nur einen minimalen (Pseudo-)Schutz. Die meisten toxischen Substanzen gelangen durch den Filter hindurch in die Lunge.
- Vaporisator: Harz/Blüten werden um 200°C erhitzt. Es werden weniger Schadstoffe gelöst als beim Verbrennungsprozess in klassischen Joints.
- E-Joint: Mit einem E-Joint wird cannabishaltige Flüssigkeit um 200°C erhitzt. Es werden weniger Schadstoffe gelöst als beim Verbrennungsprozess in klassischen Joints.
- Orale Einnahme: Cannabis wird in unveränderter Form im Körper verabreicht. Schadstoffe durch Erhitzung von Cannabis werden vermieden.

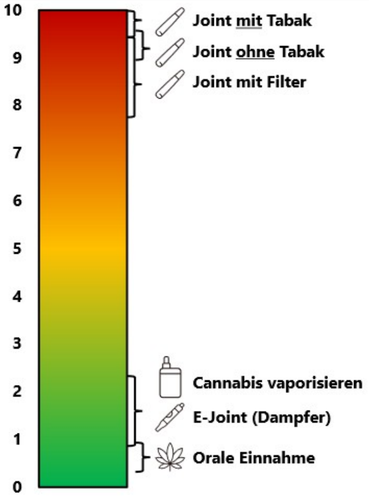

2 Tabak vermeiden

- Tabakrauch - nicht Nikotin - ist die wichtigste vermeidbare Ursache von Erkrankungen bei Personen, die Cannabis konsumieren.
- Falls Sie weiterhin Nikotin konsumieren möchten, wechseln Sie zu weniger schädlichen Konsumformen. (Nikotinersatztherapien, E-Dampfer, Nikotin Pouches)
- Lassen Sie sich in Ihrer SCRIPT Verkaufsstelle zu Tabakkonsum beraten.

3 Überdosierungen vermeiden

- Die Dauer bis die Wirkung von Cannabis gespürt wird, ist abhängig von der Konsumform.
- Cannabiskonsum durch Inhalieren führt zu einer raschen Aufnahme der Wirkstoffe über die Lunge.
- Bei oraler Einnahme ist die Wirkung verzögert und abhängig von Mahlzeiten und Tageszeit.
- Konsumieren Sie kleine Mengen und machen Sie Pause vor weiterem Konsum.
- Empfohlene Pausendauer: wenige Minuten bei inhalierter Form, bis eine Stunde bei oraler Einnahme.

4 Cannabis in der Freizeit konsumieren

- Cannabis beeinträchtigt Merkfähigkeit und psychomotorische Fähigkeiten.
- Beachten Sie die Wirkdauer von Cannabis, insbesondere vor Arbeit, Schule, Teilnahme am Strassenverkehr und Bedienen von Maschinen.
- Konsumieren Sie in einer Umgebung, in der Sie sich wohl fühlen.

5 Vermeiden von Cannabis in Kombination mit anderen psychoaktiven Substanzen und Medikamenten

- Der gleichzeitige Konsum von Cannabis und Substanzen wie z.B. Alkohol oder Drogen kann die Wirkung gegenseitig verstärken.
- Cannabis kann die Wirkung von Medikamenten beeinflussen. Lassen Sie sich durch eine Gesundheitsfachperson beraten, falls Sie Medikamente einnehmen.

6 Umfeld schützen, insbesondere Minderjährige

- Geben Sie Cannabis nicht an Dritte weiter.
- Lagern Sie Cannabis ausser Reichweite von Kindern, an einem kühlen, trockenen und luftdichten Ort.
- Schützen Sie Ihr Umfeld vor Passiv-Rauchen.

7 Hilfe holen bei Problem im Zusammenhang mit Cannabiskonsum

- Holen Sie sich Unterstützung, wenn Sie das Gefühl haben Ihr Cannabiskonsum gerate ausser Kontrolle.
- Holen Sie sich Hilfe, wenn Sie Entzugserscheinungen haben oder wenn Ihr Konsum die Arbeit, Schule oder das Sozial- und Familienleben beeinträchtigt.

Unterstützungs-angebote

www.safezone.ch
Online-Plattform für anonyme Suchtberatung

www.infodrog.ch
Schweizerische Koordinations- und Fachstelle

www.bernergesundheit.ch
Lokale Gesundheitsfachstelle Bern / Biel

www.stopsmoking.ch
Online-Plattform für Rauchstopp

SCRIPT Verkaufsstelle
Persönliche Tabak- und Cannabiskonsumberatung in Ihrer Apotheke

Studienkontakt:
info@script-studie.ch
031 684 67 79 (Mo-Fr 8-17);
Notruf (24h): 112
Prof. Dr. med. Reto Auer
Berner Institut für Hausarztmedizin (BIHAM)
Universität Bern
Mittelstrasse 43
3012 Bern

Safer Use Regeln | SCRIPT Cannabispilotprojekt | DE | V 1.2 | 07.11.2023

8 Appendix 3: Safer Use Rules with horizontal graphic

7 Empfehlungen für einen sicheren Umgang mit Cannabis

Safer Use Regeln | SCRIPT Cannabispilotprojekt | DE | V 1.2 | 07.11.2023

1 Wahl einer weniger schädlichen Konsumform von Cannabis

Die verschiedenen Formen des Cannabiskonsums lösen unterschiedliche Mengen und Arten von Schadstoffen aus.

- Joint, mit Tabak: Rauchen ist die schädlichste Cannabis-Konsumform. Tabak enthält in sich toxische Substanzen, die zu gesundheitlichen Schäden führen. Zudem hat das im Tabak enthaltene Nikotin ein starkes Abhängigkeitspotential.
- Joint, mit Filter: Der Filter bietet nur einen minimalen (Pseudo-)Schutz. Die meisten toxischen Substanzen gelangen durch den Filter hindurch in die Lunge.
- Vaporisator: Harz/Blüten werden um 200°C erhitzt. Es werden weniger Schadstoffe gelöst als beim Verbrennungsprozess in klassischen Joints.
- E-Joint: Mit einem E-Joint wird cannabishaltige Flüssigkeit um 200°C erhitzt. Es werden weniger Schadstoffe gelöst als beim Verbrennungsprozess in klassischen Joints.
- Orale Einnahme: Cannabis wird in unveränderter Form im Körper verabreicht. Schadstoffe durch Erhitzung von Cannabis werden vermieden.

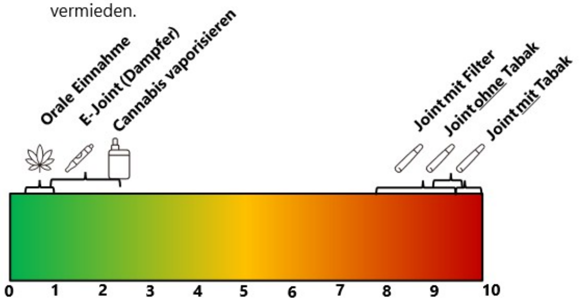

2 Tabak vermeiden

- Tabakrauch - nicht Nikotin - ist die wichtigste vermeidbare Ursache von Erkrankungen bei Personen, die Cannabis konsumieren.
- Falls Sie weiterhin Nikotin konsumieren möchten, wechseln Sie zu weniger schädlichen Konsumformen. (Nikotinersatztherapien, E-Dampfer, Nikotin Pouches)
- Lassen Sie sich in Ihrer SCRIPT Verkaufsstelle zu Tabakkonsum beraten.

3 Überdosierungen vermeiden

- Die Dauer bis die Wirkung von Cannabis gespürt wird, ist abhängig von der Konsumform.
- Cannabiskonsum durch Inhalieren führt zu einer raschen Aufnahme der Wirkstoffe über die Lunge.
- Bei oraler Einnahme ist die Wirkung verzögert und abhängig von Mahlzeiten und Tageszeit.
- Konsumieren Sie kleine Mengen und machen Sie Pause vor weiterem Konsum.
- Empfohlene Pausendauer: wenige Minuten bei inhalierter Form, bis eine Stunde bei oraler Einnahme.

4 Cannabis in der Freizeit konsumieren

- Cannabis beeinträchtigt Merkfähigkeit und psychomotorische Fähigkeiten.
- Beachten Sie die Wirkdauer von Cannabis, insbesondere vor Arbeit, Schule, Teilnahme am Strassenverkehr und Bedienen von Maschinen.
- Konsumieren Sie in einer Umgebung, in der Sie sich wohl fühlen.

5 Vermeiden von Cannabis in Kombination mit anderen psychoaktiven Substanzen und Medikamenten

- Der gleichzeitige Konsum von Cannabis und Substanzen wie z.B. Alkohol oder Drogen kann die Wirkung gegenseitig verstärken.
- Cannabis kann die Wirkung von Medikamenten beeinflussen. Lassen Sie sich durch eine Gesundheitsfachperson beraten, falls Sie Medikamente einnehmen.

6 Umfeld schützen, insbesondere Minderjährige

- Geben Sie Cannabis nicht an Dritte weiter.
- Lagern Sie Cannabis ausser Reichweite von Kindern, an einem kühlen, trockenen und luftdichten Ort.
- Schützen Sie Ihr Umfeld vor Passiv-Rauchen.

7 Hilfe holen bei Problem im Zusammenhang mit Cannabiskonsum

- Holen Sie sich Unterstützung, wenn Sie das Gefühl haben Ihr Cannabiskonsum gerate ausser Kontrolle.
- Holen Sie sich Hilfe, wenn Sie Entzugserscheinungen haben oder wenn Ihr Konsum die Arbeit, Schule oder das Sozial- und Familienleben beeinträchtigt.

Unterstützungs-angebote

www.safezone.ch
Online-Plattform für anonyme Suchtberatung

www.infodrog.ch
Schweizerische Koordinations- und Fachstelle

www.bernergesundheit.ch
Lokale Gesundheitsfachstelle Bern / Biel

www.stopsmoking.ch
Online-Plattform für Rauchstopp

SCRIPT Verkaufsstelle
Persönliche Tabak- und Cannabiskonsumberatung in Ihrer Apotheke

Studienkontakt:
info@script-studie.ch
031 684 67 79 (Mo-Fr 8-17);
Notruf (24h): 112
Prof. Dr. med. Reto Auer
Berner Institut für Hausarztmedizin (BIHAM)
Universität Bern
Mittelstrasse 43
3012 Bern

Evaluation of the group discussion of the participatory support group during the set-up phase of the SCRIPT study

The group discussion was conducted with cannabis users

SCRIPT

The Safer Cannabis – Research In Pharmacies randomized controlled Trial

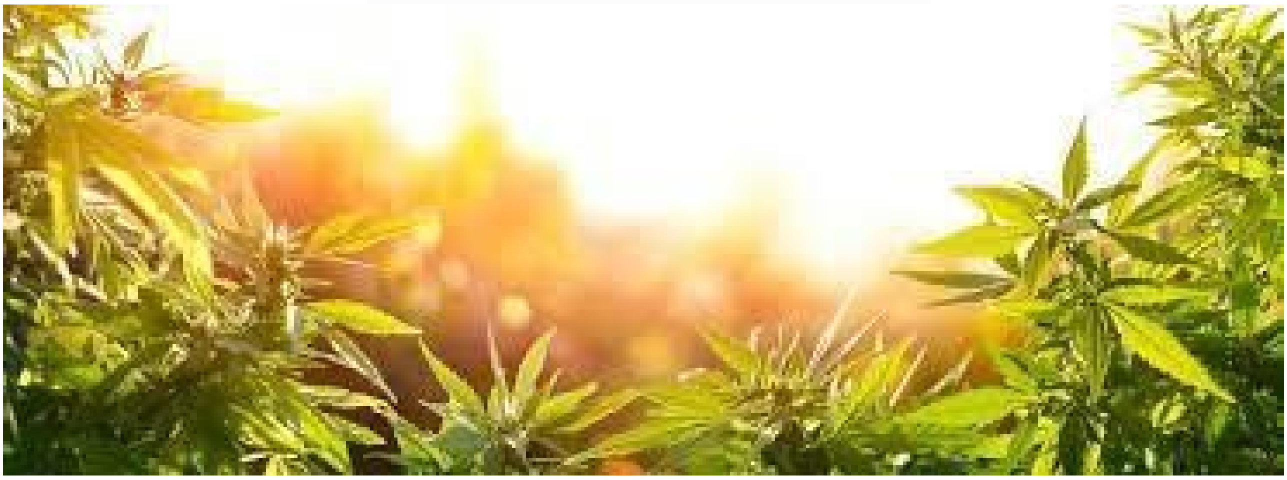

Beatrice Metry

Bern Institute of Family Medicine March

2024

Table of contents

1 Introduction 3
2 Methodological approach 4
2.1 Clarification of competence at the Cantonal Ethics Committee of Bern 4
2.2 Survey and evaluation procedures 4
2.3 Sample 4
3 Results from the group discussion 6
3.1 Co-writing an article on the work in the support group 6
3.2 Cannabis products 6
3.2.1 Feedback on the cannabis products 7
3.2.2 Feedback on the cannabis consumption devices 8
3.3 Findings from the support group for the research group 9
3.4 SCRIPT Intranet 11
3.5 Review of the work in the support group 13
3.6 Miscellaneous 15
4 Conclusion 16
5 Appendix 1: Group discussion questionnaire 18
6 Appendix 2: Findings from the support group 20

1 Introduction

In the fourth quarter of 2021, individual interviews were conducted with the nine members of the SCRIPT study's participatory support group. In July 2022, February, June and November 2023 and March 2024, the group met once each for a group discussion on the premises of the University of Bern. The individual interviews and these group discussions were analyzed and the results presented in a report.
To date, the following reports have been produced and are available:

- Evaluation of the individual interviews on SCRIPT from March 2022
- Evaluation of the group discussion on SCRIPT from July 2022
- Evaluation of the group discussion on SCRIPT from March 2023
- Evaluation of the group discussion on SCRIPT from June 2023
- Evaluation of the group discussion on SCRIPT from November 2023
- Evaluation of the group discussion on SCRIPT from March 2024 (present report)

The participatory support group of the SCRIPT study met for the fifth group discussion on March 6, 2024. Of the initial nine members of the participatory support groups, seven cannabis users were present this time, who discussed the following topics under the moderation of Beatrice Metry (research assistant):

- Collaboration on an article about the work in the support group
- Cannabis products
- Findings from the support group for the research group
- SCRIPT intranet for study participants
- Review of the work in the support group

This evaluation report outlines the sample and then presents the results for the topics along the question route, which can be found in the appendix. The questionnaire can be found in the appendix.

2 Methodological approach

The various parts of the procedure are described below. All interviewees signed an informed consent before the individual interview (2021) and agreed to the interview and the subsequent group discussion and its evaluation by signing it. This assured them of anonymity.

2.1 Clarification of responsibilities with the Bern cantonal ethics committee

On May 31, 2021, the concept for the qualitative accompanying research entitled "Use of a participatory support group consisting of cannabis-using adults as a supplement during the planning phase of the SCRIPT 2 project" was submitted to the Cantonal Ethics Commission (KEK) to check its competence. The CEC found that it was not responsible. This means that this research does not fall under Article 2, paragraph 1 of the Human Rights Act and that individual and group interviews with the selected target group can be conducted without submitting an application. After this decision[1]of June 20, 2021, the acquisition of participants was started.

2.2 Data collection and evaluation procedure

The data was collected through a group discussion along a defined question route over a period of two hours, digitally recorded and then transcribed.

The transcript was analyzed using MAXQDA software. The first step involved coding the text, followed by condensing the individual statements and writing down the results.

2.3 Sample

Eight people who had already taken part in the individual interviews and signed the corresponding informed consent were requested for the group discussion. The appointment was arranged via nuudel. One person withdrew from the support group as she no longer consumes cannabis.
Seven people had registered for this group discussion and appeared for the group discussion. The following table provides an overview of the sample during the individual interviews and the five accompanying group meetings.

[1]BASEC no. Req-2021-00609, notification of the clarification of competence is available from 20.6.2021

Table 1 Overview of the sample in the various survey moments

	**Individual interviews 4th quarter 2021**	**Group discussion 1 July 2022**	**Group discussion 2 March 2023**	**Group discussion 3 June 2023**	**Group discussion 4 Nov. 2023**	**Group discussion 5 March 2024**
**Number of of people**	9	6	5	4	5	7
**Year of birth**	1957, 1975, 1990, 1991, 1993, 1999, 2000, 2001, 2000	1957, 1975, 1991, 1993, 2001, 2002	1957, 1991, 1993, 2000, 2002	1957, 1975, 2000, 2002	1957, 1975, 1991, 1993, 2000,	1957, 1975, 1990, 1991, 1993, 2000, 2002,
**Sector Occupation**	Study (3)<br>Logistics (2)<br>Finance (1)<br>Photography (1)<br>Graphics (1)<br>Fitness (1)	Study (1)<br>Logistics (1)<br>Finance (1)<br>Photography (1)<br>Graphics (1)<br>Fitness (1)	Study (2)<br>Finance (1)<br>Graphics (1)<br>Fitness (1)	Study (2)<br>Photography (1)<br>Graphics (1)	Study (1)<br>Finance (1)<br>Photography (1)<br>Graphics (1)<br>Fitness (1)	Study (2)<br>Logistics (1)<br>Finance (1)<br>Photography (1)<br>Graphics (1)<br>Fitness (1)
**Salutation**	Mrs. 3<br>Mr. 5<br>non-binary 1	Mrs. 2<br>Mr. 3<br>non-binary 1	Woman 2<br>Mr. 3<br>non-binary 0	Woman 1<br>Mr. 2<br>non-binary 1	Woman 1<br>Mr. 3<br>non-binary 1	Mrs. 2<br>Mr. 4<br>non-binary 1
**Nationality**	Switzerland 9	Switzerland 6	Switzerland 5	Switzerland 4	Switzerland 5	Switzerland 7

3 Results from the group discussion

The results are listed below along the question route. Numbers in round brackets indicate how many respondents answered in that direction. If there are no brackets after the statement, it is an individual answer. The paragraph in the MAXQDA file (e.g. [A-56]) in which the quote can be found is listed in square brackets.
The attendees were welcomed and the agenda was presented. The agenda included the following six points: Introducing Anna Schibli (PhD student and SCRIPT study doctor), co-writing an article on the work in the monitoring group, cannabis products, findings from the monitoring group work, SCRIPT intranet and review of the monitoring group (collaboration) work.

3.1 Co-writing an article on the work of the monitoring group

Anna Schibli, SCRIPT study doctor and doctoral student, introduced herself to the support group. In her dissertation, she addresses the following question: To what extent did the support group's input influence the study protocol or intervention? She asked the members of the support group whether it was okay for her to write about this topic - the work of those present. The members of the support group said that they agreed. Anna Schibli went on to ask whether one or two people would be willing to contribute to the article. Two people volunteered to actively contribute. Anna Schibli went on to explain that it was possible to be listed as a co-author on the scientific article or to remain anonymous and only act as a silent contributor. In addition to the two interested parties, there are now two more people who will act as reserves and two other people who would like to read the article in advance and provide feedback if necessary.

Anna Schibli promised a first version for May/June 2024. She estimated the time required at one to two working days, but the commitment could be determined individually. Participants will be paid CHF 30 per hour worked. All support group members present will receive the article from Anna Schibli.

3.2 Cannabis products

The people present were allowed to take a closer look at the cannabis products. This meant that they received the cannabis products in the official sales packaging as well as the unpacked cannabis product in a jar. These jars have an integrated magnifying glass and the jars in which the flowers were shown also had a flap that could be opened to allow the product to be smelled. Three cannabis flowers and two hashish products were shown. At the same time, three vaporizers and an e-joint were presented to the accompanying group members and

to the group for a closer look. The products were then collected again. Only then were these products discussed.

3.2.1 Feedback on the cannabis products

In a first round, the people present (7) expressed their enthusiasm for the ***cannabis products on offer***. Five people said they thought the product demo jar with magnifying glass and smell tab was great, as illustrated by the following quote:

> 'It looks really professional. The packaging is similar to other CBD products. The product itself looks great. Even with this magnifying glass, I think it's great to look inside. [A-86]

A good half of the people present said that the products looked appealing (4). There were other individual comments. For example, one person commented that they had a VIP feeling because, as members of the support group, they were now able to see these cannabis products before all the other study participants. The fact that the cannabis products come from an outdoor production facility and are produced in Switzerland, as well as the selection of cannabis products, were also well received.

Three people emphasized that the ***flowers*** looked classy and that it was obviously a good production. The following quote underlines this statement.

> It [author's note: the flower] looked really classy and beautiful. [...] I would love to buy this at the kiosk. [A-138]

One person remarked that the intense lemon scent of one product did not appeal to her.

Three people agreed that the hashish ***products*** looked like pressed pollen. This was formulated as follows:

> 'Exactly, it's not really hash, it's pressed pollen. [A-133] It does look like pressed
>
> pollen, definitely. [A-136]

One voice remarked that the product demo jars for the hashish products lacked the smelling tab, as the following quote shows:

> I still think that with the hash, you could see if you could have a tab there too, so that you could still smell it. [A-88]

The ***packaging*** was described by three people as appealing or professional. The following quote confirms this statement:

> 'I think it's nice that there are no horrible pictures on the plastic packaging, like we know from cigarette packaging. It would have been possible that

> that this could also look like that. But it's nice that it looks so clean. [A-87]

Two people criticized the link behind the ***QR code*** printed on the packaging. This leads to the official SCRIPT study website. It would be useful for consumers if the QR code led directly to the product description. Another person said that the information on the packaging was easy to find.

The ***prices*** of cannabis products were generally perceived as high (4). One person replied that the product quality and safety of the study cannabis products on offer had its price. Another person underlined this with their current experience on the black market, as the following quote shows:

> Yes, it does. And now it's also dangerous with all the spray hit that's on offer. So [I] have already caught some of it myself and it's not funny at all. These synthetic cannabinoids that they have. They use CBD in China, big fields where they go over it with a spray gun. And that's dangerous. There's a lot of [THC] on one flower and almost nothing on the other flower next to it. [A-116]

Two people stated that they would continue to buy cannabis products from their friends in the future, especially because they were cheaper and of good quality.

Two people stated that the pricing was contradictory and tempted customers to buy the products with the highest THC content because the THC unit was the cheapest.

3.2.2 Feedback on the cannabis consumption devices

+ airzier air max

In addition to the cannabis products, those present had the opportunity to examine two vaporizers (Wolkenkraft Vita and Volcano Mighty) and an e-joint (OBY Aspire).

Four people stated that they already had experience with vaporizers and owned a device themselves. Three of these four people said they owned a Volcano Mighty and two of these people used it regularly. One of these people said the following:

> Yes, I use it a lot. [...] I think smoking a joint is one of the best things. But after a while I noticed that smoking too much was bad for my lungs. And yes, in the meantime I've upgraded so that I have a Volcano at home, which is a super device, but it's tied to home, i.e. to the electricity. And when I'm out and about, I have the Venti so that I can use it when I go somewhere. [A-167]

One person expressed skepticism about vaporizers at the beginning of the session. While actively looking at the devices and talking to the other members of the support group, this person changed their mind, which is reflected in the following quote.

> Because the last time, I remember, I made fun of these vaporizers. Because it annoys me that it's connected to electricity and always has to be charged. But now this little one, the Wolkenkraft Vita with wood. That was my statement here: "Aha, that's probably the next thing I'll have to deal with these [vaporizers] and then maybe (try out) this Wolkenkraft. And this big device [Volcano Mighty], well, I understand what you [other support group member] are saying. And on the one hand, it makes me happy. But I'm going to say, for the rustic stoner feeling, it's almost just...// When I talk to you [other support group member] like that, it's a bit too high end for me. I can see the point of doing it. But for my stoner ritual, when I first have to load a device and program it...// Maybe that's just the modern world, how health is promoted today. But it hasn't yet arrived in my stoner existence. But, yes. But with this one (cloud power) I'll give it a try. [A-170]

The e-joint was met with skepticism by some of the people present (3). One person said that they would refrain from trying an e-joint because they are cheap, disposable products. Another person countered this. Only the coil is replaced and the liquid refilled (change or refill the pod). The same person noted that with the pods offered in the SCRIPT study, the consumer is tied to this device. With liquids such as those offered with e-cigarettes, the pod could be refilled by the user, which would give product freedom.

3.3 Findings from the support group for the research group

The members of the support group were asked which of their feedback to the research group they thought was important. They often determined the importance based on whether their input was taken on board and implemented by the research team. The first step was to collect verbally what the people present could think of off the cuff. In a second step, they were presented with a list (6 Appendix 2) and asked to tick the five most important aspects for them individually. The results are presented in this order.

Many individual comments were made in the ***open discussion***. On

sales and the salesroom:

- Sales staff who have specialist knowledge (2).
- The sales process in the pharmacy
- A separate consultation room in the pharmacy, if the customer wishes.
- Product demo glass, so it is possible to smell and look at the products:

- Influence on the product selection

  - Hashish has been added to the product range
  - Cannabis saline suppositories were not included
- The packaging is kept neutral.

Hazard overview:

- The activated charcoal filter has been included

One person commented that the inclusive gender form had been included in the documents and texts on the SCRIPT website at the suggestion of the support group, which was a positive development.

The results from the ***individual written survey*** (see Appendix 2) are presented below in numerical order. Additional written comments from the interviewees can be found directly under each aspect listed.

- Not only cannabis flowers, but also hashish in different varieties (5)
  - Comment: Pressed pollen is more likely to be what is offered here.
- Salesperson should offer competent advice (expert information, list of varieties, prices) (5)
  - Comment: Conveys security; if possible, the salesperson should have tried the products
- Products should be visible and ideally also smellable (4)
  - Comment: Hash should also be smellable.
- Appearance of the specialist (likeability, tone) is important (4)
  - Comment: Building trust is important.
- Possible sales options apart from pharmacies: cannabis social clubs, CBD stores, neighborhood organic store, tobacco store. (3)
- Transparency on ingredients, strain, effect, THC/CBD content. (2)
  - Comment: The description of the effect could be better.
- Cannabis sales by Contact [Foundation for Addiction Support. Author's note] not attractive because stigmatizing (2)
- Stop smoking advice only on request, unobtrusive (2)
- Outdoor and indoor products of good quality
- No more than CHF 10/gram
- Organic quality appealing, price of flowers and hashish rather at the upper limit.
  - Comment: The price is too high at the upper limit. The price should be between CHF 5 - 10/gram.
- It is important that every member of the research group has smoked cannabis at least once.
- Mouth spray and creams interesting, suppositories not interesting
- Agree with the declaration of consent

- GP as contact person is viewed critically.
- Adaptation to safer use flyer: Oral effect time, position of the graphic.

The aspect "THC values with a maximum of 18% are sufficient for flowers" was provided with the following comment: Lower [THC levels author's note] could also be good.

3.4 SCRIPT Intranet

The SCRIPT intranet for study participants was rated as good by all those present (7). The interviewees were particularly positive about the fold-out recommendations for the safe use of cannabis (7). In this way, readers can decide for themselves which topic they would like more information on (7). The site was clearly structured (7), understandable and simple (2), informative (2), inviting, beautiful, sec, were other words used by the members of the support group to describe the intranet. A vote on the intranet that supports the above statements:

> I find it very clear, [...]. It is simple. I think it's good. I think it's really good. It is simply structured, you can check very quickly what you can find where. You can see the products clearly. They have listed them nicely. [279]

Two people said that all the information they needed was available.

The information under the Ritter ***Safer Use recommendations*** gave rise to the most discussion. Three people were pleased with the aspect "Choose a less harmful form of consumption", as the activated charcoal filter was included and presented. One person complained that the activated charcoal filters were not better presented. The person said that they thought it was important that cannabis users were made aware that it was healthier to smoke with a decent filter than without one. With the current presentation under the safer use rules, the study missed the opportunity to make this aspect clear.

In general, the people present (7) emphasized that they would like more in-depth information on the individual questions or aspects. Three people complained that the word "overdose" had negative connotations. They did not name an alternative to the word "overdose". Furthermore, respondents noted that under the aspect "Avoid combining cannabis with other psychoactive substances and alcohol", psychoactive substances were not addressed at all (3). The following quotes clarify what is meant.

> I find this even more exciting. You talk about psychoactive substances and drugs. But the psychoactive substances are not mentioned at all. [A- 349]
>
> I'm sure that can be embellished a bit more under the respective points. Otherwise, I think it's a good overview and a good insight. It is not overloaded.

> But if you open up the plus, especially for psychoactive substances, that you perhaps also list the drugs that are particularly .../ Or mixed use can be particularly difficult, be it with uppers and downers. [A-351]

The information under mixed use is clearly too scarce (4). Only the combination of cannabis and alcohol (3) requires more information, as it depends on the order of use, as the following quote shows:

> Well, if you ask me like that, I think it's rather very, very skimpy. I don't know, but for example the alcohol thing, that can be elaborated on. I think there's always a big difference between drinking alcohol first and then smoking another one, and having smoked one and then drinking alcohol. [A-333]

The same applies to the combination of cannabis and medication or drugs, two other voices added. Here, too, another aspect was missing, namely: What can I do if I have had mixed consumption (2)? With this meagre information, there was a high probability that people would turn to the internet for guidance. The vote to refer to suitable websites instead of filling the SCRIPT intranet with more information was supported by all those present. Possible sites mentioned were eve & rave[2], Contact[3]and Saferparty[(4)].

Four people said that an addition to the overdose would be helpful with the question: "What can I do in the event of an overdose?" And at this point list behavioral options and tips that can help in the event of an overdose. The respondents mentioned orange juice, vitamin C, showers and fresh air. One person mentioned the idea of publishing testimonials from those affected on this aspect here. Another person thought the title should be:
"Avoid overdosing when taking orally", as the two aspects listed only referred to oral intake. Another person countered that the field could also be opened up and supplemented with points for overdosing on weed.

One person commented that a "Feedback to the study management" button was missing so that they could submit observations. The intranet also lacked an option to ask questions or a link to the study contact.

(2) https://www.eve-rave.org/drogen-abc/or https://eve-rave.ch/Forum/
[3]www.contact-suchthilfe.ch
[4]https://www.saferparty.ch/

3.5 Review of the work in the support group

When asked what it was like for the people present to be an active part of a study, six people stated that it ***was*** an interesting, good ***experience***, especially to see that they, as stoners, had an influence on the study design, as the following quotes show:

> I never thought I would do something like this. I really didn't. Never. Even with the thought: I don't know what I can say about this. I don't know, I'm just a little stoner. But it was super exciting. To see for once that it does something. [A-397]

> It's really cool that people like that were able to influence the whole thing. That not only people who don't have a plan were asked. But people who actually smoke weed were asked. They are the ones who have a clue. And I think that's cool. [A- 411]

The interviewees (5) also stated that they enjoyed getting to know other stoners from different backgrounds. Four people said that they felt they were making (cannabis) history by participating in the support group. Two of the people present stated that they had been waiting for this moment for eight years. They had already registered for the first call for proposals in 2016 and had remained on the list. Two people also said that weed brings people together and that this was noticeable in the support group work. One person said that working in this group had given them the opportunity to get to know a new perspective on studies, namely as a participant. Other individual comments included: the number and spacing of the support group meetings was just right; it was cool that the cannabis products were shown; the discussion was informative. Furthermore, one person said that she found the experience and the differences between individual interviews and group discussions exciting, as the following quote shows:

> [...] the first time, we still had an individual interview and there you just say your stuff. And here [group discussion, author's note] it's mega blatant. You say something and at first it's a bit restrained and then suddenly you're discussing a lot. [...] There's a huge difference between a one-to-one conversation and being in a group and having a lively discussion about something. I think that's a really big difference and really cool. [A-427]

Most of the people present (5) said that they perceived the ***atmosphere*** during the sessions as peaceful and respectful. They attributed this to the fact that the illegality of cannabis use automatically created a sense of solidarity, a close-knit community between like-minded people. Three people also emphasized that the neutral attitude, openness and respect of the non-potheads involved (moderator, research group) had a positive effect on the atmosphere.

The interviewees cited decency and mutual respect (5) as ***unwritten rules*** within the support groups, which was demonstrated by allowing each other to make excuses (3) and also by accepting other opinions (3). One voice summarized it like this:

> 'Letting each other finish. Not trying to convince someone of your own opinion. Instead, everyone is allowed to have their opinion and this is also justified. [A-445]

The support group members were asked how they ***explain to outsiders*** what they do in this support group. Five people stated that they would take part in group discussions in which they would contribute their experience as cannabis users and in this way support the research group in an advisory capacity with regard to the study design. Two people stated that it was about the legalization of cannabis products and one person added that it was about health observations and what cannabis does to users.

The participants were also asked whether their ***attitudes towards cannabis*** and cannabis use had changed during their work in the support group. All respondents stated that their basic attitude towards cannabis and cannabis use had not changed during their time in the support group. However, one person stated that their consumption had changed during this time, as less cannabis was consumed in their environment. One person stated that they were vaping more, as this was better for their health. Another person noted that the discussion about alternative forms of consumption encouraged them to try a vaporizer and another person said that they were interested in e-joints, as the following quote shows:

> 'I've dealt with health before and started vaping before. I was already familiar with it. It made me a little bit curious about these e-liquids. So to the e-joint. [A-218]

Another person said that they thought it was cool that the focus of the study was on promoting health ***health promotion*** (healthier form of consumption) instead of abstinence as before. One person

noted the following:

> We are mega well balanced. Men, women, age, consumption-wise. A lot of consumption, less consumption, strong consumption, weak consumption. I think it's this mixture that has made it possible for us all to find something here (product range) that suits us. [A-417]

3.6 Miscellaneous

One person commented on the documents received in preparation for the first visit. It stated that the study cannabis may only be consumed by the person enrolled in the study. The person commented that this was unrealistic, as smoking weed is a social act and very few people smoke alone. They would maintain their consumption habit even with the study cannabis and smoke weed with other people.

Another person remarked that she had the following idea for society:

> Now they're all beating their heads in because of this AHV story. Legalize cannabis, tax it properly and we've saved the AHV. [A-419]

Two questions remained unanswered for the support group members. One was how many pharmacies they would be allowed to buy cannabis from. And the second question: Do the pharmacies have the devices listed on the SCRIPT intranet site on offer at all times?

When asked what they would have ***liked differently*** in the support group work, one person said that more people in the support group would have been interesting. The fact that no more people could be found surprised one part of the group (3). The other part of the group (4) could understand this, as they had people in their circle of acquaintances who were currently remaining anonymous with their cannabis use, as they feared disadvantages (e.g. at work). One person remarked that the participation in the support group could have been advertised. Ideas for putting up flyers were: Hemp store, GP surgeries, specialist agencies, testing center for cannabis.

4 Conclusion

The members of the support group agreed that Anna Schibli should write an ***article*** about the work of the support group. Two people were interested in actively contributing to the article and others indicated that they would be willing to give feedback on the article.

The people present were enthusiastic about the ***cannabis products*** and the product demo jars with magnifying glass and smell tab. The cannabis flowers looked classy. The hashish looked more like pressed pollen and not like real hashish. The prices of the cannabis products were generally rated as high. The pricing was perceived as contradictory. It encourages customers to buy the products with the highest THC content because the THC unit is the cheapest.

When asked which ***findings from the support group*** they thought were important ***for the research group***, the following five findings were mentioned the most:

- Not only cannabis flowers, but also hashish in different varieties
- Salesperson should offer competent advice (expert information, list of strains, prices)
- Products should be visible and, in the best case, also smellable
- Appearance of the specialist (likeability, tone) is important
- Alternative points of sale apart from pharmacies: Cannabis Social Club, CBD store, neighborhood organic store, tobacco shop

The ***SCRIPT intranet*** for study participants was found to be well and clearly structured by all those present. The interviewees were particularly positive about the fold-out recommendations for the safe use of cannabis. In this way, readers can decide for themselves which topic they would like more information on. In the safer use recommendations, the respondents wanted more in-depth information on the individual questions and aspects, as those who clicked on the "plus" wanted more information than was currently available below. This could also b e included with a link to recognized specialist sites such as eve & rave, Conact and Saferparty. Furthermore, the interviewees noted that, from the aspect of

"Avoid combining cannabis with other psychoactive substances and alcohol" does not mention psychoactive substances at all; this should be added. The information on mixed use is also too brief. Furthermore, the following questions and answers should be added to this page section: What can I do if I overdose? What do I do if I had mixed consumption? A button "Feedback to the study management" as well as a link to the study contact would also be desirable at this point.

The support group members stated that ***working in this support group*** had been an interesting experience for them. They were particularly moved to realize that they could influence the study design with their experience of cannabis use. They found the atmosphere within the support group meetings to be pleasant and respectful. They stated that they felt

also treated with respect by non-potheads (moderator, research group). Their attitude towards cannabis and their cannabis use had not changed as a result of their work in the support group. However, there are people who want to vape or try vaping more often, as this form of consumption is better for their health.

5 Appendix 1: Group discussion questionnaire

Preparation for final PBG meeting

Wednesday, March 6, 2024 18:30 - 20:30

Introduction, overview of the topics (5`)

- Anna Schibli Article on PBG work in SCRIPT (15`)
  - Topic: Impact of the results from the monitoring group on the study design of SCRIPT
  - Anna Schibli is happy if 1-2 people contribute to the article. Anna explains what this means.
    - Who of you is working with us?

- Present cannabis products and devices (25`)
  - Get reactions to products
    - What you like / dislike
    - What about the products meets your expectations?
    - What about the products differs from your expectations?
  - Get reactions to the devices
    - Which of you already have experience with one of these devices?
      - If so, which ones?

- Findings from the PBG work (20`)
  - You took part in the individual interview in 2021 and then in 4 support group meetings. What do you think are the most important insights you contributed? → Collect on flipchart or on ppt
  - Distribute Anna Schibli's list of topics: Look at this list. Cross the five most important topics for you.
  - Has your attitude towards cannabis and cannabis use changed during this time? If so, how?

- Intranet SCRIPT (15`)
  - Introduce page (participant, PW cannabis1000→ without space)
    - How does this page affect you?
    - What is missing? / What addition would be helpful for you?
    - What do you think is too much on this page? Why?

- Review of the PBG work (30`)
  - As of today, you have taken part in five support group meetings. What was this work like for you?
    - What made your collaboration special?
    - What unwritten rules did you follow to make this collaboration work?
    - What could a fly on the ceiling have observed if it had been watching us in the support group sessions?
    - How would you describe the atmosphere during the support group sessions?
    - Was there anything about the support group sessions that you would like to have differently next time?

- Note on group discussion by Marie-Noëlle McGarrity, Addiction Switzerland, 26.3.24 18:30 - 20:30
  - Bring printouts! Closing

and farewell (5`)

6 Appendix 2: Findings from the support group

Findings from the individual interview and the monitoring group meetings from 2021-2023

Individual interviews

- Not only cannabis flowers, but also hashish in different varieties
- Outdoor and indoor products of good quality.
- Salesperson should offer competent advice (expert information, list of varieties/prices).
- Separate consultation room for sales on request.
- Transparency with regard to ingredients, variety, effect, THC/CBD content.
- No more than 10/gram.

Group interview 1

- THC values with a maximum of 18% are sufficient for the flowers.
- Organic quality appealing, price of flowers and hashish rather at the upper limit.
- Somewhat skeptical about e-liquids, but most would give them a try.
- Products should be visible and, in the best case, also smellable.
- Open to a prevention discussion, but not intrusive. Flyers and information available at a low threshold.
- Appearance of the specialist (likeability, tone) is important.
- Important that everyone in the research group has smoked cannabis at least once.

Group interview 2

- Oral sprays and creams interesting. Suppositories not interesting.
- Possible sales options apart from pharmacies: cannabis social clubs, CBD stores, neighborhood organic store, tobacco store.
- Cannabis sales by Contact not attractive because stigmatized.

Group interview 3

- Agree with the declaration of consent.
- Website fulfills the purpose.
- GP as contact person is viewed critically.
- Stop smoking advice only on request, unobtrusive.

Group interview 4

- Adjustments to safer use flyer: Oral effect time, position of graphic.
- Video by research member on bioavailability etc. too boring.
- Videos from 4/20 basically likeable, but too unprofessional for a university.
